# *De Novo* characterization of transcriptomes from two North American *Papaipema* stem-borers (Lepidoptera: Noctuidae)

**DOI:** 10.1371/journal.pone.0191061

**Published:** 2018-01-24

**Authors:** Sara J. Oppenheim, Wiebke Feindt, Rob DeSalle, Paul Z. Goldstein

**Affiliations:** 1 Sackler Institute for Comparative Genomics, American Museum of Natural History, New York, New York, United States of America; 2 Stiftung Tierärztliche Hochschule Hannover, ITZ, Division of Ecology and Evolution, Hannover, Germany; 3 Systematic Entomology Laboratory, USDA, National Museum of Natural History, Washington DC, United States of America; National Institutes of Health, UNITED STATES

## Abstract

Stem-borers in the genus *Papaipema* (Lepidoptera: Noctuidae) range from highly polyphagous agricultural pests to specialists on more than 20 families of flowering plants, many of them highly toxic. *Papaipema* is the largest genus of noctuids endemic to North America and provides an excellent study system for the evolution of noctuid host plant use. To improve the availability of genomic resources for such investigations, we performed *de novo* transcriptome sequencing and assembly for two specialist *Papaipema* with unusual larval hosts: *P*. *speciosissima*, which is associated with ferns, and the undescribed *P*. “sp. 4,” which is associated with bamboo. The resulting transcriptomes were similar in terms of completeness, gene count, and gene identity, but we identified some 8,000 genes (~17% of each transcriptome) not shared between the two species. While some of these have identifiable orthologs in other Lepidoptera, ~5% of each transcriptome consists of species-specific genes. We examine the function of these genes and find that almost half have retrotransposon-related functional domains. The potential role of species-specific genes is discussed, and the expansion of certain retrotransposon families in *Papaipema* is examined.

## Introduction

The majority of available lepidopteran transcriptomic data are from agricultural pests and most in the Noctuoidea, which comprises the most species-rich and economically important lepidopteran superfamily [1]. Although pest species represent a small fraction of the taxonomic, ecological, and behavioral diversity of Noctuoidea, our understanding of their biology is enhanced by transcriptomic data from related species. The use of modern genomics tools to address longstanding questions about the evolution of insect host plant use [2–8] depends critically on sampling the spectrum of host use behaviors and syndromes represented throughout the order. In particular, greater sampling of species with internal feeding habits (endophagy) is needed, as it represents the condition of the first (lower glossatan) large radiation of moths [9], and has re-evolved independently in only a few more recently derived groups of larger moths.

*Papaipema* (Noctuidae: Noctuinae: Apameini) is a genus of stem- and rhizome-borers and the most species-rich noctuid genus endemic to North America. They include both highly polyphagous species, including the stalk borer *P*. *nebris* (Guenée) and specialists on an unusually diverse array of host plant families [10]. Several species of *Papaipema* are associated with chemically defended plants, including ferns (Pteridophyta), sometimes considered to have low herbivore loads, pipevines (Aristolochiaceae) [11], and umbels (Apiaceae), all of which figure prominently in the literature regarding co-evolutionary “arms race” scenarios [12]. As a whole, the noctuid tribe Apameini, to which *Papaipema* and its relatives belong, exhibits an array of larval feeding habits that correspond at least in part to variation in feeding habit and diapause [10].

As borers in the roots, rhizomes, and stems of their host plants, *Papaipema* caterpillars exemplify habits that coincide with graminivory (grass-feeding) in numerous insect groups (9). Pinpointing independent origins of internal feeding bears on our understanding of diet breadth to the extent such behaviors reflect either an escape from natural enemies or an avoidance of toxic plant defensive compounds (allelochemics) that are more concentrated in external tissues [13, 14].

As a step towards developing annotated genomic data for apameine noctuids, we generated transcriptome profiles of two *Papaipema* species. Our goal was not to compare expression levels, but rather to begin compiling a catalog of the genes present in each species. We selected two *Papaipema* species associated with plants relevant to the chemical ecology of plants and insect herbivores: The fern specialist *Papaipema speciosissima*, and an undescribed Poaceae (grass) specialist, *Papaipema* “sp. 4,” associated with native bamboo (*Arundinaria gigantea* (Walter) Muhl.). Fern feeders are of interest because strict stepwise co-evolution (*sensu* Ehrlich & Raven (1964)) is unlikely to have occurred among their herbivore specialists and because it has been asserted that, in ecological terms, ferns bear low herbivore loads relative to associates of other commonly encountered plant groups. Given the age and apparent evolutionary stasis of ferns [15], pteridivorous insects can only have colonized ferns well after their underlying chemical architecture was in place [16]. Grass feeding, in contrast, is prominent in most of the major groups of borers within the higher Lepidoptera (Goldstein et al., in prep.). Although much younger than ferns, Poaceae species (including sugarcane, maize, rice, wheat, barley, sorghum, oats, and millet) not only dominate numerous terrestrial landscapes but were among the first plants to be domesticated by humans [17] and currently account for the majority of agricultural crops by volume, acreage, and economic value. Examining the rapid diversification of specialized feeding habits will ultimately shed light on the molecular genetic bases of diet breadth, and the degree to which its modulation results in origins of pest species and outbreak behaviors.

The use of genomic data to address questions about the evolution of lepidopteran host use is hampered by the available pool of annotated genes with known functions. The evolutionary distance between model organisms with well-annotated genomes and any given species of interest to evolutionary biologists can be vast, hence many newly-sequenced genes lack readily identifiable homologs. The abundance of so-called orphan genes (sequences with no significant similarity to known proteins [18]) further complicates *de novo* analyses. In the early days of high-throughput sequencing, the occurrence of orphan genes was thought to reflect the limited taxonomic coverage of available genomes, and some previously orphan genes have indeed been “de-orphanized” as more taxa are sequenced. However, even as the number of sequenced organisms has grown, so has the number of orphan genes [18]. One of the goals of comparative genomics is to identify the genes that differ between closely related species, but identifying and annotating orphan genes found in a single species (species-specific genes, SSGs), is challenging. In this report, we use phylogenetic clustering to identify SSGs in two *Papaipema* species and compare the functions of these genes to those of orthologous common to both species. Because gene disruption and duplication via retrotransposition is an important mechanism in the emergence of orphan genes [19–21], we also examine the abundance of transposable element signatures in SSGs and orthologs.

## Materials and methods

### Transcriptome sequencing and assembly

Total RNA was extracted from a single adult moth of each species. Adult moths were collected at UV light and frozen live and without buffer in liquid nitrogen vapor. The *Papaipema speciosissima* specimen was collected on October 3, 2014 in Dukes County, MA; the *P*. “sp. 4” specimen was collected on October 3, 2013 in Union County, IL. RNA extraction was undertaken immediately upon removal from LN storage, with a brief interruption during which samples were maintained in a -80C freezer. Following dissection on a cryo-cooled work surface, we used half a thorax for each extraction. RNA was isolated using a standard Trizol protocol (https://tools.thermofisher.com/content/sfs/manuals/trizol_reagent.pdf), then processed on RNAEasy columns (Qiagen) to remove salts and other contaminants. We employed the Agilent RNA 6000 Nano assay on a BioAnalyzer to assess the overall quality of the extracted RNA and generate RIN values, and obtained accurate quantitation with the Qubit RNA BR (broad range) Assay. Total RNA was delivered to the New York Genome Center, where sequencing libraries were prepared using Illumina’s TruSeq stranded mRNA kit. Paired-end, 125bp cDNA libraries were sequenced on an Illumina HiSeq 2500, generating ~90 million reads per library.

Raw sequencing reads were visualized with FastQC (http://www.bioinformatics.babraham.ac.uk/projects/fastqc/) to determine the necessary quality filtering steps. We used Kraken [22] to eliminate any potential bacterial or viral contamination, and SortMe [23] to remove reads that were rRNA rather than mRNA to avoid misannotation of rRNAs as proteins [24]. Low quality reads and adapter sequences were eliminated with Trimmomatic [25].

**Fig 1:**
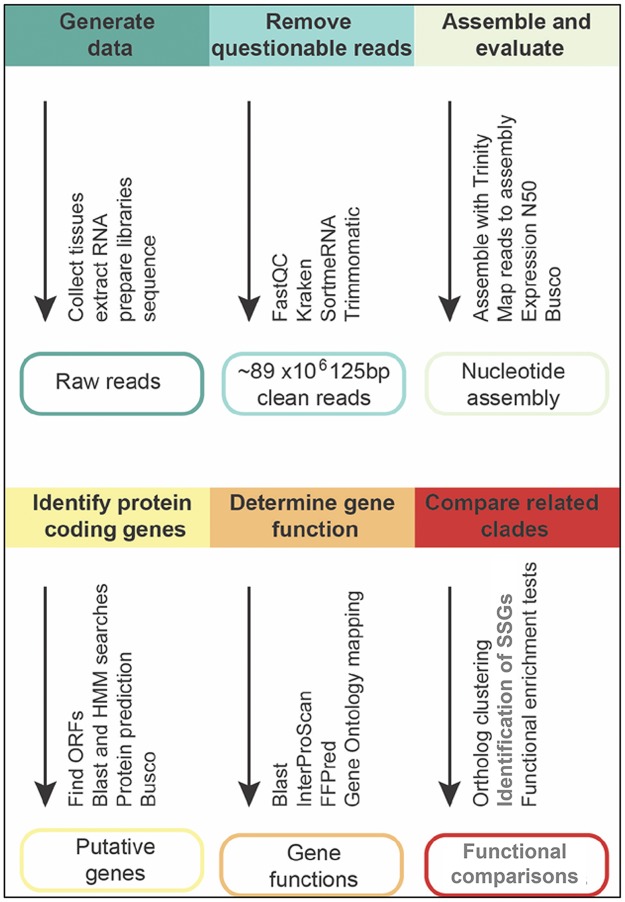
Summary of methods used.

We assembled the clean read set with Trinity [26] (v2.2.0), using the *SS_lib_type RF* option to generate strand-specific assemblies; see Fig 1 for a summary of the assembly pipeline. Summary statistics for each assembly were generated with *TrinityStats*.*pl*. The quality of the resulting assemblies was evaluated by mapping reads back to the assembly and by analyzing transcriptome completeness.

We evaluated the quality of the assembled transcriptomes by mapping the reads back to the assemblies and by examining the relationship between read coverage ("expression level") and contig length to determine the E90 N50 value. This measure is equivalent to the N50 for transcripts that represent 90% of the total normalized expression data, and we consider it a more reliable measure of assembly quality than the traditional N50 because it excludes contigs of reads with low expression levels. Such contigs are typically short because low read coverage impedes assembly [26]. We used BUSCO [27] to analyze transcriptome completeness according to conserved ortholog content.

### Identification and annotation of protein coding genes

We used TransDecoder’s 2-step prediction procedure for structural annotation (Haas & Papanicolaou et al., manuscript in prep. http://transdecoder.github.io). After identifying likely open reading frames (ORFs) with TransDecoder.LongOrfs, we used the amino acid sequence of the predicted ORFs as query sequences in (i) BLASTp searches against a custom database of all the Lepidoptera protein sequences from NCBI’s RefSeq database [28] (RefSeq sequences downloaded on February 23, 2016), and (ii) hmmscan searches against the Pfam-A database of protein family HMMs (hidden Markov models) [29]. ORF predictions and database search results were combined for the prediction of protein coding genes using Transdecoder.Predict, which allows for the retention of regions that contain ORFs longer than 900 bp (even if they lack database hits), and sequences that have BLAST hits or matches to Pfam HMM profiles (even if they lack complete ORFs). The resulting sets of putative protein coding genes for each species was used in all downstream analyses.

Annotation of the identified genes was performed with BLASTp and InterProScan 5 [30] searches. BLAST and InterProScan results were imported to Blast2GO [31] and mapped to GO terms with an annotation score cutoff of 35. Annotations were filtered to retain only those corresponding to annotated arthropod genes (taxa: 6656, Arthropoda). Sequences lacking GO terms at this stage were subjected to additional analysis using FFPred 2.0, a “homology-independent” tool for GO term prediction [32]. The reliability of FFPred predictions is measured by the Matthews Correlation Coefficient (MCC) in the underlying support vector regression model, with values close to 1 considered highly reliable [32]. The FFPred results were filtered to include only GO predictions whose posterior probabilities were ≥ 0.9 and whose support vector regression models were classified as highly reliable.

To evaluate the representation of full-length protein-coding genes, we used BLASTp to search our custom database, using the translated peptide sequences as a query.

### Ortholog identification and SSGs

To determine orthology between protein coding genes from *P*. sp. 4 and *P*. *speciosissima*, we used OrthoPipe, a stand-alone pipeline version of OrthoDB 2.3.1 [33]. Default parameters were modified to increase stringency by setting MIN_OVERLAP = 50 and MAX_EVALUE = 1.0e-^5^.

Because SSGs are at greater risk of being misassembled than genes for which orthologs are known from multiple species, we conducted additional quality checks on SSGs, beginning with TransRate [34], a tool for evaluating *de novo* transcriptome assemblies, to compare the contig scores for orthologous (shared) versus species-specific genes. The TransRate contig score, which is not weighted by expression level, reflects how well a given contig is supported by read evidence [34], and those sequences whose underlying contig score was below the TransRate cutoff (the minimum acceptable contig score that maximizes the overall assembly score) were excluded from further analysis. Because an absence of orthologs detected between *P*. sp. 4 and *P*. *speciosissima* need not imply that a gene is truly species-specific (merely that no orthologs were identified in our dataset), we conducted an additional set of BLASTp searches against NCBI’s nr database [35] to determine whether putative SSGs had orthologs in any other species.

### Transposable element evaluation

Endogenous transposable elements (TEs or “jumping genes”) are DNA sequences that can shift positions on the genome [36]. Genes with InterPro signatures related to TEs were assessed for the presence of IPR000477 (Reverse transcriptase domain), which is the only domain shared by all retrotransposons [37]. Functional retrotrotransposons are defined by the co-occurrence of multiple domains, specifically a GAG-pre-integrase domain, a peptidase domain, a reverse transcriptase domain, a ribonuclease H-like domain, and an integrase domain. To examine the characteristics of TE-related signatures in *Papaipema* genes, we selected those with InterPro signatures corresponding to at least two of these domains for further analysis. The resulting set of genes was analyzed first with NCBI’s CD-Search, which uses position-specific scoring matrices to compare a query sequence to the Conserved Domain Database (a collection of multiple sequence alignment models for conserved, well-annotated protein domains) [38]. We then compared the domain architecture of these genes to known retrotransposon families [39] to determine likely identities and assign them to TE families.

*Ty1/Copia* is one of the oldest and most representative families of LTR retroelements in eukaryotes and probably predates the origin of plants [37]. To examine the relationship between Ty1/Copia elements from *Papaipema* and those of other insects, we first conducted a PSI-BLAST (position-specific iterated BLAST [40]) search against all RefSeq Endopterygota proteins. We collected the highest-scoring hit sequences from lepidopteran species and holometabolous insects outside Lepidoptera, and used MUSCLE [41] to generate a multiple sequence alignment that included *Papaipema* Ty1/Copia genes. We then used FastTree 2.1 [42] to infer a maximum-likelihood phylogram, visualized with FigTree v4.1.3 [43].

## Results

### Transcriptome assemblies

Assembly metrics were similar for the two *Papaipema* species (Table 1). We detected little exogenous content (as determined by Kraken assessment) and read qualities were uniformly high. The read quality and contaminant filtering steps removed less than 1% of the reads; since we have observed significantly higher proportions of “bad” reads in other data sets, it is not our intention to discourage filtering.

**Table 1:** Summary of transcriptome sequencing and assembly results.

Source	Metric	*P*. *sp*.*4*	*P*. *speciosissima*
**Raw data**	Number of 125bp reads	90.2 M	90.5 M
	Total bp of sequence data	11.3 Gbp	11.3 Gbp
**Clean up**	Percent of reads removed by Kraken	0.2	0.3
	Percent of reads removed by SortMeRNA	0.4	0.4
	Percent of reads removed by Trimmomatic	0.000005	0
	Percent of reads remaining after clean up	99.4	99.3
**Assembly**	Total assembly length	85.5 kbp	97.2 kbp
	Contig N50	1.7 kbp	2.2 kbp
	Expression 90 N50	2.4 kbp	2.4 kbp
	Number of Trinity "genes"	77 K	45 K
	Number of Trinity "isoforms"	95 K	69 K
	Percent GC	39.1	39.2
	Percent of reads aligned to assembly	87.2	88.1
	Percent of aligned reads in proper pairs	72.7	73.3
	Percent of arthropod core genes missing (BUSCO)	13.3	13.1
**Putative protein coding genes**	Number of putative genes	26.6 K	31.9 K
	Percent GC	46.3	46
	Percent of arthropod core genes missing (BUSCO)	13	13.4

Almost 90% of the raw reads were represented in each assembly, and more than 70% of the reads mapped to each assembly in proper pairs. For both species, the E90 N50 (the N50 for transcripts that represent 90% of the total normalized expression data) was greater than the traditional N50: 2,365 bp versus 1,737 bp for *P*. sp. 4 and 2,411 versus 2,159 for *P*. *speciosissima* (S1 Fig).

The initial Trinity assemblies contained more putative genes (77,000 for *P*. sp. 4, 45,000 for *P*. *speciosissima*) than are found in a typical lepidopteran genome. Such inflation is characteristic of *de novo* transcriptome assemblies and is thought to result from the assembly of incomplete reads [44]. After TransDecoder analysis and the removal of redundant sequences (those with 100% sequence similarity), a final set of 23,278 putative protein-coding genes in *P*. sp. 4 and 23,964 in *P*. *speciosissima* was retained.

BUSCO [27] analysis of transcriptome completeness showed that ~87% of the expected Arthropod single-copy orthologs were present for both *P*. sp. 4 and *P*. *speciosissima*. BLASTp searches to evaluate the representation of full-length protein-coding genes showed that almost 40% of the query sequences covered 100% of their top BLAST hits (S2 Fig), while the rest were distributed evenly across lower coverage bins ranging from 10–90%. E-values were generally significant at a level beyond the default 1e^-5^ cutoff (S3 Fig), with the majority of hits having E-values ≤ 1e-180.

### Annotation

Following the BLASTp searches, there were 4,533 sequences from *P*. sp. 4, and 2,436 from *P*. *speciosissima* that had no BLAST hit. InterPro annotations were assigned to ~77% of the genes in each species (Table 2). The most commonly identified InterPro signatures, which were similar in the two species, are summarized in S1 Table.

**Table 2:** Annotation results.

Taxa	N	With Blast hit	With InterPro signature	With GO annotation	Not Shared^1^	Species specific^2^
***P*. *sp*.*4***	23,278	18,745	18,227	18,739	2,677	1,285
***P*. *speciosissima***	23,964	21,528	18,494	19,564	2,918	1,144

^1^Has Blast hit to Lepidoptera, but has no ortholog in the other Papaipema species

^2^No Blast hit to Lepidoptera, and no ortholog in the other Papaipema species

GO term mapping based on the results of BLAST, InterPro, and FFPred searches resulted in GO annotation for ~80% of the genes from each species. See S4 Fig for summaries of the most common GO terms in each species, and S5 Fig for the distribution of GO annotation scores As expected for non-model organisms, most GO annotations were “Inferred from Electronic Annotation” (IEA), meaning they result from computational annotation and have not yet been reviewed by a GO curator [45]. See S2 Table for a summary of GO evidence codes.

### Ortholog identification and SSGs

**Fig 2:**
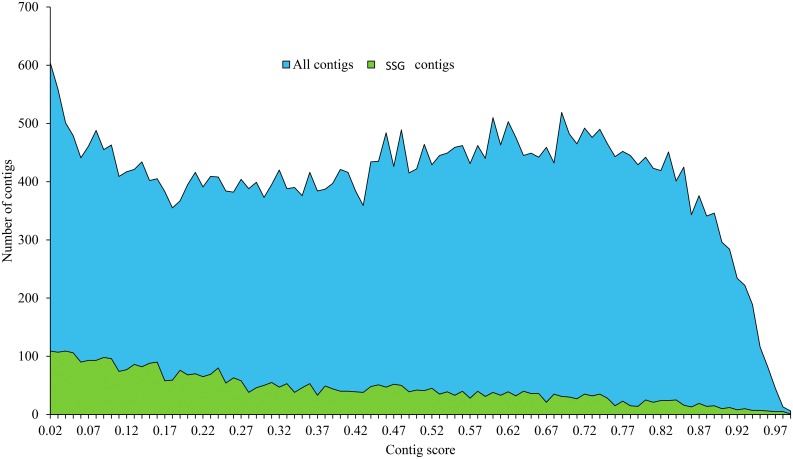
TransRate contig support scores for species-specific *Papaipema* genes versus those with identifiable orthologs in both species.

Genes were classified into 10,207 clusters, each with 2 to 110 genes (average cluster size 3.3). A narrow majority of clusters (51%) were single-copy orthologs containing one gene from each species. Some 8,000 genes, representing about 17% of each transcriptome, failed to cluster as orthologs. These were provisionally designated species-specific genes (SSGs), having been found only in one of the two *Papaipema* species. The support scores for contigs containing putative SSGs were similar to those containing identifiable orthologs (Fig 2). After BLASTP searches against the nr database, 30% of these putative SSGs had no hit, and these were treated as legitimate SSGs.

### Assessment of functional divergence

**Fig 3:**
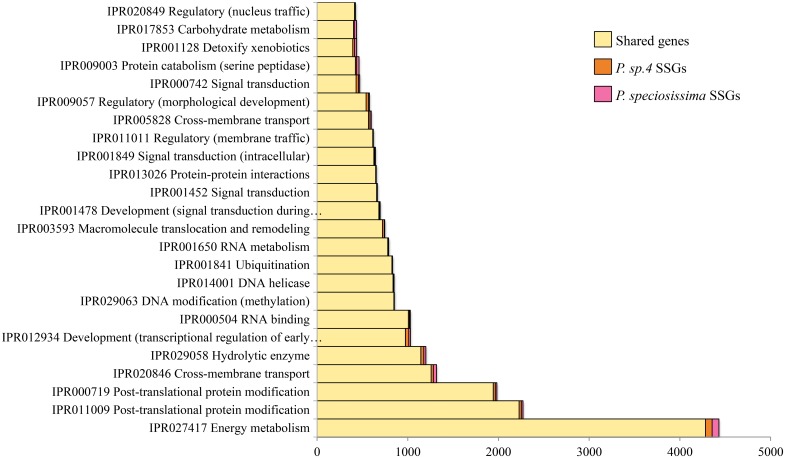
Most frequent InterPro signatures in orthologous *Papaipema* genes. Summary functions shown—see S3 Table for full information.

In comparing the InterPro signatures of orthologous versus species-specific genes, we noted numerous InterPro signatures more abundant among orthologs than among SSGs (Fig 3). The majority of these were related to energy metabolism (P-loop NTPases), protein production and integrity (peptidases and protein kinases), and cross-membrane transport of small solutes (the major facilitator superfamily of sugar transporters). Other signatures common in shared orthologs included detoxification (Cytochrome P450s) and DNA modification (helicases and methyltransferases).

**Fig 4:**
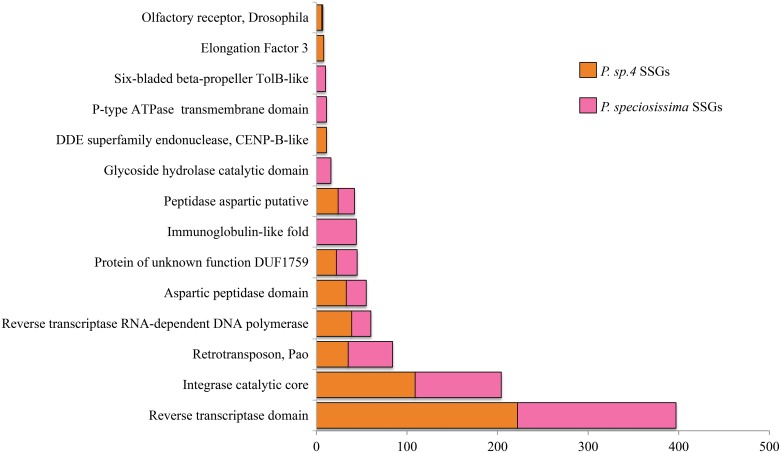
InterPro signatures found only in species-specific genes.

More than fifty InterPro signatures appeared exclusively in species-specific genes (Fig 4), and others were far more common in SSGs than in shared orthologs (S3 Table). Some of the SSG signatures were present in genes from both *Papaipema* species, while others occurred in only one. The most common signatures among SSGs involved DNA-mediated transposition, with 45% containing a transposable element domain. Other notable signatures included chemosensory receptors from *Drosophila* and Lepidoptera and glycoside hydrolases involved in carbohydrate metabolism.

### Transposable element evaluation

**Fig 5:**
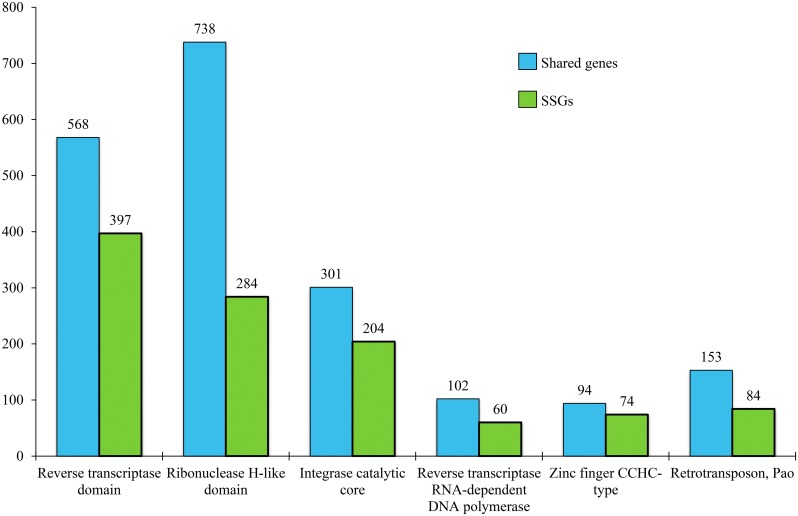
Retrotransposon-related InterPro signatures in orthologs and species-specific genes. Number of genes shown at top of each bar.

InterPro signature analysis showed that TE-related domains and families were abundant among SSGs. Although the total number of genes with TE-related signatures was higher among shared orthologs (Fig 5), the relative abundance of TE-related signatures was much greater in SSGs. In the most extreme case, 16% of all InterPro annotated SSGs had signatures for IPR000477 (Reverse transcriptase domain) versus 1.4% of shared genes. Using NCBI’s CD-Search to compare the domain architecture of SSGs to known retrotransposon families [39], we found 68 SSGs that grouped into three TE families: BEL/Pao, Ty1/Copia, and Ty3/Gypsy, all of which are LTR (long terminal repeat) retrotransposons (S6 Fig). While all three families occur in both *Papaipema* species, the frequency of each family differed between them (Table 3).

**Table 3:** Retrotransposon families identified in species-specific genes.

	Ty1/Copia	Ty3/Gypsy	BEL/Pao
***P*. *sp*.*4***	2	23	6
***P*. *speciosissima***	6	17	14

**Fig 6:**
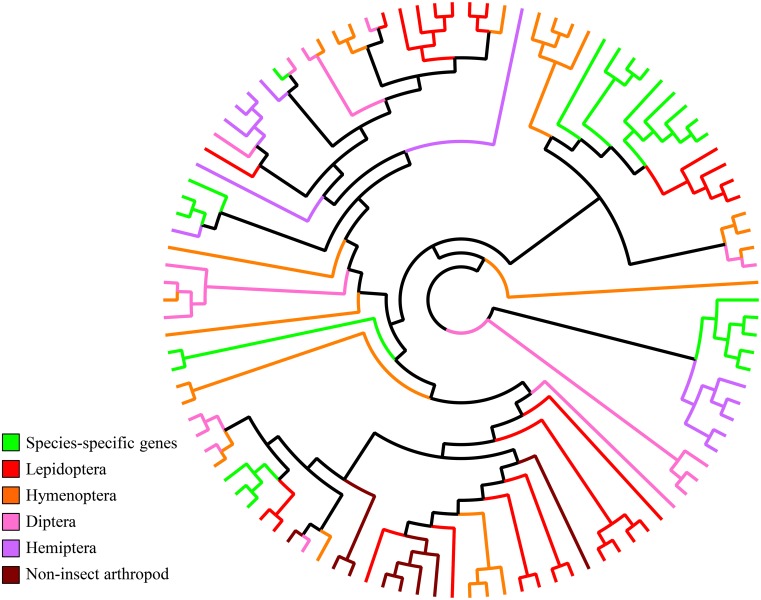
Phylogram of retrotransposons from Hymenoptera, Diptera, and Lepidoptera, including putative Ty1/Copia genes identified in this study.

The pairwise similarity between Ty1/Copia elements from *Papaipema* SSGs and those from other insects ranged from 30–40%. In the maximum-likelihood phylogram of Ty1/Copia relationships (Fig 6), most of the genes from other lepidopteran species grouped into taxonomically diverse clusters (containing members of *Spodoptera*, *Bombyx*, *Amyelois*, and *Plutella*), while the *Papaipema* genes displayed more taxon-specific differentiation and shared a node only with *Sesamia*, a closely related stem-borer in the sister subtribe of the Apameini.

## Discussion

Despite their disparate life histories and specialization on unrelated hosts, *de novo* comparison of two *Papaipema* transcriptomes revealed substantial similarity in gene content, no doubt reflecting the recency of this North American radiation. More than 19,000 genes were identifiable as shared orthologs, and were associated primarily with basic protein and energy metabolism. Other shared genes, such as detoxification enzymes in the Cytochrome P450 group, bear directly on the chemical ecology of plants and insect herbivores. However, almost 20% of each species’ genes lacked orthologs in the other species. While many of these unshared genes had orthologous counterparts in other insects, almost a third of them (more than 1,000 genes in each species) appear to be species-specific genes without orthologs among previously sequenced genes.

It is now generally recognized that as many as a third of the genes in a given genome lack recognizable homologs in other species [46]. Putative species-specific genes share several characteristics that raise doubts about their status, namely: shorter ORFs, fewer exons, lower expression levels and higher tissue specificity levels than genes with identified orthologs [47]. The most convincing evidence that species-specific genes are “real” comes from functional validation studies demonstrating that many of them are essential to normal development in model organisms [48, 49].

Two of the InterPro signatures found only in species-specific *Papaipema* genes are directly relevant to the evolution of diet breadth: olfactory receptors and aspartic peptidases. Olfactory receptors are involved in the detection of volatile odorant cues from plants, and can determine the range of plant species that herbivores accept as hosts [50]. Aspartic peptidases, which break down the peptide bonds in dietary proteins, are essential to the survival of plant feeding insects [51]. Lineage-specific evolution of aspartic peptidases appears to be common in insects with different feeding habits [52]. Within *Papaipema*, the presence of species-specific genes in these two categories suggests they may contribute to sensory and digestive adaptations to endophagous feeding.

Several processes contributing to the emergence of species-specific genes have been identified [53]; at this writing, it appears that gene duplication (including duplication via retrotransposition) and TE “domestication” are major creative forces in the emergence of species-specific genes [19–21]. TEs are an abundant source of biochemically active elements such as transcription factor-binding sites, and TE insertions generate genomic rearrangements that can foster the emergence of species-specific genes [19, 54–56].

The high frequency of TE-related InterPro signatures in the species-specific genes identified in this study is noteworthy. In *D*. *melanogaster*, TE insertion rates typically range from 0.005 to 0.00005 insertions per-copy per-generation [57], but their potential impact on fitness is greater than these low numbers suggest. Transposition events can cause recessive lethal mutations and reduce viability [58], and purifying selection is inefficient at eliminating them because transposons can propagate within a genome to exceed the Mendelian segregation ratios imposed by meiosis [59]. Though the mechanisms that prevent TEs from dominating the genome are not yet known, it is clear that host species, including insects, have evolved a variety of TE-silencing strategies, including DNA methylation, chromatin remodeling, and microRNAs [60–63].

There is growing evidence, however, that the net impact of transposition events may be neutral or even positive. Transposon insertions increase genetic diversity by translocating genomic sequences and reshuffling exons, potentially creating novel gene products in a single step [64], and can alter gene expression patterns by inserting into regulatory regions [65]. While the results of most of these genomic “experiments” are likely to be deleterious, several well-documented examples demonstrate that TEs are an important source of adaptive innovation. In *D*. *melanogaster*, for example, upregulation of the Cytochrome P450 gene cyp6g1, caused by an upstream retrotransposon insertion, confers resistance to several insecticides [66]. Other examples of host co-option of TEs for regulation of host genes are widespread [62], and suggest that TE insertions can contribute to rapid transcriptional rewiring [67]. Many TEs become more active in stressful conditions [68, 69], and their ability to create new genetic variability when conditions are challenging may serve their hosts as an inducible stress response mechanism [70, 71].

In the present study, the over-representation of TE-related signatures among species-specific genes suggests that the dynamics underlying the evolution of “new” genes may have played a role in the rapid diversification of host plant range and feeding habits in endophagous herbivores. Currently we are unable to say whether the TE-like genes we identified are fully functional transposable elements or if they represent domestication events. One set of apparently functional TEs (from the Ty1/Copia family of LTR retrotransposons) displayed elevated rates of differentiation in *Papaipema*, but this may simply reflect the limited taxonomic diversity of available transcriptome data. Future comparative studies that include a mixture of internal and external feeders from a range of taxonomic levels within the Lepidoptera will show whether TEs and TE-related sequences are equally common in all species-specific genes or if their abundance is particular to *Papaipema* and other endophages. Analysis of a broader data set would also allow for a comparison of the rates of orphan gene emergence in different taxa, and whether duplication, transposition, or other mechanisms are prevalent. It is our hope that continued comparative work towards understanding the evolutionary origins of endophagy will help to illuminate the rapid diversification of lepidopteran feeding habits that simultaneously exemplifies the ability of insects to respond to natural selection and explains the origins of agricultural pests that threaten worldwide food security.

## Supporting information

S1 Fig Contig N50 by expression level. (TIFF)

S2 Fig Top BLAST hit coverage for assembled contigs and predicted protein coding genes. (TIFF)

S3 Fig BLAST hit E-value distribution for predicted protein coding genes. (TIFF)

S4 Fig Most frequent GO terms for predicted protein coding genes. A. Biological Process GOs; B. Molecular Function GOs; C. Cellular Component GOs.
(TIFF)

S5 Fig Annotation score distribution for predicted protein coding genes. (TIFF)

S6 Fig Representative domain architectures for retrotransposon families identified in species-specific genes. A) Ty3/Gypsy retrotransposon from *P*. *speciosissima*; B) Ty1/Copia retrotransposon from *P*. *sp*.*4*; C) Retrotransposon Pao sequence from *P*. *speciosissima*.
(TIFF)

S1 File Supplemental methods. Detailed bioinformatic methods with examples of all commands used.
(PDF)

S1 Table Most frequent InterPro signatures. (PDF)

S2 Table GO annotation evidence codes. (PDF)

S3 Table InterPro signatures in orthologs and species-specific genes. (PDF)
