# Supplementary figures and images for: De Novo characterization of transcriptomes from two North American Papaipema stem-borers (Lepidoptera: Noctuidae)
Source: PLoS One. 2018 Jan 24;13(1):e0191061. doi: 10.1371/journal.pone.0191061 (PMC5783364; doi:10.1371/journal.pone.0191061)

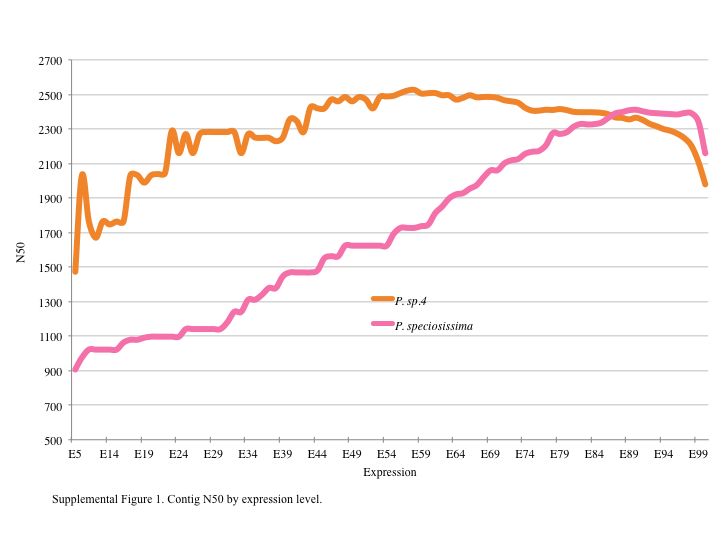

Supplement: S1 Fig — (TIFF) [file pone.0191061.s001.tiff]

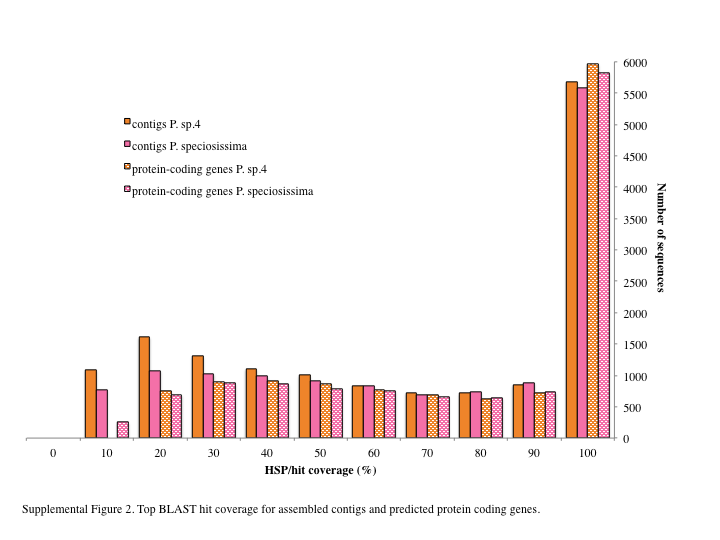

Supplement: S2 Fig — (TIFF) [file pone.0191061.s002.tiff]

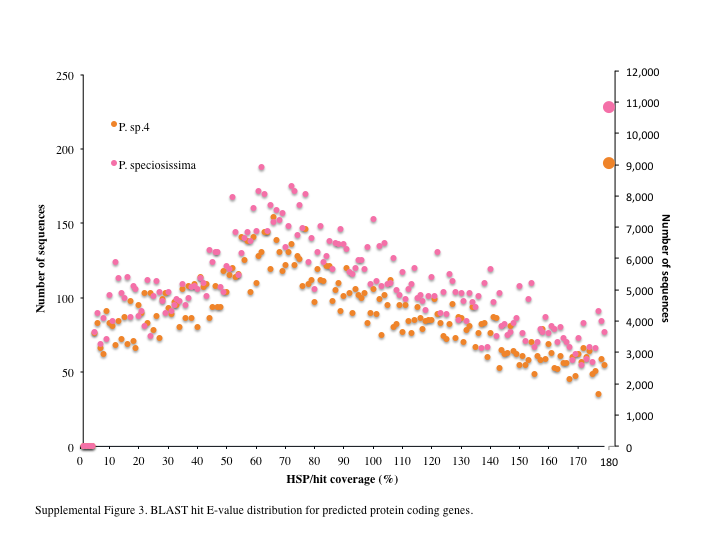

Supplement: S3 Fig — (TIFF) [file pone.0191061.s003.tiff]

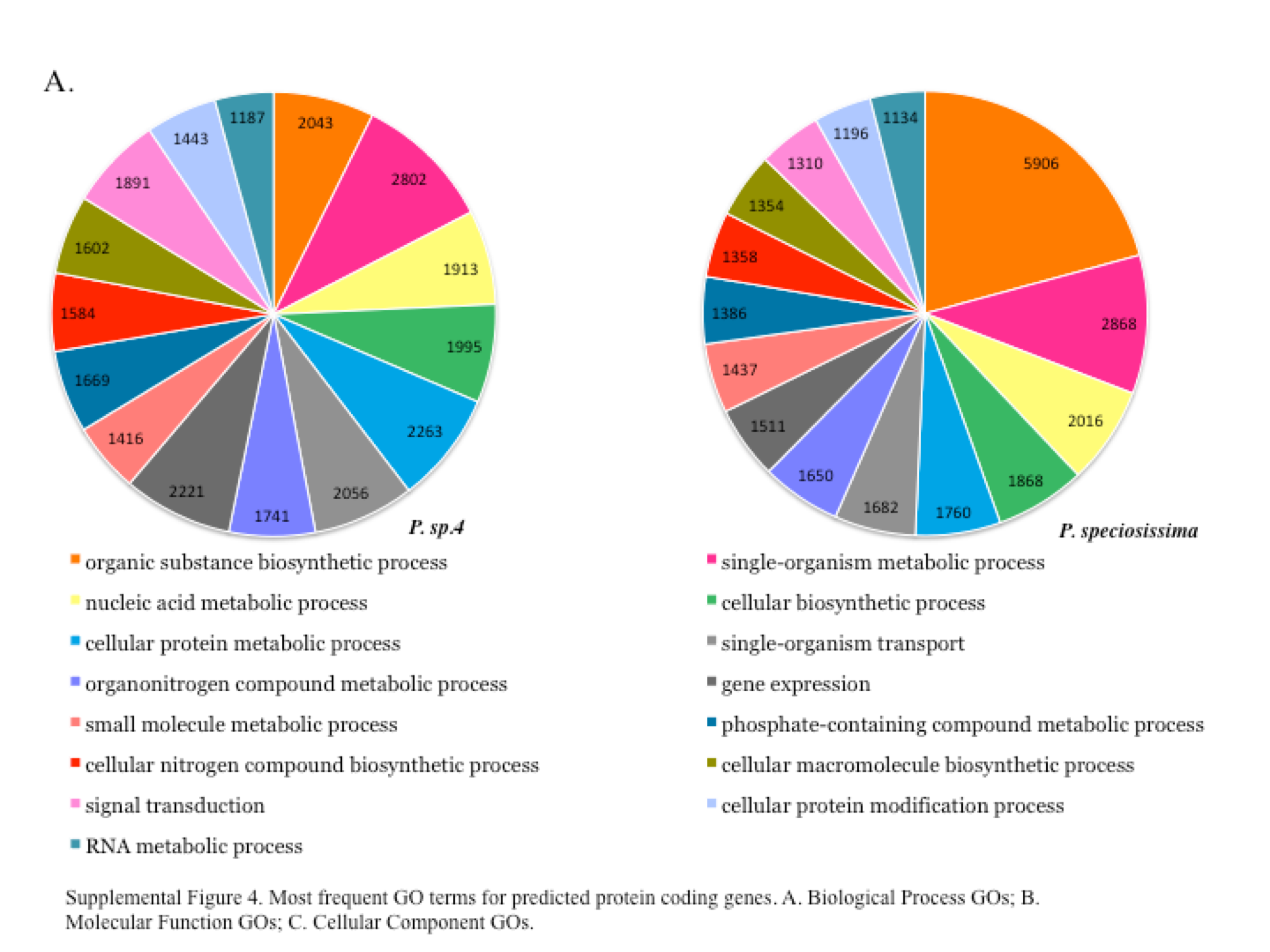

Supplement: S4 Fig — A. Biological Process GOs; B. Molecular Function GOs; C. Cellular Component GOs. (TIFF) [file pone.0191061.s004.tiff]

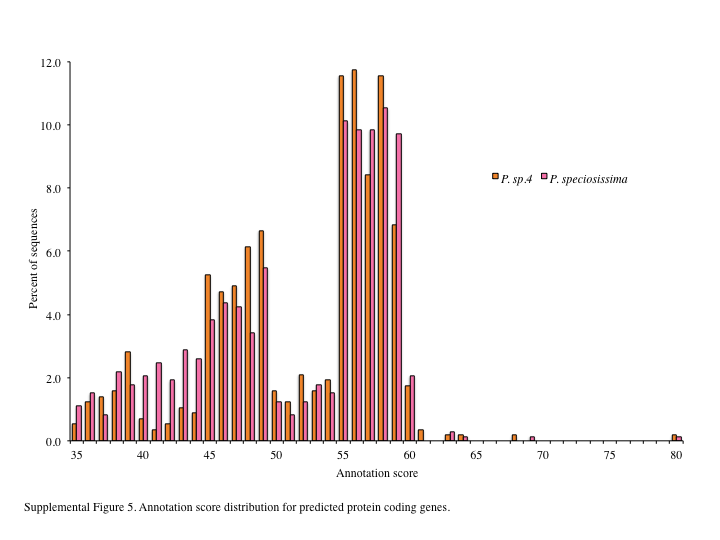

Supplement: S5 Fig — (TIFF) [file pone.0191061.s005.tiff]

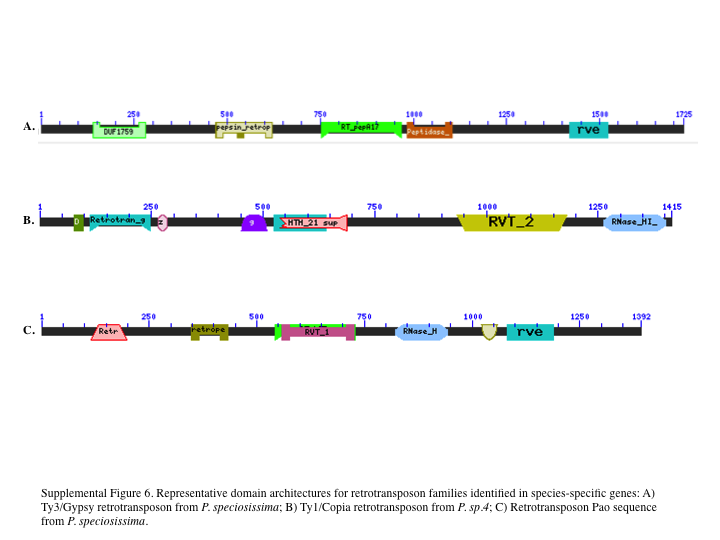

Supplement: S6 Fig — A) Ty3/Gypsy retrotransposon from P. speciosissima; B) Ty1/Copia retrotransposon from P. sp.4; C) Retrotransposon Pao sequence from P. speciosissima. (TIFF) [file pone.0191061.s006.tiff]
