# Supplementary material for: De Novo characterization of transcriptomes from two North American Papaipema stem-borers (Lepidoptera: Noctuidae)
Source: PLoS One. 2018 Jan 24;13(1):e0191061. doi: 10.1371/journal.pone.0191061 (PMC5783364; doi:10.1371/journal.pone.0191061)
Supplement: S1 File — Detailed bioinformatic methods with examples of all commands used. (PDF) [file pone.0191061.s007.pdf]

Bioinformatics methods

COMPUTATIONAL WORKFLOW

RAW READ PROCESSING:
1. FastQC on raw reads
2. Kraken on raw reads to ID and remove contaminants
3. SortmeRNA to filter out rRNA
4. Trimmomatic to filter by quality & remove adapters
5. FastQC on "clean" reads

ASSEMBLY AND ASSESMENT:
6. Use Trinity to assemble filtered read set
7. QC with TrinStats, Busco
8. Map reads back to assembly, get stats (bowtie_PE_separate_then_join.pl)
9. Get Expression N50 values (align_and_estimate_abundance.pl, abundance_estimates_to_matrix.pl, contig_ExN50_statistic.pl)
10. TransRate to get quality scores for contigs and assemblies
11. Do a BlastX search against LepRefSeq DB
12. Assess completeness of transcripts (analyze_blastPlus_topHit_coverage.pl)

IDENTIFICATION OF PROTEIN CODING GENES:
13. Transdecoder longest_orfs to extract ORFS
14. QC again with TrinStats, Busco
15. Assess completeness of transcripts (analyze_blastPlus_topHit_coverage.pl)
16. Do a BlastP search against LepRefSeq DB
17. Assess completeness of transcripts (analyze_blastPlus_topHit_coverage.pl)
18. TransDecoder_Predict to get peptides
   a. This includes running a BlastX search against LepRefSeq DB, and doing hmmscan using Pfam DB. Both use the transdecoder_longest_orfs as query. Output is the peptides.
19. QC again with TrinStats, Busco

FUNCTIONAL ANNOTATION:
20. Do a BlastP search against LepRefSeq DB.
21. Assess completeness of transcripts (analyze_blastPlus_topHit_coverage.pl)
22. For sequences that had no hit against the LepRefSeq DB, do a search against all of RefSeq.
23. For sequences that still have no hit, do a search against all NON-RefSeq lepidoptera.
24. For sequences that still have no hit, do FFPred.
25. Do Interproscan.

ORTHOLOG CLUSTERING:
26. Identify ortholog clusters with OrthoDB standalone (OrthoPipe)
27. Identify putatively species-specific (not clustered) sequences

COMPARISONS:
28. GO term mapping with Blast2Go
29. Functional enrichment tests in Blast2GO
30. Functional annotation and comparison of species-specific genes

EXAMPLES OF COMMANDS:
1. FastQC on raw reads
```
$FastQC/fastqc <reads.fastq> -o /path/to/output/dir/ -t <num_threads>
```

2. Kraken on raw reads to ID and remove contaminants
Run separately for R1 and R2:
$kraken-0.10.5-beta/scripts/kraken --db /path/to/Kraken_DB --preload --fastq-input --threads <N> --unclassified-out /path/to/non_kraken_reads.fastq --classified-out /path/to/kraken_reads.fastq /path/to/raw_reads.fastq<or raw_reads.fastq.gz>

From unclassified-out, extract pairs with both members of pair (R1 and R2) non-kraken. ##Output of this script will be <pairs_R1.fastq> <pairs_R2.fastq>
$python /path/to/fastqCombinePairedEnd.py nonKraken_R1.fastq nonKraken_R2.fastq

3. SortmeRNA to filter out rRNA
Index rRNA dbs (you have to have these installed already). They only need to be indexed once.

merge paired non-kraken (nK) read files (must be fasta or fastq, not .gz):
$bash /path/to/merge-paired-reads.sh /path/to/nK_pairs_R1.fastq /path/to/pairs_R2.fastq /path/to/output/merged_nK_reads.fastq&

RUN sortmerna:
$sortmerna-2.0-linux-64/sortmerna --ref /path/to/databases/and/indexes/sortmerna-2.0-linux-64/rRNA_databases/silva-bac-16s-id90.fasta,/mnt/data27/oppenheim/src/sortmerna-2.0-linux-64/index/silva-bac-16s-db: <you can have multiple DB+index pairs, separated by <:> --reads merged_nK_reads.fastq --fastx --aligned /path/to/output/that/IS/rRNA/merged_reads_rRNA.fastq --other /path/to/output/that/isNOT/rRNA/merged_nK_reads_NONrRNA.fastq --log -v -a <num_threads> --paired_in -e 1e-20

##un-merge paired read output files
$bash /path/to/unmerge-paired-reads.sh /path/to/merged_nK_reads_NONrRNA.fastq /path/to/R1/output/nK_nR_R1.fastq /path/to/R2/output/nK_nR_R2.fastq

##re-pair the reads to retain only sets with both R1 and R2 classified as non-kraken, non-rRNA
$python /path/to/fastqCombinePairedEnd.py /path/to/nK_nR_R1.fastq /path/to/nK_nR_R2.fastq

4. Trimmomatic
$java -jar /path/to/Trimmomatic-0.32/trimmomatic-0.32.jar PE -phred33 input_forward.fq.gz input_reverse.fq.gz output_forward_paired.fq.gz output_forward_unpaired.fq.gz output_reverse_paired.fq.gz output_reverse_unpaired.fq.gz ILLUMINACLIP:TruSeq3-PE.fa:2:30:10 LEADING:3 TRAILING:3 SLIDINGWINDOW:4:15 MINLEN:36

5. FastQC on "clean" reads
$FastQC/fastqc /path/to/nK_nR_R1.fastq -o /path/to/output/dir/ -t <num_threads>

6. Use Trinity to assemble fltered read set
$export _JAVA_OPTIONS="-Xms640M -Xmx640M"
$export PATH=${PATH}:/path/to/trinityrnaseq
$export PATH=${PATH}:/path/to/trinityrnaseq:/path/to/bowtie-0.12.7
$export PATH=${PATH}:/path/to/samtools-0.1.19

$/path/to/trinityrnaseq/Trinity --JM 10G --trimmomatic <to run trimmomatic before assembly, if not done earlier> --seqType fq --SS_lib_type RF <only if your data are strand specific> --left /path/to/nr_nk_R1.fastq --right /path/to/nr_nk_R2.fastq --full_cleanup --bflyGCThreads 2 --CPU <num_threads> --output /path/to/output/Assembly_nR_nK > /optional/path/to/stderr/Assembly_nR_nK.stderr

7. QC with TrinStats, Busco
Trinity stats:
$perl /path/to/trinityrnaseq-2.0.6/util/TrinityStats.pl /path/to/assembly/nR_nK.Trinity.fasta

Busco:
$cd /path/to/output/directory/
$export PATH=$PATH:/path/to/hmmer-3.1/bin/
$export PATH=$PATH:/path/to/EMBOSS/bin/
$/path/to/BUSCO_v1.1b1/BUSCO_v1.1b1.py -o <output_name> -in /path/to/assembly/nR_nK.Trinity.fasta -l /path/to/Busco/lineage/BUSCO_v1.1b1/arthropoda -m genome <specify mode: genome, transcriptome, gene set (OGS)> -c <num_threads> -f <to overwite previous results with same name>

8. Map reads back to assembly, get stats (bowtie_PE_separate_then_join.pl)
$/path/to/trinityrnaseq/util/bowtie_PE_separate_then_join.pl --seqType fq --left /path/to/nK_nR_R1.fastq --right /path/to/nK_nR_R2.fastq --target /path/to/assembly/nR_nK.Trinity.fasta --aligner bowtie --SS_lib_type RF <if SS data> --output /path/to/output/nR_nK.Trinity.fasta.ReadStats -- -p <num_threads> --all --best --strata -m 300

##An output directory is created and should include the files:
bowtie_out.nameSorted.bam : alignments sorted by read name
bowtie_out.coordSorted.bam : alignments sorted by coordinate.

##To get alignment statistics, run the following on the name-sorted bam file:
$/path/to/trinityrnaseq/util/SAM_nameSorted_to_uniq_count_stats.pl /path/to/nR_nK.Trinity.fasta.ReadStats/nR_nK.Trinity.fasta.ReadStats.nameSorted.bam > /path/to/redirect/and/name/output/nR_nK.Trinity.fasta_Read_Stats

9. Get Expression N50 values (align_and_estimate_abundance.pl, abundance_estimates_to_matrix.pl, contig_ExN50_statistic.pl)
##Prepare reference
$/path/to/trinityrnaseq/util/align_and_estimate_abundance.pl --transcripts /path/to/assembly/nR_nK.Trinity.fasta --est_method RSEM --aln_method bowtie --trinity_mode --prep_reference

##Align reads to reference
$/path/to/trinityrnaseq/util/align_and_estimate_abundance.pl --transcripts /path/to/assembly/nR_nK.Trinity.fasta --seqType fq --SS_lib_type RF --thread_count 2 --left /path/to/nK_nR_R1.fastq --right /path/to/nK_nR_R2.fastq --est_method RSEM --aln_method bowtie --trinity_mode --prep_reference --output_prefix /path/to/and/prefix/of/output/reads_to_assem

##Construct a matrix of counts and a matrix of normalized expression values

```
$/path/to/trinityrnaseq/util/abundance_estimates_to_matrix.pl --est_method RSEM /path/to/reads_to_assem.isoforms.results --out_prefix /path/to/output/reads_to_assem_expression

##If you only have one sample, you can't make a "matrix." Instead, extract needed values from the isoforms.results file:
$cat /path/to/reads_to_assem.isoforms.results | perl -lane 'print "$F[0]\t$F[5]";' > /path/to/output/reads_to_assem.isoforms.results.mini_matrix

##Get Contig Expression N50 Statistic:
$/path/to/trinityrnaseq/util/misc/contig_ExN50_statistic.pl /path/to/reads_to_assem.isoforms.results.mini_matrix /path/to/assembly/nR_nK.Trinity.fasta > /path/to/output/ExN50_results.txt
```

10. TransRate

```
$/path/to/transrate --assembly /path/to/assembly.fasta --left /path/to/reads.R1.fastq --right /path/to/reads.R2.fastq --output /path/to/output_directory
```

11. Do a BlastX search against LepRefSeq DB

```
$blastx -query /path/to/assembly/nR_nK.Trinity.fasta -db /mnt/data27/oppenheim/BlastDBs/22316_LepRefSeq.DB -max_target_seqs 1 -outfmt 11 -evalue 1e-5 -num_threads 4 -out /path/to/output/nR_nK.Trinity_blastx_to_22316_LepRefSeq.11
```

12. Assess completeness of transcripts (analyze_blastPlus_topHit_coverage.pl)

Convert Blast result to outfmt 6:

```
$blast_formatter -archive blast_output.11 -outfmt 6 -out blast_output.6
```

If blast result has more than 1 hit per query, first extract only the top hit:

```
$sort -k1,1 -k12,12gr -k11,11g -k3,3gr blast_output.6 | sort -u -k1,1 --merge > bestHits_blast_output.6
qseqid sseqid pident length mismatch gapopen qstart qend sstart send evalue bitscore
```

Assess (output will be bestHits_blast_output.6.txt):

```
$/path/to/trinityrnaseq/util/analyze_blastPlus_topHit_coverage.pl bestHits_blast_output.6 /path/to/assembly/nR_nK.Trinity.fasta /path/to/fasta_file/of/blast_db/22316_LepRefSeq.fasta
```

Group the multiple HSPs per transcript/database_match pairing like so:

```
$/path/to/trinityrnaseq/util/misc/blast_outfmt6_group_segments.pl bestHits_blast_output.6.txt /path/to/assembly/nR_nK.Trinity.fasta /path/to/fasta_file/of/blast_db/22316_LepRefSeq.fasta > /path/to/output/bestHits_blast_output.6.txt.grouped
```

Get histogram for grouped coverage:

```
$/path/to/trinityrnaseq/util/misc/blast_outfmt6_group_segments.tophit_coverage.pl /path/to/bestHits_blast_output.6.txt.grouped > /path/to/output/bestHits_blast_output.6.txt.grouped_percent_coverage_by_length
```

13. Transdecoder.LongOrfs to extract ORFS

```
$cd /path/to/directory/where/assembly/is/
$/path/to/TransDecoder-2.0.1/TransDecoder.LongOrfs -t <assembly.fasta> -S <only if data are strand specific>
```

14. QC again with TrinStats, Busco
See step 7

15. Assess completeness of transcripts (analyze_blastPlus_topHit_coverage.pl)
See step 12

16. Do a BlastP search against LepRefSeq DB
```
$blastp -query /path/to/longest_orfs/nR_nK.Trinity.fasta_longest_orfs.pep -db
/mnt/data27/oppenheim/BlastDBs/22316_LepRefSeq.DB -max_target_seqs 1 -outfmt 11
-evalue 1e-5 -num_threads 4 -out
/path/to/output/nR_nK.Trinity.fasta_longest_orfs.pep_blastp_to_22316_LepRefSeq.1
1
```

17. Assess completeness of transcripts (analyze_blastPlus_topHit_coverage.pl)
See step 12

18. TransDecoder_Predict to get peptides (This includes running a BlastP search against LepRefSeq DB (step 16), and doing hmmscan against Pfam DB. Both use the transdecoder_longest_orfs as query. Output is the peptides.)

Blastp output was produced in step 16, must be converted to outfmt6:
```
$blast_formatter -archive file.outfmt11 -outfmt 6 -out file.outfmt6
```

RUN hmmscan:
```
$hmmscan --cpu 6 --domtblout
/path/to/output/nR_nK.Trinity.fasta_longest_orfs.pep.domtblout
/path/to/PfamDB/Pfam-A.hmm
/path/to/longest_orfs/nR_nK.Trinity.fasta_longest_orfs.pep
```

Transdecoder.Predict must be run in the directory that now contains the nR_nK.Trinity.fasta_transdecoder_dir (where the longest_orfs.pep file is):
```
$/path/to/TransDecoder-2.0.1/TransDecoder.Predict -t
/path/to/assembly/nR_nK.Trinity.fasta    --retain_long_orfs <length in nt of
ORFs to keep even if they had no hit> --retain_pfam_hits
/path/to/nR_nK.Trinity.fasta_longest_orfs.pep.domtblout  --retain_blastp_hits
/path/to/blast/output/nR_nK.Trinity.fasta_longest_orfs.pep_blastp_to_22316_LepRe
fSeq.outfmt6
```

The output from transdecoder.Predict contains "*" symbols. These must be removed before further analysis.
```
$sed -i 's/\*//g' nR_nK.Trinity.fasta_transdecoder.pep
```

19. QC again with TrinStats, Busco
See step 7

20. Do a BlastP search against LepRefSeq DB.
```
$blastp -query
/path/to/transdecoder_peptides/nR_nK.Trinity.fasta_transdecoder.pep -db
/mnt/data27/oppenheim/BlastDBs/22316_LepRefSeq.DB -max_target_seqs 1 -outfmt 11
-evalue 1e-5 -num_threads 4 -out
/path/to/output/nR_nK.Trinity.fasta_transdecoder.pep_blastp_to_22316_LepRefSeq.1
1
```

21. Assess completeness of transcripts (analyze_blastPlus_topHit_coverage.pl)

See step 12

22. For sequences that had no hit against the LepRefSeq DB, do a search against all of RefSeq.
Extract the "no hits" IDs from the blast.xml file (perl script "NoHit_XML_parser.pl")

Use ID list to make a "no hits" fasta file by:

Make blast DB of the peptide assembly:

```
$makeblastdb -in /path/to/nR_nK.Trinity.fasta_transdecoder.pep -dbtype prot -parse_seqids -out nR_nK.Trinity.fasta_transdecoder.pep.DB
```

Extract fasta sequences for the "no hits" set:

```
$blastdbcmd -db nR_nK.Trinity.fasta_transdecoder.pep.DB -dbtype prot -entry_batch NoHits.list -outfmt %f -out nR_nK.Trinity.fasta_transdecoder.pep.NoHits.fasta
```

Blast the no hits set:

```
$blastp -query /path/to/nR_nK.Trinity.fasta_transdecoder.pep.NoHits.fasta -db refseq_prot -max_target_seqs 1 -outfmt 11 -evalue 1e-5 -num_threads <N> -out /path/to/output/nR_nK.Trinity.fasta_transdecoder.pep_blastp_to_AllRefSeq.11
```

23. For sequences that still have no hit, do a search against all non-RefSeq lepidoptera.
Repeat step 22 for sequences that had no hit against the non-RefSeq lepidoptera to get the new "no hits" set, then blast against the nr DB.

24. For sequences that still have no hit, do FFPred.
Repeat above steps to get a final "no hits" set.

```
$perl /path/to/FFPred2/FFPred.pl -i /path/to/final_no_hits_set.fasta -o /path/to/FFPred/output/directory
```

FFPred runs these tools:

- In-house C++code to characterize amino acid composition
- In-house C++code to identify Sequence features
- MEMSAT-SVM to identify transmembrane segments
- PSIPRED 3.3 to predict secondary structure PSIPRED 3.3
- DISOPRED 2.43 to predict intrinsically disordered regions
- SignalP 4.0 to identify signal peptides
- WoLF PSORT 0.2 to identify subcellular localization
- epestfind in EMBOSS 6.4.0 to identify PEST regions
- Pfilt to identify low complexity regions
- COILS 2.2 to identify coiled coils
- NetPhos 3.1 to identify Phosphorylation sites
- NetNGlyc 1.0c to identify N-linked glycosylation sites
- NetOGlyc 3.1d to identify O-GalNAc-glycosylation sites

25. Run Interproscan.

```
$/path/to/interproscan_55/interproscan-5.16-55.0/interproscan.sh --input /path/to/nR_nK.Trinity.fasta_transdecoder.pep --formats xml --output-file-base /path/to/IPS_output --iprlookup --goterms --pathways --tempdir /path/to/interproscan_55/interproscan-5.16-55.0/TEMP --seqtype p
```

InterProScan runs these tools:

*SignalP_GRAM_POSITIVE (4.1) : SignalP (organism type gram-positive prokaryotes) predicts the presence and location of signal peptide cleavage sites in amino acid sequences for gram-positive prokaryotes.
*Hamap (201511.02) : High-quality Automated and Manual Annotation of Microbial Proteomes
*ProDom (2006.1) : ProDom is a comprehensive set of protein domain families automatically generated from the UniProt Knowledge Database.
*TMHMM (2.0c) : Prediction of transmembrane helices in proteins
*SignalP_EUK (4.1) : SignalP (organism type eukaryotes) predicts the presence and location of signal peptide cleavage sites in amino acid sequences for eukaryotes.
*PANTHER (10.0) : The PANTHER (Protein ANalysis THrough Evolutionary Relationships) Classification System is a unique resource that classifies genes by their functions, using published scientific experimental evidence and evolutionary relationships to predict function even in the absence of direct experimental evidence.
*SMART (6.2) : SMART allows the identification and analysis of domain architectures based on Hidden Markov Models or HMMs
*Phobius (1.01) : A combined transmembrane topology and signal peptide predictor
*PRINTS (42.0) : A fingerprint is a group of conserved motifs used to characterise a protein family
*SUPERFAMILY (1.75) : SUPERFAMILY is a database of structural and functional annotation for all proteins and genomes.
*PIRSF (3.01) : The PIRSF concept is being used as a guiding principle to provide comprehensive and non-overlapping clustering of UniProtKB sequences into a hierarchical order to reflect their evolutionary relationships.
*Pfam (28.0) : A large collection of protein families, each represented by multiple sequence alignments and hidden Markov models (HMMs)
*Gene3D (3.5.0) : Structural assignment for whole genes and genomes using the CATH domain structure database
*Coils (2.2.1) : Prediction of Coiled Coil Regions in Proteins
*ProSiteProfiles (20.113) : PROSITE consists of documentation entries describing protein domains, families and functional sites as well as associated patterns and profiles to identify them
*TIGRFAM (15.0) : TIGRFAMs are protein families based on Hidden Markov Models or HMMs
*ProSitePatterns (20.113) : PROSITE consists of documentation entries describing protein domains, families and functional sites as well as associated patterns and profiles to identify them
*SignalP_GRAM_NEGATIVE (4.1) : SignalP (organism type gram-negative prokaryotes) predicts the presence and location of signal peptide cleavage sites in amino acid sequences for gram-negative prokaryotes.
*SFLD (2) : SFLDs are protein families based on Hidden Markov Models or HMMs
*CDD (3.14) : Prediction of CDD domains in Proteins
*MobiDBLite (1.0) : Prediction of disordered domains Regions in Proteins

26. Ortholog evaluation

Generate the TaxID file:

```
+<taxid1> <absolute path fasta filename1>
+Species1   /path/to/Species1.fasta
.
.
```

+SpeciesN /path/to/SpeciesN.fasta

Create new directory and enter:
$mkdir Stemborer_OrthoDB
$cd Stemborer_OrthoDB

Run interactive setup script:
$/path/to/OrthoDB_soft_2.3.1/ORTHOPIPE-6.0.4/bin/setup.sh

This will generate a script: setup_project_soppenheim.sh
Running setup_project_soppenheim.sh will set up the project directory, and generate a pipeline.sh script

Check the pipeline script:
$/path/to/project_directory/pipeline.sh -xp

Run OrthoPipe to cluster sequences:
$/path/to/project_directory/pipeline.sh -r all

Parameters used:

```
export DIR_PIPELINE=/array1/soppenheim/src/OrthoDB_soft_2.3.1/ORTHOPIPE-6.0.4
export DIR_ORTHOPIPE=/array1/soppenheim/src/OrthoDB_soft_2.3.1/ORTHOPIPE-6.0.4
export DIR_PROJECT=/home/soppenheim/array1/stemborer_orthoDB/423_Run
export PL_TODO=423_ODb.todo
export COMPRESS_DATA=0
export DATA_TYPE=PROT
export DIR_BRHCLUS=/home/soppenheim/array1/src//OrthoDB_soft_2.3.1/BRHCLUS-2.1.7/bin
export DIR_BLAST=/usr/local/software/bin
export DIR_BLASTPLUS=/usr/local/software/bin
export DIR_PARALIGN=
export DIR_SWIPE=/home/soppenheim/array1/src//swipe/Linux
export DIR_CDHIT=/home/soppenheim/array1/src//cdhit
export DIR_WUBLAST=
export LIC_PARALIGN=
export ALIGNMENT_LABEL=SWIPE
export MASKER_LABEL=SEGMASKER
export SELECT_LABEL=CDHIT
export CLUSTER_LABEL=BRHCLUS
export SCHEDULER_LABEL=NONE
export MIN_OVERLAP=50
export SELECT_PID=97
export MAX_EVALUE=1.0e-5
export ALIGNMENT_MAXEVAL_SCALE=100.0
export ALIGNMENT_NUMALIGNMENTS=100
export ALIGNMENT_EFFDBSZ=0
export ALIGNMENT_MATRIX=0
export BRHCLUS_PAIREVAL_SCALE=0.001
export BRHCLUS_OPTS=
export OP_NJOBMAX_BATCH=200
export OP_NJOBMAX_LOCAL=25
```

Final cluster file is /path/to/project_directory/Clusters/MyProject.og

Post-processing:
Associate SeqIDs used in clusters with original sequence IDs:

Remove header stuff from the MyProject.og:
```
$sed '/^#/d' MyProject.og > NewFile.og
```

Concatenate all the fs.maptxt files:
```
$cat Species1.fs.maptxt ... SpeciesN.fs.maptxt > AllSpecies.fs.maptxt
```

Sort the .og and .maptext files by the ODb TaxID:
```
$sort AllSpecies.fs.maptxt > AllSpecies.fs.maptxt.sorted
$sort NewFile.og -k2 > NewFile.og.sorted
```

Join them by the TaxID:
```
$join -1 1 -2 2 AllSpecies.fs.maptxt.sorted NewFile.og.sorted -t $'\t' >
BothNames_NewFile.og
```

Extract only needed information:
```
$cut -f1-3,10 BothNames_NewFile.og > Limited_BothNames_NewFile.og
```

Convert ODbID into species ID:
```
$sed -i 's/:.*\t/\t/g' Limited_BothNames_NewFile.og
```

Add a header line:
```
$sed -i '1i SpeciesID\tClusterID\tCluster_type\tOriginal_SeqID'
Limited_BothNames_NewFile.og
```

27. Find "species-specific" genes (those that that did not cluster):

Restore original names to clustered sequences:
```
$ ./sbin/remap.py -f Cluster/MyProject.og -m Rawdata/SpeciesOne.fs.maptxt
-m Rawdata/SpeciesTwo.fs.maptxt -m Rawdata/SpeciesThree.fs.maptxt -k >
MyProject_OriginalIDs.og
```

Reformat:
```
$sed -i 's/ /\t/g' MyProject_OriginalIDs.og
```

Get ID column only:
```
$cut -f2 MyProject_OriginalIDs.og>ClusteredSeqs_OriginalIDs.txt
```

Sort:
```
$sort ClusteredSeqs_OriginalIDs.txt -o ClusteredSeqs_OriginalIDs.txt
```

Reformat list of all sequences:
```
$sed -i 's/ /\t/g' Rawdata/all.fs.maptxt
```

Get ID column only:
```
$cut -f2 Rawdata/all.fs.maptxt> AllSeqIDs.txt
```

Sort:
```
$sort AllSeqIDs.txt -o AllSeqIDs.txt
```

Compare clustered list to full SeqID list, extract the IDs found only in the full list:
```
$comm -13 ClusteredSeqs_OriginalIDs.txt
AllSeqIDs.txt>NotClusteredSeqIDs.txt
```

28. GO term mapping with Blast2GO

FFPred results must be parsed into a Blast2GO-style .annot file before they can be imported. Use perl script "parse_ffpred_B2G.pl"

Import into Blast2GO as three different studies, otherwise the blast hits overwrite as they are loaded:

1) nR_nK.Trinity.fasta_transdecoder.pep (fasta file), blast results from LepRefSeq (xml), Interproscan results (xml), and FFPred results (as .annot; do by using "load annotations" command)
2) nR_nK.Trinity.fasta_transdecoder.pep and blast results from AllRefSeq
3) nR_nK.Trinity.fasta_transdecoder.pep and blast results from Not_RefSeq

For each study, do mapping and annotation as described in Blast2GO manual.

For studies 2 and 3, export annotations, then import them into study 1. This will add the blast results without overwriting. Once everything is in one study, merge Interproscan to GO annotation, then procede with other analyses.

29. Functional enrichment tests in Blast2GO
Using the sequence lists created in step 27, test whether GO terms or InterPro signatures are over- or under-represented in species-specific genes. In Blast2GO, run Fisher's Exact Test with a specified test and reference set.

30. Functional annotation and comparison of species-specific genes
CD-Search analyses conducted online at https://www.ncbi.nlm.nih.gov/Structure/bwrpsb/bwrpsb.cgi

Parameters used:
Data source: CDSEARCH/cdd v3.16
E-Value cut-off: 0.01
Composition-corrected scoring: Applied
Low-complexity regions: Not filtered

BLASTp searches against RefSeq with species-specific genes that had CD-Search hits to retrotransposon families

Extract the Lepidoptera and top non-Lepidoptera hit sequences

Using Muscle, align SSGs and the extracted hit sequences:

```
$muscle -in Seqs_plus_RefSeqs.fasta -out Seqs_plus_RefSeqs.Muscle.alignment
```

Make a tree:

```
$/path/to/FastTree Seqs_plus_RefSeqs.Muscle.alignment > Seqs_plus_RefSeqs.Muscle.alignment.tree
```

Visualize tree with FigTree desktop dmg
