# Supplementary material for: De Novo characterization of transcriptomes from two North American Papaipema stem-borers (Lepidoptera: Noctuidae)
Source: PLoS One. 2018 Jan 24;13(1):e0191061. doi: 10.1371/journal.pone.0191061 (PMC5783364; doi:10.1371/journal.pone.0191061)
Supplement: S1 Table — (PDF) [file pone.0191061.s008.pdf]

Supplemental Table 1. Most frequent InterPro signatures.

**InterPro signature**	**InterPro description**	***Papaipema sp.4***	***Papaipema speciosissima***
IPR005828	Major facilitator, sugar transporter-like	134	143
IPR008042	Retrotransposon, Pao	126	179
IPR000276	G protein-coupled receptor, rhodopsin-like	115	80
IPR003579	Small GTPase superfamily, Rab type	111	101
IPR001806	Small GTPase superfamily	100	98
IPR011701	Major facilitator superfamily	98	153
IPR005312	Protein of unknown function DUF1759	97	134
IPR001128	Cytochrome P450	94	136
IPR020849	Small GTPase superfamily, Ras type	87	87
IPR002198	Short-chain dehydrogenase/reductase SDR	86	112
IPR001314	Peptidase S1A, chymotrypsin-type	81	99
IPR006201	Neurotransmitter-gated ion-channel	76	31
IPR003578	Small GTPase superfamily, Rho type	74	82
IPR002347	Glucose/ribitol dehydrogenase	71	98
IPR026983	Dynein heavy chain	57	53
IPR003663	Sugar/inositol transporter	44	43
IPR001395	Aldo/keto reductase/potassium channel subunit beta	44	34
IPR004875	DDE superfamily endonuclease, CENP-B-like	44	32
IPR006111	Archaeal RpoK/eukaryotic RPB6 RNA polymerase subunit	40	37
IPR013126	Heat shock protein 70 family	40	20
IPR010562	Haemolymph juvenile hormone binding	39	40
IPR021896	Transposase protein	39	40
IPR001071	Cellular retinaldehyde binding/alpha-tocopherol transport	38	46
IPR001757	P-type ATPase	38	46
IPR024156	Small GTPase superfamily, ARF type	38	29
IPR029033	Histidine phosphatase superfamily	37	66
IPR002041	Ran GTPase	37	49
IPR022048	Protein of unknown function DUF3609	36	24
IPR027640	Kinesin-like protein	35	37
IPR018499	Tetraspanin/Peripherin	35	32
IPR006028	Gamma-aminobutyric acid A receptor/Glycine receptor alpha	35	18
IPR000215	Serpin family	34	42
IPR026055	Fatty acyl-CoA reductase	33	47
IPR000618	Insect cuticle protein	32	47
IPR002293	Amino acid/polyamine transporter I	32	27
IPR003929	Potassium channel, calcium-activated, BK, alpha subunit	31	20
IPR028325	Voltage-gated potassium channel	30	30
IPR020471	Aldo/keto reductase	30	27
IPR002213	UDP-glucuronosyl/UDP-glucosyltransferase	29	56
IPR000832	GPCR, family 2, secretin-like	29	52
IPR022812	Dynamin superfamily	29	35
IPR006539	P-type ATPase, subfamily IV	28	29

InterPro signature	InterPro description	*Papaipema sp.4*	*Papaipema speciosissima*
IPR006170	Pheromone/general odorant binding protein	28	24
IPR015925	Ryanodine receptor-related	28	18
IPR000301	Tetraspanin	27	30
IPR002159	CD36 antigen	27	19
IPR002077	Voltage-dependent calcium channel, alpha-1 subunit	27	17
IPR005055	Insect odorant-binding protein A10/Ejaculatory bulb-specific protein 3	26	45
IPR001930	Peptidase M1, alanine aminopeptidase/leukotriene A4 hydrolase	26	25
IPR006689	Small GTPase superfamily, ARF/SAR type	26	18
IPR000175	Sodium:neurotransmitter symporter	26	18
IPR000734	Triacylglycerol lipase family	25	41
IPR026082	ABC transporter A, ABCA	25	29
IPR024939	Calcium-activated potassium channel Slo	24	25
IPR030564	Myotubularin family	24	20
IPR010285	DNA helicase Pif1-like	24	9
IPR000560	Histidine phosphatase superfamily, clade-2	23	39
IPR001734	Sodium/solute symporter	23	25
IPR000648	Oxysterol-binding protein	23	24
IPR015433	Phosphatidylinositol Kinase	23	24
IPR002067	Mitochondrial carrier protein	22	22
IPR004344	Tubulin-tyrosine ligase/Tubulin polyglutamylase	22	17
IPR024936	Cyclophilin-type peptidyl-prolyl cis-trans isomerase	21	29
IPR001723	Nuclear hormone receptor	20	35
IPR000286	Histone deacetylase superfamily	20	32
IPR026791	Dedicator of cytokinesis	20	23
IPR031107	Small heat shock protein HSP20	20	22
IPR001353	Proteasome, subunit alpha/beta	20	17
IPR013128	Peptidase C1A	19	39
IPR001888	Transposase, type 1	19	25
IPR020636	Calcium/calmodulin-dependent/calcium-dependent protein kinase	19	24
IPR001436	Alpha crystallin/Heat shock protein	19	22
IPR012132	Glucose-methanol-choline oxidoreductase	19	16
IPR022058	Protein of unknown function DUF3610	19	6
IPR002076	ELO family	18	36
IPR027244	Vacuolar membrane-associated protein Iml1	18	25
IPR004299	Membrane bound O-acyl transferase, MBOAT	18	16
IPR017996	Major royal jelly protein/Protein yellow	18	15
IPR006077	Vinculin/alpha-catenin	18	15
IPR003280	Two pore domain potassium channel	18	14
IPR010255	Haem peroxidase	17	25
IPR019791	Haem peroxidase, animal	17	25
IPR011603	2-oxoglutarate dehydrogenase E1 component	17	23
IPR031734	Transcription activator MBF2	17	19
IPR015902	Glycoside hydrolase, family 13	17	17

InterPro signature	InterPro description	*Papaipema sp.4*	*Papaipema speciosissima*
IPR004117	Olfactory receptor, Drosophila	17	10
IPR026847	Vacuolar protein sorting-associated protein 13	17	10
IPR002129	Pyridoxal phosphate-dependent decarboxylase	17	8
IPR009053	Prefoldin	16	29
IPR000718	Peptidase M13	16	27
IPR028846	Recoverin family	16	26
IPR003840	DNA helicase	16	21
IPR024950	Dual specificity phosphatase	16	21
IPR001360	Glycoside hydrolase, family 1	16	17
IPR018422	Cation/H+ exchanger, CPA1 family	16	7
IPR005522	Inositol polyphosphate kinase	15	18
IPR005446	Voltage-dependent calcium channel, L-type, alpha-1 subunit	15	15
IPR002423	Chaperonin Cpn60/TCP-1 family	15	14
IPR001508	Ionotropic glutamate receptor, metazoa	15	14
IPR031327	Mini-chromosome maintenance protein	15	12
IPR003968	Potassium channel, voltage dependent, Kv	15	12
IPR002490	V-type ATPase, V0 complex, 116kDa subunit family	14	30
IPR001753	Crotonase superfamily	14	26
IPR001873	Na+ channel, amiloride-sensitive	14	24
IPR000798	Ezrin/radixin/moesin-like	14	17
IPR000322	Glycoside hydrolase family 31	14	15
IPR007122	Villin/Gelsolin	14	15
IPR008734	Phosphorylase kinase alpha/beta subunit	14	14
IPR000462	CDP-alcohol phosphatidyltransferase	14	13
IPR000092	Polyprenyl synthetase	14	13
IPR001548	Peptidase M2, peptidyl-dipeptidase A	14	12
IPR001902	SLC26A/SulP transporter	14	12
IPR015500	Peptidase S8, subtilisin-related	14	10
IPR024134	Superoxide dismutase (Cu/Zn) / superoxide dismutase copper chaperone	14	10
IPR004142	NDRG	14	6
IPR015868	Glutaminase	14	5
IPR004709	Na+/H+ exchanger	14	5
IPR000217	Tubulin	13	21
IPR004000	Actin family	13	20
IPR004878	Otopetrin	13	15
IPR015510	Peptidoglycan recognition protein	13	14
IPR013788	Hemocyanin/hexamerin	13	13
IPR013333	Ryanodine receptor	13	11
IPR026103	Harbinger transposase-derived nuclease, animal	13	9
IPR004835	Chitin synthase	13	8
IPR002455	GPCR family 3, GABA-B receptor	13	0
IPR001619	Sec1-like protein	12	26
IPR023566	Peptidyl-prolyl cis-trans isomerase, FKBP-type	12	22

InterPro signature	InterPro description	*Papaipema sp.4*	*Papaipema speciosissima*
IPR019323	Active zone protein ELKS	12	19
IPR006329	AMP deaminase	12	18
IPR000615	Bestrophin	12	16
IPR021134	Bestrophin/UPF0187	12	16
IPR023088	3'5'-cyclic nucleotide phosphodiesterase	12	14
IPR004162	E3 ubiquitin-protein ligase SIN-like	12	14
IPR000413	Integrin alpha chain	12	14
IPR000120	Amidase	12	12
IPR003689	Zinc/iron permease	12	11
IPR023610	Phosphatidylinositol-4-phosphate 5-kinase	12	10
IPR031127	E3 ubiquitin ligase RBR family	12	9
IPR002610	Peptidase S54, rhomboid	12	9
IPR023332	Proteasome A-type subunit	12	8
IPR001404	Heat shock protein Hsp90 family	12	6
IPR003286	RNA-directed DNA polymerase, eukaryota	12	6
IPR001067	Nuclear translocator	11	15
IPR015655	Protein phosphatase 2C family	11	12
IPR002317	Serine-tRNA ligase, type1	11	12
IPR017998	Chaperone tailless complex polypeptide 1 (TCP-1) amidotransferase, subunit B /E	11	11
IPR014472	Choline/ethanolamine phosphotransferase	11	11
IPR008530	Coiled-coil domain-containing protein 22	11	11
IPR001503	Glycosyl transferase family 10	11	11
IPR005331	Sulfotransferase	11	11
IPR025705	Beta-hexosaminidase	11	10
IPR002343	Paraneoplastic encephalomyelitis antigen	11	10
IPR002616	tRNA-guanine(15) transglycosylase-like	11	9
IPR022953	ATP-dependent 6-phosphofructokinase	11	7
IPR028559	Filamin A	11	6
IPR001804	Isocitrate and isopropylmalate dehydrogenases family	11	6
IPR015812	Integrin beta subunit	10	27
IPR013078	Histidine phosphatase superfamily, clade-1	10	25
IPR000533	Tropomyosin	10	24
IPR007603	Choline transporter-like	10	22
IPR001192	Phosphoinositide phospholipase C family	10	21
IPR003000	Sirtuin family	10	21
IPR000425	Major intrinsic protein	10	19
IPR001563	Peptidase S10, serine carboxypeptidase	10	18
IPR002453	Beta tubulin	10	16
IPR001164	Arf GTPase activating protein	10	15
IPR001978	Troponin	10	14
IPR011613	Glycoside hydrolase family 15/Phosphorylase b kinase regulatory chain family	10	13
IPR004119	Protein of unknown function DUF227	10	13
IPR004031	PMP-22/EMP/MP20/Claudin superfamily	10	12

InterPro signature	InterPro description	*Papaipema sp.4*	*Papaipema speciosissima*
IPR019410	Lysine methyltransferase	10	11
IPR007248	Mpv17/PMP22	10	11
IPR005024	Snf7 family	10	11
IPR005599	GPI mannosyltransferase	10	10
IPR030217	Nuclear RNA export factor	10	10
IPR008914	Phosphatidylethanolamine-binding protein PEBP	10	10
IPR025799	Protein arginine N-methyltransferase	10	10
IPR000698	Arrestin	10	9
IPR000542	Acyltransferase ChoActase/COT/CPT	10	8
IPR005746	Thioredoxin	10	8
IPR025956	Cytoplasmic dynein 1 intermediate chain 1/2	10	7
IPR026739	AP complex subunit beta	10	6
IPR026962	Katanin p80 subunit B1	10	5
IPR000597	Ribosomal protein L3	10	3
IPR002394	Nicotinic acetylcholine receptor	10	2
IPR004481	Sodium/potassium/calcium exchanger	10	2
IPR002289	Gamma-aminobutyric-acid A receptor, beta subunit	10	1
IPR024138	Pericentriolar material 1 protein	9	20
IPR002524	Cation efflux protein	9	13
IPR022684	Peptidase C2, calpain family	9	12
IPR006439	HAD hydrolase, subfamily IA	9	11
IPR001696	Voltage gated sodium channel, alpha subunit	9	11
IPR000850	Adenylate kinase/UMP-CMP kinase	9	10
IPR018150	Aminoacyl-tRNA synthetase, class II (D/K/N)=-like	9	10
IPR027663	Dynactin subunit 1	9	10
IPR007259	Gamma-tubulin complex component protein	9	9
IPR001807	Chloride channel, voltage gated	9	8
IPR018017	Nucleoside phosphorylase	9	8
IPR028427	Peptide methionine sulfoxide reductase	9	8
IPR005511	Senescence marker protein-30 (SMP-30)	9	7
IPR005016	Serine incorporator/TMS membrane protein	9	7
IPR031106	CCAAT/enhancer-binding protein C/EBP	9	6
IPR006598	Lipopolysaccharide-modifying protein	9	6
IPR000611	Neuropeptide Y receptor family	9	6
IPR004156	Organic anion transporter polypeptide OATP	9	6
IPR027094	Mitofusin family	9	5
IPR003938	Potassium channel, voltage-dependent, EAG/ELK/ERG	9	4
IPR016092	FeS cluster insertion protein	9	3
IPR015680	Glutamate-Gated Chloride Channel	9	3
IPR024931	Importin subunit alpha	9	1
IPR015688	Elongation Factor 3	9	0
IPR007632	Anoctamin	8	26
IPR019956	Ubiquitin	8	23

InterPro signature	InterPro description	*Papaipema sp.4*	*Papaipema speciosissima*
IPR001464	Annexin	8	21
IPR003084	Histone deacetylase	8	20
IPR013790	Dwarfin	8	18
IPR015505	Coronin	8	16
IPR001019	Guanine nucleotide binding protein (G-protein) , alpha subunit	8	15
IPR001310	Histidine triad (HIT) protein	8	15
IPR032135	Protein of unknown function DUF4817	8	14
IPR006941	Ribonuclease CAF1	8	13
IPR013657	UAA transporter	8	13
IPR027072	Heat shock factor protein 1	8	11
IPR027725	Heat shock transcription factor family	8	11
IPR014371	Sterol O-acyltransferase, ACAT/DAG/ARE types	8	11
IPR002777	Prefoldin beta-like	8	10
IPR023267	RNA (C5-cytosine) methyltransferase	8	10
IPR006179	5'-Nucleotidase/apyrase	8	9
IPR006838	FAR-17a/AIG1-like protein	8	8
IPR021190	Peptidase M10A	8	8
IPR002591	Type I phosphodiesterase/nucleotide pyrophosphatase/phosphate transferase	8	7
IPR030364	K/Cl co-transporter 3	8	6
IPR016355	Nuclear hormone receptor family 5	8	6
IPR000246	Peptidase T2, asparaginase 2	8	6
IPR000682	Protein-L-isoaspartate(D-aspartate) O-methyltransferase	8	6
IPR019427	7TM GPCR, serpentine receptor class w (Srw)	8	5
IPR027775	C2H2- zinc finger protein family	8	5
IPR004245	Protein of unknown function DUF229	8	5
IPR006875	Sarcoglycan complex subunit protein	8	4
IPR025958	SID1 transmembrane family	8	4
IPR000584	Voltage-dependent calcium channel, L-type, beta subunit	8	4
IPR010977	Aromatic-L-amino-acid decarboxylase	8	3
IPR027741	Dynamin-1	8	3
IPR000228	RNA 3'-terminal phosphate cyclase	8	3
IPR027080	Uncoordinated protein 13 (Unc-13)	8	3
IPR002113	Adenine nucleotide translocator 1	8	2
IPR001055	Adrenodoxin	8	2
IPR006032	Ribosomal protein S12/S23	8	2
IPR029158	Stimulator of interferon genes protein	8	1
IPR002456	GPCR family 3, gamma-aminobutyric acid receptor, type B1	8	0
IPR015449	Potassium channel, calcium-activated, SK	7	26
IPR004345	TB2/DP1/HVA22-related protein	7	22
IPR000333	Ser/Thr protein kinase, TGFB receptor	7	17
IPR005062	SAC3/GANP/THP3	7	16
IPR030376	Cap-specific mRNA (nucleoside-2-O-)-methyltransferase 1	7	11
IPR031057	DOCK1 homologue	7	11

InterPro signature	InterPro description	*Papaipema sp.4*	*Papaipema speciosissima*
IPR015876	Fatty acid desaturase, type 1, core	7	11
IPR000649	Initiation factor 2B-related	7	11
IPR005037	Pre-mRNA-splicing factor 38	7	11
IPR002495	Glycosyl transferase, family 8	7	10
IPR027005	Glycosyltransferase 39-like	7	10
IPR008380	HAD-superfamily hydrolase, subfamily IG, 5'-nucleotidase	7	10
IPR009886	HCaRG	7	10
IPR000760	Inositol monophosphatase	7	10
IPR010291	Ion channel regulatory protein, UNC-93	7	10
IPR031828	Myofilin	7	10
IPR005552	Scramblase	7	10
IPR003392	Patched	7	9
IPR005282	Lysosomal cystine transporter	7	8
IPR006544	P-type ATPase, subfamily V	7	8
IPR023333	Proteasome B-type subunit	7	8
IPR027231	Semaphorin	7	8
IPR021151	GINS complex	7	7
IPR003057	Invertebrate colouration protein	7	7
IPR026113	Methyltransferase-like	7	7
IPR001666	Phosphatidylinositol transfer protein	7	7
IPR012464	Protein of unknown function DUF1676	7	7
IPR002139	Ribokinase	7	7
IPR004263	Exostosin-like	7	6
IPR004434	Isocitrate dehydrogenase NAD-dependent	7	6
IPR029559	Membrane protein Tms1-like	7	6
IPR008758	Peptidase S28	7	6
IPR017446	Polyprenyl synthetase-related	7	6
IPR028820	Sodium channel protein 60E	7	6
IPR005033	YEATS	7	6
IPR012258	Acyl-CoA oxidase	7	5
IPR001580	Calreticulin/calnexin	7	5
IPR023561	Carbonic anhydrase, alpha-class	7	5
IPR000647	CTF transcription factor/nuclear factor 1	7	5
IPR003378	Fringe-like	7	5
IPR029136	Nuclear protein MDM1	7	5
IPR023608	Protein-glutamine gamma-glutamyltransferase, eukaryota	7	5
IPR001406	Pseudouridine synthase I, TruA	7	5
IPR000764	Uridine kinase-like	7	5
IPR002331	Pancreatic lipase	7	4
IPR006916	Popeye protein	7	4
IPR027705	Flotillin family	6	22
IPR003094	Fructose-2,6-bisphosphatase	6	14
IPR005814	Aminotransferase class-III	6	13

InterPro signature	InterPro description	*Papaipema sp.4*	*Papaipema speciosissima*
IPR001130	TatD family	6	12
IPR027251	Diacylglycerol O-acyltransferase 1	6	9
IPR030224	Sla2 family	6	9
IPR015720	TMP21-related	6	9
IPR005946	Ribose-phosphate diphosphokinase	6	8
IPR017351	LIM and senescent cell antigen-like-containing domain protein 1	5	19
IPR006084	XPG/Rad2 endonuclease	5	15
IPR016449	Potassium channel, inwardly rectifying, Kir	5	14
IPR004127	Prefoldin alpha-like	5	13
IPR006214	Bax inhibitor 1-related	5	12
IPR002659	Glycosyl transferase, family 31	5	12
IPR001273	Aromatic amino acid hydroxylase	5	11
IPR001382	Glycoside hydrolase family 47	5	11
IPR005937	26S proteasome subunit P45	5	10
IPR001916	Glycoside hydrolase, family 22	5	10
IPR026314	YLP motif-containing protein 1	5	10
IPR007274	Ctr copper transporter	5	9
IPR031720	Protein of unknown function DUF4728	5	9
IPR002155	Thiolase	5	9
IPR015712	DNA-directed RNA polymerase, subunit 2	5	8
IPR000639	Epoxide hydrolase-like	5	8
IPR019416	Nuclear cap-binding protein subunit 3	5	8
IPR029585	Peroxidase, insect	5	8
IPR026827	Proteasome component ECM29/Translational activator GCN1	5	8
IPR028669	Syntaxin 1	5	8
IPR010678	Digestive organ expansion factor, predicted	4	27
IPR003859	Beta-1,4-galactosyltransferase	4	21
IPR009145	U2 auxiliary factor small subunit	4	21
IPR027272	Piezo family	4	19
IPR007258	Vps52	4	15
IPR001661	Glycoside hydrolase, family 37	4	14
IPR028516	Sorbin and SH3 domain-containing protein 2	4	14
IPR012388	CDK5 and ABL1 enzyme substrate 1/2	4	12
IPR006509	Splicing factor, RBM39-like	4	11
IPR008011	Complex 1 LYR protein	4	10
IPR031259	Intracellular lipid binding protein	4	10
IPR001714	Peptidase M24, methionine aminopeptidase	4	10
IPR015615	Transforming growth factor-beta-related	4	10
IPR000101	Gamma-glutamyltranspeptidase	4	9
IPR032063	Protein of unknown function DUF4804	4	9
IPR005026	SAPAP family	4	9
IPR004316	SWEET sugar transporter	4	9
IPR000672	Tetrahydrofolate dehydrogenase/cyclohydrolase	4	9

**InterPro signature**	**InterPro description**	***Papaipema sp.4***	***Papaipema speciosissima***
IPR021629	Mediator complex, subunit Med23	3	17
IPR031214	Ecotropic viral integration site 5 protein	3	13
IPR001269	tRNA-dihydrouridine synthase	3	12
IPR009077	Proteasome activator pa28	3	11
IPR000003	Retinoid X receptor/HNF4	3	11
IPR023600	Folylpolyglutamate synthase, eukaryota	3	10
IPR001645	Folylpolyglutamate synthetase	3	10
IPR027986	T-cell activation inhibitor, mitochondrial	3	10
IPR001217	Transcription factor STAT	3	10
IPR008551	Transport and Golgi organisation protein 2	3	10
IPR013935	TRAPP II complex, Trs120	3	10
IPR001171	Ergosterol biosynthesis ERG4/ERG24	3	9
IPR002467	Peptidase M24A, methionine aminopeptidase, subfamily 1	3	9
IPR026059	Rab3-GAP regulatory subunit	3	9
IPR001958	Tetracycline resistance protein TetA/multidrug resistance protein MdtG	3	9
IPR029705	UPF0505 family	3	9
IPR001312	Hexokinase	2	21
IPR007129	Ubiquinol-cytochrome c chaperone, CBP3	2	11
IPR021150	Ubiquinol-cytochrome c chaperone/UPF0174	2	11
IPR032062	Protein of unknown function DUF4803	2	10
IPR004908	ATPase, V1 complex, subunit H	2	9
IPR016208	Aldehyde oxidase/xanthine dehydrogenase	1	12
IPR015767	Rho GTPase activating protein	1	12
IPR008349	Mitogen-activated protein (MAP) kinase, ERK1/2	1	11
IPR019149	Protein of unknown function DUF2048	1	11
IPR019179	Coiled-coil domain-containing protein 149-A	1	10
IPR002151	Kinesin light chain	1	10
IPR011419	ATP12, ATPase F1F0-assembly protein	1	9
