# Supplementary material for: De Novo characterization of transcriptomes from two North American Papaipema stem-borers (Lepidoptera: Noctuidae)
Source: PLoS One. 2018 Jan 24;13(1):e0191061. doi: 10.1371/journal.pone.0191061 (PMC5783364; doi:10.1371/journal.pone.0191061)
Supplement: S2 Table — (PDF) [file pone.0191061.s009.pdf]

Supplemental Table 2. GO annotation evidence codes.

**Evidence type**	**Evidence Code**	***Papaipema sp.4***	***Papaipema speciosissima***	**Evidence code definition**
Automatically-Assigned	Inferred from Electronic Annotation (IEA)	19,414	16,147	Used for annotations that depend directly on computation or automated transfer of annotations from a database, particularly when the analysis is performed internally and not published. A key feature that distinguishes this evidence code from others is that it is not made by a curator; use IEA when no curator has checked the specific annotation to verify its accuracy
Computational Analysis	Inferred from Sequence or Structural Similarity (ISS)	6	16	The ISS evidence code or one of its sub-categories should be used whenever a sequence-based analysis forms the basis for an annotation and review of the evidence and annotation has been done manually. If the annotation has not been reviewed manually, the correct evidence code is IEA, even if the evidence supporting the annotation is all sequence based.
Experimental	Inferred from Direct Assay (IDA)	9	11	The IDA evidence code is used to indicate a direct assay was carried out to determine the function, process, or component indicated by the GO term.
Experimental	Inferred from Physical Interaction (IPI)	1	3	Covers physical interactions between the entity of interest and another molecule (such as a protein, ion or complex). IPI can be thought of as a type of IDA, where the actual binding partner or target can be specified, using "with" in the with/from field. The GO term ‘protein binding’ (GO:0005515) should only be used with the evidence code IPI and an identifier in the ‘with’ field. A reciprocal annotation must also be made to indicate the interaction in the opposite direction.
Experimental	Inferred from Mutant Phenotype (IMP)	1	4	The IMP evidence code covers those cases when the function, process or cellular localization of a gene product is inferred based on differences in the function, process, or cellular localization between two different alleles of the corresponding gene. The IMP code is used for cases where one allele may be designated 'wild-type' and another as 'mutant'. It is also used in cases where allelic variation occurs naturally and no specific allele is designated as wild-type or mutant.
