# Supplementary material for: De Novo characterization of transcriptomes from two North American Papaipema stem-borers (Lepidoptera: Noctuidae)
Source: PLoS One. 2018 Jan 24;13(1):e0191061. doi: 10.1371/journal.pone.0191061 (PMC5783364; doi:10.1371/journal.pone.0191061)
Supplement: S3 Table — (PDF) [file pone.0191061.s010.pdf]

Supplemental Table 3. InterPro signatures in shared and species-specific genes.

**InterPro signature**	**InterPro description**	**Shared *Papaipema* genes**	***Papaipema sp.4* SSGs**	***Papaipema speciosissima* SSGs**
IPR027417	P-loop containing nucleoside triphosphate hydrolase	4281	75	75
IPR007087	Zinc finger C2H2	3338	68	32
IPR015880	Zinc finger C2H2-like	3127	61	27
IPR011009	Protein kinase-like domain	2227	25	19
IPR000719	Protein kinase domain	1943	22	18
IPR016024	Armadillo-type fold	1847	12	8
IPR017986	WD40-repeat-containing domain	1788	21	19
IPR002290	Serine/threonine/dual specificity protein kinase catalytic domain	1369	7	9
IPR011993	PH domain-like	1288	16	17
IPR020846	Major facilitator superfamily domain	1258	24	35
IPR016040	NADP-binding domain	1195	25	37
IPR012677	Nucleotide-binding alpha-beta plait domain	1181	17	12
IPR029058	Alpha/Beta hydrolase fold	1145	31	24
IPR011990	Tetratricopeptide-like helical domain	1009	11	4
IPR000504	RNA recognition motif domain	1007	11	10
IPR012934	Zinc finger AD-type	974	37	19
IPR020683	Ankyrin repeat-containing domain	908	15	10
IPR007110	Immunoglobulin-like domain	870	25	46
IPR029063	S-adenosyl-L-methionine-dependent methyltransferase	847	2	2

InterPro signature	InterPro description	Shared *Papaipema* genes	*Papaipema sp.4* SSGs	*Papaipema speciosissima* SSGs
IPR014001	Helicase superfamily 1/2 ATP-binding domain	838	3	8
IPR001841	Zinc finger RING-type	825	3	4
IPR001650	Helicase C-terminal	779	3	7
IPR032675	Leucine-rich repeat domain L domain-like	757	5	19
IPR012337	Ribonuclease H-like domain	738	139	145
IPR003599	Immunoglobulin subtype	733	19	23
IPR003593	AAA+ ATPase domain	721	21	4
IPR001478	PDZ domain	682	9	4
IPR011992	EF-hand domain pair	661	12	6
IPR013098	Immunoglobulin I-set	659	19	33
IPR001452	SH3 domain	657	3	4
IPR013026	Tetratricopeptide repeat-containing domain	646	5	2
IPR003598	Immunoglobulin subtype 2	637	19	19
IPR011991	Winged helix-turn-helix DNA-binding domain	634	3	8
IPR001849	Pleckstrin homology domain	627	8	9
IPR012336	Thioredoxin-like fold	621	9	6
IPR011011	Zinc finger FYVE/PHD-type	614	3	4
IPR011701	Major facilitator superfamily	573	6	5
IPR000477	Reverse transcriptase domain	568	222	175
IPR005828	Major facilitator, sugar transporter-like	566	18	13

InterPro signature	InterPro description	Shared *Papaipema* genes	*Papaipema sp.4* SSGs	*Papaipema speciosissima* SSGs
IPR029071	Ubiquitin-related domain	555	3	3
IPR011545	DEAD/DEAH box helicase domain	553	3	7
IPR009057	Homeodomain-like	541	25	11
IPR011333	SKP1/BTB/POZ domain	530	13	4
IPR002048	EF-hand domain	528	9	5
IPR000210	BTB/POZ domain	502	12	4
IPR003579	Small GTPase superfamily, Rab type	486	5	4
IPR012340	Nucleic acid-binding OB-fold	479	1	7
IPR013320	Concanavalin A-like lectin/glucanase domain	472	5	7
IPR005225	Small GTP-binding protein domain	472	2	6
IPR001245	Serine-threonine/tyrosine-protein kinase catalytic domain	470	5	5
IPR001806	Small GTPase superfamily	466	6	5
IPR002198	Short-chain dehydrogenase/reductase SDR	465	7	6
IPR013761	Sterile alpha motif/pointed domain	457	2	0
IPR014756	Immunoglobulin E-set	446	6	7
IPR023214	HAD-like domain	443	6	4
IPR000742	EGF-like domain	431	26	13
IPR003961	Fibronectin type III	429	8	22
IPR000008	C2 domain	426	5	4
IPR009003	Peptidase S1 PA clan	422	14	27

**InterPro signature**	**InterPro description**	**Shared *Papaipema* genes**	***Papaipema sp.4* SSGs**	***Papaipema speciosissima* SSGs**
IPR002347	Glucose/ribitol dehydrogenase	422	6	5
IPR029021	Protein-tyrosine phosphatase-like	422	3	4
IPR020849	Small GTPase superfamily, Ras type	413	5	5
IPR001965	Zinc finger PHD-type	410	1	0
IPR005135	Endonuclease/exonuclease/phosphatase	409	49	32
IPR017853	Glycoside hydrolase superfamily	402	8	25
IPR003439	ABC transporter-like	402	22	7
IPR029044	Nucleotide-diphospho-sugar transferases	401	5	3
IPR023753	FAD/NADP-binding domain	399	4	13
IPR001128	Cytochrome P450	394	19	23
IPR001254	Serine proteases trypsin domain	388	13	25
IPR023395	Mitochondrial carrier domain	385	7	10
IPR003578	Small GTPase superfamily, Rho type	374	4	3
IPR002018	Carboxylesterase type B	369	17	9
IPR015424	Pyridoxal phosphate-dependent transferase	363	3	11
IPR008984	SMAD/FHA domain	361	2	2
IPR005821	Ion transport domain	360	17	16
IPR019787	Zinc finger PHD-finger	345	1	1
IPR000980	SH2 domain	338	1	1
IPR000571	Zinc finger CCCH-type	327	4	1

**InterPro signature**	**InterPro description**	**Shared *Papaipema* genes**	***Papaipema sp.4* SSGs**	***Papaipema speciosissima* SSGs**
IPR006578	MADF domain	324	4	3
IPR001584	Integrase catalytic core	301	109	95
IPR001314	Peptidase S1A, chymotrypsin-type	300	7	5
IPR014014	RNA helicase DEAD-box type Q motif	298	2	5
IPR009071	High mobility group box domain	294	2	5
IPR001623	DnaJ domain	289	0	0
IPR011598	Myc-type basic helix-loop-helix bHLH domain	287	3	1
IPR001810	F-box domain	284	1	4
IPR002035	von Willebrand factor type A	282	2	2
IPR003959	ATPase AAA-type core	277	1	3
IPR001660	Sterile alpha motif domain	277	2	0
IPR028889	Ubiquitin specific protease domain	275	3	1
IPR020568	Ribosomal protein S5 domain 2-type fold	274	3	5
IPR001251	CRAL-TRIO lipid binding domain	273	2	6
IPR002401	Cytochrome P450, E-class, group I	271	6	6
IPR029033	Histidine phosphatase superfamily	269	4	7
IPR001715	Calponin homology domain	266	1	5
IPR016135	Ubiquitin-conjugating enzyme/RWD-like	264	0	0
IPR029052	Metallo-dependent phosphatase-like	263	7	2
IPR000253	Forkhead-associated FHA domain	263	2	2

InterPro signature	InterPro description	Shared *Papaipema* genes	*Papaipema sp.4* SSGs	*Papaipema speciosissima* SSGs
IPR001394	Peptidase C19 ubiquitin carboxyl-terminal hydrolase	263	2	1
IPR008936	Rho GTPase activation protein	262	2	3
IPR008942	ENTH/VHS	261	1	1
IPR001202	WW domain	250	2	6
IPR010987	Glutathione S-transferase C-terminal-like	249	7	1
IPR004088	K Homology domain type 1	245	4	1
IPR000387	Tyrosine specific protein phosphatases domain	243	1	3
IPR020635	Tyrosine-protein kinase catalytic domain	239	2	5
IPR016181	Acyl-CoA N-acyltransferase	239	2	3
IPR029055	Nucleophile aminohydrolases N-terminal	236	1	1
IPR004843	Calcineurin-like phosphoesterase domain apaH type	235	5	1
IPR001214	SET domain	228	0	1
IPR000276	G protein-coupled receptor, rhodopsin-like	224	11	2
IPR000195	Rab-GTPase-TBC domain	223	3	3
IPR004827	Basic-leucine zipper domain	223	1	1
IPR002041	Ran GTPase	223	0	1
IPR009060	UBA-like	219	2	3
IPR009030	Growth factor receptor cysteine-rich domain	218	10	4
IPR000873	AMP-dependent synthetase/ligase	216	7	5
IPR001878	Zinc finger CCHC-type	211	36	38

InterPro signature	InterPro description	Shared *Papaipema* genes	*Papaipema sp.4* SSGs	*Papaipema speciosissima* SSGs
IPR027806	Harbinger transposase-derived nuclease domain	210	23	30
IPR001005	SANT/Myb domain	210	2	2
IPR008978	HSP20-like chaperone	210	1	1
IPR000198	Rho GTPase-activating protein domain	209	2	1
IPR003663	Sugar/inositol transporter	208	2	0
IPR011527	ABC transporter type 1 transmembrane domain	202	20	5
IPR017452	GPCR rhodopsin-like 7TM	202	2	10
IPR000014	PAS domain	202	0	1
IPR000330	SNF2-related N-terminal domain	201	1	1
IPR009000	Translation protein beta-barrel domain	198	3	11
IPR023299	P-type ATPase cytoplasmic domain N	197	2	5
IPR002219	Protein kinase C-like phorbol ester/diacylglycerol-binding domain	196	2	2
IPR016187	C-type lectin fold	195	14	6
IPR000467	G-patch domain	194	2	1
IPR000215	Serpin family	193	2	9
IPR001487	Bromodomain	193	1	3
IPR004087	K Homology domain	193	4	0
IPR008979	Galactose-binding domain-like	192	4	3
IPR001881	EGF-like calcium-binding domain	191	7	2
IPR013766	Thioredoxin domain	191	2	1

InterPro signature	InterPro description	Shared *Papaipema* genes	*Papaipema sp.4* SSGs	*Papaipema speciosissima* SSGs
IPR001683	Phox homologous domain	191	0	2
IPR000608	Ubiquitin-conjugating enzyme E2	191	0	0
IPR009072	Histone-fold	188	0	5
IPR000626	Ubiquitin domain	187	2	1
IPR000299	FERM domain	186	1	3
IPR008974	TRAF-like	182	2	1
IPR010562	Haemolymph juvenile hormone binding	181	6	5
IPR001757	P-type ATPase	181	5	4
IPR001071	Cellular retinaldehyde binding/alpha-tocopherol transport	181	3	1
IPR032466	Metal-dependent hydrolase	178	5	4
IPR017981	GPCR family 2-like	178	4	0
IPR001781	Zinc finger LIM-type	177	9	2
IPR000618	Insect cuticle protein	177	1	0
IPR006195	Aminoacyl-tRNA synthetase class II	176	0	0
IPR011511	Variant SH3 domain	175	0	0
IPR008250	P-type ATPase A domain	174	2	8
IPR019748	FERM central domain	174	2	2
IPR016161	Aldehyde/histidinol dehydrogenase	172	4	5
IPR000560	Histidine phosphatase superfamily, clade-2	172	2	6
IPR023796	Serpin domain	170	10	6

InterPro signature	InterPro description	Shared *Papaipema* genes	*Papaipema sp.4* SSGs	*Papaipema speciosissima* SSGs
IPR011047	Quinoprotein alcohol dehydrogenase-like superfamily	170	2	3
IPR011074	CRAL/TRIO N-terminal domain	169	0	1
IPR018499	Tetraspanin/Peripherin	168	2	1
IPR019749	Band 4.1 domain	168	1	2
IPR024936	Cyclophilin-type peptidyl-prolyl cis-trans isomerase	168	0	1
IPR002085	Alcohol dehydrogenase superfamily, zinc-type	167	3	1
IPR011032	GroES-like	167	1	3
IPR000961	AGC-kinase C-terminal	166	1	5
IPR013767	PAS fold	166	0	0
IPR001356	Homeobox domain	165	2	3
IPR000734	Triacylglycerol lipase family	163	2	4
IPR001279	Metallo-beta-lactamase	162	1	0
IPR000219	Dbl homology DH domain	161	0	2
IPR004046	Glutathione S-transferase C-terminal	160	4	1
IPR003595	Protein-tyrosine phosphatase catalytic	160	1	2
IPR029000	Cyclophilin-like domain	159	1	0
IPR015590	Aldehyde dehydrogenase domain	158	4	4
IPR000182	GNAT domain	158	1	3
IPR000340	Dual specificity phosphatase catalytic domain	158	1	1
IPR004210	BESS motif	157	0	6

InterPro signature	InterPro description	Shared *Papaipema* genes	*Papaipema sp.4* SSGs	*Papaipema speciosissima* SSGs
IPR002130	Cyclophilin-type peptidyl-prolyl cis-trans isomerase domain	157	1	0
IPR003877	SPRY domain	156	5	3
IPR000832	GPCR, family 2, secretin-like	156	0	2
IPR016197	Chromo domain-like	155	3	2
IPR013057	Amino acid transporter transmembrane domain	154	2	7
IPR001357	BRCT domain	154	0	1
IPR007502	Helicase-associated domain	153	0	0
IPR000301	Tetraspanin	150	2	1
IPR024156	Small GTPase superfamily, ARF type	147	1	0
IPR006612	Zinc finger C2CH-type	146	13	8
IPR008145	Guanylate kinase/L-type calcium channel beta subunit	146	1	2
IPR006020	PTB/PI domain	145	4	2
IPR000073	Alpha/beta hydrolase fold-1	145	3	0
IPR027640	Kinesin-like protein	145	0	2
IPR000717	Proteasome component PCI domain	145	0	1
IPR011012	Longin-like domain	145	0	1
IPR015919	Cadherin-like	144	9	8
IPR004045	Glutathione S-transferase N-terminal	141	3	3
IPR009100	Acyl-CoA dehydrogenase/oxidase N-terminal and middle domain	141	2	3
IPR009075	Acyl-CoA dehydrogenase/oxidase C-terminal	139	2	5

InterPro signature	InterPro description	Shared *Papaipema* genes	*Papaipema sp.4* SSGs	*Papaipema speciosissima* SSGs
IPR017850	Alkaline-phosphatase-like core domain	138	0	2
IPR002999	Tudor domain	137	0	0
IPR000242	PTP type protein phosphatase	136	1	2
IPR002557	Chitin binding domain	136	0	3
IPR001304	C-type lectin	134	12	4
IPR001395	Aldo/keto reductase/potassium channel subunit beta	134	7	5
IPR013128	Peptidase C1A	132	6	1
IPR004154	Anticodon-binding	132	0	2
IPR013525	ABC-2 type transporter	132	1	1
IPR001723	Nuclear hormone receptor	132	0	1
IPR016137	RGS domain	132	0	0
IPR001870	B30.2/SPRY domain	131	5	3
IPR018957	Zinc finger C3HC4 RING-type	131	0	1
IPR000536	Nuclear hormone receptor ligand-binding domain	130	1	0
IPR001353	Proteasome, subunit alpha/beta	130	1	0
IPR001173	Glycosyltransferase 2-like	129	1	1
IPR004012	RUN domain	129	0	2
IPR002067	Mitochondrial carrier protein	128	7	0
IPR029045	ClpP/crotonase-like domain	128	2	0
IPR013763	Cyclin-like	128	0	0

InterPro signature	InterPro description	Shared *Papaipema* genes	*Papaipema sp.4* SSGs	*Papaipema speciosissima* SSGs
IPR023210	NADP-dependent oxidoreductase domain	127	5	7
IPR000953	Chromo/chromo shadow domain	127	3	1
IPR000795	Transcription factor GTP-binding domain	126	1	6
IPR015797	NUDIX hydrolase domain-like	126	2	1
IPR000595	Cyclic nucleotide-binding domain	124	1	0
IPR001628	Zinc finger nuclear hormone receptor-type	124	0	0
IPR028002	Myb/SANT-like DNA-binding domain	123	6	5
IPR001054	Adenylyl cyclase class-3/4/guanylyl cyclase	123	5	2
IPR029787	Nucleotide cyclase	123	5	2
IPR008144	Guanylate kinase-like domain	123	1	1
IPR000086	NUDIX hydrolase domain	123	1	0
IPR018490	Cyclic nucleotide-binding-like	123	1	0
IPR002126	Cadherin	122	8	8
IPR009080	Aminoacyl-tRNA synthetase class 1a anticodon-binding	122	2	0
IPR031107	Small heat shock protein HSP20	122	1	0
IPR017946	PLC-like phosphodiesterase TIM beta/alpha-barrel domain	121	6	0
IPR015940	Ubiquitin-associated domain	121	0	2
IPR006091	Acyl-CoA oxidase/dehydrogenase central domain	120	1	3
IPR008967	p53-like transcription factor DNA-binding	120	2	0
IPR006569	CID domain	120	0	1

InterPro signature	InterPro description	Shared *Papaipema* genes	*Papaipema sp.4* SSGs	*Papaipema speciosissima* SSGs
IPR020471	Aldo/keto reductase	119	4	4
IPR011021	Arrestin-like N-terminal	119	1	3
IPR002213	UDP-glucuronosyl/UDP-glucosyltransferase	118	0	7
IPR013809	ENTH domain	118	2	0
IPR032695	Integrin domain	118	2	0
IPR008949	Isoprenoid synthase domain	117	2	2
IPR018980	FERM C-terminal PH-like domain	116	1	1
IPR008928	Six-hairpin glycosidase-like	115	0	1
IPR008952	Tetraspanin EC2 domain	114	1	1
IPR003604	Zinc finger U1-type	113	2	2
IPR016064	NAD kinase/diacylglycerol kinase-like domain	113	2	1
IPR001436	Alpha crystallin/Heat shock protein	113	1	0
IPR011029	Death-like domain	113	0	1
IPR020843	Polyketide synthase enoylreductase domain	112	1	0
IPR002159	CD36 family	111	2	0
IPR003347	JmjC domain	111	1	0
IPR006073	GTP binding domain	111	0	1
IPR011709	Domain of unknown function DUF1605	111	0	0
IPR029060	PIN domain-like	110	6	0
IPR014720	Double-stranded RNA-binding domain	110	0	3

InterPro signature	InterPro description	Shared *Papaipema* genes	*Papaipema sp.4* SSGs	*Papaipema speciosissima* SSGs
IPR023566	Peptidyl-prolyl cis-trans isomerase, FKBP-type	110	1	0
IPR001930	Peptidase M1, alanine aminopeptidase/leukotriene A4 hydrolase	110	0	0
IPR001753	Crotonase superfamily	109	0	2
IPR018979	FERM N-terminal	109	1	1
IPR013818	Lipase N-terminal	108	4	2
IPR001594	Zinc finger DHHC-type palmitoyltransferase	108	2	0
IPR017455	Zinc finger FYVE-related	108	2	0
IPR002068	Alpha crystallin/Hsp20 domain	108	0	1
IPR030564	Myotubularin family	108	1	0
IPR020103	Pseudouridine synthase catalytic domain	108	0	0
IPR007588	Zinc finger FLYWCH-type	107	19	6
IPR001752	Kinesin motor domain	107	2	1
IPR005055	Insect odorant-binding protein A10/Ejaculatory bulb-specific protein 3	107	0	2
IPR025110	AMP-binding enzyme C-terminal domain	107	2	0
IPR001199	Cytochrome b5-like heme/steroid binding domain	107	0	1
IPR021109	Aspartic peptidase domain	106	33	22
IPR008927	6-phosphogluconate dehydrogenase C-terminal domain-like	106	1	0
IPR011013	Galactose mutarotase-like domain	106	0	0
IPR013099	Potassium channel domain	105	0	4
IPR001507	Zona pellucida domain	105	1	1

InterPro signature	InterPro description	Shared *Papaipema* genes	*Papaipema sp.4* SSGs	*Papaipema speciosissima* SSGs
IPR004839	Aminotransferase class I/classII	104	1	5
IPR017892	Protein kinase C-terminal	104	1	4
IPR003029	S1 domain	104	1	0
IPR031734	Transcription activator MBF2	104	0	0
IPR029061	Thiamin diphosphate-binding fold	103	0	1
IPR008042	Retrotransposon, Pao	102	35	49
IPR006539	P-type ATPase, subfamily IV	102	1	2
IPR002049	Laminin EGF domain	102	0	2
IPR005123	Oxoglutarate/iron-dependent dioxygenase	102	1	1
IPR000159	Ras-associating RA domain	102	0	1
IPR001932	PPM-type phosphatase domain	102	1	0
IPR006903	RNA polymerase II-binding domain	102	0	1
IPR001164	Arf GTPase activating protein	102	0	0
IPR029034	Cystine-knot cytokine	102	0	0
IPR022755	Zinc finger double-stranded RNA binding	101	4	1
IPR000306	FYVE zinc finger	101	1	0
IPR023780	Chromo domain	101	0	1
IPR004299	Membrane bound O-acyl transferase, MBOAT	101	0	0
IPR009091	Regulator of chromosome condensation 1/beta-lactamase-inhibitor protein II	101	0	0
IPR000727	Target SNARE coiled-coil homology domain	100	5	1

InterPro signature	InterPro description	Shared *Papaipema* genes	*Papaipema sp.4* SSGs	*Papaipema speciosissima* SSGs
IPR000286	Histone deacetylase superfamily	100	1	3
IPR026791	Dedicator of cytokinesis	100	1	2
IPR015947	PUA-like domain	100	0	0
IPR016159	Cullin repeat-like-containing domain	100	0	0
IPR000569	HECT domain	99	2	3
IPR001619	Sec1-like protein	99	1	3
IPR002423	Chaperonin Cpn60/TCP-1 family	99	3	0
IPR018484	Carbohydrate kinase FGGY N-terminal	99	2	1
IPR000668	Peptidase C1A papain C-terminal	98	0	4
IPR023801	Histone deacetylase domain	98	3	1
IPR000555	JAB1/MPN/MOV34 metalloenzyme domain	98	0	0
IPR000644	CBS domain	97	1	1
IPR000994	Peptidase M24 structural domain	97	1	1
IPR010569	Myotubularin-like phosphatase domain	97	0	1
IPR000594	THIF-type NAD/FAD binding fold	96	1	3
IPR001902	SLC26A/SulP transporter	96	3	1
IPR017938	Riboflavin synthase-like beta-barrel	96	0	3
IPR001876	Zinc finger RanBP2-type	96	1	1
IPR007123	Gelsolin-like domain	96	0	2
IPR013154	Alcohol dehydrogenase N-terminal	96	1	1

InterPro signature	InterPro description	Shared *Papaipema* genes	*Papaipema sp.4* SSGs	*Papaipema speciosissima* SSGs
IPR007889	DNA binding HTH domain Psq-type	95	8	0
IPR029056	Ribokinase-like	95	1	2
IPR007052	CS domain	95	1	0
IPR000313	PWWP domain	95	0	0
IPR013103	Reverse transcriptase RNA-dependent DNA polymerase	94	39	21
IPR017884	SANT domain	94	1	2
IPR006077	Vinculin/alpha-catenin	94	1	0
IPR000483	Cysteine-rich flanking region C-terminal	94	0	0
IPR000859	CUB domain	93	4	3
IPR008991	Translation protein SH3-like domain	93	2	4
IPR011022	Arrestin C-terminal-like domain	93	1	5
IPR000092	Polyprenyl synthetase	93	2	2
IPR001247	Exoribonuclease phosphorolytic domain 1	93	1	0
IPR002893	Zinc finger MYND-type	93	1	0
IPR015655	Protein phosphatase 2C family	93	0	1
IPR001590	Peptidase M12B ADAM/reprolysin	93	0	0
IPR014782	Peptidase M1 membrane alanine aminopeptidase N-terminal	93	0	0
IPR011249	Metalloenzyme LuxS/M16 peptidase-like	92	1	4
IPR002083	MATH/TRAF domain	92	2	1
IPR027409	GroEL-like apical domain	92	0	1

**InterPro signature**	**InterPro description**	**Shared *Papaipema* genes**	***Papaipema sp.4* SSGs**	***Papaipema speciosissima* SSGs**
IPR011547	SLC26A/SulP transporter domain	91	1	3
IPR000648	Oxysterol-binding protein	91	2	1
IPR024950	Dual specificity phosphatase	91	0	3
IPR032710	NTF2-like domain	91	0	3
IPR001494	Importin-beta N-terminal domain	91	0	0
IPR002553	Clathrin/coatomer adaptor adaptin-like N-terminal	91	0	0
IPR006076	FAD dependent oxidoreductase	91	0	0
IPR010920	LSM domain	91	0	0
IPR029057	Phosphoribosyltransferase-like	91	0	0
IPR004014	Cation-transporting P-type ATPase N-terminal	90	1	2
IPR006153	Cation/H+ exchanger	90	2	0
IPR005112	dDENN domain	90	0	1
IPR006594	LIS1 homology motif	90	0	0
IPR001609	Myosin head motor domain	89	7	7
IPR026055	Fatty acyl-CoA reductase	89	7	4
IPR029035	DHS-like NAD/FAD-binding domain	89	3	1
IPR000326	Phosphatidic acid phosphatase type 2/haloperoxidase	89	0	1
IPR006689	Small GTPase superfamily, ARF/SAR type	89	1	0
IPR013162	CD80-like immunoglobulin C2-set	89	0	1
IPR006671	Cyclin N-terminal	89	0	0

InterPro signature	InterPro description	Shared *Papaipema* genes	*Papaipema sp.4* SSGs	*Papaipema speciosissima* SSGs
IPR013106	Immunoglobulin V-set domain	89	0	0
IPR004344	Tubulin-tyrosine ligase/Tubulin polyglutamylase	88	5	5
IPR019791	Haem peroxidase, animal	88	2	1
IPR004119	Protein of unknown function DUF227	88	1	1
IPR011038	Calycin-like	88	2	0
IPR015812	Integrin beta subunit	88	0	2
IPR001179	FKBP-type peptidyl-prolyl cis-trans isomerase domain	88	0	1
IPR013149	Alcohol dehydrogenase C-terminal	88	0	1
IPR002076	ELO family	87	1	5
IPR010255	Haem peroxidase	87	3	1
IPR006186	Serine/threonine-specific protein phosphatase/bis5-nucleosyl-tetraphosphatase	87	1	0
IPR003689	Zinc/iron permease	87	0	0
IPR004000	Actin family	87	0	0
IPR029062	Class I glutamine amidotransferase-like	87	0	0
IPR028082	Periplasmic binding protein-like I	86	2	2
IPR003656	Zinc finger BED-type	85	4	3
IPR022812	Dynamin superfamily	85	2	1
IPR011330	Glycoside hydrolase/deacetylase beta/alpha-barrel	85	0	1
IPR023088	3'5'-cyclic nucleotide phosphodiesterase	85	1	0
IPR028325	Voltage-gated potassium channel	84	8	4

InterPro signature	InterPro description	Shared *Papaipema* genes	*Papaipema sp.4* SSGs	*Papaipema speciosissima* SSGs
IPR001194	DENN domain	84	0	1
IPR017927	Ferredoxin reductase-type FAD-binding domain	84	0	1
IPR001132	SMAD domain Dwarfin-type	84	0	0
IPR020636	Calcium/calmodulin-dependent/calcium-dependent protein kinase	83	2	4
IPR004161	Translation elongation factor EFTu/EF1A domain 2	83	1	4
IPR015433	Phosphatidylinositol Kinase	83	2	3
IPR000436	Sushi/SCR/CCP domain	83	2	2
IPR001025	Bromo adjacent homology BAH domain	83	1	0
IPR002919	Trypsin Inhibitor-like cysteine rich domain	83	0	1
IPR013078	Histidine phosphatase superfamily, clade-1	83	1	0
IPR015510	Peptidoglycan recognition protein	83	0	0
IPR011705	BTB/Kelch-associated	82	5	0
IPR001828	Receptor ligand binding region	82	2	2
IPR000798	Ezrin/radixin/moesin-like	82	2	1
IPR001107	Band 7 domain	82	0	1
IPR002073	3'5'-cyclic nucleotide phosphodiesterase catalytic domain	82	0	1
IPR003131	Potassium channel tetramerisation-type BTB domain	82	1	0
IPR001763	Rhodanese-like domain	82	0	0
IPR006111	Archaeal RpoK/eukaryotic RPB6 RNA polymerase subunit	81	9	6
IPR003890	MIF4G-like type 3	81	0	0

InterPro signature	InterPro description	Shared *Papaipema* genes	*Papaipema sp.4* SSGs	*Papaipema speciosissima* SSGs
IPR000175	Sodium:neurotransmitter symporter	80	7	2
IPR000172	Glucose-methanol-choline oxidoreductase N-terminal	80	2	5
IPR006600	HTH CenpB-type DNA-binding domain	80	5	1
IPR001208	MCM domain	80	0	1
IPR016093	MIR motif	80	0	1
IPR010472	Formin FH3 domain	80	0	0
IPR020422	Dual specificity protein phosphatase domain	80	0	0
IPR011335	Restriction endonuclease type II-like	79	5	3
IPR003010	Carbon-nitrogen hydrolase	79	2	0
IPR000403	Phosphatidylinositol 3-/4-kinase catalytic domain	79	0	1
IPR001678	SAM-dependent methyltransferase RsmB/NOP2-type	79	0	0
IPR002867	IBR domain	79	0	0
IPR025799	Protein arginine N-methyltransferase	79	0	0
IPR031327	Mini-chromosome maintenance protein	79	0	0
IPR005312	Protein of unknown function DUF1759	78	22	23
IPR006604	Disulphide knot CLIP	78	1	4
IPR001206	Diacylglycerol kinase catalytic domain	78	2	1
IPR002350	Kazal domain	78	0	1
IPR006068	Cation-transporting P-type ATPase C-terminal	78	1	0
IPR014768	Rho GTPase-binding/formin homology 3 GBD/FH3 domain	78	0	1

InterPro signature	InterPro description	Shared *Papaipema* genes	*Papaipema sp.4* SSGs	*Papaipema speciosissima* SSGs
IPR015902	Glycoside hydrolase, family 13	78	1	0
IPR018485	Carbohydrate kinase FGGY C-terminal	78	0	1
IPR000413	Integrin alpha chain	78	0	0
IPR001373	Cullin N-terminal	78	0	0
IPR002314	Aminoacyl-tRNA synthetase class II G/ P/ S/T	78	0	0
IPR002293	Amino acid/polyamine transporter I	77	1	3
IPR004841	Amino acid permease/ SLC12A domain	77	0	0
IPR011417	AP180 N-terminal homology ANTH domain	76	2	0
IPR000697	WH1/EVH1 domain	76	0	1
IPR007863	Peptidase M16 C-terminal	76	0	1
IPR029064	50S ribosomal protein L30e-like	76	0	1
IPR001163	LSM domain eukaryotic/archaea-type	76	0	0
IPR003034	SAP domain	75	6	0
IPR023578	Ras guanine nucleotide exchange factor domain	75	0	1
IPR003607	HD/PDEase domain	75	0	0
IPR009038	GOLD domain	75	0	0
IPR010989	t-SNARE	74	1	1
IPR015847	Exoribonuclease phosphorolytic domain 2	74	0	1
IPR001079	Galectin carbohydrate recognition domain	74	0	0
IPR030381	Dynamin-type guanine nucleotide-binding G domain	73	1	1

InterPro signature	InterPro description	Shared *Papaipema* genes	*Papaipema sp.4* SSGs	*Papaipema speciosissima* SSGs
IPR001607	Zinc finger UBP-type	73	1	0
IPR005113	uDENN domain	73	0	1
IPR011146	HIT-like domain	73	1	0
IPR000834	Peptidase M14 carboxypeptidase A	73	0	0
IPR022700	Proteinase regulatory CLIP domain	72	1	3
IPR004162	E3 ubiquitin-protein ligase SIN-like	72	1	1
IPR000433	Zinc finger ZZ-type	72	0	1
IPR011053	Single hybrid motif	72	1	0
IPR017998	Chaperone tailless complex polypeptide 1 TCP-1)	72	1	0
IPR018121	Seven-in-absentia protein TRAF-like domain	72	0	0
IPR011765	Peptidase M16 N-terminal	71	1	1
IPR000120	Amidase	71	0	1
IPR013786	Acyl-CoA dehydrogenase/oxidase N-terminal	71	1	0
IPR029030	Caspase-like domain	71	0	1
IPR029515	LAR-interacting protein, Liprin	71	0	1
IPR023267	RNA C5-cytosine methyltransferase	71	0	0
IPR013120	Male sterility NAD-binding	70	4	6
IPR013201	Cathepsin propeptide inhibitor domain I29	70	2	2
IPR000731	Sterol-sensing domain	70	1	0
IPR006573	NEUZ domain	70	1	0

**InterPro signature**	**InterPro description**	**Shared *Papaipema* genes**	***Papaipema sp.4* SSGs**	***Papaipema speciosissima* SSGs**
IPR001375	Peptidase S9 prolyl oligopeptidase catalytic domain	70	0	0
IPR004182	GRAM domain	69	2	1
IPR015883	Glycoside hydrolase family 20 catalytic domain	69	0	3
IPR008921	DNA polymerase III clamp loader complex gamma/delta/delta subunit C-terminal	69	0	1
IPR000333	Ser/Thr protein kinase, TGFB receptor	69	0	0
IPR001067	Nuclear translocator	69	0	0
IPR002300	Aminoacyl-tRNA synthetase class Ia	69	0	0
IPR009001	Translation elongation factor EF1A/initiation factor IF2gamma C-terminal	68	3	7
IPR007632	Anoctamin	68	0	4
IPR002369	Integrin beta subunit N-terminal	68	2	0
IPR007248	Mpv17/PMP22	68	1	1
IPR017446	Polyprenyl synthetase-related	68	1	1
IPR003616	Post-SET domain	68	1	0
IPR016185	Pre-ATP-grasp domain	68	0	1
IPR000315	B-box-type zinc finger	68	0	0
IPR002909	IPT domain	68	0	0
IPR003280	Two pore domain potassium channel	68	0	0
IPR003892	Ubiquitin system component Cue	68	0	0
IPR004364	Aminoacyl-tRNA synthetase class II D/K/N	68	0	0
IPR018150	Aminoacyl-tRNA synthetase, class II D/K/N)-like	68	0	0

InterPro signature	InterPro description	Shared *Papaipema* genes	*Papaipema sp.4* SSGs	*Papaipema speciosissima* SSGs
IPR019410	Lysine methyltransferase	68	0	0
IPR013126	Heat shock protein 70 family	67	27	5
IPR001223	Glycoside hydrolase family 18 catalytic domain	67	1	3
IPR000772	Ricin B lectin domain	67	1	2
IPR000845	Nucleoside phosphorylase domain	67	0	1
IPR001433	Oxidoreductase FAD/NADP-binding	67	0	1
IPR001565	Synaptotagmin	67	0	0
IPR008969	Carboxypeptidase-like regulatory domain	67	0	0
IPR010473	Formin GTPase-binding domain	67	0	0
IPR013790	Dwarfin	67	0	0
IPR001365	Adenosine/AMP deaminase domain	66	2	0
IPR002562	3'-5' exonuclease domain	66	0	1
IPR003100	PAZ domain	66	0	1
IPR007603	Choline transporter-like	66	0	0
IPR016158	Cullin homology	66	0	0
IPR032940	Calmodulin-regulated spectrin-associated protein	66	0	0
IPR002156	Ribonuclease H domain	65	7	7
IPR000938	CAP Gly-rich domain	65	1	2
IPR007122	Villin/Gelsolin	65	2	1
IPR009053	Prefoldin	65	0	2

InterPro signature	InterPro description	Shared *Papaipema* genes	*Papaipema sp.4* SSGs	*Papaipema speciosissima* SSGs
IPR000651	Ras-like guanine nucleotide exchange factor N-terminal	65	0	1
IPR023631	Amidase signature domain	65	1	0
IPR000904	Sec7 domain	65	0	0
IPR013155	Methionyl/Valyl/Leucyl/Isoleucyl-tRNA synthetase anticodon-binding	65	0	0
IPR006202	Neurotransmitter-gated ion-channel ligand-binding domain	64	4	7
IPR017996	Major royal jelly protein/Protein yellow	64	7	0
IPR018422	Cation/H+ exchanger, CPA1 family	64	0	1
IPR000157	Toll/interleukin-1 receptor homology TIR domain	64	0	0
IPR002524	Cation efflux protein	64	0	0
IPR003118	Pointed domain	64	0	0
IPR013520	Exonuclease RNase T/DNA polymerase III	64	0	0
IPR013649	Integrin alpha-2	64	0	0
IPR005814	Aminotransferase class-III	63	1	1
IPR011761	ATP-grasp fold	63	0	2
IPR001791	Laminin G domain	63	0	1
IPR008930	Terpenoid cyclases/protein prenyltransferase alpha-alpha toroid	63	1	0
IPR010994	RuvA domain 2-like	63	0	0
IPR013655	PAS fold-3	63	0	0
IPR031775	cGMP-dependent protein kinase interacting domain	63	0	0
IPR002123	Phospholipid/glycerol acyltransferase	62	0	2

InterPro signature	InterPro description	Shared *Papaipema* genes	*Papaipema sp.4* SSGs	*Papaipema speciosissima* SSGs
IPR013657	UAA transporter	62	1	0
IPR000237	GRIP	62	0	0
IPR016193	Cytidine deaminase-like	62	0	0
IPR023332	Proteasome A-type subunit	62	0	0
IPR024571	ERAP1-like C-terminal domain	62	0	0
IPR000217	Tubulin	61	3	6
IPR001041	2Fe-2S ferredoxin-type iron-sulfur binding domain	61	4	1
IPR003594	Histidine kinase-like ATPase C-terminal domain	61	1	2
IPR006084	XPG/Rad2 endonuclease	61	0	3
IPR003653	Ulp1 protease family C-terminal catalytic domain	61	2	0
IPR015505	Coronin	61	0	2
IPR031160	F-BAR domain	61	1	1
IPR004875	DDE superfamily endonuclease domain	60	5	0
IPR003968	Potassium channel, voltage dependent, Kv	60	3	1
IPR000591	DEP domain	60	1	2
IPR001563	Peptidase S10, serine carboxypeptidase	60	1	2
IPR001309	Peptidase C14 p20 domain	60	0	1
IPR002502	N-acetylmuramoyl-L-alanine amidase domain	60	0	1
IPR006201	Neurotransmitter-gated ion-channel	59	12	5
IPR006170	Pheromone/general odorant binding protein	59	3	6

**InterPro signature**	**InterPro description**	**Shared *Papaipema* genes**	***Papaipema sp.4* SSGs**	***Papaipema speciosissima* SSGs**
IPR015925	Ryanodine receptor-related	59	3	2
IPR009022	Elongation factor G III-V domain	59	0	3
IPR016039	Thiolase-like	59	2	1
IPR016166	FAD-binding type 2	59	2	1
IPR000566	Lipocalin/cytosolic fatty-acid binding domain	59	2	0
IPR001509	NAD-dependent epimerase/dehydratase N-terminal domain	59	1	0
IPR018163	Threonyl/alanyl tRNA synthetase class II-like putative editing domain	59	0	1
IPR000331	Rap GTPase activating protein domain	59	0	0
IPR004821	Cytidyltransferase-like domain	59	0	0
IPR006026	Peptidase metallopeptidase	59	0	0
IPR006619	Peptidoglycan recognition protein family domain metazoa/bacteria	59	0	0
IPR013151	Immunoglobulin	58	2	2
IPR002483	PWI domain	58	1	2
IPR003000	Sirtuin family	58	0	3
IPR001374	R3H domain	58	1	0
IPR002469	Dipeptidylpeptidase IV N-terminal domain	58	0	0
IPR017896	4Fe-4S ferredoxin-type iron-sulphur binding domain	58	0	0
IPR023333	Proteasome B-type subunit	58	0	0
IPR029526	PiggyBac transposable element-derived protein	57	13	5
IPR021896	Transposase protein	57	6	4

InterPro signature	InterPro description	Shared *Papaipema* genes	*Papaipema sp.4* SSGs	*Papaipema speciosissima* SSGs
IPR023271	Aquaporin-like	57	2	2
IPR020454	Diacylglycerol/phorbol-ester binding	57	1	1
IPR000192	Aminotransferase class V domain	57	0	1
IPR001060	FCH domain	57	1	0
IPR007867	Glucose-methanol-choline oxidoreductase C-terminal	57	1	0
IPR023346	Lysozyme-like domain	57	1	0
IPR003613	U box domain	57	0	0
IPR005599	GPI mannosyltransferase	57	0	0
IPR008422	Homeobox KN domain	57	0	0
IPR013010	Zinc finger SIAH-type	57	0	0
IPR015897	CHK kinase-like	57	0	0
IPR022613	Calmodulin-regulated spectrin-associated protein CH domain	57	0	0
IPR022775	AP complex mu/sigma subunit	57	0	0
IPR002937	Amine oxidase	56	1	2
IPR027450	Alpha-ketoglutarate-dependent dioxygenase AlkB-like	56	0	2
IPR004156	Organic anion transporter polypeptide OATP	56	1	0
IPR030217	Nuclear RNA export factor	56	0	1
IPR000585	Hemopexin-like domain	56	0	0
IPR000756	Diacylglycerol kinase accessory domain	56	0	0
IPR001895	Ras guanine-nucleotide exchange factors catalytic domain	56	0	0

InterPro signature	InterPro description	Shared *Papaipema* genes	*Papaipema sp.4* SSGs	*Papaipema speciosissima* SSGs
IPR003123	VPS9 domain	56	0	0
IPR006896	Sec23/Sec24 trunk domain	56	0	0
IPR011051	RmlC-like cupin domain	56	0	0
IPR032104	Spaetzle	56	0	0
IPR008737	Peptidase aspartic putative	55	24	18
IPR002129	Pyridoxal phosphate-dependent decarboxylase	55	4	0
IPR000462	CDP-alcohol phosphatidyltransferase	55	1	1
IPR000089	Biotin/lipoyl attachment	55	1	0
IPR000602	Glycoside hydrolase family 38 N-terminal domain	55	0	1
IPR001627	Sema domain	55	1	0
IPR006047	Glycosyl hydrolase family 13 catalytic domain	55	0	1
IPR008734	Phosphorylase kinase alpha/beta subunit	55	0	1
IPR003954	RNA recognition motif domain eukaryote	55	0	0
IPR004365	OB-fold nucleic acid binding domain AA-tRNA synthetase-type	55	0	0
IPR004709	Na+/H+ exchanger	55	0	0
IPR006214	Bax inhibitor 1-related	55	0	0
IPR006895	Zinc finger Sec23/Sec24-type	55	0	0
IPR011583	Chitinase II	55	0	0
IPR013632	DNA recombination and repair protein Rad51 C-terminal	55	0	0
IPR015720	TMP21-related	55	0	0

InterPro signature	InterPro description	Shared *Papaipema* genes	*Papaipema sp.4* SSGs	*Papaipema speciosissima* SSGs
IPR018222	Nuclear transport factor 2 eukaryote	55	0	0
IPR020588	DNA recombination and repair protein RecA-like ATP-binding domain	55	0	0
IPR006029	Neurotransmitter-gated ion-channel transmembrane domain	54	2	8
IPR024084	Isopropylmalate dehydrogenase-like domain	54	0	4
IPR024134	Superoxide dismutase Cu/Zn / superoxide dismutase copper chaperone	54	1	1
IPR006680	Amidohydrolase-related	54	0	1
IPR012990	Sec23/Sec24 beta-sandwich	54	0	1
IPR000917	Sulfatase N-terminal	54	0	0
IPR002645	STAS domain	54	0	0
IPR003650	Orange domain	54	0	0
IPR006575	RWD domain	54	0	0
IPR022967	RNA-binding domain S1	54	0	0
IPR005522	Inositol polyphosphate kinase	53	1	3
IPR000494	Receptor L-domain	53	0	3
IPR026590	Sirtuin family catalytic core domain	53	3	0
IPR028846	Recoverin family	53	0	3
IPR001388	Synaptobrevin	53	0	2
IPR005024	Snf7 family	53	1	1
IPR000322	Glycoside hydrolase family 31	53	0	1
IPR001320	Ionotropic glutamate receptor	53	1	0

InterPro signature	InterPro description	Shared *Papaipema* genes	*Papaipema sp.4* SSGs	*Papaipema speciosissima* SSGs
IPR001709	Flavoprotein pyridine nucleotide cytochrome reductase	53	0	1
IPR002931	Transglutaminase-like	53	0	1
IPR009886	HCaRG	53	1	0
IPR032631	P-type ATPase N-terminal	53	1	0
IPR000418	Ets domain	53	0	0
IPR000999	Ribonuclease III domain	53	0	0
IPR002939	Chaperone DnaJ C-terminal	53	0	0
IPR003958	Transcription factor CBF/NF-Y/archaeal histone domain	53	0	0
IPR008971	HSP40/DnaJ peptide-binding	53	0	0
IPR024964	CTLH/CRA C-terminal to LisH motif domain	53	0	0
IPR030395	Glycerophosphodiester phosphodiesterase domain	53	0	0
IPR003084	Histone deacetylase	52	1	3
IPR004148	BAR domain	52	2	0
IPR025705	Beta-hexosaminidase	52	2	0
IPR000640	Translation elongation factor EFG V domain	52	0	1
IPR001496	SOCS box domain	52	1	0
IPR000426	Proteasome alpha-subunit N-terminal domain	52	0	0
IPR002870	Peptidase M12B propeptide	52	0	0
IPR006589	Glycosyl hydrolase family 13 subfamily catalytic domain	52	0	0
IPR006900	Sec23/Sec24 helical domain	52	0	0

InterPro signature	InterPro description	Shared *Papaipema* genes	*Papaipema sp.4* SSGs	*Papaipema speciosissima* SSGs
IPR012132	Glucose-methanol-choline oxidoreductase	52	0	0
IPR003008	Tubulin/FtsZ GTPase domain	51	6	3
IPR027244	Vacuolar membrane-associated protein Iml1	51	3	3
IPR031986	Serine protease gd N-terminal domain	51	4	1
IPR001548	Peptidase M2, peptidyl-dipeptidase A	51	1	3
IPR001424	Superoxide dismutase copper/zinc binding domain	51	2	1
IPR001762	Disintegrin domain	51	1	0
IPR003392	Patched	51	0	1
IPR004031	PMP-22/EMP/MP20/Claudin superfamily	51	1	0
IPR014836	Integrin beta subunit cytoplasmic domain	51	1	0
IPR001012	UBX domain	51	0	0
IPR001382	Glycoside hydrolase family 47	51	0	0
IPR001739	Methyl-CpG DNA binding	51	0	0
IPR001818	Peptidase M10 metallopeptidase	51	0	0
IPR002877	Ribosomal RNA methyltransferase FtsJ domain	51	0	0
IPR002913	START domain	51	0	0
IPR003114	Phox-associated domain	51	0	0
IPR004274	FCP1 homology domain	51	0	0
IPR005101	Cryptochrome/DNA photolyase FAD-binding domain	51	0	0
IPR009008	Valyl/Leucyl/Isoleucyl-tRNA synthetase editing domain	51	0	0

InterPro signature	InterPro description	Shared *Papaipema* genes	*Papaipema sp.4* SSGs	*Papaipema speciosissima* SSGs
IPR009145	U2 auxiliary factor small subunit	51	0	0
IPR013968	Polyketide synthase ketoreductase domain	51	0	0
IPR016177	DNA-binding domain	51	0	0
IPR016491	Septin	51	0	0
IPR021190	Peptidase M10A	51	0	0
IPR025714	Methyltransferase domain	51	0	0
IPR028565	Mu homology domain	51	0	0
IPR007125	Histone H2A/H2B/H3	50	0	4
IPR015425	Formin FH2 domain	50	2	1
IPR011050	Pectin lyase fold/virulence factor	50	2	0
IPR031127	E3 ubiquitin ligase RBR family	50	2	0
IPR001766	Fork head domain	50	0	1
IPR002942	RNA-binding S4 domain	50	0	1
IPR011613	Glycoside hydrolase family 15/Phosphorylase b kinase regulatory chain family	50	0	1
IPR015500	Peptidase S8, subtilisin-related	50	0	1
IPR029059	Alpha/beta hydrolase fold-5	50	0	1
IPR000488	Death domain	50	0	0
IPR027005	Glycosyltransferase 39-like	50	0	0
IPR027705	Flotillin family	50	0	0
IPR006028	Gamma-aminobutyric acid A receptor/Glycine receptor alpha	49	6	2

**InterPro signature**	**InterPro description**	**Shared *Papaipema* genes**	***Papaipema sp.4* SSGs**	***Papaipema speciosissima* SSGs**
IPR027791	Galactosyltransferase C-terminal	49	2	1
IPR000061	SWAP/Surp	49	1	1
IPR007109	Brix domain	49	0	2
IPR009068	S15/NS1 RNA-binding	49	0	2
IPR015048	Domain of unknown function DUF1899	49	2	0
IPR003307	W2 domain	49	1	0
IPR011054	Rudiment single hybrid motif	49	0	1
IPR029070	Chitinase insertion domain	49	0	1
IPR001007	VWFC domain	49	0	0
IPR002014	VHS domain	49	0	0
IPR002125	Cytidine and deoxycytidylate deaminases zinc-binding	49	0	0
IPR002478	PUA domain	49	0	0
IPR003124	WH2 domain	49	0	0
IPR005018	DOMON domain	49	0	0
IPR005937	26S proteasome subunit P45	49	0	0
IPR008598	Drought induced 19 protein type zinc-binding domain	49	0	0
IPR017930	Myb domain	49	0	0
IPR028980	Creatinase/Aminopeptidase P N-terminal	49	0	0
IPR005062	SAC3/GANP/THP3	48	0	4
IPR000718	Peptidase M13	48	1	2

**InterPro signature**	**InterPro description**	**Shared *Papaipema* genes**	***Papaipema sp.4* SSGs**	***Papaipema speciosissima* SSGs**
IPR001217	Transcription factor STAT	48	0	1
IPR001310	Histidine triad HIT protein	48	0	1
IPR002495	Glycosyl transferase, family 8	48	0	1
IPR004038	Ribosomal protein L7Ae/L30e/S12e/Gadd45	48	0	1
IPR009465	Spondin N-terminal	48	0	1
IPR014013	Helicase superfamily 1/2 ATP-binding domain DinG/Rad3-type	48	0	1
IPR032630	P-type ATPase C-terminal	48	1	0
IPR001017	Dehydrogenase E1 component	48	0	0
IPR003126	Zinc finger UBR-type	48	0	0
IPR003649	B-box C-terminal	48	0	0
IPR006222	Glycine cleavage T-protein-like N-terminal	48	0	0
IPR006595	CTLH C-terminal LisH motif	48	0	0
IPR007259	Gamma-tubulin complex component protein	48	0	0
IPR011539	Rel homology domain RHD DNA-binding domain	48	0	0
IPR012947	Threonyl/alanyl tRNA synthetase SAD	48	0	0
IPR013041	Coatomer/clathrin adaptor appendage Ig-like subdomain	48	0	0
IPR017877	Myb-like domain	48	0	0
IPR017932	Glutamine amidotransferase type 2 domain	48	0	0
IPR023561	Carbonic anhydrase, alpha-class	48	0	0
IPR001503	Glycosyl transferase family 10	47	1	1

InterPro signature	InterPro description	Shared *Papaipema* genes	*Papaipema sp.4* SSGs	*Papaipema speciosissima* SSGs
IPR000209	Peptidase S8/S53 domain	47	1	0
IPR001879	GPCR family 2 extracellular hormone receptor domain	47	0	1
IPR001916	Glycoside hydrolase, family 22	47	0	1
IPR000812	Transcription factor TFIIB	47	0	0
IPR002659	Glycosyl transferase, family 31	47	0	0
IPR002716	PIN domain	47	0	0
IPR007197	Radical SAM	47	0	0
IPR013019	MAD homology MH1	47	0	0
IPR025724	GAG-pre-integrase domain	46	15	5
IPR026082	ABC transporter A, ABCA	46	3	3
IPR003619	MAD homology 1 Dwarfin-type	46	3	2
IPR031167	OBG-type guanine nucleotide-binding G domain	46	0	2
IPR000683	Oxidoreductase N-terminal	46	1	0
IPR005475	Transketolase-like pyrimidine-binding domain	46	0	1
IPR006094	FAD linked oxidase N-terminal	46	0	1
IPR006439	HAD hydrolase, subfamily IA	46	0	1
IPR017351	LIM and senescent cell antigen-like-containing domain protein 1	46	0	1
IPR027231	Semaphorin	46	0	1
IPR003605	GS domain	46	0	0
IPR005033	YEATS	46	0	0

InterPro signature	InterPro description	Shared *Papaipema* genes	*Papaipema sp.4* SSGs	*Papaipema speciosissima* SSGs
IPR010614	DEAD2	46	0	0
IPR012338	Beta-lactamase/transpeptidase-like	46	0	0
IPR015868	Glutaminase	46	0	0
IPR021852	Domain of unknown function DUF3456	46	0	0
IPR026094	G protein pathway suppressor 2	46	0	0
IPR028995	Glycoside hydrolase families 57/38 central domain	46	0	0
IPR030442	Liprin-alpha	46	0	0
IPR031315	LNS2/PITP	46	0	0
IPR001192	Phosphoinositide phospholipase C family	45	0	8
IPR016035	Acyl transferase/acyl hydrolase/lysophospholipase	45	0	4
IPR005331	Sulfotransferase	45	0	3
IPR001734	Sodium/solute symporter	45	1	1
IPR003165	Piwi domain	45	0	2
IPR003859	Beta-1,4-galactosyltransferase	45	0	2
IPR005607	BSD domain	45	1	1
IPR002013	SAC domain	45	1	0
IPR017984	Chromo domain subgroup	45	0	1
IPR000535	Major sperm protein MSP domain	45	0	0
IPR000837	AP-1 transcription factor	45	0	0
IPR005824	KOW	45	0	0

**InterPro signature**	**InterPro description**	**Shared *Papaipema* genes**	***Papaipema sp.4* SSGs**	***Papaipema speciosissima* SSGs**
IPR008962	PapD-like	45	0	0
IPR013216	Methyltransferase type 11	45	0	0
IPR015341	Glycoside hydrolase family 38 central domain	45	0	0
IPR015712	DNA-directed RNA polymerase, subunit 2	45	0	0
IPR027995	Galactosyltransferase N-terminal	45	0	0
IPR030379	Septin-type guanine nucleotide-binding G domain	45	0	0
IPR000909	Phosphatidylinositol-specific phospholipase C X domain	44	4	0
IPR031847	Domain of unknown function DUF4749	44	2	2
IPR006643	ZASP	44	1	2
IPR018491	SLC12A transporter C-terminal	44	1	2
IPR000362	Fumarate lyase family	44	1	1
IPR000850	Adenylate kinase/UMP-CMP kinase	44	1	1
IPR008948	L-Aspartase-like	44	1	1
IPR015917	Peptidase C14A homology domain	44	0	1
IPR020067	Frizzled domain	44	1	0
IPR027925	MCM N-terminal domain	44	0	1
IPR031446	Pericentriolar material 1 protein C-terminal	44	1	0
IPR000679	Zinc finger GATA-type	44	0	0
IPR002477	Peptidoglycan binding-like	44	0	0
IPR006115	6-phosphogluconate dehydrogenase NADP-binding	44	0	0

InterPro signature	InterPro description	Shared *Papaipema* genes	*Papaipema sp.4* SSGs	*Papaipema speciosissima* SSGs
IPR011059	Metal-dependent hydrolase composite domain	44	0	0
IPR011682	Glycosyl hydrolase family 38 C-terminal	44	0	0
IPR015413	Methionyl/Leucyl tRNA synthetase	44	0	0
IPR032397	Rel homology dimerisation domain	44	0	0
IPR004160	Translation elongation factor EFTu/EF1A C-terminal	43	3	7
IPR024940	Transcription factor TCF/LEF	43	3	1
IPR003097	FAD-binding type 1	43	0	3
IPR006085	XPG N-terminal	43	3	0
IPR016449	Potassium channel, inwardly rectifying, Kir	43	0	3
IPR011611	Carbohydrate kinase PfkB	43	0	2
IPR031961	Domain of unknown function DUF4780	43	2	0
IPR002058	PAP/25A-associated	43	1	0
IPR003033	SCP2 sterol-binding domain	43	0	1
IPR003094	Fructose-2,6-bisphosphatase	43	1	0
IPR005479	Carbamoyl-phosphate synthetase large subunit-like ATP-binding domain	43	0	1
IPR005746	Thioredoxin	43	1	0
IPR000649	Initiation factor 2B-related	43	0	0
IPR002312	Aspartyl/Asparaginyl-tRNA synthetase, class IIb	43	0	0
IPR006667	SLC41 divalent cation transporters integral membrane domain	43	0	0
IPR007185	DNA polymerase alpha/epsilon subunit B	43	0	0

InterPro signature	InterPro description	Shared *Papaipema* genes	*Papaipema sp.4* SSGs	*Papaipema speciosissima* SSGs
IPR007330	MIT	43	0	0
IPR007529	Zinc finger HIT-type	43	0	0
IPR013143	PCI/PINT associated module	43	0	0
IPR014876	DEK C-terminal	43	0	0
IPR019147	Suppressor of white apricot N-terminal domain	43	0	0
IPR024096	NO signalling/Golgi transport ligand-binding domain	43	0	0
IPR032425	Talin N-terminal F0 domain	43	0	0
IPR001736	Phospholipase D/Transphosphatidylase	42	2	1
IPR006941	Ribonuclease CAF1	42	0	3
IPR008753	Peptidase M13 N-terminal domain	42	2	1
IPR000472	Activin types I and II receptor domain	42	1	0
IPR002641	Patatin/Phospholipase A2-related	42	0	1
IPR005037	Pre-mRNA-splicing factor 38	42	1	0
IPR006140	D-isomer specific 2-hydroxyacid dehydrogenase NAD-binding domain	42	0	1
IPR022761	Fumarate lyase N-terminal	42	1	0
IPR024138	Pericentriolar material 1 protein	42	0	1
IPR029018	Chitobiase/beta-hexosaminidase domain 2-like	42	0	1
IPR029019	Beta-hexosaminidase eukaryotic type N-terminal	42	0	1
IPR029069	HotDog domain	42	0	1
IPR000246	Peptidase T2, asparaginase 2	42	0	0

**InterPro signature**	**InterPro description**	**Shared *Papaipema* genes**	***Papaipema sp.4* SSGs**	***Papaipema speciosissima* SSGs**
IPR001148	Alpha carbonic anhydrase	42	0	0
IPR001156	Transferrin-like domain	42	0	0
IPR005301	MOB kinase activator family	42	0	0
IPR006204	GHMP kinase N-terminal domain	42	0	0
IPR006554	Helicase-like DEXD box c2 type	42	0	0
IPR006677	tRNA intron endonuclease catalytic domain-like	42	0	0
IPR007696	DNA mismatch repair protein MutS core	42	0	0
IPR008152	Clathrin adaptor alpha/beta/gamma-adaptin appendage Ig-like subdomain	42	0	0
IPR008758	Peptidase S28	42	0	0
IPR010291	Ion channel regulatory protein, UNC-93	42	0	0
IPR010526	Sodium ion transport-associated	42	0	0
IPR019018	Rab-binding domain FIP-RBD	42	0	0
IPR000542	Acyltransferase ChoActase/COT/CPT	41	4	2
IPR000421	Coagulation factor 5/8 C-terminal domain	41	3	1
IPR013111	EGF-like domain extracellular	41	2	2
IPR001804	Isocitrate and isopropylmalate dehydrogenases family	41	3	0
IPR002861	Reeler domain	41	0	3
IPR001401	Dynamin GTPase domain	41	1	1
IPR001464	Annexin	41	0	2
IPR002317	Serine-tRNA ligase, type1	41	2	0

**InterPro signature**	**InterPro description**	**Shared *Papaipema* genes**	***Papaipema sp.4* SSGs**	***Papaipema speciosissima* SSGs**
IPR006211	Furin-like cysteine-rich domain	41	0	2
IPR013209	Lipin/Ned1/Smp2 LNS2	41	2	0
IPR020616	Thiolase N-terminal	41	1	1
IPR001315	CARD domain	41	0	1
IPR019525	Nuclear respiratory factor 1 NLS/DNA-binding dimerisation domain	41	1	0
IPR022782	Actin interacting protein 3 C-terminal	41	0	1
IPR003323	OTU domain	41	0	0
IPR006638	Elongator protein 3/MiaB/NifB	41	0	0
IPR014764	Defective-in-cullin neddylation protein	41	0	0
IPR015894	Guanylate-binding protein N-terminal	41	0	0
IPR015898	G-protein gamma-like domain	41	0	0
IPR016055	Alpha-D-phosphohexomutase alpha/beta/alpha I/II/III	41	0	0
IPR022728	Period circadian-like C-terminal	41	0	0
IPR002616	tRNA-guanine15 transglycosylase-like	40	1	1
IPR002713	FF domain	40	0	2
IPR005552	Scramblase	40	1	1
IPR000672	Tetrahydrofolate dehydrogenase/cyclohydrolase	40	1	0
IPR004170	WWE domain	40	0	1
IPR006179	5'-Nucleotidase/apyrase	40	1	0
IPR013079	6-phosphofructo-2-kinase	40	0	1

InterPro signature	InterPro description	Shared *Papaipema* genes	*Papaipema sp.4* SSGs	*Papaipema speciosissima* SSGs
IPR020631	Tetrahydrofolate dehydrogenase/cyclohydrolase NADP-binding domain	40	0	1
IPR000300	Inositol polyphosphate-related phosphatase	40	0	0
IPR000409	BEACH domain	40	0	0
IPR001190	SRCR domain	40	0	0
IPR003395	RecF/RecN/SMC N-terminal	40	0	0
IPR006050	DNA photolyase N-terminal	40	0	0
IPR006838	FAR-17a/AIG1-like protein	40	0	0
IPR010678	Digestive organ expansion factor, predicted	40	0	0
IPR013598	Exportin-1/Importin-beta-like	40	0	0
IPR013784	Carbohydrate-binding-like fold	40	0	0
IPR018497	Peptidase M13 C-terminal domain	40	0	0
IPR019594	Ionotropic glutamate receptor L-glutamate and glycine-binding domain	40	0	0
IPR020630	Tetrahydrofolate dehydrogenase/cyclohydrolase catalytic domain	40	0	0
IPR022247	Casein kinase 1 gamma C-terminal	40	0	0
IPR025313	Domain of unknown function DUF4217	40	0	0
IPR007527	Zinc finger SWIM-type	39	6	1
IPR000615	Bestrophin	39	0	3
IPR021134	Bestrophin/UPF0187	39	0	3
IPR002655	Acyl-CoA oxidase C-terminal	39	1	1
IPR006139	D-isomer specific 2-hydroxyacid dehydrogenase catalytic domain	39	0	2

InterPro signature	InterPro description	Shared *Papaipema* genes	*Papaipema sp.4* SSGs	*Papaipema speciosissima* SSGs
IPR000699	RIH RyR and IP3R Homology domain	39	0	1
IPR001578	Peptidase C12 ubiquitin carboxyl-terminal hydrolase	39	1	0
IPR002610	Peptidase S54, rhomboid	39	1	0
IPR010935	SMCs flexible hinge	39	0	1
IPR000687	RIO kinase	39	0	0
IPR000738	WHEP-TRS domain	39	0	0
IPR001269	tRNA-dihydrouridine synthase	39	0	0
IPR002100	Transcription factor MADS-box	39	0	0
IPR002138	Peptidase C14 caspase non-catalytic subunit p10	39	0	0
IPR002591	Type I phosphodiesterase/nucleotide pyrophosphatase/phosphate transferase	39	0	0
IPR004826	Basic leucine zipper domain Maf-type	39	0	0
IPR005844	Alpha-D-phosphohexomutase alpha/beta/alpha domain I	39	0	0
IPR006544	P-type ATPase, subfamily V	39	0	0
IPR007484	Peptidase M28	39	0	0
IPR007757	MT-A70-like	39	0	0
IPR008251	Chromo shadow domain	39	0	0
IPR008917	Transcription factor Skn-1-like DNA-binding domain	39	0	0
IPR013750	GHMP kinase C-terminal domain	39	0	0
IPR013801	STAT transcription factor DNA-binding	39	0	0
IPR014371	Sterol O-acyltransferase, ACAT/DAG/ARE types	39	0	0

InterPro signature	InterPro description	Shared *Papaipema* genes	*Papaipema sp.4* SSGs	*Papaipema speciosissima* SSGs
IPR014758	Methionyl-tRNA synthetase	39	0	0
IPR032474	Protein argonaute N-terminal	39	0	0
IPR000863	Sulfotransferase domain	38	7	2
IPR001404	Heat shock protein Hsp90 family	38	4	1
IPR027272	Piezo family	38	0	3
IPR001714	Peptidase M24, methionine aminopeptidase	38	0	1
IPR003057	Invertebrate colouration protein	38	0	1
IPR006086	XPG-I domain	38	1	0
IPR022041	Farnesoic acid O-methyl transferase	38	1	0
IPR030224	Sla2 family	38	0	1
IPR000760	Inositol monophosphatase	38	0	0
IPR000857	MyTH4 domain	38	0	0
IPR001222	Zinc finger TFIIS-type	38	0	0
IPR001834	NADH:cytochrome b5 reductase CBR)	38	0	0
IPR002108	Actin-depolymerising factor homology domain	38	0	0
IPR002928	Myosin tail	38	0	0
IPR003397	Mitochondrial inner membrane translocase subunit Tim17/Tim22/Tim23/peroxisomal protein PMP24	38	0	0
IPR005045	CDC50/LEM3 family	38	0	0
IPR006933	HAP1 N-terminal	38	0	0
IPR007120	DNA-directed RNA polymerase subunit 2 domain 6	38	0	0

InterPro signature	InterPro description	Shared *Papaipema* genes	*Papaipema sp.4* SSGs	*Papaipema speciosissima* SSGs
IPR007728	Pre-SET domain	38	0	0
IPR011764	Biotin carboxylation domain	38	0	0
IPR016194	SPOC-like C-terminal domain	38	0	0
IPR027007	DHR-1 domain	38	0	0
IPR031905	Flotillin C-terminal domain	38	0	0
IPR008280	Tubulin/FtsZ C-terminal	37	5	1
IPR000425	Major intrinsic protein	37	2	3
IPR001180	Citron homology CNH domain	37	1	1
IPR001936	Ras GTPase-activating protein	37	0	2
IPR004127	Prefoldin alpha-like	37	0	2
IPR013694	VIT domain	37	2	0
IPR024729	Ubiquitin carboxyl-terminal hydrolase 7 ICP0-binding domain	37	2	0
IPR000639	Epoxide hydrolase-like	37	0	1
IPR001300	Peptidase C2 calpain catalytic domain	37	1	0
IPR002653	Zinc finger A20-type	37	1	0
IPR005804	Fatty acid desaturase domain	37	1	0
IPR009061	Putative DNA-binding domain	37	1	0
IPR010908	Longin domain	37	0	1
IPR014472	Choline/ethanolamine phosphotransferase	37	0	1
IPR000034	Laminin IV	37	0	0

InterPro signature	InterPro description	Shared *Papaipema* genes	*Papaipema sp.4* SSGs	*Papaipema speciosissima* SSGs
IPR000990	Innexin	37	0	0
IPR001605	Pleckstrin homology domain spectrin-type	37	0	0
IPR001737	Ribosomal RNA adenine methyltransferase KsgA/Erm	37	0	0
IPR002305	Aminoacyl-tRNA synthetase, class Ic	37	0	0
IPR002343	Paraneoplastic encephalomyelitis antigen	37	0	0
IPR004130	GPN-loop GTPase	37	0	0
IPR006555	ATP-dependent helicase C-terminal	37	0	0
IPR006608	Domain of unknown function DM14	37	0	0
IPR008333	Oxidoreductase FAD-binding domain	37	0	0
IPR008914	Phosphatidylethanolamine-binding protein PEBP	37	0	0
IPR014020	Tensin phosphatase C2 domain	37	0	0
IPR014847	FERM adjacent FA	37	0	0
IPR016293	Peptidase M10A, stromelysin-type	37	0	0
IPR017926	Glutamine amidotransferase	37	0	0
IPR018205	VHS subgroup	37	0	0
IPR018501	DDT domain superfamily	37	0	0
IPR020045	5'-3' exonuclease C-terminal domain	37	0	0
IPR024583	Voltage-gated Na+ ion channel cytoplasmic domain	37	0	0
IPR025659	Tubby C-terminal-like domain	37	0	0
IPR028375	KA1 domain/Ssp2 C-terminal	37	0	0

InterPro signature	InterPro description	Shared *Papaipema* genes	*Papaipema sp.4* SSGs	*Papaipema speciosissima* SSGs
IPR029028	Alpha/beta knot methyltransferases	37	0	0
IPR013788	Hemocyanin/hexamerin	36	5	2
IPR018316	Tubulin/FtsZ 2-layer sandwich domain	36	5	1
IPR032394	Anoctamin dimerisation domain	36	4	0
IPR024989	Major facilitator superfamily associated domain	36	3	0
IPR016102	Succinyl-CoA synthetase-like	36	0	2
IPR027652	Pre-mRNA-processing-splicing factor 8	36	0	2
IPR032063	Protein of unknown function DUF4804	36	0	2
IPR000082	SEA domain	36	0	1
IPR001606	ARID DNA-binding domain	36	1	0
IPR004934	Tropomodulin	36	0	1
IPR006620	Prolyl 4-hydroxylase alpha subunit	36	1	0
IPR019394	Testis-specific protein TEX28/transmembrane and coiled-coil domains protein	36	1	0
IPR020617	Thiolase C-terminal	36	1	0
IPR022684	Peptidase C2, calpain family	36	0	1
IPR030137	Tropomodulin, invertebrate	36	0	1
IPR000412	ABC-2 transporter	36	0	0
IPR000664	Lethal2 giant larvae protein	36	0	0
IPR000682	Protein-L-isoaspartateD-aspartate O-methyltransferase	36	0	0
IPR001303	Class II aldolase/adducin N-terminal	36	0	0

**InterPro signature**	**InterPro description**	**Shared *Papaipema* genes**	***Papaipema sp.4* SSGs**	***Papaipema speciosissima* SSGs**
IPR001508	Ionotropic glutamate receptor, metazoa	36	0	0
IPR001846	von Willebrand factor type D domain	36	0	0
IPR002687	Nop domain	36	0	0
IPR004516	Histidine-tRNA ligase/ATP phosphoribosyltransferase regulatory subunit	36	0	0
IPR004882	Luc7-related	36	0	0
IPR013577	Lethal giant larvae homologue 2	36	0	0
IPR013979	Translation initiation factor beta propellor-like domain	36	0	0
IPR016156	FAD/NAD-linked reductase dimerisation domain	36	0	0
IPR019406	Zinc finger C2H2 APLF-like	36	0	0
IPR020946	Flavin monooxygenase-like	36	0	0
IPR022712	Beta-Casp domain	36	0	0
IPR031314	Deoxynucleoside kinase domain	36	0	0
IPR004835	Chitin synthase	35	4	1
IPR000533	Tropomyosin	35	0	4
IPR001130	TatD family	35	2	1
IPR028427	Peptide methionine sulfoxide reductase	35	2	1
IPR028782	Protein pangolin	35	2	1
IPR011016	Zinc finger RING-CH-type	35	1	1
IPR000095	CRIB domain	35	0	1
IPR001972	Stomatin family	35	1	0

InterPro signature	InterPro description	Shared *Papaipema* genes	*Papaipema sp.4* SSGs	*Papaipema speciosissima* SSGs
IPR003137	PA domain	35	1	0
IPR005811	ATP-citrate lyase/succinyl-CoA ligase	35	0	1
IPR006329	AMP deaminase	35	0	1
IPR018017	Nucleoside phosphorylase	35	1	0
IPR028364	Ribosomal protein L1/ribosomal biogenesis protein	35	1	0
IPR000757	Glycoside hydrolase family 16	35	0	0
IPR003175	Cyclin-dependent kinase inhibitor	35	0	0
IPR003316	E2F/DP family winged-helix DNA-binding domain	35	0	0
IPR004263	Exostosin-like	35	0	0
IPR006133	DNA-directed DNA polymerase family B exonuclease domain	35	0	0
IPR006149	EB domain	35	0	0
IPR006636	Heat shock chaperonin-binding	35	0	0
IPR006875	Sarcoglycan complex subunit protein	35	0	0
IPR011704	ATPase dynein-related AAA domain	35	0	0
IPR016164	FAD-linked oxidase-like C-terminal	35	0	0
IPR017448	SRCR-like domain	35	0	0
IPR021151	GINS complex	35	0	0
IPR024969	Rpn11/EIF3F C-terminal	35	0	0
IPR028570	Triple functional domain protein	35	0	0
IPR031703	Lipin middle domain	35	0	0

InterPro signature	InterPro description	Shared *Papaipema* genes	*Papaipema sp.4* SSGs	*Papaipema speciosissima* SSGs
IPR001360	Glycoside hydrolase family 1	34	2	6
IPR000949	ELM2 domain	34	1	2
IPR002490	V-type ATPase, V0 complex, 116kDa subunit family	34	0	3
IPR000194	ATPase F1/V1/A1 complex alpha/beta subunit nucleotide-binding domain	34	1	1
IPR000232	Heat shock factor HSF-type DNA-binding	34	1	1
IPR015937	Aconitase/isopropylmalate dehydratase	34	1	1
IPR027072	Heat shock factor protein 1	34	1	1
IPR027725	Heat shock transcription factor family	34	1	1
IPR000582	Acyl-CoA-binding protein ACBP	34	1	0
IPR001236	Lactate/malate dehydrogenase N-terminal	34	0	1
IPR001580	Calreticulin/calnexin	34	1	0
IPR001666	Phosphatidylinositol transfer protein	34	1	0
IPR003032	Ryanodine receptor Ryr	34	0	1
IPR003938	Potassium channel, voltage-dependent, EAG/ELK/ERG	34	1	0
IPR005282	Lysosomal cystine transporter	34	0	1
IPR006693	Partial AB-hydrolase lipase domain	34	0	1
IPR012676	TGS-like	34	0	1
IPR014821	Inositol 145-trisphosphate/ryanodine receptor	34	0	1
IPR032923	Kinesin-like protein KIF13A	34	0	1
IPR000539	Frizzled protein	34	0	0

**InterPro signature**	**InterPro description**	**Shared *Papaipema* genes**	***Papaipema sp.4* SSGs**	***Papaipema speciosissima* SSGs**
IPR001296	Glycosyl transferase family 1	34	0	0
IPR001305	Heat shock protein DnaJ cysteine-rich domain	34	0	0
IPR001369	PNP/MTAP phosphorylase	34	0	0
IPR001406	Pseudouridine synthase I, TruA	34	0	0
IPR001661	Glycoside hydrolase, family 37	34	0	0
IPR002155	Thiolase	34	0	0
IPR004142	NDRG	34	0	0
IPR004147	UbiB domain	34	0	0
IPR004181	Zinc finger MIZ-type	34	0	0
IPR004254	AdipoR/Haemolysin-III-related	34	0	0
IPR005482	Biotin carboxylase C-terminal	34	0	0
IPR005720	Dihydroorotate dehydrogenase domain	34	0	0
IPR006560	AWS domain	34	0	0
IPR006876	LMBR1-like membrane protein	34	0	0
IPR007274	Ctr copper transporter	34	0	0
IPR007644	RNA polymerase beta subunit protrusion	34	0	0
IPR007645	RNA polymerase Rpb2 domain 3	34	0	0
IPR007861	DNA mismatch repair protein MutS clamp	34	0	0
IPR009832	Protein of unknown function DUF1397	34	0	0
IPR015526	Frizzled/secreted frizzled-related protein	34	0	0

InterPro signature	InterPro description	Shared *Papaipema* genes	*Papaipema sp.4* SSGs	*Papaipema speciosissima* SSGs
IPR019474	Ubiquitin conjugation factor E4 core	34	0	0
IPR027775	C2H2- zinc finger protein family	34	0	0
IPR006571	TLDc domain	33	4	0
IPR001263	Phosphoinositide 3-kinase accessory PIK domain	33	2	0
IPR004010	Cache domain	33	0	2
IPR012936	Endoplasmic reticulum vesicle transporter C-terminal	33	1	1
IPR003846	Uncharacterised protein family UPF0061	33	0	1
IPR004172	L27 domain	33	0	1
IPR004279	Perilipin	33	0	1
IPR004871	Cleavage/polyadenylation specificity factor A subunit C-terminal	33	0	1
IPR010997	HRDC-like	33	1	0
IPR015449	Potassium channel, calcium-activated, SK	33	0	1
IPR019591	Mrp/NBP35 ATP-binding protein	33	0	1
IPR022776	TRM13/UPF0224 family U11-48K-like CHHC zinc finger domain	33	0	1
IPR000156	Ran binding domain	33	0	0
IPR000690	Zinc finger C2H2-type matrin	33	0	0
IPR001557	L-lactate/malate dehydrogenase	33	0	0
IPR002404	IRS-type PTB domain	33	0	0
IPR005481	Biotin carboxylase-like N-terminal domain	33	0	0
IPR007146	Sas10/Utp3/C1D	33	0	0

InterPro signature	InterPro description	Shared *Papaipema* genes	*Papaipema sp.4* SSGs	*Papaipema speciosissima* SSGs
IPR007271	Nucleotide-sugar transporter	33	0	0
IPR008011	Complex 1 LYR protein	33	0	0
IPR008183	Aldose 1-/Glucose-6-phosphate 1-epimerase	33	0	0
IPR013020	DNA helicase DNA repair), Rad3 type	33	0	0
IPR013977	Glycine cleavage T-protein C-terminal barrel domain	33	0	0
IPR015615	Transforming growth factor-beta-related	33	0	0
IPR015722	Histone-lysine N-methyltransferase Trr	33	0	0
IPR015955	Lactate dehydrogenase/glycoside hydrolase family 4 C-terminal	33	0	0
IPR019177	Golgin subfamily A member 5	33	0	0
IPR020478	AT hook-like	33	0	0
IPR022383	Lactate/malate dehydrogenase C-terminal	33	0	0
IPR024518	Domain of unknown function DUF3421	33	0	0
IPR030386	GB1/RHD3-type guanine nucleotide-binding G domain	33	0	0
IPR002791	Domain of unknown function DUF89	32	2	2
IPR027663	Dynactin subunit 1	32	2	1
IPR000896	Hemocyanin/hexamerin middle domain	32	1	1
IPR008922	Uncharacterised domain di-copper centre	32	1	1
IPR010982	Lambda repressor-like DNA-binding domain	32	1	1
IPR031481	Fucosyltransferase N-terminal	32	1	1
IPR002934	Polymerase nucleotidyl transferase domain	32	1	0

**InterPro signature**	**InterPro description**	**Shared *Papaipema* genes**	***Papaipema sp.4* SSGs**	***Papaipema speciosissima* SSGs**
IPR003096	Smooth muscle protein/calponin	32	0	1
IPR011004	Trimeric LpxA-like	32	0	1
IPR013606	IMD/I-BAR domain	32	0	1
IPR023608	Protein-glutamine gamma-glutamyltransferase, eukaryota	32	1	0
IPR027748	Tubulin polyglutamylase TTLL-4	32	1	0
IPR031259	Intracellular lipid binding protein	32	0	1
IPR000432	DNA mismatch repair protein MutS C-terminal	32	0	0
IPR000866	Alkyl hydroperoxide reductase subunit C/ Thiol specific antioxidant	32	0	0
IPR001696	Voltage gated sodium channel, alpha subunit	32	0	0
IPR002504	NAD kinase	32	0	0
IPR002933	Peptidase M20	32	0	0
IPR003891	Initiation factor eIF-4 gamma MA3	32	0	0
IPR004434	Isocitrate dehydrogenase NAD-dependent	32	0	0
IPR005016	Serine incorporator/TMS membrane protein	32	0	0
IPR005946	Ribose-phosphate diphosphokinase	32	0	0
IPR006634	TRAM/LAG1/CLN8 homology domain	32	0	0
IPR007642	RNA polymerase Rpb2 domain 2	32	0	0
IPR007865	Aminopeptidase P N-terminal	32	0	0
IPR008972	Cupredoxin	32	0	0
IPR012919	SUN domain	32	0	0

InterPro signature	InterPro description	Shared *Papaipema* genes	*Papaipema sp.4* SSGs	*Papaipema speciosissima* SSGs
IPR019559	Cullin protein neddylation domain	32	0	0
IPR020097	Pseudouridine synthase I TruA alpha/beta domain	32	0	0
IPR025749	Sphingomyelin synthase-like domain	32	0	0
IPR026739	AP complex subunit beta	32	0	0
IPR026823	Complement Clr-like EGF domain	32	0	0
IPR028651	ING family	32	0	0
IPR029067	CDC48 domain 2-like	32	0	0
IPR029529	Transcription factor IIIB subunit Brf1	32	0	0
IPR011010	DNA breaking-rejoining enzyme catalytic core	31	3	9
IPR005203	Hemocyanin C-terminal	31	2	4
IPR000698	Arrestin	31	3	2
IPR032628	Adenylate cyclase N-terminal	31	2	1
IPR001030	Aconitase/3-isopropylmalate dehydratase large subunit alpha/beta/alpha domain	31	1	1
IPR008963	Purple acid phosphatase-like N-terminal	31	2	0
IPR015914	Purple acid phosphatase N-terminal	31	2	0
IPR000814	TATA-box binding protein	31	0	1
IPR001604	DNA/RNA non-specific endonuclease	31	1	0
IPR003150	DNA-binding RFX-type winged-helix domain	31	1	0
IPR004302	Chitin-binding domain 3	31	1	0
IPR004686	Tricarboxylate/iron carrier	31	0	1

InterPro signature	InterPro description	Shared *Papaipema* genes	*Papaipema sp.4* SSGs	*Papaipema speciosissima* SSGs
IPR007822	Lanthionine synthetase C-like	31	0	1
IPR008530	Coiled-coil domain-containing protein 22	31	0	1
IPR010703	Dedicator of cytokinesis C-terminal	31	1	0
IPR013748	Replication factor C C-terminal	31	0	1
IPR022735	Domain of unknown function DUF3585	31	0	1
IPR027357	DHR-2 domain	31	1	0
IPR000239	GPCR kinase	31	0	0
IPR000537	UbiA prenyltransferase family	31	0	0
IPR000577	Carbohydrate kinase, FGGY	31	0	0
IPR000720	Copper type II ascorbate-dependent monooxygenase/peptidyl-alpha-hydroxyglycine alpha-amidating lyase	31	0	0
IPR000960	Flavin monooxygenase FMO	31	0	0
IPR001139	Glycoside hydrolase, family 30	31	0	0
IPR001392	Clathrin adaptor, mu subunit	31	0	0
IPR002501	Pseudouridine synthase II N-terminal	31	0	0
IPR002777	Prefoldin beta-like	31	0	0
IPR004179	Sec63 domain	31	0	0
IPR004589	DNA helicase, ATP-dependent, RecQ type	31	0	0
IPR005843	Alpha-D-phosphohexomutase C-terminal	31	0	0
IPR016355	Nuclear hormone receptor family 5	31	0	0
IPR023209	D-amino-acid oxidase	31	0	0

**InterPro signature**	**InterPro description**	**Shared *Papaipema* genes**	***Papaipema sp.4* SSGs**	***Papaipema speciosissima* SSGs**
IPR029099	Ribose-phosphate pyrophosphokinase N-terminal domain	31	0	0
IPR030142	Putative calcium-activated chloride channel protein, ciliates	31	0	0
IPR032421	Protein O-mannosyl-transferase C-terminal four TM domain	31	0	0
IPR009025	DNA-directed RNA polymerase RBP11-like dimerisation domain	30	4	0
IPR031597	KELK-motif containing domain	30	0	3
IPR008253	Marvel domain	30	1	1
IPR008909	DALR anticodon binding	30	2	0
IPR012678	Ribosomal protein L23/L15e core domain	30	1	1
IPR013558	CTNNB1 binding N-teminal	30	1	1
IPR002717	Histone acetyltransferase domain MYST-type	30	1	0
IPR004100	ATPase F1 complex alpha/beta subunit N-terminal domain	30	1	0
IPR011257	DNA glycosylase	30	1	0
IPR013662	RyR/IP3R Homology associated domain	30	0	1
IPR000380	DNA topoisomerase, type IA	30	0	0
IPR001104	3-oxo-5-alpha-steroid 4-dehydrogenase C-terminal	30	0	0
IPR001772	Kinase associated domain 1 KA1	30	0	0
IPR003191	Guanylate-binding protein C-terminal	30	0	0
IPR004143	Biotinyl protein ligase BPL and lipoyl protein ligase LPL catalytic domain	30	0	0
IPR004853	Sugar phosphate transporter domain	30	0	0
IPR005442	Glutathione S-transferase, omega-class	30	0	0

InterPro signature	InterPro description	Shared *Papaipema* genes	*Papaipema sp.4* SSGs	*Papaipema speciosissima* SSGs
IPR005788	Disulphide isomerase	30	0	0
IPR006172	DNA-directed DNA polymerase, family B	30	0	0
IPR006206	Mevalonate/galactokinase	30	0	0
IPR006576	BRK domain	30	0	0
IPR008366	Nuclear factor of activated T cells NFAT)	30	0	0
IPR010918	AIR synthase-related protein C-terminal domain	30	0	0
IPR011030	Vitellinogen superhelical	30	0	0
IPR011037	Pyruvate kinase-like insert domain	30	0	0
IPR011063	tRNAIle-lysidine/2-thiocytidine synthase N-terminal	30	0	0
IPR012101	Biotinidase, eukaryotic	30	0	0
IPR013024	Butirosin biosynthesis BtrG-like	30	0	0
IPR013178	Histone acetyltransferase Rtt109/CBP	30	0	0
IPR013857	NADH:ubiquinone oxidoreductase intermediate-associated protein 30	30	0	0
IPR015927	Peptidase S24/S26A/S26B/S26C	30	0	0
IPR016274	Histidine acid phosphatase, eukaryotic	30	0	0
IPR017923	Transcription factor IIS N-terminal	30	0	0
IPR018492	Ribosomal protein L7Ae/L8/Nhp2 family	30	0	0
IPR018500	DDT domain subgroup	30	0	0
IPR019323	Active zone protein ELKS	30	0	0
IPR019339	CBF1-interacting co-repressor CIR N-terminal domain	30	0	0

InterPro signature	InterPro description	Shared *Papaipema* genes	*Papaipema sp.4* SSGs	*Papaipema speciosissima* SSGs
IPR019448	EEIG1/EHBP1 N-terminal domain	30	0	0
IPR019479	Peroxiredoxin C-terminal	30	0	0
IPR019759	Peptidase S24/S26A/S26B	30	0	0
IPR023405	DNA topoisomerase type IA core domain	30	0	0
IPR024610	Inhibitor of growth protein N-terminal histone-binding	30	0	0
IPR026728	UHRF1-binding protein 1-like	30	0	0
IPR029043	Glycine cleavage T-protein/YgfZ C-terminal domain	30	0	0
IPR031162	CBP/p300-type histone acetyltransferase domain	30	0	0
IPR026983	Dynein heavy chain	29	12	6
IPR001995	Peptidase A2A retrovirus catalytic	29	7	4
IPR002453	Beta tubulin	29	3	4
IPR007282	NOT2/NOT3/NOT5	29	2	0
IPR000891	Pyruvate carboxyltransferase	29	1	1
IPR025733	Iron/zinc purple acid phosphatase-like C-terminal domain	29	2	0
IPR025995	RNA binding activity-knot of a chromodomain	29	2	0
IPR026962	Katanin p80 subunit B1	29	2	0
IPR002467	Peptidase M24A, methionine aminopeptidase, subfamily 1	29	0	1
IPR005775	P-type ATPase, subfamily IIC	29	0	1
IPR007829	TM2 domain	29	0	1
IPR013333	Ryanodine receptor	29	1	0

InterPro signature	InterPro description	Shared *Papaipema* genes	*Papaipema sp.4* SSGs	*Papaipema speciosissima* SSGs
IPR027351	#NAME?	29	1	0
IPR027658	Formin, protein diaphanous	29	0	1
IPR032642	DNA mismatch repair protein Msh2	29	0	1
IPR000402	Sodium/potassium-transporting ATPase subunit beta	29	0	0
IPR000642	Peptidase M41	29	0	0
IPR000924	Glutamyl/glutaminyl-tRNA synthetase	29	0	0
IPR001597	Aromatic amino acid beta-eliminating lyase/threonine aldolase	29	0	0
IPR001747	Lipid transport protein N-terminal	29	0	0
IPR002119	Histone H2A	29	0	0
IPR003069	Ecdysteroid receptor	29	0	0
IPR004183	Extradiol ring-cleavage dioxygenase class III enzyme subunit B	29	0	0
IPR006034	Asparaginase/glutaminase	29	0	0
IPR006134	DNA-directed DNA polymerase family B multifunctional domain	29	0	0
IPR006145	Pseudouridine synthase RsuA/RluB/C/D/E/F	29	0	0
IPR006383	HAD-superfamily hydrolase, subfamily IB, PSPase-like	29	0	0
IPR006935	Helicase/UvrB N-terminal	29	0	0
IPR007066	RNA polymerase Rpb1 domain 3	29	0	0
IPR008999	Actin cross-linking	29	0	0
IPR009051	Alpha-helical ferredoxin	29	0	0
IPR011084	DNA repair metallo-beta-lactamase	29	0	0

InterPro signature	InterPro description	Shared *Papaipema* genes	*Papaipema sp.4* SSGs	*Papaipema speciosissima* SSGs
IPR012976	NOSIC	29	0	0
IPR013144	CRA domain	29	0	0
IPR013497	DNA topoisomerase type IA central	29	0	0
IPR016188	PurM-like N-terminal domain	29	0	0
IPR019416	Nuclear cap-binding protein subunit 3	29	0	0
IPR019581	Pre-mRNA-processing-splicing factor 8 U5-snRNA-binding	29	0	0
IPR020476	NUDIX hydrolase	29	0	0
IPR022157	Dynein associated protein	29	0	0
IPR023970	Methylthiotransferase/radical SAM-type protein	29	0	0
IPR029136	Nuclear protein MDM1	29	0	0
IPR030378	Circularly permuted CP-type guanine nucleotide-binding G domain	29	0	0
IPR031962	Domain of unknown function DUF4781	29	0	0
IPR022048	Protein of unknown function DUF3609	28	2	5
IPR001019	Guanine nucleotide binding protein G-protein), alpha subunit	28	1	3
IPR019080	YqaJ viral recombinase	28	2	1
IPR002331	Pancreatic lipase	28	2	0
IPR009078	Ferritin-like superfamily	28	0	2
IPR022284	Glycerol-3-phosphate O-acyltransferase/Dihydroxyacetone phosphate acyltransferase	28	1	1
IPR000352	Peptide chain release factor class I/class II	28	0	1
IPR002376	Formyl transferase N-terminal	28	1	0

**InterPro signature**	**InterPro description**	**Shared *Papaipema* genes**	***Papaipema sp.4* SSGs**	***Papaipema speciosissima* SSGs**
IPR004095	TGS	28	0	1
IPR004481	Sodium/potassium/calcium exchanger	28	1	0
IPR008334	5'-Nucleotidase C-terminal	28	0	1
IPR009033	Calreticulin/calnexin P domain	28	0	1
IPR011025	G protein alpha subunit helical insertion	28	1	0
IPR011259	Ezrin/radixin/moesin C-terminal	28	1	0
IPR016071	Staphylococcal nuclease SNase-like OB-fold	28	0	1
IPR021131	Ribosomal protein L18e/L15P	28	0	1
IPR022164	Kinesin-like	28	1	0
IPR023674	Ribosomal protein L1-like	28	0	1
IPR000836	Phosphoribosyltransferase domain	28	0	0
IPR001807	Chloride channel, voltage gated	28	0	0
IPR001839	Transforming growth factor-beta C-terminal	28	0	0
IPR002092	DNA-directed RNA polymerase, phage-type	28	0	0
IPR002109	Glutaredoxin	28	0	0
IPR002153	Transient receptor potential channel, canonical	28	0	0
IPR002303	Valine-tRNA ligase	28	0	0
IPR003265	HhH-GPD domain	28	0	0
IPR004022	DDT domain	28	0	0
IPR004240	Nonaspanin TM9SF)	28	0	0

InterPro signature	InterPro description	Shared *Papaipema* genes	*Papaipema sp.4* SSGs	*Papaipema speciosissima* SSGs
IPR004307	TspO/MBR-related protein	28	0	0
IPR006630	RNA-binding protein Lupus La	28	0	0
IPR007083	RNA polymerase Rpb1 domain 4	28	0	0
IPR008153	Clathrin adaptor gamma-adaptin appendage	28	0	0
IPR010303	Domain of unknown function DUF902 CREBbp	28	0	0
IPR010497	Epoxide hydrolase N-terminal	28	0	0
IPR011524	SARAH domain	28	0	0
IPR012164	DNA-directed RNA polymerase subunit/transcription factor S	28	0	0
IPR012258	Acyl-CoA oxidase	28	0	0
IPR013137	Zinc finger TFIIB-type	28	0	0
IPR013618	Domain of unknown function DUF1736	28	0	0
IPR014743	Chloride channel core	28	0	0
IPR015338	Exostosin C-terminal	28	0	0
IPR016763	Vesicle-associated membrane-protein-associated protein	28	0	0
IPR017941	Rieske [2Fe-2S] iron-sulphur domain	28	0	0
IPR021629	Mediator complex, subunit Med23	28	0	0
IPR022140	Kinesin-like KIF1-type	28	0	0
IPR022158	Inositol phosphatase	28	0	0
IPR025605	OST-HTH/LOTUS domain	28	0	0
IPR027073	5'-3' exoribonuclease	28	0	0

InterPro signature	InterPro description	Shared *Papaipema* genes	*Papaipema sp.4* SSGs	*Papaipema speciosissima* SSGs
IPR029001	Inosine triphosphate pyrophosphatase-like	28	0	0
IPR031185	MAGUK p55 subfamily member 5	28	0	0
IPR002077	Voltage-dependent calcium channel, alpha-1 subunit	27	3	6
IPR002558	I/LWEQ domain	27	1	2
IPR000793	ATPase F1/V1/A1 complex alpha/beta subunit C-terminal	27	1	1
IPR001711	Phospholipase C phosphatidylinositol-specific Y domain	27	2	0
IPR011603	2-oxoglutarate dehydrogenase E1 component	27	2	0
IPR013608	VWA N-terminal	27	0	2
IPR029023	Tensin phosphatase lipid phosphatase domain	27	2	0
IPR000304	Pyrroline-5-carboxylate reductase	27	0	1
IPR001564	Nucleoside diphosphate kinase	27	1	0
IPR002259	Equilibrative nucleoside transporter	27	0	1
IPR008254	Flavodoxin/nitric oxide synthase	27	0	1
IPR018164	Alanyl-tRNA synthetase class IIc N-terminal	27	1	0
IPR027707	Troponin T	27	0	1
IPR027799	Replication termination factor 2 RING-finger	27	1	0
IPR029039	Flavoprotein-like domain	27	0	1
IPR031468	Synaptotagmin-like mitochondrial-lipid-binding domain	27	0	1
IPR000631	ATP-dependent S)-NADP)H-hydrate dehydratase	27	0	0
IPR001212	Somatomedin B domain	27	0	0

InterPro signature	InterPro description	Shared *Papaipema* genes	*Papaipema sp.4* SSGs	*Papaipema speciosissima* SSGs
IPR002090	Na+/H+ exchanger, isoform 6 NHE6)	27	0	0
IPR002554	Protein phosphatase 2A, regulatory B subunit, B56	27	0	0
IPR004129	Glycerophosphoryl diester phosphodiesterase	27	0	0
IPR004152	GAT domain	27	0	0
IPR004316	SWEET sugar transporter	27	0	0
IPR005026	SAPAP family	27	0	0
IPR005036	CBM21 carbohydrate binding type-21 domain	27	0	0
IPR005178	Organic solute transporter subunit alpha/Transmembrane protein 184	27	0	0
IPR005612	CCAAT-binding factor	27	0	0
IPR005999	Glycerol kinase	27	0	0
IPR006166	ERCC4 domain	27	0	0
IPR006984	rRNA-processing protein Fcf1/Utp23	27	0	0
IPR007070	GPI ethanolamine phosphate transferase 1	27	0	0
IPR007258	Vps52	27	0	0
IPR007286	EAP30	27	0	0
IPR009637	Lung seven transmembrane receptor-like	27	0	0
IPR011039	Transcription Factor IIF Rap30/Rap74 interaction	27	0	0
IPR011234	Fumarylacetoacetase C-terminal-related	27	0	0
IPR011706	Multicopper oxidase type 2	27	0	0
IPR013937	Sorting nexin C-terminal	27	0	0

InterPro signature	InterPro description	Shared *Papaipema* genes	*Papaipema sp.4* SSGs	*Papaipema speciosissima* SSGs
IPR015507	Ribosomal RNA large subunit methyltransferase E	27	0	0
IPR016435	Diphthamide synthesis DPH1/DPH2	27	0	0
IPR018629	Transport protein XK	27	0	0
IPR019378	GDP-fucose protein O-fucosyltransferase	27	0	0
IPR024771	SUZ domain	27	0	0
IPR027251	Diacylglycerol O-acyltransferase 1	27	0	0
IPR029036	Pyrroline-5-carboxylate reductase dimerisation domain	27	0	0
IPR029066	PLP-binding barrel	27	0	0
IPR011057	Mss4-like	26	1	5
IPR001978	Troponin	26	1	4
IPR005517	Translation elongation factor EFG/EF2 domain IV	26	0	3
IPR015767	Rho GTPase activating protein	26	0	3
IPR001519	Ferritin	26	2	0
IPR000101	Gamma-glutamyltranspeptidase	26	0	1
IPR001098	DNA-directed DNA polymerase family A palm domain	26	0	1
IPR002139	Ribokinase	26	1	0
IPR008139	Saposin B type domain	26	0	1
IPR008958	Transglutaminase C-terminal	26	0	1
IPR011001	Saposin-like	26	0	1
IPR019384	Retinoic acid induced 16-like protein	26	0	1

InterPro signature	InterPro description	Shared *Papaipema* genes	*Papaipema sp.4* SSGs	*Papaipema speciosissima* SSGs
IPR024990	Anaphase-promoting complex subunit 1	26	1	0
IPR026784	Constitutive coactivator of PPAR-gamma	26	0	1
IPR029123	Splicing factor RBM39 linker	26	1	0
IPR000023	Phosphofructokinase domain	26	0	0
IPR000197	Zinc finger TAZ-type	26	0	0
IPR000243	Peptidase T1A, proteasome beta-subunit	26	0	0
IPR000270	PB1 domain	26	0	0
IPR000716	Thyroglobulin type-1	26	0	0
IPR001278	Arginine-tRNA ligase	26	0	0
IPR002059	Cold-shock protein DNA-binding	26	0	0
IPR002151	Kinesin light chain	26	0	0
IPR003342	Glycosyl transferase family 39/83	26	0	0
IPR005886	UDP-glucose 4-epimerase GalE	26	0	0
IPR006577	UAS	26	0	0
IPR006598	Lipopolysaccharide-modifying protein	26	0	0
IPR006629	LPS-induced tumour necrosis factor alpha factor	26	0	0
IPR007194	Transport protein particle TRAPP component	26	0	0
IPR007647	RNA polymerase Rpb2 domain 5	26	0	0
IPR009581	FAM20 C-terminal	26	0	0
IPR011129	Cold shock protein	26	0	0

InterPro signature	InterPro description	Shared *Papaipema* genes	*Papaipema sp.4* SSGs	*Papaipema speciosissima* SSGs
IPR012423	Chromatin modification-related protein Eaf7/MRGBP	26	0	0
IPR013022	Xylose isomerase-like TIM barrel domain	26	0	0
IPR013800	STAT transcription factor all-alpha domain	26	0	0
IPR013894	RecQ mediated genome instability protein N-terminal	26	0	0
IPR014811	Argonaute linker 1 domain	26	0	0
IPR015010	Rap1 Myb domain	26	0	0
IPR015649	Schwannomin interacting protein 1	26	0	0
IPR015819	Lipid transport protein beta-sheet shell	26	0	0
IPR015988	STAT transcription factor coiled coil	26	0	0
IPR018834	DNA/RNA-binding domain Est1-type	26	0	0
IPR019540	Phosphatidylinositol-glycan biosynthesis class S protein	26	0	0
IPR019577	SPARC/Testican calcium-binding domain	26	0	0
IPR020058	Glutamyl/glutaminyl-tRNA synthetase class Ib catalytic domain	26	0	0
IPR022102	Holliday junction regulator protein family C-terminal	26	0	0
IPR022310	NAD/GMP synthase	26	0	0
IPR022953	ATP-dependent 6-phosphofructokinase	26	0	0
IPR023115	Translation initiation factor IF- 2 domain 3	26	0	0
IPR023577	CYTH-like domain	26	0	0
IPR024869	FAM20	26	0	0
IPR026699	Exosome complex RNA-binding protein 1/RRP40/RRP4	26	0	0

InterPro signature	InterPro description	Shared *Papaipema* genes	*Papaipema sp.4* SSGs	*Papaipema speciosissima* SSGs
IPR029068	Glyoxalase/Bleomycin resistance protein/Dihydroxybiphenyl dioxygenase	26	0	0
IPR031865	Domain of unknown function DUF4757	26	0	0
IPR032098	Acyltransferase C-terminal domain	26	0	0
IPR032405	Kinesin-associated	26	0	0
IPR032443	RAWUL domain	26	0	0
IPR032632	Peptidase M16 middle/third domain	26	0	0
IPR029047	Heat shock protein 70kD peptide-binding domain	25	3	16
IPR015359	Phosphoinositide-specific phospholipase C EF-hand-like domain	25	4	0
IPR001312	Hexokinase	25	0	3
IPR020575	Heat shock protein Hsp90 N-terminal	25	1	2
IPR000375	Dynamin central domain	25	1	1
IPR001926	Tryptophan synthase beta subunit-like PLP-dependent enzyme	25	2	0
IPR002297	DNA-directed DNA-polymerase, family A, mitochondria	25	1	1
IPR005122	Uracil-DNA glycosylase-like	25	2	0
IPR007320	Programmed cell death protein 2 C-terminal	25	2	0
IPR031057	DOCK1 homologue	25	1	1
IPR031756	Beta-13-glucan-binding protein N-terminal	25	0	2
IPR000228	RNA 3'-terminal phosphate cyclase	25	1	0
IPR001632	G-protein beta subunit	25	1	0
IPR002539	MaoC-like domain	25	0	1

InterPro signature	InterPro description	Shared *Papaipema* genes	*Papaipema sp.4* SSGs	*Papaipema speciosissima* SSGs
IPR002735	Translation initiation factor IF2/IF5	25	0	1
IPR005201	Glycoside hydrolase family 85	25	1	0
IPR013792	RNA 3'-terminal phosphate cyclase/enolpyruvate transferase alpha/beta	25	0	1
IPR014930	Myotonic dystrophy protein kinase coiled coil	25	1	0
IPR015414	Transmembrane protein TMEM64/TMEM41	25	1	0
IPR016190	Translation initiation factor IF2/IF5 zinc-binding	25	0	1
IPR017920	COMM domain	25	0	1
IPR020464	LanC-like protein, eukaryotic	25	0	1
IPR023797	RNA 3'-terminal phosphate cyclase domain	25	0	1
IPR027741	Dynamin-1	25	0	1
IPR032062	Protein of unknown function DUF4803	25	0	1
IPR032979	Cytosolic endo-beta-N-acetylglucosaminidase	25	0	1
IPR026847	Vacuolar protein sorting-associated protein 13	25	0	0
IPR000003	Retinoid X receptor/HNF4	25	0	0
IPR000010	Cystatin domain	25	0	0
IPR000043	Adenosylhomocysteinase	25	0	0
IPR000764	Uridine kinase-like	25	0	0
IPR001094	Flavodoxin-like	25	0	0
IPR001461	Aspartic peptidase A1 family	25	0	0
IPR001648	Ribosomal protein S18	25	0	0

InterPro signature	InterPro description	Shared *Papaipema* genes	*Papaipema sp.4* SSGs	*Papaipema speciosissima* SSGs
IPR001910	Inosine/uridine-preferring nucleoside hydrolase domain	25	0	0
IPR002344	Lupus La protein	25	0	0
IPR002848	Translin	25	0	0
IPR002890	Alpha-2-macroglobulin N-terminal	25	0	0
IPR003018	GAF domain	25	0	0
IPR003092	Two pore domain potassium channel, TASK family	25	0	0
IPR003349	JmjN domain	25	0	0
IPR003448	Molybdopterin biosynthesis MoaE	25	0	0
IPR003609	PAN/Apple domain	25	0	0
IPR003674	Oligosaccharyl transferase, STT3 subunit	25	0	0
IPR004113	FAD-linked oxidase C-terminal	25	0	0
IPR005176	Potentiating neddylation domain	25	0	0
IPR005636	DTW	25	0	0
IPR006641	YqgF/RNase H-like domain	25	0	0
IPR007526	SWIRM domain	25	0	0
IPR007646	RNA polymerase Rpb2 domain 4	25	0	0
IPR008636	Hook-related protein family	25	0	0
IPR008676	MRG	25	0	0
IPR008977	PHM/PNGase F domain	25	0	0
IPR009011	Mannose-6-phosphate receptor binding domain	25	0	0

**InterPro signature**	**InterPro description**	**Shared *Papaipema* genes**	***Papaipema sp.4* SSGs**	***Papaipema speciosissima* SSGs**
IPR010339	TIP49 C-terminal	25	0	0
IPR011108	RNA-metabolising metallo-beta-lactamase	25	0	0
IPR011645	Haem NO binding associated	25	0	0
IPR012981	PIH1 family	25	0	0
IPR013996	PX-associated sorting nexin 13	25	0	0
IPR015255	Vitellinogen open beta-sheet	25	0	0
IPR015496	Ubiquilin	25	0	0
IPR017861	Kae1/TsaD family	25	0	0
IPR017961	DNA polymerase Y-family little finger domain	25	0	0
IPR018123	WWE domain subgroup	25	0	0
IPR022764	Peptidase S54 rhomboid domain	25	0	0
IPR024706	Peroxiredoxin, AhpC-type	25	0	0
IPR025256	Domain of unknown function DUF4203	25	0	0
IPR025887	Glycoside hydrolase family 31 N-terminal domain	25	0	0
IPR026059	Rab3-GAP regulatory subunit	25	0	0
IPR026079	Cerebellar degeneration-related protein 2	25	0	0
IPR026541	MRG domain	25	0	0
IPR027238	RuvB-like	25	0	0
IPR029016	GAF domain-like	25	0	0
IPR029346	Ubiquitin carboxyl-terminal hydrolase C-terminal	25	0	0

InterPro signature	InterPro description	Shared *Papaipema* genes	*Papaipema sp.4* SSGs	*Papaipema speciosissima* SSGs
IPR029489	O-GlcNAc transferase C-terminal	25	0	0
IPR030376	Cap-specific mRNA nucleoside-2-O-)-methyltransferase 1	25	0	0
IPR030445	Histone H3-K79 methyltransferase	25	0	0
IPR031867	MiT/TFE transcription factors N-terminal	25	0	0
IPR032284	ATP-dependent DNA helicase RecQ zinc-binding domain	25	0	0
IPR032454	Histone H2A C-terminal domain	25	0	0
IPR032781	ABC-transporter extension domain	25	0	0
IPR032817	Mon2 C-terminal	25	0	0
IPR033121	Peptidase family A1 domain	25	0	0
IPR003840	DNA helicase	24	3	5
IPR004245	Protein of unknown function DUF229	24	3	0
IPR006011	Syntaxin N-terminal domain	24	2	1
IPR008380	HAD-superfamily hydrolase, subfamily IG, 5'-nucleotidase	24	1	1
IPR008551	Transport and Golgi organisation protein 2	24	0	2
IPR013799	STAT transcription factor protein interaction	24	2	0
IPR022058	Protein of unknown function DUF3610	24	1	1
IPR001273	Aromatic amino acid hydroxylase	24	0	1
IPR007081	RNA polymerase Rpb1 domain 5	24	0	1
IPR010978	tRNA-binding arm	24	0	1
IPR011650	Peptidase M20 dimerisation domain	24	0	1

InterPro signature	InterPro description	Shared *Papaipema* genes	*Papaipema sp.4* SSGs	*Papaipema speciosissima* SSGs
IPR013584	RAP domain	24	1	0
IPR016189	Translation initiation factor IF2/IF5 N-terminal	24	0	1
IPR019774	Aromatic amino acid hydroxylase C-terminal	24	1	0
IPR026314	YLP motif-containing protein 1	24	0	1
IPR028939	Pyrroline-5-carboxylate reductase catalytic N-terminal	24	1	0
IPR000261	EH domain	24	0	0
IPR000572	Oxidoreductase molybdopterin-binding domain	24	0	0
IPR000800	Notch domain	24	0	0
IPR000905	Gcp-like domain	24	0	0
IPR000906	ZU5 domain	24	0	0
IPR000967	Zinc finger NF-X1-type	24	0	0
IPR001055	Adrenodoxin	24	0	0
IPR001078	2-oxoacid dehydrogenase acyltransferase catalytic domain	24	0	0
IPR001126	UmuC domain	24	0	0
IPR002030	Mitochondrial brown fat uncoupling protein	24	0	0
IPR002075	Nuclear transport factor 2	24	0	0
IPR002167	Graves disease carrier protein	24	0	0
IPR002171	Ribosomal protein L2	24	0	0
IPR003195	Transcription initiation factor IID, 18kDa subunit	24	0	0
IPR003378	Fringe-like	24	0	0

**InterPro signature**	**InterPro description**	**Shared *Papaipema* genes**	***Papaipema sp.4* SSGs**	***Papaipema speciosissima* SSGs**
IPR003738	SOS response associated peptidase SRAP)	24	0	0
IPR005627	Copper homeostasis protein CutC	24	0	0
IPR005637	TAP C-terminal TAP-C domain	24	0	0
IPR006171	Toprim domain	24	0	0
IPR006845	Pex N-terminal	24	0	0
IPR006939	SNF5/SMARCB1/INI1	24	0	0
IPR007590	CWC16 protein	24	0	0
IPR007651	Lipin N-terminal	24	0	0
IPR008335	Eukaryotic molybdopterin oxidoreductase	24	0	0
IPR009019	K homology domain prokaryotic type	24	0	0
IPR010660	Notch NOD domain	24	0	0
IPR011707	Multicopper oxidase type 3	24	0	0
IPR012317	PolyADP-ribose polymerase catalytic domain	24	0	0
IPR019526	Nuclear respiratory factor-1 activation binding domain	24	0	0
IPR019535	Little elongation complex subunit 2 C-terminal	24	0	0
IPR019580	Pre-mRNA-processing-splicing factor 8 U6-snRNA-binding	24	0	0
IPR023186	Inosine/uridine-preferring nucleoside hydrolase	24	0	0
IPR023648	Copper homeostasis CutC domain	24	0	0
IPR025151	ELYS-like domain	24	0	0
IPR025789	Histone-lysine N-methyltransferase DOT1 domain	24	0	0

InterPro signature	InterPro description	Shared *Papaipema* genes	*Papaipema sp.4* SSGs	*Papaipema speciosissima* SSGs
IPR029320	Acyl-coenzyme A oxidase N-terminal	24	0	0
IPR032319	Polyribonucleotide 5'-hydroxyl-kinase Clp1 P-loop domain	24	0	0
IPR032816	SNARE associated Golgi protein	24	0	0
IPR013658	SMP-30/Gluconolactonase/LRE-like region	23	2	3
IPR011262	DNA-directed RNA polymerase insert domain	23	4	0
IPR011263	DNA-directed RNA polymerase RpoA/D/Rpb3-type	23	4	0
IPR002452	Alpha tubulin	23	0	2
IPR015876	Fatty acid desaturase, type 1, core	23	1	1
IPR018165	Alanyl-tRNA synthetase class IIc core domain	23	1	1
IPR022672	Hexokinase N-terminal	23	2	0
IPR000584	Voltage-dependent calcium channel, L-type, beta subunit	23	1	0
IPR001991	Sodium:dicarboxylate symporter	23	1	0
IPR004178	Calmodulin-binding domain	23	1	0
IPR005052	Legume-like lectin	23	1	0
IPR005097	Saccharopine dehydrogenase NADP binding domain	23	1	0
IPR005810	Succinyl-CoA ligase, alpha subunit	23	1	0
IPR006509	Splicing factor, RBM39-like	23	0	1
IPR006692	Coatomer WD associated region	23	1	0
IPR007695	DNA mismatch repair protein MutS-like N-terminal	23	1	0
IPR007860	DNA mismatch repair protein MutS connector domain	23	1	0

InterPro signature	InterPro description	Shared *Papaipema* genes	*Papaipema sp.4* SSGs	*Papaipema speciosissima* SSGs
IPR012896	Integrin beta subunit tail	23	1	0
IPR022673	Hexokinase C-terminal	23	1	0
IPR029919	Protein flightless-1	23	1	0
IPR032017	FAM76 protein	23	0	1
IPR000323	Copper type II ascorbate-dependent monooxygenase N-terminal	23	0	0
IPR001577	Peptidase M8, leishmanolysin	23	0	0
IPR001977	Dephospho-CoA kinase	23	0	0
IPR002131	Glycoprotein hormone receptor family	23	0	0
IPR002301	Isoleucine-tRNA ligase	23	0	0
IPR002320	Threonine-tRNA ligase, class IIa	23	0	0
IPR002634	BolA protein	23	0	0
IPR002678	GTP cyclohydrolase 1 type 2/Nif3	23	0	0
IPR002884	Proprotein convertase P	23	0	0
IPR003116	Raf-like Ras-binding	23	0	0
IPR003606	Pre-SET zinc-binding sub-group	23	0	0
IPR003648	Splicing factor motif	23	0	0
IPR003971	Potassium channel, voltage dependent, Kv9	23	0	0
IPR005146	B3/B4 tRNA-binding domain	23	0	0
IPR006357	HAD-superfamily hydrolase, subfamily IIA	23	0	0
IPR006816	ELMO domain	23	0	0

**InterPro signature**	**InterPro description**	**Shared *Papaipema* genes**	***Papaipema sp.4* SSGs**	***Papaipema speciosissima* SSGs**
IPR006977	Yip1 domain	23	0	0
IPR007034	Ribosome biogenesis protein BMS1/TSR1 C-terminal	23	0	0
IPR007641	RNA polymerase Rpb2 domain 7	23	0	0
IPR009014	Transketolase C-terminal/Pyruvate-ferredoxin oxidoreductase domain II	23	0	0
IPR009635	Neural proliferation differentiation control-1	23	0	0
IPR012901	N2227-like	23	0	0
IPR013085	Zinc finger U1-C type	23	0	0
IPR013273	Peptidase M12B, ADAM-TS	23	0	0
IPR013806	Kringle-like fold	23	0	0
IPR014182	Alcohol dehydrogenase, zinc-binding type 1	23	0	0
IPR014436	Extradiol aromatic ring-opening dioxygenase, DODA type	23	0	0
IPR014906	Pre-mRNA processing factor 4 PRP4-like	23	0	0
IPR015878	S-adenosyl-L-homocysteine hydrolase NAD binding domain	23	0	0
IPR016082	Ribosomal protein L30 ferredoxin-like fold domain	23	0	0
IPR018881	Uncharacterised protein family UPF0565	23	0	0
IPR020864	Membrane attack complex component/perforin MACPF domain	23	0	0
IPR021012	Down syndrome cell adhesion molecule C-terminal	23	0	0
IPR021859	XRN-Two Binding Domain XTBD	23	0	0
IPR022669	Ribosomal protein L2 C-terminal	23	0	0
IPR022682	Peptidase C2 calpain large subunit domain III	23	0	0

InterPro signature	InterPro description	Shared *Papaipema* genes	*Papaipema sp.4* SSGs	*Papaipema speciosissima* SSGs
IPR024557	CCR4-Not complex Not1 subunit domain of unknown function DUF3819	23	0	0
IPR025721	Exosome complex component N-terminal domain	23	0	0
IPR026307	Transmembrane protein 132	23	0	0
IPR026509	Transmembrane protein 183	23	0	0
IPR027324	Microtubule associated protein MAP2/MAP4/Tau	23	0	0
IPR027794	tRNase Z endonuclease	23	0	0
IPR029008	Rab5-interacting protein family	23	0	0
IPR029041	FAD-linked oxidoreductase-like	23	0	0
IPR029426	FAM86	23	0	0
IPR030319	Plasma membrane calcium-transporting ATPase 1/4	23	0	0
IPR030798	Arfaptin family	23	0	0
IPR031675	Serine-threonine protein phosphatase N-terminal	23	0	0
IPR031692	EH domain-containing protein N-terminal	23	0	0
IPR032191	CCR4-NOT transcription complex subunit 1 CAF1-binding domain	23	0	0
IPR032471	GAIN domain N-terminal	23	0	0
IPR032503	FAD dependent oxidoreductase central domain	23	0	0
IPR014873	Voltage-dependent calcium channel alpha-1 subunit IQ domain	22	8	3
IPR000796	Aspartate/other aminotransferase	22	4	0
IPR002402	Cytochrome P450, E-class, group II	22	1	2
IPR002498	Phosphatidylinositol-4-phosphate 5-kinase core	22	1	2

InterPro signature	InterPro description	Shared *Papaipema* genes	*Papaipema sp.4* SSGs	*Papaipema speciosissima* SSGs
IPR003108	Growth-arrest-specific protein 2 domain	22	1	2
IPR004009	Myosin N-terminal SH3-like	22	1	2
IPR005511	Senescence marker protein-30 SMP-30)	22	3	0
IPR013094	Alpha/beta hydrolase fold-3	22	3	0
IPR002652	Importin-alpha importin-beta-binding domain	22	2	0
IPR004328	BRO1 domain	22	2	0
IPR006586	ADAM cysteine-rich	22	0	2
IPR015680	Glutamate-Gated Chloride Channel	22	1	1
IPR018011	Carbohydrate sulfotransferase-related	22	0	2
IPR023341	MABP domain	22	0	2
IPR032778	Growth factor receptor domain 4	22	2	0
IPR003781	CoA-binding	22	0	1
IPR004837	Sodium/calcium exchanger membrane region	22	0	1
IPR006108	3-hydroxyacyl-CoA dehydrogenase C-terminal	22	1	0
IPR006797	PRELI/MSF1 domain	22	1	0
IPR007305	Vesicle transport protein, Got1/SFT2-like	22	1	0
IPR007533	Cytochrome c oxidase assembly protein CtaG/Cox11	22	0	1
IPR009020	Proteinase inhibitor propeptide	22	1	0
IPR013650	ATP-grasp fold succinyl-CoA synthetase-type	22	0	1
IPR015415	Vps4 oligomerisation C-terminal	22	0	1

**InterPro signature**	**InterPro description**	**Shared *Papaipema* genes**	***Papaipema sp.4* SSGs**	***Papaipema speciosissima* SSGs**
IPR019143	JNK/Rab-associated protein-1 N-terminal	22	1	0
IPR019680	Mediator complex subunit Med1	22	1	0
IPR029585	Peroxidase, insect	22	0	1
IPR000001	Kringle	22	0	0
IPR000223	Peptidase S26A, signal peptidase I	22	0	0
IPR000573	Aconitase A/isopropylmalate dehydratase small subunit swivel domain	22	0	0
IPR000722	RNA polymerase alpha subunit	22	0	0
IPR000971	Globin	22	0	0
IPR001260	Coproporphyrinogen III oxidase, aerobic	22	0	0
IPR002472	Palmitoyl protein thioesterase	22	0	0
IPR002547	tRNA-binding domain	22	0	0
IPR002698	5-formyltetrahydrofolate cyclo-ligase	22	0	0
IPR002792	TRAM domain	22	0	0
IPR002809	Protein of unknown function DUF106, transmembrane	22	0	0
IPR002905	tRNA methyltransferase, Trm1	22	0	0
IPR003102	Coactivator CBP pKID	22	0	0
IPR003130	Dynamin GTPase effector	22	0	0
IPR004360	Glyoxalase/fosfomycin resistance/dioxygenase domain	22	0	0
IPR004457	Zinc finger ZPR1-type	22	0	0
IPR004521	Uncharacterised domain CHP00451	22	0	0

InterPro signature	InterPro description	Shared *Papaipema* genes	*Papaipema sp.4* SSGs	*Papaipema speciosissima* SSGs
IPR004823	TATA box binding protein associated factor TAF	22	0	0
IPR004859	Putative 5-3 exonuclease	22	0	0
IPR004911	Gamma interferon inducible lysosomal thiol reductase GILT	22	0	0
IPR005078	Peptidase C54	22	0	0
IPR005320	Peptidase S51	22	0	0
IPR006164	Ku70/Ku80 beta-barrel domain	22	0	0
IPR006592	RNA polymerase N-terminal	22	0	0
IPR006599	CARP motif	22	0	0
IPR006823	Neutral/alkaline nonlysosomal ceramidase	22	0	0
IPR007080	RNA polymerase Rpb1 domain 1	22	0	0
IPR007238	DNA primase large subunit, eukaryotic/archaeal	22	0	0
IPR008417	B-cell receptor-associated protein 29/31	22	0	0
IPR009050	Globin-like	22	0	0
IPR009077	Proteasome activator pa28	22	0	0
IPR009851	Modifier of rudimentary Modr	22	0	0
IPR010504	Arfaptin homology AH domain	22	0	0
IPR011060	Ribulose-phosphate binding barrel	22	0	0
IPR011656	Notch NODP domain	22	0	0
IPR012926	TMPIT-like	22	0	0
IPR012948	AARP2CN	22	0	0

InterPro signature	InterPro description	Shared *Papaipema* genes	*Papaipema sp.4* SSGs	*Papaipema speciosissima* SSGs
IPR015430	Cyclin T	22	0	0
IPR015807	Histidine-tRNA ligase	22	0	0
IPR015928	Aconitase/3-isopropylmalate dehydratase swivel	22	0	0
IPR017107	Adaptor protein complex AP-1, gamma subunit	22	0	0
IPR017901	C-CAP/cofactor C-like domain	22	0	0
IPR019258	Mediator complex, subunit Med4	22	0	0
IPR019362	Methylmalonic aciduria and homocystinuria type D protein	22	0	0
IPR019542	Enhancer of polycomb-like N-terminal	22	0	0
IPR019585	26S proteasome regulatory subunit Rpn7/COP9 signalosome complex subunit 1	22	0	0
IPR020850	GTPase effector domain	22	0	0
IPR022683	Peptidase C2 calpain domain III	22	0	0
IPR024931	Importin subunit alpha	22	0	0
IPR026063	ATP synthase subunit s, mitochondrial	22	0	0
IPR026254	E3 ubiquitin-protein ligase RNF31	22	0	0
IPR027141	Like-Sm LSM domain containing protein, LSm4/SmD1/SmD3	22	0	0
IPR027986	T-cell activation inhibitor, mitochondrial	22	0	0
IPR028677	Synaptotagmin-10	22	0	0
IPR029257	Rab3GAP regulatory subunit C-terminal	22	0	0
IPR029944	EH domain-binding protein 1	22	0	0
IPR030160	Voltage-dependent calcium channel, T-type, alpha-1H subunit	22	0	0

InterPro signature	InterPro description	Shared *Papaipema* genes	*Papaipema sp.4* SSGs	*Papaipema speciosissima* SSGs
IPR030387	Bms1/Tsr1-type G domain	22	0	0
IPR031329	Neutral/alkaline non-lysosomal ceramidase N-terminal	22	0	0
IPR031937	PNN-interacting serine/arginine-rich protein	22	0	0
IPR032193	CCR4-NOT transcription complex subunit 1 TTP binding domain	22	0	0
IPR032194	CCR4-NOT transcription complex subunit 1 HEAT repeat	22	0	0
IPR032282	Hydroxyacylglutathione hydrolase C-terminal domain	22	0	0
IPR031649	Voltage-dependent L-type calcium channel IQ-associated domain	21	8	3
IPR002020	Citrate synthase	21	3	1
IPR006176	3-hydroxyacyl-CoA dehydrogenase NAD binding	21	3	1
IPR000308	14-3-3 protein	21	0	2
IPR000851	Ribosomal protein S5	21	0	2
IPR005378	Vacuolar protein sorting-associated protein 35	21	0	2
IPR016034	Phosphatidylinositol-4-phosphate 5-kinase core subgroup	21	1	1
IPR019956	Ubiquitin	21	1	1
IPR024909	Cysteinyl-tRNA synthetase/mycothiol ligase	21	2	0
IPR032914	VAM6/VPS39 family	21	0	2
IPR002136	Ribosomal protein L4/L1e	21	0	1
IPR002602	Domain of unknown function DB	21	1	0
IPR003306	WIF domain	21	0	1
IPR004790	Isocitrate dehydrogenase NADP-dependent	21	1	0

InterPro signature	InterPro description	Shared *Papaipema* genes	*Papaipema sp.4* SSGs	*Papaipema speciosissima* SSGs
IPR005141	eRF1 domain 2	21	0	1
IPR005574	RNA polymerase II, Rpb4	21	0	1
IPR005615	Glutathione synthase, eukaryotic	21	0	1
IPR008197	WAP-type 'four-disulfide core' domain	21	1	0
IPR008597	Destabilase	21	1	0
IPR009460	Ryanodine Receptor TM 4-6	21	0	1
IPR011626	Alpha-macroglobulin complement component	21	1	0
IPR012162	Polyribonucleotide nucleotidyltransferase	21	1	0
IPR018731	Autophagy-related protein 13	21	0	1
IPR026811	Cip1-interacting zinc finger protein	21	0	1
IPR028457	ABI family	21	1	0
IPR031866	Domain of unknown function DUF4758	21	1	0
IPR032672	TmcA/NAT10/Kre33	21	1	0
IPR032803	PLD-like domain	21	1	0
IPR000868	Isochorismatase-like	21	0	0
IPR001102	Transglutaminase N-terminal	21	0	0
IPR001117	Multicopper oxidase type 1	21	0	0
IPR001630	cAMP response element binding CREB protein	21	0	0
IPR001645	Folylpolyglutamate synthetase	21	0	0
IPR001728	Thyroid hormone receptor	21	0	0

InterPro signature	InterPro description	Shared *Papaipema* genes	*Papaipema sp.4* SSGs	*Papaipema speciosissima* SSGs
IPR001905	Ammonium transporter	21	0	0
IPR001940	Peptidase S1C	21	0	0
IPR001962	Asparagine synthase	21	0	0
IPR002307	Tyrosine-tRNA ligase	21	0	0
IPR002549	Transmembrane protein TqsA-like	21	0	0
IPR002638	Quinolinate phosphoribosyl transferase C-terminal	21	0	0
IPR002891	Adenylyl-sulfate kinase	21	0	0
IPR002912	ACT domain	21	0	0
IPR003186	Proteasome activator pa28 C-terminal domain	21	0	0
IPR003602	DNA topoisomerase type IA DNA-binding domain	21	0	0
IPR004099	Pyridine nucleotide-disulphide oxidoreductase dimerisation domain	21	0	0
IPR004101	Mur ligase C-terminal	21	0	0
IPR004522	Asparagine-tRNA ligase	21	0	0
IPR005554	Nrap protein	21	0	0
IPR005846	Alpha-D-phosphohexomutase alpha/beta/alpha domain III	21	0	0
IPR005936	Peptidase, FtsH	21	0	0
IPR006175	YjgF/YER057c/UK114 family	21	0	0
IPR006568	PSP proline-rich	21	0	0
IPR007033	RAB6-interacting golgin	21	0	0
IPR007130	Diacylglycerol acyltransferase	21	0	0

**InterPro signature**	**InterPro description**	**Shared *Papaipema* genes**	***Papaipema sp.4* SSGs**	***Papaipema speciosissima* SSGs**
IPR007229	Nicotinate phosphoribosyltransferase family	21	0	0
IPR007379	Tim44-like domain	21	0	0
IPR007604	CP2 transcription factor	21	0	0
IPR007811	DNA-directed RNA polymerase III subunit RPC4	21	0	0
IPR008532	Domain of unknown function DUF814	21	0	0
IPR008733	Peroxisomal biogenesis factor 11	21	0	0
IPR008853	TMEM9	21	0	0
IPR009688	Domain of unknown function DUF1279	21	0	0
IPR010159	N-acyl-L-amino-acid amidohydrolase	21	0	0
IPR010294	ADAM-TS Spacer 1	21	0	0
IPR011489	EMI domain	21	0	0
IPR011513	Non-structural maintenance of chromosomes element 1	21	0	0
IPR011607	Methylglyoxal synthase-like domain	21	0	0
IPR013621	Ion transport N-terminal	21	0	0
IPR013713	Exportin/Importin Cse1-like	21	0	0
IPR015153	EF-hand domain type 1	21	0	0
IPR015403	Sec7 C-terminal	21	0	0
IPR016460	Coatomer beta subunit COPB1)	21	0	0
IPR016635	Adaptor protein complex, sigma subunit	21	0	0
IPR016817	Mannose-P-dolichol utilization defect 1 protein	21	0	0

InterPro signature	InterPro description	Shared *Papaipema* genes	*Papaipema sp.4* SSGs	*Papaipema speciosissima* SSGs
IPR017407	Serine/threonine-protein kinase Rio1	21	0	0
IPR017782	Hydroxyacylglutathione hydrolase	21	0	0
IPR019155	Uncharacterised protein family FPL	21	0	0
IPR021832	Ankyrin repeat domain-containing protein 13	21	0	0
IPR021886	MgsA AAA+ ATPase C-terminal	21	0	0
IPR022189	Smoothelin	21	0	0
IPR022801	Ribosomal protein S4/S9	21	0	0
IPR023591	Ribosomal protein S2 flavodoxin-like domain	21	0	0
IPR023600	Folylpolyglutamate synthase, eukaryota	21	0	0
IPR024041	Ammonium transporter AmtB-like domain	21	0	0
IPR024169	Serine-pyruvate aminotransferase/2-aminoethylphosphonate-pyruvate transaminase	21	0	0
IPR024704	Structural maintenance of chromosomes protein	21	0	0
IPR024951	Sulphate adenylyltransferase catalytic domain	21	0	0
IPR025614	Cell morphogenesis protein N-terminal	21	0	0
IPR025980	ATP-sulfurylase PUA-like domain	21	0	0
IPR027053	Disintegrin and metalloproteinase domain-containing protein 10	21	0	0
IPR030127	I-BAR domain containing protein MIM/ABBA	21	0	0
IPR031334	Piezo non-specific cation channel R-Ras-binding domain	21	0	0
IPR031805	Piezo domain	21	0	0
IPR032376	Dedicator of cytokinesis N-terminal domain	21	0	0

**InterPro signature**	**InterPro description**	**Shared *Papaipema* genes**	***Papaipema sp.4* SSGs**	***Papaipema speciosissima* SSGs**
IPR032678	tRNA synthetases class I catalytic domain	21	0	0
IPR032691	Guanine nucleotide exchange factor N-terminal	21	0	0
IPR000782	FAS1 domain	20	0	6
IPR001790	Ribosomal protein L10P	20	3	0
IPR008210	Phosphoenolpyruvate carboxykinase N-terminal	20	0	3
IPR024743	Dynein heavy chain coiled coil stalk	20	2	1
IPR004345	TB2/DP1/HVA22-related protein	20	1	1
IPR006032	Ribosomal protein S12/S23	20	2	0
IPR012972	NLE	20	1	1
IPR001695	Lysyl oxidase	20	0	1
IPR003169	GYF domain	20	0	1
IPR003673	CoA-transferase family III	20	0	1
IPR004097	DHHA2 domain	20	0	1
IPR004177	DDHD domain	20	0	1
IPR004765	Niemann-Pick C type protein	20	0	1
IPR005314	Peptidase C50, separase	20	1	0
IPR013130	Ferric reductase transmembrane component-like domain	20	1	0
IPR015063	USP8 dimerisation domain	20	0	1
IPR019133	Mitochondrial inner membrane protein Mitofilin	20	1	0
IPR019179	Coiled-coil domain-containing protein 149-A	20	0	1

InterPro signature	InterPro description	Shared *Papaipema* genes	*Papaipema sp.4* SSGs	*Papaipema speciosissima* SSGs
IPR019372	Lipoma HMGIC fusion partner-like protein	20	1	0
IPR019582	RNA recognition motif spliceosomal PrP8	20	1	0
IPR027246	Eukaryotic porin/Tom40	20	0	1
IPR027989	Domain of unknown function DUF4461	20	1	0
IPR028021	Katanin p80 subunit C-terminal	20	0	1
IPR028934	Vacuolar protein sorting protein 26 related	20	1	0
IPR000203	GPS motif	20	0	0
IPR000297	Peptidyl-prolyl cis-trans isomerase PpiC-type	20	0	0
IPR000451	NF-kappa-B/Dorsal	20	0	0
IPR001048	Aspartate/glutamate/uridylate kinase	20	0	0
IPR001283	Cysteine-rich secretory protein, allergen V5/Tpx-1-related	20	0	0
IPR001525	C-5 cytosine methyltransferase	20	0	0
IPR001708	Membrane insertase OXA1/ALB3/YidC	20	0	0
IPR001727	Uncharacterised protein family UPF0016	20	0	0
IPR001865	Ribosomal protein S2	20	0	0
IPR001891	Malic oxidoreductase	20	0	0
IPR002001	GPCR, family 2, diuretic hormone receptor	20	0	0
IPR002044	Carbohydrate binding module family 20	20	0	0
IPR002133	S-adenosylmethionine synthetase	20	0	0
IPR002744	Domain of unknown function DUF59	20	0	0

InterPro signature	InterPro description	Shared *Papaipema* genes	*Papaipema sp.4* SSGs	*Papaipema speciosissima* SSGs
IPR002833	Peptidyl-tRNA hydrolase, PTH2	20	0	0
IPR002857	Zinc finger CXXC-type	20	0	0
IPR003121	SWIB/MDM2 domain	20	0	0
IPR003380	Transforming protein Ski	20	0	0
IPR004006	DhaK domain	20	0	0
IPR004083	Regulatory associated protein of TOR	20	0	0
IPR004506	tRNA-specific 2-thiouridylase	20	0	0
IPR005845	Alpha-D-phosphohexomutase alpha/beta/alpha domain II	20	0	0
IPR006887	Domain of unknown function DUF625	20	0	0
IPR007012	PolyA polymerase central domain	20	0	0
IPR007482	Protein-tyrosine phosphatase-like, PTPLA	20	0	0
IPR007582	TFIID subunit WD40-associated region	20	0	0
IPR007803	Aspartyl/asparaginy/proline hydroxylase	20	0	0
IPR007965	Alpha-tubulin N-acetyltransferase	20	0	0
IPR008045	DNA replication licensing factor Mcm2	20	0	0
IPR008209	Phosphoenolpyruvate carboxykinase, GTP-utilising	20	0	0
IPR008349	Mitogen-activated protein MAP kinase, ERK1/2	20	0	0
IPR009027	Ribosomal protein L9/RNase H1 N-terminal	20	0	0
IPR010304	Survival motor neuron	20	0	0
IPR010400	PITH domain	20	0	0

InterPro signature	InterPro description	Shared *Papaipema* genes	*Papaipema sp.4* SSGs	*Papaipema speciosissima* SSGs
IPR010876	NICE-3 predicted	20	0	0
IPR011268	Purine nucleoside phosphorylase	20	0	0
IPR012921	Spen paralogue and orthologue SPOC C-terminal	20	0	0
IPR012974	NOP5 N-terminal	20	0	0
IPR013121	Ferric reductase NAD binding domain	20	0	0
IPR013221	Mur ligase central	20	0	0
IPR013243	SCA7 domain	20	0	0
IPR013813	Endoribonuclease L-PSP/chorismate mutase-like	20	0	0
IPR014044	CAP domain	20	0	0
IPR014492	PolyA polymerase	20	0	0
IPR015016	Splicing factor 3B subunit 1	20	0	0
IPR015633	E2F Family	20	0	0
IPR018162	Alanine-tRNA ligase class IIc anti-codon-binding domain	20	0	0
IPR018974	Tex-like protein N-terminal	20	0	0
IPR019128	Sister chromatid cohesion protein Dcc1	20	0	0
IPR019369	Protein-lysine N-methyltransferase Efm5	20	0	0
IPR019383	Golgin subfamily A member 7/ERF4	20	0	0
IPR019544	Tetratricopeptide SHNi-TPR domain	20	0	0
IPR020598	Ribosomal RNA adenine methylase transferase N-terminal	20	0	0
IPR022271	Lipocalin, ApoD type	20	0	0

**InterPro signature**	**InterPro description**	**Shared *Papaipema* genes**	***Papaipema sp.4* SSGs**	***Papaipema speciosissima* SSGs**
IPR022636	S-adenosylmethionine synthetase superfamily	20	0	0
IPR023476	Peptidyl-tRNA hydrolase II domain	20	0	0
IPR024810	Mab-21 domain	20	0	0
IPR025258	Putative zinc-RING and/or ribbon	20	0	0
IPR026854	Vacuolar protein sorting-associated protein 13 N-terminal domain	20	0	0
IPR027013	Caskin-1	20	0	0
IPR027486	Ribosomal protein S10 domain	20	0	0
IPR028020	ASX homology domain	20	0	0
IPR031145	Invertebrate aquaporin-10	20	0	0
IPR032346	Transcriptional repressor p66 coiled-coil MBD2-interaction domain	20	0	0
IPR032472	Argonaute linker 2 domain	20	0	0
IPR033047	Dynamin-like 120 kDa protein, mitochondrial	20	0	0
IPR010285	DNA helicase Pif1-like	19	4	1
IPR000235	Ribosomal protein S5/S7	19	3	0
IPR001958	Tetracycline resistance protein TetA/multidrug resistance protein MdtG	19	0	2
IPR007707	Transforming acidic coiled-coil	19	1	1
IPR026172	Gamma-secretase-activating protein family	19	1	1
IPR031176	ELL/occludin family	19	2	0
IPR000704	Casein kinase II, regulatory subunit	19	0	1
IPR001093	IMP dehydrogenase/GMP reductase	19	0	1

InterPro signature	InterPro description	Shared *Papaipema* genes	*Papaipema sp.4* SSGs	*Papaipema speciosissima* SSGs
IPR002656	Acyltransferase 3	19	1	0
IPR005140	eRF1 domain 1/Pelota-like	19	0	1
IPR008954	Moesin tail domain	19	1	0
IPR011128	Glycerol-3-phosphate dehydrogenase NAD-dependent N-terminal	19	1	0
IPR011685	LETM1-like	19	0	1
IPR016090	Phospholipase A2 domain	19	1	0
IPR017426	Nuclear receptor coactivator	19	0	1
IPR018998	Endoribonuclease XendoU	19	1	0
IPR019273	Lunapark domain	19	1	0
IPR000076	K/Cl co-transporter	19	0	0
IPR000897	Signal recognition particle SRP54 subunit GTPase domain	19	0	0
IPR000956	Stathmin family	19	0	0
IPR001063	Ribosomal protein L22/L17	19	0	0
IPR001537	tRNA/rRNA methyltransferase SpoU type	19	0	0
IPR002220	DapA-like	19	0	0
IPR002318	Alanine-tRNA ligase, class IIc	19	0	0
IPR002319	Phenylalanyl-tRNA synthetase	19	0	0
IPR002500	Phosphoadenosine phosphosulphate reductase	19	0	0
IPR002624	Deoxynucleoside kinase	19	0	0
IPR002710	Dilute domain	19	0	0

InterPro signature	InterPro description	Shared *Papaipema* genes	*Papaipema sp.4* SSGs	*Papaipema speciosissima* SSGs
IPR003601	DNA topoisomerase type IA domain 2	19	0	0
IPR003618	Transcription elongation factor S-II central domain	19	0	0
IPR004367	Cyclin C-terminal domain	19	0	0
IPR004842	SLC12A transporter family	19	0	0
IPR005142	eRF1 domain 3	19	0	0
IPR005302	Molybdenum cofactor sulfurase C-terminal	19	0	0
IPR005303	MOSC N-terminal beta barrel	19	0	0
IPR005782	P-type ATPase, subfamily IIA, SERCA-type	19	0	0
IPR005835	Nucleotidyl transferase domain	19	0	0
IPR006109	Glycerol-3-phosphate dehydrogenase NAD-dependent C-terminal	19	0	0
IPR006168	Glycerol-3-phosphate dehydrogenase, NAD-dependent	19	0	0
IPR006405	Nicotinate phosphoribosyltransferase pncB-type	19	0	0
IPR006762	Gtr1/RagA G protein	19	0	0
IPR006768	Cwf19-like C-terminal domain-1	19	0	0
IPR007233	Sybindin-like protein	19	0	0
IPR007244	NatC Nalpha)-terminal acetyltransferase, Mak10 subunit	19	0	0
IPR007599	Derlin	19	0	0
IPR007754	N-acetylglucosaminyltransferase II	19	0	0
IPR008015	GMP phosphodiesterase delta subunit	19	0	0
IPR008089	Nucleotide sugar epimerase	19	0	0

InterPro signature	InterPro description	Shared *Papaipema* genes	*Papaipema sp.4* SSGs	*Papaipema speciosissima* SSGs
IPR008166	Glycosyltransferase family 92	19	0	0
IPR008795	Prominin	19	0	0
IPR009543	Vacuolar protein sorting-associated protein 13 SHR-binding domain	19	0	0
IPR010622	FAST kinase leucine-rich	19	0	0
IPR010911	Rab-binding domain	19	0	0
IPR011893	Selenoprotein, Rdx type	19	0	0
IPR012198	cAMP-dependent protein kinase regulatory subunit	19	0	0
IPR012310	DNA ligase ATP-dependent central	19	0	0
IPR012580	NUC153	19	0	0
IPR013150	Transcription factor TFIIB cyclin-like domain	19	0	0
IPR013272	Vps72/YL1 C-terminal	19	0	0
IPR013566	EF hand associated type-1	19	0	0
IPR013636	Domain of unknown function DUF1741	19	0	0
IPR013719	Domain of unknown function DUF1747	19	0	0
IPR013822	Signal recognition particle SRP54 subunit helical bundle	19	0	0
IPR014009	PIK-related kinase	19	0	0
IPR014012	Helicase/SANT-associated domain	19	0	0
IPR014775	L27 domain C-terminal	19	0	0
IPR014789	PolyA-specific ribonuclease RNA-binding	19	0	0
IPR015395	C-myb C-terminal	19	0	0

InterPro signature	InterPro description	Shared *Papaipema* genes	*Papaipema sp.4* SSGs	*Papaipema speciosissima* SSGs
IPR017331	Peptidoglycan recognition protein, PGRP-S	19	0	0
IPR017751	Glycerol-3-phosphate dehydrogenase, NAD-dependent, eukaryotic	19	0	0
IPR017852	GPI ethanolamine phosphate transferase 1 C-terminal	19	0	0
IPR017890	Transcription elongation factor S-IIM	19	0	0
IPR018022	tRNA dimethylallyltransferase	19	0	0
IPR018155	Hyaluronidase	19	0	0
IPR019156	Ataxin-10 domain	19	0	0
IPR019335	Conserved oligomeric Golgi complex subunit 7	19	0	0
IPR019396	Transmembrane Fragile-X-F-associated protein	19	0	0
IPR019427	7TM GPCR, serpentine receptor class w Srw)	19	0	0
IPR019440	Cohesin loading factor	19	0	0
IPR019447	DNA/RNA-binding protein Kin17 conserved domain	19	0	0
IPR019453	Vacuolar sorting protein 39/Transforming growth factor beta receptor-associated domain 2	19	0	0
IPR019510	Protein kinase A anchor protein nuclear localisation signal domain	19	0	0
IPR019773	Tyrosine 3-monooxygenase-like	19	0	0
IPR020860	MIRO domain	19	0	0
IPR021013	ATPase, vacuolar ER assembly factor, Vma12	19	0	0
IPR021149	Oligosaccharyl transferase complex, subunit OST3/OST6	19	0	0
IPR022309	Ribosomal protein S8e/ribosomal biogenesis NSA2	19	0	0
IPR022409	PKD/Chitinase domain	19	0	0

InterPro signature	InterPro description	Shared *Papaipema* genes	*Papaipema sp.4* SSGs	*Papaipema speciosissima* SSGs
IPR022666	Ribosomal Proteins L2 RNA binding domain	19	0	0
IPR024548	Copper type II ascorbate-dependent monooxygenase C-terminal	19	0	0
IPR025958	SID1 transmembrane family	19	0	0
IPR026028	ATPase, V0 complex, subunit 116kDa, eukaryotic	19	0	0
IPR026053	Hermansky-Pudlak syndrome 1 protein	19	0	0
IPR026092	Retinoic acid-induced protein 2/sine oculis-binding protein homologue	19	0	0
IPR026749	Transmembrane protein 135	19	0	0
IPR026850	FANCL C-terminal domain	19	0	0
IPR027881	Protein SOGA	19	0	0
IPR028271	RNMT-activating mini protein	19	0	0
IPR028288	SCAR/WAVE family	19	0	0
IPR028435	Plakophilin/Delta catenin	19	0	0
IPR028472	Eyes absent family	19	0	0
IPR028501	Endophilin-A	19	0	0
IPR028673	Syntaxin-16	19	0	0
IPR029154	3-hydroxyisobutyrate dehydrogenase NAD-binding domain	19	0	0
IPR029428	FAM195	19	0	0
IPR030374	Polyamine biosynthesis domain	19	0	0
IPR031119	STAR-domain RNA-binding protein GLD-1/HOW	19	0	0
IPR031966	PHD finger protein 12 MRG binding domain	19	0	0

InterPro signature	InterPro description	Shared *Papaipema* genes	*Papaipema sp.4* SSGs	*Papaipema speciosissima* SSGs
IPR032863	Alkylated DNA repair protein alkB homologue 8	19	0	0
IPR029048	Heat shock protein 70kD C-terminal domain	18	1	14
IPR001888	Transposase, type 1	18	1	5
IPR011646	KAP family P-loop domain	18	5	0
IPR028881	PAN2 domain	18	5	0
IPR002403	Cytochrome P450, E-class, group IV	18	3	0
IPR000589	Ribosomal protein S15	18	2	0
IPR001672	Phosphoglucose isomerase PGI)	18	1	1
IPR001873	Na+ channel, amiloride-sensitive	18	0	2
IPR008276	Concentrative nucleoside transporter	18	0	2
IPR000109	Proton-dependent oligopeptide transporter family	18	0	1
IPR001670	Alcohol dehydrogenase iron-type	18	1	0
IPR002420	Phosphatidylinositol 3-kinase C2 domain	18	1	0
IPR006644	Dystroglycan-type cadherin-like	18	1	0
IPR006964	NUDE protein C-terminal	18	1	0
IPR007014	FUN14	18	1	0
IPR007701	Interferon-related developmental regulator N-terminal	18	1	0
IPR008775	Phytanoyl-CoA dioxygenase	18	0	1
IPR010326	Exocyst complex component Sec6	18	0	1
IPR013935	TRAPP II complex, Trs120	18	0	1

**InterPro signature**	**InterPro description**	**Shared *Papaipema* genes**	***Papaipema sp.4* SSGs**	***Papaipema speciosissima* SSGs**
IPR014730	Electron transfer flavoprotein alpha/beta-subunit N-terminal	18	1	0
IPR019149	Abhydrolase domain containing 18	18	0	1
IPR019438	Potential Queuosine, Q, salvage protein family	18	1	0
IPR029772	Tectonin beta-propeller repeat-containing protein 2	18	1	0
IPR000058	Zinc finger AN1-type	18	0	0
IPR000705	Galactokinase	18	0	0
IPR000933	Glycoside hydrolase, family 29	18	0	0
IPR001057	Glutamate/acetylglutamate kinase	18	0	0
IPR001129	Membrane-associated, eicosanoid/glutathione metabolism MAPEG protein	18	0	0
IPR001232	S-phase kinase-associated protein 1-like	18	0	0
IPR001253	Translation initiation factor 1A eIF-1A)	18	0	0
IPR001441	Decaprenyl diphosphate synthase-like	18	0	0
IPR001510	Zinc finger PARP-type	18	0	0
IPR001567	Peptidase M3A/M3B	18	0	0
IPR001912	Ribosomal protein S4/S9 N-terminal	18	0	0
IPR001952	Alkaline phosphatase	18	0	0
IPR002668	Concentrative nucleoside transporter N-terminal domain	18	0	0
IPR003338	CDC48 N-terminal subdomain	18	0	0
IPR003887	LEM domain	18	0	0
IPR003972	Potassium channel, voltage dependent, Kv1	18	0	0

InterPro signature	InterPro description	Shared *Papaipema* genes	*Papaipema sp.4* SSGs	*Papaipema speciosissima* SSGs
IPR004201	CDC48 domain 2	18	0	0
IPR004887	Glutathione synthase substrate-binding eukaryotic	18	0	0
IPR004888	Glycoside hydrolase family 63	18	0	0
IPR005839	Methylthiotransferase	18	0	0
IPR006048	Alpha-amylase/branching enzyme C-terminal all beta	18	0	0
IPR006083	Phosphoribulokinase/uridine kinase	18	0	0
IPR006285	Ubiquitin-like modifier-activating enzyme Atg7	18	0	0
IPR006379	HAD-superfamily hydrolase, subfamily IIB	18	0	0
IPR006545	EYA domain	18	0	0
IPR006561	DZF domain	18	0	0
IPR006615	Peptidase C19 ubiquitin-specific peptidase DUSP domain	18	0	0
IPR006622	Iron sulphur-containing domain CDGSH-type subfamily	18	0	0
IPR006761	Twisted gastrulation Tsg protein	18	0	0
IPR006767	Cwf19-like protein C-terminal domain-2	18	0	0
IPR007007	Ninjurin	18	0	0
IPR007010	PolyA polymerase RNA-binding domain	18	0	0
IPR007042	Arsenite-resistance protein 2	18	0	0
IPR007129	Ubiquinol-cytochrome c chaperone, CBP3	18	0	0
IPR007150	Checkpoint protein Hus1/Mec3	18	0	0
IPR007327	Tumour protein D52	18	0	0

InterPro signature	InterPro description	Shared *Papaipema* genes	*Papaipema sp.4* SSGs	*Papaipema speciosissima* SSGs
IPR007699	SGS domain	18	0	0
IPR007720	N-acetylglucosaminyl transferase component	18	0	0
IPR007768	Suppressor of fused	18	0	0
IPR007862	Adenylate kinase active site lid domain	18	0	0
IPR008646	Herpesvirus UL45-like	18	0	0
IPR009010	Aspartate decarboxylase-like domain	18	0	0
IPR009401	Mediator complex, subunit Med13	18	0	0
IPR009533	Protein of unknown function DUF1151	18	0	0
IPR010414	FRG1-like	18	0	0
IPR010596	Methuselah N-terminal domain	18	0	0
IPR011015	LEM/LEM-like domain	18	0	0
IPR011044	Quinoprotein amine dehydrogenase beta chain-like	18	0	0
IPR011068	Nucleotidyltransferase class I C-terminal-like	18	0	0
IPR011270	Purine nucleoside phosphorylase I, inosine/guanosine-specific	18	0	0
IPR011530	Ribosomal RNA adenine dimethylase	18	0	0
IPR011625	Alpha-2-macroglobulin N-terminal 2	18	0	0
IPR012913	Glucosidase II beta subunit-like	18	0	0
IPR012975	NOPS	18	0	0
IPR013555	Transient receptor ion channel domain	18	0	0
IPR013567	EF hand associated type-2	18	0	0

InterPro signature	InterPro description	Shared *Papaipema* genes	*Papaipema sp.4* SSGs	*Papaipema speciosissima* SSGs
IPR013625	Tensin phosphotyrosine-binding domain	18	0	0
IPR013848	Methylthiotransferase N-terminal	18	0	0
IPR014719	Ribosomal protein L7/L12 C-terminal/adaptor protein ClpS-like	18	0	0
IPR014908	Nucleoporin Nup133/Nup155-like N-terminal	18	0	0
IPR014912	Sep15/SelM redox	18	0	0
IPR015404	Sorting nexin Vps5-like C-terminal	18	0	0
IPR015517	Deoxycytidylate deaminase-related	18	0	0
IPR016073	SKP1 component POZ domain	18	0	0
IPR017096	BTB-kelch protein	18	0	0
IPR018307	AVL9/DENND6 domain	18	0	0
IPR018392	LysM domain	18	0	0
IPR018967	Iron sulphur-containing domain CDGSH-type	18	0	0
IPR019318	Guanine nucleotide exchange factor, Ric8	18	0	0
IPR019458	Telomerase activating protein Est1	18	0	0
IPR019539	Galactokinase galactose-binding domain	18	0	0
IPR019729	Gloverin-like protein	18	0	0
IPR020405	Atypical dual specificity phosphatase, subfamily A	18	0	0
IPR020417	Atypical dual specificity phosphatase	18	0	0
IPR020795	Origin recognition complex, subunit 3	18	0	0
IPR021150	Ubiquinol-cytochrome c chaperone/UPF0174	18	0	0

InterPro signature	InterPro description	Shared *Papaipema* genes	*Papaipema sp.4* SSGs	*Papaipema speciosissima* SSGs
IPR021925	Protein of unknown function DUF3538	18	0	0
IPR022591	Transcription initiation factor TFIID subunit 1 domain of unknown function	18	0	0
IPR023362	PH-BEACH domain	18	0	0
IPR024104	Pseudokinase tribbles family/serine-threonine-protein kinase 40	18	0	0
IPR024129	Sphingomyelin phosphodiesterase 4	18	0	0
IPR024642	SUZ-C domain	18	0	0
IPR024678	Serine/threonine-protein kinase OSR1/WNK CCT domain	18	0	0
IPR026069	Fuzzy protein	18	0	0
IPR026319	Zinc finger C2HC domain-containing protein	18	0	0
IPR026700	Coiled-coil domain-containing protein 142	18	0	0
IPR027065	Lon protease	18	0	0
IPR027659	Beta-sarcoglycan	18	0	0
IPR029005	LIM-domain binding protein/SEUSS	18	0	0
IPR029559	Membrane protein Tms1-like	18	0	0
IPR030174	LIM domain-binding protein 2	18	0	0
IPR032176	Domain of unknown function DUF5009	18	0	0
IPR032337	Cell-cycle alteration and expression-elevated protein in tumour	18	0	0
IPR032415	TRPM tetramerisation domain	18	0	0
IPR032839	Rab3-GAP regulatory subunit N-terminal	18	0	0
IPR032847	Pre-mRNA-processing factor 17	18	0	0

InterPro signature	InterPro description	Shared *Papaipema* genes	*Papaipema sp.4* SSGs	*Papaipema speciosissima* SSGs
IPR032857	Alpha-ketoglutarate-dependent dioxygenase alkB homologue 4	18	0	0
IPR032974	Polyprenol kinase family	18	0	0
IPR033122	Letm1 ribosome-binding domain	18	0	0
IPR024939	Calcium-activated potassium channel Slo	17	2	6
IPR008274	Aldehyde oxidase/xanthine dehydrogenase molybdopterin binding	17	0	7
IPR004273	Dynein heavy chain domain	17	1	3
IPR021935	Domain of unknown function DUF3548	17	2	2
IPR032718	PiggyBac transposable element-derived protein 4 C-terminal zinc-ribbon	17	3	1
IPR029705	UPF0505 family	17	0	3
IPR000611	Neuropeptide Y receptor family	17	2	0
IPR000988	Ribosomal protein L24e-related	17	1	1
IPR001985	S-adenosylmethionine decarboxylase	17	1	1
IPR012496	TMC	17	2	0
IPR013025	Ribosomal protein L25/L23	17	1	1
IPR016067	S-adenosylmethionine decarboxylase core	17	1	1
IPR031106	CCAAT/enhancer-binding protein C/EBP	17	1	1
IPR000744	NSF attachment protein	17	1	0
IPR001599	Alpha-2-macroglobulin	17	0	1
IPR005011	SNU66/SART1 family	17	0	1
IPR006994	Transcription factor 25	17	1	0

**InterPro signature**	**InterPro description**	**Shared *Papaipema* genes**	***Papaipema sp.4* SSGs**	***Papaipema speciosissima* SSGs**
IPR011017	TRASH domain	17	1	0
IPR012308	DNA ligase ATP-dependent N-terminal	17	1	0
IPR012720	T-complex protein 1, eta subunit	17	1	0
IPR013791	RNA 3'-terminal phosphate cyclase insert domain	17	0	1
IPR017862	SKI-interacting protein, SKIP	17	0	1
IPR020479	Homeodomain metazoa	17	1	0
IPR022033	RAVE complex protein Rav1 C-terminal	17	1	0
IPR025224	Cell cycle and apoptosis regulator protein	17	1	0
IPR025697	CLU domain	17	1	0
IPR026705	Hid-1/Ecm30	17	1	0
IPR030398	DG-type SEA domain	17	1	0
IPR032423	AAA ATPase C-terminal domain	17	1	0
IPR000163	Prohibitin	17	0	0
IPR000407	Nucleoside phosphatase GDA1/CD39	17	0	0
IPR000587	Creatinase N-terminal	17	0	0
IPR001040	Translation Initiation factor eIF- 4e	17	0	0
IPR001085	Serine hydroxymethyltransferase	17	0	0
IPR001171	Ergosterol biosynthesis ERG4/ERG24	17	0	0
IPR001329	Glycoside hydrolase family 56, bee venom hyaluronidase	17	0	0
IPR001544	Aminotransferase class IV	17	0	0

InterPro signature	InterPro description	Shared *Papaipema* genes	*Papaipema sp.4* SSGs	*Papaipema speciosissima* SSGs
IPR001593	Ribosomal protein S3Ae	17	0	0
IPR001937	Galactose-1-phosphate uridyl transferase, class I	17	0	0
IPR001945	RAD3/XPD family	17	0	0
IPR001971	Ribosomal protein S11	17	0	0
IPR001997	Calponin/LIMCH1	17	0	0
IPR002121	HRDC domain	17	0	0
IPR002202	Hydroxymethylglutaryl-CoA reductase, class I/II	17	0	0
IPR002930	Glycine cleavage system H-protein	17	0	0
IPR003021	Rad1/Rec1/Rad17	17	0	0
IPR003111	ATP-dependent protease La LON substrate-binding domain	17	0	0
IPR003172	MD-2-related lipid-recognition domain	17	0	0
IPR004139	Glycosyl transferase, family 13	17	0	0
IPR004480	Monothiol glutaredoxin-related	17	0	0
IPR004567	Type II pantothenate kinase	17	0	0
IPR004579	ERCC1/RAD10/SWI10 family	17	0	0
IPR005066	Moybdenum cofactor oxidoreductase dimerisation	17	0	0
IPR005793	Formyl transferase C-terminal	17	0	0
IPR005849	Galactose-1-phosphate uridyl transferase N-terminal	17	0	0
IPR006207	Cystine knot C-terminal	17	0	0
IPR006259	Adenylate kinase subfamily	17	0	0

InterPro signature	InterPro description	Shared *Papaipema* genes	*Papaipema sp.4* SSGs	*Papaipema speciosissima* SSGs
IPR006331	Adenosine deaminase-related growth factor	17	0	0
IPR006567	PUG domain	17	0	0
IPR007019	Surfeit locus 6	17	0	0
IPR007148	Small-subunit processome Utp12	17	0	0
IPR007209	RNase L inhibitor RLI possible metal-binding domain	17	0	0
IPR007504	H/ACA ribonucleoprotein complex, subunit Gar1/Naf1	17	0	0
IPR007758	Nucleoporin NSP1-like C-terminal	17	0	0
IPR008388	ATPase, V1 complex, subunit S1	17	0	0
IPR008837	Serendipity locus alpha	17	0	0
IPR009028	Coatomer/calthrin adaptor appendage C-terminal subdomain	17	0	0
IPR009067	TAFII-230 TBP-binding	17	0	0
IPR010286	Ribosomal RNA large subunit methyltransferase F-like	17	0	0
IPR010507	Zinc finger MYM-type	17	0	0
IPR010770	Ecd family	17	0	0
IPR011034	Formyl transferase C-terminal-like	17	0	0
IPR011320	Ribonuclease H1 N-terminal	17	0	0
IPR011644	Heme-NO binding	17	0	0
IPR011948	Dullard phosphatase domain eukaryotic	17	0	0
IPR012302	Malic enzyme NAD-binding	17	0	0
IPR012309	DNA ligase ATP-dependent C-terminal	17	0	0

InterPro signature	InterPro description	Shared *Papaipema* genes	*Papaipema sp.4* SSGs	*Papaipema speciosissima* SSGs
IPR013112	FAD-binding 8	17	0	0
IPR013283	ABC transporter ABCE	17	0	0
IPR013562	tRNAMet cytidine acetyltransferase TmcA N-terminal	17	0	0
IPR014311	Guanine deaminase	17	0	0
IPR016286	Alpha-L-fucosidase, metazoa-type	17	0	0
IPR016346	Guanine nucleotide-binding protein, beta subunit	17	0	0
IPR016467	DNA recombination and repair protein, RecA-like	17	0	0
IPR016580	Cell cycle checkpoint, Hus1	17	0	0
IPR017243	Biogenesis of lysosome-related organelles complex 1 subunit 5	17	0	0
IPR019007	WW domain binding protein 11	17	0	0
IPR019087	Mediator complex, subunit Med15	17	0	0
IPR019180	Oxidoreductase-like N-terminal	17	0	0
IPR019393	WASH complex, subunit strumpellin	17	0	0
IPR019395	Transmembrane protein 161A/B	17	0	0
IPR019532	Nuclear RNA-splicing-associated protein, SR-25	17	0	0
IPR019572	Ubiquitin-activating enzyme catalytic cysteine domain	17	0	0
IPR021900	Protein of unknown function DUF3512	17	0	0
IPR022052	Histone-binding protein RBBP4 N-terminal	17	0	0
IPR022352	Insulin family	17	0	0
IPR022630	S-adenosylmethionine synthetase C-terminal	17	0	0

InterPro signature	InterPro description	Shared *Papaipema* genes	*Papaipema sp.4* SSGs	*Papaipema speciosissima* SSGs
IPR023231	GSKIP domain	17	0	0
IPR023398	Translation Initiation factor eIF- 4e-like	17	0	0
IPR024193	Ku80	17	0	0
IPR024511	WD repeat-containing and planar cell polarity effector protein Fritz	17	0	0
IPR024768	Meiosis arrest female protein 1	17	0	0
IPR025136	Domain of unknown function DUF4071	17	0	0
IPR025696	rRNA-processing arch domain	17	0	0
IPR025816	RrmJ-type ribose 2-O-methyltransferase domain	17	0	0
IPR026010	Nucleoporin NSP1/NUP62	17	0	0
IPR026270	Signal recognition particle, SRP72 subunit	17	0	0
IPR026683	Serine/threonine-protein kinase TOR	17	0	0
IPR026736	Protein virilizer	17	0	0
IPR026874	Glucosidase 2 subunit beta	17	0	0
IPR027062	Carboxypeptidase M	17	0	0
IPR027080	Uncoordinated protein 13 Unc-13)	17	0	0
IPR027094	Mitofusin family	17	0	0
IPR028146	Glucosidase II beta subunit N-terminal	17	0	0
IPR028261	Dihydroprymidine dehydrogenase domain II	17	0	0
IPR029190	Ribosomal RNA-processing protein 14/surfeit locus protein 6 C-terminal domain	17	0	0
IPR029325	IP3R-interacting domain	17	0	0

InterPro signature	InterPro description	Shared *Papaipema* genes	*Papaipema sp.4* SSGs	*Papaipema speciosissima* SSGs
IPR029909	Actin-related protein 1 family	17	0	0
IPR030397	SEPARIN core domain	17	0	0
IPR031645	Vacuolar protein sorting-associated protein 13 C-terminal	17	0	0
IPR031658	Cyclin C-terminal domain 2	17	0	0
IPR032197	Ubiquitin-like modifier-activating enzyme Atg7 N-terminal	17	0	0
IPR032377	STAR protein homodimerisation region	17	0	0
IPR032835	Rho GTPase-activating protein FF domain	17	0	0
IPR032862	Alpha-ketoglutarate-dependent dioxygenase alkB homologue 6	17	0	0
IPR003929	Potassium channel, calcium-activated, BK, alpha subunit	16	8	5
IPR005446	Voltage-dependent calcium channel, L-type, alpha-1 subunit	16	1	6
IPR000928	SNAP-25	16	3	0
IPR002579	Peptide methionine sulphoxide reductase MrsB	16	1	2
IPR006773	26S proteasome complex ubiquitin receptor, subunit Rpn13	16	1	2
IPR019452	Vacuolar sorting protein 39/Transforming growth factor beta receptor-associated domain 1	16	1	1
IPR000262	FMN-dependent dehydrogenase	16	1	0
IPR000372	Leucine-rich repeat N-terminal domain	16	1	0
IPR000674	Aldehyde oxidase/xanthine dehydrogenase a/b hammerhead	16	0	1
IPR006046	Alpha amylase	16	1	0
IPR006590	RNA polymerase II Rpb4 core	16	1	0
IPR008806	RNA polymerase III Rpc82 C -terminal	16	1	0

**InterPro signature**	**InterPro description**	**Shared *Papaipema* genes**	***Papaipema sp.4* SSGs**	***Papaipema speciosissima* SSGs**
IPR009617	Seipin family	16	0	1
IPR011332	Zinc-binding ribosomal protein	16	0	1
IPR012133	Alpha-hydroxy acid dehydrogenase, FMN-dependent	16	0	1
IPR012462	Peptidase C78, ubiquitin fold modifier-specific peptidase 1/2	16	0	1
IPR012493	Renin receptor-like	16	1	0
IPR019355	Cell cycle regulator Mat89Bb	16	1	0
IPR022226	Protein of unknown function DUF3752	16	1	0
IPR023606	CoA-transferase family III domain	16	1	0
IPR028054	Protein of unknown function DUF4481	16	1	0
IPR032029	ADAM17 membrane-proximal domain	16	1	0
IPR032815	Peptidase S8 pro-domain	16	1	0
IPR000011	Ubiquitin/SUMO-activating enzyme E1	16	0	0
IPR000245	V-ATPase proteolipid subunit	16	0	0
IPR000741	Fructose-bisphosphate aldolase, class-I	16	0	0
IPR000997	Cholinesterase	16	0	0
IPR001024	PLAT/LH2 domain	16	0	0
IPR001033	Alpha-catenin	16	0	0
IPR001044	XPG/Rad2 endonuclease, eukaryotes	16	0	0
IPR001414	G-protein coupled receptor 143	16	0	0
IPR001805	Adenosine kinase	16	0	0

InterPro signature	InterPro description	Shared *Papaipema* genes	*Papaipema sp.4* SSGs	*Papaipema speciosissima* SSGs
IPR002060	Squalene/phytoene synthase	16	0	0
IPR002470	Peptidase S9A, prolyl oligopeptidase	16	0	0
IPR002509	NodB homology domain	16	0	0
IPR003117	cAMP-dependent protein kinase regulatory subunit dimerization-anchoring domain	16	0	0
IPR003153	Adaptor protein Cbl N-terminal helical	16	0	0
IPR003350	CUT domain	16	0	0
IPR004108	Iron hydrogenase large subunit C-terminal	16	0	0
IPR004198	Zinc finger C5HC2-type	16	0	0
IPR004443	YjeF N-terminal domain	16	0	0
IPR004803	Queuine tRNA-ribosyltransferase	16	0	0
IPR004895	Prenylated rab acceptor PRA1	16	0	0
IPR004981	Tryptophan 2,3-dioxygenase	16	0	0
IPR005161	Ku70/Ku80 N-terminal alpha/beta	16	0	0
IPR005172	CRC domain	16	0	0
IPR005248	Probable nicotinate-nucleotide adenylyltransferase	16	0	0
IPR005429	Lysosome membrane protein II	16	0	0
IPR005617	Groucho/TLE N-terminal Q-rich domain	16	0	0
IPR005792	Protein disulphide isomerase	16	0	0
IPR006169	GTP1/OBG domain	16	0	0
IPR006408	P-type ATPase, subfamily IIB	16	0	0

InterPro signature	InterPro description	Shared *Papaipema* genes	*Papaipema sp.4* SSGs	*Papaipema speciosissima* SSGs
IPR006640	SprT-like	16	0	0
IPR006642	Zinc finger Rad18-type putative	16	0	0
IPR006676	tRNA-splicing endonuclease	16	0	0
IPR006722	Trafficking protein particle complex subunit 2	16	0	0
IPR007207	CCR4-Not complex component Not N-terminal domain	16	0	0
IPR007245	GPI transamidase component PIG-T	16	0	0
IPR007246	GPI transamidase component Gaa1	16	0	0
IPR007255	Conserved oligomeric Golgi complex subunit 8	16	0	0
IPR007266	Endoplasmic reticulum oxidoreductin 1	16	0	0
IPR007268	Rad9/Ddc1	16	0	0
IPR007852	Cdc73/Parafibromin	16	0	0
IPR008050	DNA replication licensing factor Mcm7	16	0	0
IPR008269	Peptidase S16 Lon C-terminal	16	0	0
IPR008367	Regucalcin	16	0	0
IPR008429	Cleft lip and palate transmembrane 1	16	0	0
IPR008603	Dynactin subunit 4	16	0	0
IPR008851	Transcription initiation factor IIF, alpha subunit	16	0	0
IPR009016	Iron hydrogenase	16	0	0
IPR009210	Activating signal cointegrator 1 complex subunit 1	16	0	0
IPR009398	Adenylate cyclase conserved domain	16	0	0

**InterPro signature**	**InterPro description**	**Shared *Papaipema* genes**	***Papaipema sp.4* SSGs**	***Papaipema speciosissima* SSGs**
IPR009454	Lipid transport open beta-sheet	16	0	0
IPR010635	Heparan sulphate 6-sulfotransferase/Protein-tyrosine sulfotransferase	16	0	0
IPR010926	Class I myosin tail homology domain	16	0	0
IPR011184	DNA mismatch repair MutS	16	0	0
IPR011419	ATP12, ATPase F1F0-assembly protein	16	0	0
IPR011687	Ribosome biogenesis protein Nop53/GLTSCR2	16	0	0
IPR012001	Thiamine pyrophosphate enzyme N-terminal TPP-binding domain	16	0	0
IPR012020	AB hydrolase 4 family	16	0	0
IPR012849	Abl-interactor homeo-domain homologous domain	16	0	0
IPR013194	Histone deacetylase interacting domain	16	0	0
IPR013233	Glycosylphosphatidylinositol-mannosyltransferase I, PIG-X/PBN1	16	0	0
IPR013537	Acetyl-CoA carboxylase central domain	16	0	0
IPR013548	Plexin cytoplasmic RasGAP domain	16	0	0
IPR013627	DNA polymerase alpha subunit B N-terminal	16	0	0
IPR014052	DNA primase, small subunit, eukaryotic/archaeal	16	0	0
IPR014741	Adaptor protein Cbl EF hand-like	16	0	0
IPR014742	Adaptor protein Cbl SH2-like	16	0	0
IPR014770	Munc13 homology 1	16	0	0
IPR014772	Mammalian uncoordinated homology 13 domain 2	16	0	0
IPR014889	Transcription factor DP C-terminal	16	0	0

**InterPro signature**	**InterPro description**	**Shared *Papaipema* genes**	***Papaipema sp.4* SSGs**	***Papaipema speciosissima* SSGs**
IPR015154	EF-hand domain type 2	16	0	0
IPR015285	RIO2 kinase winged helix domain N-terminal	16	0	0
IPR015418	Chromatin modification-related protein Eaf6	16	0	0
IPR015527	Peptidase C26, gamma-glutamyl hydrolase	16	0	0
IPR015648	Transcription factor DP	16	0	0
IPR016292	Epoxide hydrolase	16	0	0
IPR016655	Prefoldin subunit 3	16	0	0
IPR017714	Methylthioribulose-1-phosphate dehydratase	16	0	0
IPR017921	Zinc finger CTCHY-type	16	0	0
IPR018149	Lysyl-tRNA synthetase class II C-terminal	16	0	0
IPR019148	Nuclear protein DGCR14	16	0	0
IPR019442	Domain of unknown function DUF2428 death-receptor-like	16	0	0
IPR019515	Vacuolar protein sorting-associated protein 54 N-terminal	16	0	0
IPR019835	SWIB domain	16	0	0
IPR021181	Mitochondrial Rho GTPase	16	0	0
IPR021785	Protein of unknown function DUF3350	16	0	0
IPR021933	Protein of unknown function DUF3546	16	0	0
IPR022049	FAM69 protein-kinase domain	16	0	0
IPR022096	SBF1/SBF2 domain	16	0	0
IPR022207	Genetic suppressor element-like	16	0	0

InterPro signature	InterPro description	Shared *Papaipema* genes	*Papaipema sp.4* SSGs	*Papaipema speciosissima* SSGs
IPR022628	S-adenosylmethionine synthetase N-terminal	16	0	0
IPR022629	S-adenosylmethionine synthetase central domain	16	0	0
IPR022750	Interferon regulatory factor 2-binding protein 1 & 2 zinc finger	16	0	0
IPR023321	PINIT domain	16	0	0
IPR024088	Tyrosine-tRNA ligase, bacterial-type	16	0	0
IPR024159	Adaptor protein Cbl PTB domain	16	0	0
IPR024162	Adaptor protein Cbl	16	0	0
IPR024253	Phosducin thioredoxin-like domain	16	0	0
IPR024874	Transcription factor Maf	16	0	0
IPR025927	Potential DNA-binding domain	16	0	0
IPR025934	NudC N-terminal domain	16	0	0
IPR026156	Folliculin-interacting protein family	16	0	0
IPR026544	Smoothened	16	0	0
IPR026873	Geranylgeranyl transferase type-2 subunit beta	16	0	0
IPR027109	SWR1-complex protein 4/DNA methyltransferase 1-associated protein 1	16	0	0
IPR027226	E3 SUMO-protein ligase PIAS3	16	0	0
IPR028019	Protein of unknown function DUF4508	16	0	0
IPR028103	Spatacsin	16	0	0
IPR028307	Lin-54 family	16	0	0
IPR028935	WHIM3 domain	16	0	0

InterPro signature	InterPro description	Shared *Papaipema* genes	*Papaipema sp.4* SSGs	*Papaipema speciosissima* SSGs
IPR029277	Single domain Von Willebrand factor type C domain	16	0	0
IPR029409	Transmembrane protein 237	16	0	0
IPR030030	Scaffold protein salvador	16	0	0
IPR030082	Neprilysin-21	16	0	0
IPR030164	Translocator protein	16	0	0
IPR030484	Serine/threonine-protein kinase Rio2	16	0	0
IPR031121	KH domain containing protein	16	0	0
IPR031148	Plexin family	16	0	0
IPR031436	Transmembrane protein TMEM132 C-terminal	16	0	0
IPR031814	ALG11 mannosyltransferase N-terminal	16	0	0
IPR031824	E3 ubiquitin-protein ligase RNF220 middle domain	16	0	0
IPR032076	Tetratricopeptide repeat protein 5 OB fold domain	16	0	0
IPR032396	Spindle assembly abnormal protein 6 N-terminal	16	0	0
IPR032639	Tex protein YqgF-like domain	16	0	0
IPR033162	Tubulin-folding cofactor D	16	0	0
IPR000878	Tetrapyrrole methylase	15	3	0
IPR004551	Diphthine synthase	15	0	3
IPR010977	Aromatic-L-amino-acid decarboxylase	15	3	0
IPR015813	Pyruvate/Phosphoenolpyruvate kinase-like domain	15	0	3
IPR029065	Enolase C-terminal domain-like	15	0	3

InterPro signature	InterPro description	Shared *Papaipema* genes	*Papaipema sp.4* SSGs	*Papaipema speciosissima* SSGs
IPR003005	Amphiphysin	15	0	2
IPR003358	tRNA guanine-N-7 methyltransferase, Trmb type	15	1	1
IPR006581	VPS10	15	2	0
IPR010164	Ornithine aminotransferase	15	1	1
IPR010442	PET domain	15	1	1
IPR011496	Beta-N-acetylglucosaminidase	15	2	0
IPR016270	Phospholipase D, phosphatidylserine synthase type	15	1	1
IPR023410	14-3-3 domain	15	2	0
IPR027106	U4/U6 small nuclear ribonucleoprotein Prp4	15	1	1
IPR031777	Sortilin C-terminal	15	2	0
IPR031778	Sortilin N-terminal	15	2	0
IPR003973	Potassium channel, voltage dependent, Kv2	15	0	1
IPR005822	Ribosomal protein L13	15	1	0
IPR006565	Bromodomain associated domain	15	0	1
IPR012591	PRO8NT domain	15	1	0
IPR012592	PROCN domain	15	1	0
IPR013680	Voltage-dependent calcium channel alpha-2/delta subunit conserved region	15	0	1
IPR014830	Glycolipid transfer protein domain	15	0	1
IPR020560	Phosphoribosylglycinamide synthetase C-domain	15	0	1
IPR020829	Glyceraldehyde 3-phosphate dehydrogenase catalytic domain	15	0	1

InterPro signature	InterPro description	Shared *Papaipema* genes	*Papaipema sp.4* SSGs	*Papaipema speciosissima* SSGs
IPR020831	Glyceraldehyde/Erythrose phosphate dehydrogenase family	15	1	0
IPR026914	Calsyntenin	15	1	0
IPR029017	Enolase N-terminal domain-like	15	0	1
IPR030045	Alpha-catulin 1	15	1	0
IPR031717	Multifunctional 2-oxoglutarate metabolism enzyme C-terminal	15	0	1
IPR033133	Pumilio homology domain	15	1	0
IPR000074	Apolipoprotein A/E	15	0	0
IPR000559	Formate-tetrahydrofolate ligase, FTHFS	15	0	0
IPR000754	Ribosomal protein S9	15	0	0
IPR000801	Putative esterase	15	0	0
IPR001111	Transforming growth factor-beta N-terminal	15	0	0
IPR001354	Mandelate racemase/muconate lactonizing enzyme/methylaspartate ammonia-lyase	15	0	0
IPR001529	DNA-directed RNA polymerase M/15kDa subunit	15	0	0
IPR001656	Pseudouridine synthase, TruD	15	0	0
IPR001697	Pyruvate kinase	15	0	0
IPR001816	Translation elongation factor EFTs/EF1B	15	0	0
IPR002051	Haem oxygenase	15	0	0
IPR002223	Pancreatic trypsin inhibitor Kunitz domain	15	0	0
IPR002313	Lysine-tRNA ligase, class II	15	0	0
IPR002654	Glycosyl transferase, family 25	15	0	0

InterPro signature	InterPro description	Shared *Papaipema* genes	*Papaipema sp.4* SSGs	*Papaipema speciosissima* SSGs
IPR002755	DNA primase, small subunit	15	0	0
IPR002816	Pheromone shutdown, TraB	15	0	0
IPR002903	Ribosomal RNA small subunit methyltransferase H	15	0	0
IPR003020	Bicarbonate transporter, eukaryotic	15	0	0
IPR003888	FY-rich N-terminal	15	0	0
IPR003893	Iroquois-class homeodomain protein	15	0	0
IPR004523	Aspartyl-tRNA synthetase, archaeal-type	15	0	0
IPR004552	1-acyl-sn-glycerol-3-phosphate acyltransferase	15	0	0
IPR004733	Phosphoribosylformylglycinamidine cyclo-ligase	15	0	0
IPR004802	tRNA pseudouridine synthase B family	15	0	0
IPR004836	Sodium/calcium exchanger protein	15	0	0
IPR005204	Hemocyanin N-terminal	15	0	0
IPR005365	Nitrogen permease regulator 3	15	0	0
IPR005576	RNA polymerase Rpb7 N-terminal	15	0	0
IPR005841	Alpha-D-phosphohexomutase superfamily	15	0	0
IPR005850	Galactose-1-phosphate uridyl transferase C-terminal	15	0	0
IPR005935	Diphosphomevalonate/phosphomevalonate decarboxylase	15	0	0
IPR006121	Heavy metal-associated domain HMA	15	0	0
IPR006696	Protein of unknown function DUF423	15	0	0
IPR006709	Small-subunit processome, Utp14	15	0	0

InterPro signature	InterPro description	Shared *Papaipema* genes	*Papaipema sp.4* SSGs	*Papaipema speciosissima* SSGs
IPR006886	DNA-directed RNA polymerase III subunit Rpc5	15	0	0
IPR007220	Origin recognition complex, subunit 2	15	0	0
IPR007276	Nucleolar protein 14	15	0	0
IPR007781	Alpha-N-acetylglucosaminidase	15	0	0
IPR008049	DNA replication licensing factor Mcm6	15	0	0
IPR008075	Lipocalin-1 receptor	15	0	0
IPR008631	Glycogen synthase	15	0	0
IPR008672	Spindle assembly checkpoint component Mad1	15	0	0
IPR009262	Solute carrier family 35 member SLC35F1/F2/F6	15	0	0
IPR009294	Gamma-secretase subunit Aph-1	15	0	0
IPR009828	Protein of unknown function DUF1394	15	0	0
IPR009976	Exocyst complex component Sec10-like	15	0	0
IPR010059	Uridine phosphorylase, eukaryotic	15	0	0
IPR010548	BNIP3	15	0	0
IPR010796	B9 domain	15	0	0
IPR010849	Gonadal family	15	0	0
IPR011035	Ribosomal protein L25/Gln-tRNA synthetase anti-codon-binding domain	15	0	0
IPR011531	Bicarbonate transporter C-terminal	15	0	0
IPR011710	Coatomer beta subunit C-terminal	15	0	0
IPR012301	Malic enzyme N-terminal domain	15	0	0

**InterPro signature**	**InterPro description**	**Shared *Papaipema* genes**	***Papaipema sp.4* SSGs**	***Papaipema speciosissima* SSGs**
IPR012960	Dyskerin-like	15	0	0
IPR012961	ATP-dependent RNA helicase Ski2 C-terminal	15	0	0
IPR013219	Ribosomal protein S27/S33, mitochondrial	15	0	0
IPR013578	Peptidase M16C associated	15	0	0
IPR013725	DNA replication factor RFC1 C-terminal	15	0	0
IPR013769	Band 3 cytoplasmic domain	15	0	0
IPR013893	Ribonuclease P, Rpp40	15	0	0
IPR014039	Translation elongation factor EFTs/EF1B dimerisation	15	0	0
IPR014186	S-formylglutathione hydrolase	15	0	0
IPR014812	Vacuolar protein sorting-associated protein 51	15	0	0
IPR014853	Uncharacterised domain cysteine-rich	15	0	0
IPR015211	Peptidase M1 leukotriene A4 hydrolase aminopeptidase C-terminal	15	0	0
IPR015525	Breast cancer type 2 susceptibility protein	15	0	0
IPR015793	Pyruvate kinase barrel	15	0	0
IPR016053	Haem oxygenase-like	15	0	0
IPR016084	Haem oxygenase-like multi-helical	15	0	0
IPR016528	Vacuolar protein sorting-associated protein 11	15	0	0
IPR016534	Vacuolar protein sorting-associated protein 16	15	0	0
IPR016697	Aquaporin 11/12	15	0	0
IPR017423	tRNA adenine58)-N1))-methyltransferase non-catalytic subunit TRM6	15	0	0

InterPro signature	InterPro description	Shared *Papaipema* genes	*Papaipema sp.4* SSGs	*Papaipema speciosissima* SSGs
IPR017770	RNA 3'-terminal phosphate cyclase type 1	15	0	0
IPR018203	GDP dissociation inhibitor	15	0	0
IPR018593	tRNA-splicing endonuclease subunit Sen15	15	0	0
IPR018797	Uncharacterised protein family FAM98	15	0	0
IPR019129	Folate-sensitive fragile site protein Fra10Ac1	15	0	0
IPR019154	Arb2 domain	15	0	0
IPR019159	Coiled-coil domain of unkwon function DUF2037	15	0	0
IPR019166	Apolipoprotein O//MICOS complex subunit Mic27	15	0	0
IPR019172	Osteopetrosis-associated transmembrane protein 1 precursor	15	0	0
IPR019311	Protein of unknown function DUF2362	15	0	0
IPR019465	Conserved oligomeric Golgi complex subunit 5	15	0	0
IPR019523	Protein phosphatase 1 regulatory subunit 15A/B C-terminal	15	0	0
IPR020059	Glutamyl/glutaminyl-tRNA synthetase class Ib anti-codon binding domain	15	0	0
IPR020519	Uncharacterised protein family UPF0672	15	0	0
IPR021429	Mediator complex subunit Med24 N-terminal	15	0	0
IPR022042	snRNA-activating protein complex, subunit 3	15	0	0
IPR022192	Mitochondrial degradasome RNA helicase subunit C-terminal domain	15	0	0
IPR022229	Peptidase S8A tripeptidyl peptidase II	15	0	0
IPR022783	GC-rich sequence DNA-binding factor domain	15	0	0
IPR023270	tRNA C5-cytosine methyltransferase, NCL1	15	0	0

**InterPro signature**	**InterPro description**	**Shared *Papaipema* genes**	***Papaipema sp.4* SSGs**	***Papaipema speciosissima* SSGs**
IPR024861	Donson	15	0	0
IPR024864	Nucleoporin Nup54/Nup57/Nup44	15	0	0
IPR025260	Domain of unknown function DUF4208	15	0	0
IPR025609	Lsm14 N-terminal	15	0	0
IPR026291	G patch domain-containing protein 2	15	0	0
IPR026822	Spp2/MOS2 G-patch domain	15	0	0
IPR026827	Proteasome component Ecm29/Translational activator Gcn1	15	0	0
IPR026947	Ubinuclein middle domain	15	0	0
IPR027075	Cleavage and polyadenylation specificity factor subunit 2	15	0	0
IPR027145	Periodic tryptophan protein 2	15	0	0
IPR027350	Glycosyltransferase family 23 GT23 domain	15	0	0
IPR027521	U6 snRNA phosphodiesterase Usb1	15	0	0
IPR027664	Actin-related protein 5	15	0	0
IPR027670	Exostosin-1	15	0	0
IPR028084	Folliculin-interacting protein N-terminal domain	15	0	0
IPR028085	Folliculin-interacting protein middle domain	15	0	0
IPR028446	Juxtamembrane domain-associated catenin	15	0	0
IPR028554	Ras GTPase-activating protein 1	15	0	0
IPR029382	Lysosomal transcription factor, NCU-G1	15	0	0
IPR029444	Integrator complex subunit 5 C-terminal	15	0	0

InterPro signature	InterPro description	Shared *Papaipema* genes	*Papaipema sp.4* SSGs	*Papaipema speciosissima* SSGs
IPR029446	Coatomer beta subunit appendage platform domain	15	0	0
IPR029702	DNA polymerase alpha catalytic subunit	15	0	0
IPR029806	cAMP-responsive element-binding protein 3-like protein 3	15	0	0
IPR030295	Lysosomal thioesterase PPT2	15	0	0
IPR030605	Kelch-like protein 8	15	0	0
IPR031152	Plexin domain-containing protein	15	0	0
IPR031241	ATP-binding cassette sub-family D member 3	15	0	0
IPR032378	ZC3H15/TMA46 family C-terminal	15	0	0
IPR032393	Stromal interaction molecule Orai1-activating region	15	0	0
IPR032955	Geranylgeranyl transferase type-2 subunit alpha	15	0	0
IPR032976	YjeF N-terminal domain-containing protein, eukaryotes	15	0	0
IPR033031	SCC2/Nipped-B family	15	0	0
IPR003767	Malate/L-lactate dehydrogenase	14	1	4
IPR010998	Integrase Lambda-type N-terminal	14	4	1
IPR000164	Histone H3/CENP-A	14	4	0
IPR004165	Coenzyme A transferase family I	14	3	0
IPR023798	Ribosomal protein S7 domain	14	0	3
IPR001465	Malate synthase	14	1	1
IPR004687	Lysosomal-associated transmembrane protein 4/5	14	2	0
IPR011076	Malate synthase-like	14	1	1

InterPro signature	InterPro description	Shared *Papaipema* genes	*Papaipema sp.4* SSGs	*Papaipema speciosissima* SSGs
IPR015679	Phospholipase D family	14	2	0
IPR018400	Tetraspanning orphan receptor	14	2	0
IPR023574	Ribosomal protein L4 domain	14	1	1
IPR024462	Beta-glucosidase GBA2 type N-terminal	14	2	0
IPR000218	Ribosomal protein L14b/L23e	14	1	0
IPR002117	p53 tumour suppressor family	14	0	1
IPR003068	Transcription factor COUP	14	0	1
IPR004015	SKI-interacting protein SKIP SNW domain	14	1	0
IPR004598	Transcription factor TFIIH subunit p52/Tfb2	14	1	0
IPR005107	CO dehydrogenase flavoprotein C-terminal	14	0	1
IPR005818	Linker histone H1/H5 domain H15	14	1	0
IPR008506	Protein of unknown function DUF788, TMEM208	14	1	0
IPR008521	Magnesium transporter NIPA	14	0	1
IPR008564	Protein of unknown function DUF846, eukaryotic	14	0	1
IPR011174	Ezrin/radixin/moesin	14	0	1
IPR011615	p53 DNA-binding domain	14	1	0
IPR012943	Centrosomin N-terminal motif 1	14	1	0
IPR013148	Glycosyl hydrolase family 32 N-terminal	14	0	1
IPR013810	Ribosomal protein S5 N-terminal	14	1	0
IPR015245	Nuclear RNA export factor Tap RNA-binding domain	14	1	0

InterPro signature	InterPro description	Shared *Papaipema* genes	*Papaipema sp.4* SSGs	*Papaipema speciosissima* SSGs
IPR015672	The Golgi pH regulator/GPCR-type G protein	14	0	1
IPR016643	26S proteasome regulatory complex, non-ATPase subcomplex, Rpn1 subunit	14	1	0
IPR017905	ERV/ALR sulfhydryl oxidase domain	14	1	0
IPR019139	Leucine-rich repeat flightless-interacting protein	14	1	0
IPR020561	Phosphoribosylglycinamide synthetase ATP-grasp A domain	14	0	1
IPR021418	THO complex subunitTHOC2 C-terminal	14	1	0
IPR021869	Ribonuclease Zc3h12a-like NYN domain	14	1	0
IPR023296	Glycosyl hydrolase five-bladed beta-propellor domain	14	0	1
IPR025223	S1-like RNA binding domain	14	0	1
IPR026741	Protein strawberry notch	14	1	0
IPR029710	DNA ligase 4	14	0	1
IPR032852	DNA oxidative demethylase ALKBH2	14	1	0
IPR000358	Ribonucleotide reductase small subunit	14	0	0
IPR000406	Rho protein GDP-dissociation inhibitor	14	0	0
IPR000447	FAD-dependent glycerol-3-phosphate dehydrogenase	14	0	0
IPR000811	Glycosyl transferase, family 35	14	0	0
IPR000922	D-galactoside/L-rhamnose binding SUEL lectin domain	14	0	0
IPR000977	DNA ligase, ATP-dependent	14	0	0
IPR001559	Phosphotriesterase	14	0	0
IPR001631	DNA topoisomerase I	14	0	0

InterPro signature	InterPro description	Shared *Papaipema* genes	*Papaipema sp.4* SSGs	*Papaipema speciosissima* SSGs
IPR001859	Trypanosoma cruzi ribosomal protein P2-like	14	0	0
IPR001921	Ribosomal protein L7A/L8	14	0	0
IPR002306	Tryptophan-tRNA ligase	14	0	0
IPR002433	Ornithine decarboxylase	14	0	0
IPR002466	Adenosine deaminase/editase	14	0	0
IPR002489	Glutamate synthase alpha subunit C-terminal	14	0	0
IPR002646	Poly A polymerase head domain	14	0	0
IPR002733	AMMECR1 domain	14	0	0
IPR002842	ATPase, V1/A1 complex, subunit E	14	0	0
IPR002915	DeoC/FbaB/ lacD aldolase	14	0	0
IPR002932	Glutamate synthase domain	14	0	0
IPR003103	BAG domain	14	0	0
IPR003128	Villin headpiece	14	0	0
IPR003185	Proteasome activator pa28 N-terminal domain	14	0	0
IPR003690	Mitochodrial transcription termination factor	14	0	0
IPR003734	Domain of unknown function DUF155	14	0	0
IPR004007	DhaL domain	14	0	0
IPR004134	Peptidase C1B, bleomycin hydrolase	14	0	0
IPR005012	Daxx protein	14	0	0
IPR005373	Uncharacterised protein family UPF0183	14	0	0

**InterPro signature**	**InterPro description**	**Shared *Papaipema* genes**	***Papaipema sp.4* SSGs**	***Papaipema speciosissima* SSGs**
IPR005631	Flavinator of succinate dehydrogenase	14	0	0
IPR006102	Glycoside hydrolase family 2 immunoglobulin-like beta-sandwich	14	0	0
IPR006800	Pellino family	14	0	0
IPR006849	IKI3	14	0	0
IPR006963	Molybdopterin oxidoreductase 4Fe-4S domain	14	0	0
IPR007015	DNA polymerase V	14	0	0
IPR007230	Peptidase S59 nucleoporin	14	0	0
IPR007275	YTH domain	14	0	0
IPR007528	RINT-1/TIP-20	14	0	0
IPR007741	Ribosomal protein/NADH dehydrogenase domain	14	0	0
IPR007751	Domain of unknown function DUF676 lipase-like	14	0	0
IPR007807	Helicase domain	14	0	0
IPR007857	Protein arginine N-methyltransferase PRMT5	14	0	0
IPR007871	Methyltransferase TRM13	14	0	0
IPR007991	RNA polymerase I specific transcription initiation factor RRN3	14	0	0
IPR007998	Protein of unknown function DUF719	14	0	0
IPR008046	DNA replication licensing factor Mcm3	14	0	0
IPR008211	Laminin N-terminal	14	0	0
IPR008658	Kinesin-associated protein 3	14	0	0
IPR008710	Nicastrin	14	0	0

**InterPro signature**	**InterPro description**	**Shared *Papaipema* genes**	***Papaipema sp.4* SSGs**	***Papaipema speciosissima* SSGs**
IPR008858	TROVE domain	14	0	0
IPR008895	Vps72/YL1 family	14	0	0
IPR009066	Alpha-2-macroglobulin receptor-associated protein domain 1	14	0	0
IPR009138	Neural cell adhesion	14	0	0
IPR009283	Apyrase	14	0	0
IPR009288	Gamma-glutamylcyclotransferase AIG2-like	14	0	0
IPR009539	Vang-like protein	14	0	0
IPR009784	Protein of unknown function DUF1349	14	0	0
IPR010003	HARP domain	14	0	0
IPR010061	Methylmalonate-semialdehyde dehydrogenase	14	0	0
IPR010347	Tyrosyl-DNA phosphodiesterase I	14	0	0
IPR010483	Alpha-2-macroglobulin RAP C-terminal	14	0	0
IPR010643	Helical and beta-bridge domain	14	0	0
IPR010909	PLAC	14	0	0
IPR010996	DNA polymerase beta-like N-terminal domain	14	0	0
IPR011343	Deoxyribose-phosphate aldolase	14	0	0
IPR011442	TAF6 C-terminal HEAT repeat domain	14	0	0
IPR011538	NADH-ubiquinone oxidoreductase 51 kDa subunit FMN-binding domain	14	0	0
IPR011697	Peptidase C26	14	0	0
IPR011722	Hemimethylated DNA-binding domain	14	0	0

InterPro signature	InterPro description	Shared *Papaipema* genes	*Papaipema sp.4* SSGs	*Papaipema speciosissima* SSGs
IPR012419	Cas1p 10 TM acyl transferase domain	14	0	0
IPR012446	Calcium release-activated calcium channel protein	14	0	0
IPR012588	Exosome-associated factor Rrp6 N-terminal	14	0	0
IPR012792	3-oxoacid CoA-transferase subunit A	14	0	0
IPR012916	RED-like N-terminal	14	0	0
IPR012932	Vitamin K epoxide reductase	14	0	0
IPR013176	Protein of unknown function DUF1712, fungi	14	0	0
IPR013315	Spectrin alpha chain SH3 domain	14	0	0
IPR013499	DNA topoisomerase I eukaryotic-type	14	0	0
IPR013500	DNA topoisomerase I catalytic core eukaryotic-type	14	0	0
IPR013547	Prolyl 4-hydroxylase alpha-subunit N-terminal	14	0	0
IPR013842	GTP-binding protein LepA C-terminal	14	0	0
IPR014100	GTP-binding protein Obg/CgtA	14	0	0
IPR014645	Target of Myb protein 1	14	0	0
IPR014840	Hpc2-related domain	14	0	0
IPR014883	VRR-NUC domain	14	0	0
IPR014893	Ku C-terminal	14	0	0
IPR015222	Mitochondrial matrix Mmp37	14	0	0
IPR015342	Peroxisome biogenesis factor 1 N-terminal psi beta-barrel fold	14	0	0
IPR015427	Synaptotagmin 7	14	0	0

InterPro signature	InterPro description	Shared *Papaipema* genes	*Papaipema sp.4* SSGs	*Papaipema speciosissima* SSGs
IPR015727	Protein kinase C mu-related	14	0	0
IPR016092	FeS cluster insertion protein	14	0	0
IPR016125	Peptidase C15, pyroglutamyl peptidase I-like	14	0	0
IPR016152	Phosphotransferase/anion transporter	14	0	0
IPR016695	Purine 5'-nucleotidase	14	0	0
IPR018615	Ribosomal protein L55, mitochondrial	14	0	0
IPR018732	Dpy-19/Dpy-19-like	14	0	0
IPR018955	Branched-chain alpha-ketoacid dehydrogenase kinase/Pyruvate dehydrogenase kinase N-terminal	14	0	0
IPR018997	PUB domain	14	0	0
IPR019188	Small nuclear RNA activating complex SNAPc), subunit SNAP43	14	0	0
IPR019269	Biogenesis of lysosome-related organelles complex-1, subunit 2	14	0	0
IPR019308	Protein of unknown function DUF2359, TMEM214	14	0	0
IPR019309	WASH complex, subunit CCDC53	14	0	0
IPR019371	Uncharacterised domain KxDL	14	0	0
IPR019388	Fat storage-inducing transmembrane protein	14	0	0
IPR019451	RNA polymerase II assembly factor Rtp1 C-terminal	14	0	0
IPR019529	SNARE-complex protein Syntaxin-18 N-terminal	14	0	0
IPR021139	NYN domain limkain-b1-type	14	0	0
IPR021394	Mediator complex subunit Med25 PTOV activation and synapsin 2	14	0	0
IPR022088	Intraflagellar transport complex B protein 46	14	0	0

InterPro signature	InterPro description	Shared *Papaipema* genes	*Papaipema sp.4* SSGs	*Papaipema speciosissima* SSGs
IPR022542	Domain of unknown function DUF3730	14	0	0
IPR022644	Orn/DAP/Arg decarboxylase 2 N-terminal	14	0	0
IPR023273	RNA C5-cytosine methyltransferase, NOP2	14	0	0
IPR023397	S-adenosyl-L-methionine-dependent methyltransferase MraW recognition domain	14	0	0
IPR023473	AMMECR1	14	0	0
IPR024142	Superoxide dismutase 1 copper chaperone	14	0	0
IPR024146	Claspin	14	0	0
IPR024789	Anaphase-promoting complex subunit 4	14	0	0
IPR024977	Anaphase-promoting complex subunit 4 WD40 domain	14	0	0
IPR025202	Phospholipase D-like domain	14	0	0
IPR025220	NFRKB winged helix-like domain	14	0	0
IPR025483	Lipase, eukaryotic	14	0	0
IPR025653	Peroxisome biogenesis factor 1	14	0	0
IPR025704	E3 ubiquitin ligase, UBR4	14	0	0
IPR025834	Topoisomerase I C-terminal domain	14	0	0
IPR026180	KAT8 regulatory NSL complex subunit 1	14	0	0
IPR026209	Wolframin family	14	0	0
IPR026750	Protein N-terminal asparagine amidohydrolase	14	0	0
IPR026773	Trichoplein keratin filament-binding protein	14	0	0
IPR026818	Adenomatous polyposis coli APC family	14	0	0

InterPro signature	InterPro description	Shared *Papaipema* genes	*Papaipema sp.4* SSGs	*Papaipema speciosissima* SSGs
IPR026895	ER membrane protein complex subunit 1	14	0	0
IPR027031	Glycyl-tRNA synthetase/DNA polymerase subunit gamma-2	14	0	0
IPR027105	U4/U6 small nuclear ribonucleoprotein Prp31	14	0	0
IPR027131	Structural maintenance of chromosomes protein 5	14	0	0
IPR027673	Exostosin-2	14	0	0
IPR027805	Transposase Helix-turn-helix domain	14	0	0
IPR027817	Costars domain	14	0	0
IPR027902	Protein of unknown function DUF4487	14	0	0
IPR027951	Domain of unknown function DUF4477	14	0	0
IPR028108	Protein of unknown function DUF4505	14	0	0
IPR028823	Sodium leak channel non-selective protein	14	0	0
IPR029038	Methionyl-tRNA synthetase Zn-domain	14	0	0
IPR029347	Raptor N-terminal CASPase-like domain	14	0	0
IPR029704	UPF0428 family	14	0	0
IPR029765	Diphosphomevalonate decarboxylase	14	0	0
IPR030038	C-mannosyltransferase Dpy-19	14	0	0
IPR030230	GPN-loop GTPase 1	14	0	0
IPR030559	DNA polymerase zeta catalytic subunit	14	0	0
IPR031047	DNA helicase Ino80	14	0	0
IPR031238	Peroxisomal long-chain fatty acid import protein 1	14	0	0

**InterPro signature**	**InterPro description**	**Shared *Papaipema* genes**	***Papaipema sp.4* SSGs**	***Papaipema speciosissima* SSGs**
IPR031335	Glycosyl hydrolase family 63 C-terminal	14	0	0
IPR031437	Transmembrane protein family 132 middle domain	14	0	0
IPR031557	N-CoR GPS2-interacting domain	14	0	0
IPR031595	Protein-only RNase P C-terminal	14	0	0
IPR031656	Alpha-glycerophosphate oxidase C-terminal	14	0	0
IPR031727	Galactose mutarotase N-terminal barrel	14	0	0
IPR031919	Alpha-L-fucosidase C-terminal	14	0	0
IPR032150	Protein of unknown function DUF4820	14	0	0
IPR032302	THO complex subunit 2 N-terminal domain	14	0	0
IPR032432	Radical SAM C-terminal extension	14	0	0
IPR032453	Homeobox protein PKNOX/Meis N-terminal	14	0	0
IPR032467	Yemanuclein	14	0	0
IPR032563	DAMP1 SANT/Myb-like domain	14	0	0
IPR032819	tRNA pseudouridylate synthase B C-terminal	14	0	0
IPR009056	Cytochrome c-like domain	13	0	5
IPR002379	V-ATPase proteolipid subunit C-like domain	13	1	3
IPR002888	[2Fe-2S]-binding	13	3	1
IPR001568	Ribonuclease T2-like	13	2	1
IPR005294	ATPase, F1 complex, alpha subunit	13	2	1
IPR005484	Ribosomal protein L18/L5	13	3	0

InterPro signature	InterPro description	Shared *Papaipema* genes	*Papaipema sp.4* SSGs	*Papaipema speciosissima* SSGs
IPR005532	Sulfatase-modifying factor enzyme	13	3	0
IPR002346	Molybdopterin dehydrogenase FAD-binding	13	2	0
IPR005809	Succinyl-CoA synthetase, beta subunit	13	2	0
IPR005324	Ribosomal protein S5 C-terminal	13	2	0
IPR015788	Moesin/ezrin/radixin homologue 2	13	1	1
IPR016180	Ribosomal protein L10e/L16	13	1	1
IPR018289	MULE transposase domain	13	1	1
IPR019464	RNA polymerase II elongation factor ELL N-terminal	13	0	2
IPR030364	K/Cl co-transporter 3	13	2	0
IPR028407	Phospholipase C, Plc21C	13	0	1
IPR002589	Macro domain	13	0	1
IPR003817	Phosphatidylserine decarboxylase-related	13	1	0
IPR004500	Prolyl-tRNA synthetase, class IIa, bacterial-type	13	1	0
IPR006593	Cytochrome b561/ferric reductase transmembrane	13	1	0
IPR006735	Protein of unknown function DUF602	13	0	1
IPR007304	TAP42-like protein	13	0	1
IPR007832	RNA polymerase Rpc34	13	1	0
IPR007856	Saposin-like type B region 1	13	0	1
IPR008138	Saposin B type region 2	13	0	1
IPR008383	Apoptosis inhibitory 5	13	0	1

**InterPro signature**	**InterPro description**	**Shared *Papaipema* genes**	***Papaipema sp.4* SSGs**	***Papaipema speciosissima* SSGs**
IPR010358	Brain/reproductive organ-expressed protein	13	0	1
IPR013749	Pyridoxamine kinase/Phosphomethylpyrimidine kinase	13	1	0
IPR020828	Glyceraldehyde 3-phosphate dehydrogenase NADP binding domain	13	0	1
IPR020838	DBINO domain	13	0	1
IPR024317	Dynein heavy chain P-loop containing D4 domain	13	0	1
IPR029404	Protein of unknown function with TPD sequence-motif	13	0	1
IPR000007	Tubby C-terminal	13	0	0
IPR000183	Ornithine/DAP/Arg decarboxylase	13	0	0
IPR000184	Bacterial surface antigen D15	13	0	0
IPR000233	Cadherin cytoplasmic domain	13	0	0
IPR000511	Cytochrome c/c1 haem-lyase	13	0	0
IPR000519	P-type trefoil domain	13	0	0
IPR000659	Pyridoxamine 5'-phosphate oxidase	13	0	0
IPR000816	Peptidase C15, pyroglutamyl peptidase I	13	0	0
IPR000860	Porphobilinogen deaminase	13	0	0
IPR000996	Clathrin light chain	13	0	0
IPR001161	Helicase XPB/Ssl2	13	0	0
IPR001289	Nuclear transcription factor Y subunit A	13	0	0
IPR001352	Ribonuclease HII/HIII	13	0	0
IPR001506	Peptidase M12A astacin	13	0	0

InterPro signature	InterPro description	Shared *Papaipema* genes	*Papaipema sp.4* SSGs	*Papaipema speciosissima* SSGs
IPR001518	Argininosuccinate synthase	13	0	0
IPR001608	Alanine racemase N-terminal	13	0	0
IPR001765	Carbonic anhydrase	13	0	0
IPR001998	Xylose isomerase	13	0	0
IPR002000	Lysosome-associated membrane glycoprotein	13	0	0
IPR002081	Cryptochrome/DNA photolyase class 1	13	0	0
IPR002157	Cobalamin vitamin B12)-binding transporter, eukaryotic	13	0	0
IPR002164	Nucleosome assembly protein NAP)	13	0	0
IPR002182	NB-ARC	13	0	0
IPR002418	Transcription regulator Myc	13	0	0
IPR002650	Sulphate adenylyltransferase	13	0	0
IPR002695	AICARFT/IMPCHase bienzyme	13	0	0
IPR002818	DJ-1/PfpI	13	0	0
IPR002843	ATPase, V0 complex, c/d subunit	13	0	0
IPR002889	Carbohydrate-binding WSC	13	0	0
IPR003166	Transcription factor TFIIE beta subunit DNA-binding domain	13	0	0
IPR003379	Carboxylase conserved domain	13	0	0
IPR003382	Flavoprotein	13	0	0
IPR003692	Hydantoinase B/oxoprolinase	13	0	0
IPR003698	Lipoyl synthase	13	0	0

**InterPro signature**	**InterPro description**	**Shared *Papaipema* genes**	***Papaipema sp.4* SSGs**	***Papaipema speciosissima* SSGs**
IPR003889	FY-rich C-terminal	13	0	0
IPR003950	Potassium channel, voltage-dependent, ELK	13	0	0
IPR003953	FAD-dependent oxidoreductase 2 FAD binding domain	13	0	0
IPR004098	Prp18	13	0	0
IPR004141	Strictosidine synthase	13	0	0
IPR004361	Glyoxalase I	13	0	0
IPR004583	DNA repair protein Rad4	13	0	0
IPR004584	DNA repair protein Rad50, eukaryotes	13	0	0
IPR004739	GMP synthase N-terminal	13	0	0
IPR004879	Domain of unknown function DUF255	13	0	0
IPR004907	ATPase, V1 complex, subunit C	13	0	0
IPR005034	Dicer dimerisation domain	13	0	0
IPR005336	Mitochondrial pyruvate carrier	13	0	0
IPR005339	GINS complex, subunit Psf1	13	0	0
IPR005366	Uncharacterised protein family UPF0172	13	0	0
IPR005408	Two pore domain potassium channel, TWIK family	13	0	0
IPR005654	ATPase, AFG1-like	13	0	0
IPR005708	Homogentisate 1,2-dioxygenase	13	0	0
IPR005772	ATPase, V1 complex, subunit F, eukaryotic	13	0	0
IPR005817	Wnt	13	0	0

**InterPro signature**	**InterPro description**	**Shared *Papaipema* genes**	***Papaipema sp.4* SSGs**	***Papaipema speciosissima* SSGs**
IPR005959	Fumarylacetoacetase	13	0	0
IPR006196	RNA-binding domain S1 IF1 type	13	0	0
IPR006287	DJ-1	13	0	0
IPR006536	HnRNP-L/PTB	13	0	0
IPR006621	Nose resistant-to-fluoxetine protein N-terminal	13	0	0
IPR006656	Molybdopterin oxidoreductase	13	0	0
IPR006678	tRNA intron endonuclease N-terminal	13	0	0
IPR006925	Vps16 C-terminal	13	0	0
IPR006968	Root UVB sensitive family	13	0	0
IPR007000	Phospholipase B-like	13	0	0
IPR007085	DNA/pantothenate metabolism flavoprotein C-terminal	13	0	0
IPR007144	Small-subunit processome, Utp11	13	0	0
IPR007225	Exocyst complex subunit Sec15-like	13	0	0
IPR007290	Arv1 protein	13	0	0
IPR007308	Protein of unknown function DUF408	13	0	0
IPR007356	tRNA guanine-N1-)-methyltransferase, eukaryotic	13	0	0
IPR007666	ADP-specific phosphofructokinase/glucokinase	13	0	0
IPR007726	SS18 family	13	0	0
IPR007797	Transcription factor AF4/FMR2	13	0	0
IPR007884	DREV methyltransferase	13	0	0

InterPro signature	InterPro description	Shared *Papaipema* genes	*Papaipema sp.4* SSGs	*Papaipema speciosissima* SSGs
IPR008162	Inorganic pyrophosphatase	13	0	0
IPR008218	ATPase, V1 complex, subunit F	13	0	0
IPR008336	DNA topoisomerase I DNA binding eukaryotic-type	13	0	0
IPR008376	Synembryn	13	0	0
IPR008428	Chondroitin N-acetylgalactosaminyltransferase	13	0	0
IPR008568	Uncharacterised conserved protein UCP010045, transmembrane eukaryotic	13	0	0
IPR008614	Acidic fibroblast growth factor intracellular-binding protein	13	0	0
IPR008728	Elongator complex protein 4	13	0	0
IPR008907	P25-alpha	13	0	0
IPR008996	Cytokine, IL-1-like	13	0	0
IPR009146	Groucho/transducin-like enhancer	13	0	0
IPR009723	Ribonuclease P/MRP subunit POP1	13	0	0
IPR009730	Micro-fibrillar-associated protein 1 C-terminal	13	0	0
IPR010226	NADH-quinone oxidoreductase, chain I	13	0	0
IPR010448	Torsin	13	0	0
IPR010606	Mib-herc2	13	0	0
IPR010844	Occludin homology domain	13	0	0
IPR011043	Galactose oxidase/kelch beta-propeller	13	0	0
IPR011078	Uncharacterised protein family UPF0001	13	0	0
IPR011576	Pyridoxamine 5'-phosphate oxidase-like FMN-binding domain	13	0	0

**InterPro signature**	**InterPro description**	**Shared *Papaipema* genes**	***Papaipema sp.4* SSGs**	***Papaipema speciosissima* SSGs**
IPR012000	Thiamine pyrophosphate enzyme central domain	13	0	0
IPR012099	Midasin	13	0	0
IPR012349	FMN-binding split barrel	13	0	0
IPR012394	Aldehyde dehydrogenase NADP)-dependent	13	0	0
IPR012791	3-oxoacid CoA-transferase subunit B	13	0	0
IPR012904	8-oxoguanine DNA glycosylase N-terminal	13	0	0
IPR012937	FAM46 family	13	0	0
IPR012945	Tubulin binding cofactor C-like domain	13	0	0
IPR013029	Domain of unknown function DUF933	13	0	0
IPR013507	DNA mismatch repair protein C-terminal	13	0	0
IPR013641	Protein KTI12/L-seryl-tRNASec kinase	13	0	0
IPR013924	Ribonuclease H2, subunit C	13	0	0
IPR013953	FACT complex subunit Spt16p/Cdc68p	13	0	0
IPR013994	Carbohydrate-binding WSC subgroup	13	0	0
IPR014388	3-oxoacid CoA-transferase	13	0	0
IPR015252	Breast cancer type 2 susceptibility protein helical domain	13	0	0
IPR015390	Rabaptin GTPase-Rab5 binding domain	13	0	0
IPR015616	Growth/differentiation factor 8	13	0	0
IPR015795	Pyruvate kinase C-terminal	13	0	0
IPR015987	Uncharacterised conserved protein UCP022704	13	0	0

InterPro signature	InterPro description	Shared *Papaipema* genes	*Papaipema sp.4* SSGs	*Papaipema speciosissima* SSGs
IPR016049	RNA polymerase Rpc34-like	13	0	0
IPR016151	DNA mismatch repair protein MutS N-terminal	13	0	0
IPR016179	Insulin-like	13	0	0
IPR016342	AP-1, 2,4 complex subunit beta	13	0	0
IPR016438	ATP-dependent RNA helicase Ski2	13	0	0
IPR016527	Origin recognition complex subunit 4	13	0	0
IPR016727	ATPase, V0 complex, subunit d	13	0	0
IPR017245	BLOC-1 complex, subunit 3	13	0	0
IPR017393	SWI/SNF chromatin-remodeling complex, component hSNF5/Ini1	13	0	0
IPR017893	DBB domain	13	0	0
IPR018119	Strictosidine synthase conserved region	13	0	0
IPR018477	Bicaudal-D protein, microtubule-associated	13	0	0
IPR018781	Transmembrane protein adipocyte-associated 1	13	0	0
IPR018961	DnaJ homologue subfamily C member 28 conserved domain	13	0	0
IPR019049	Nucleoporin protein Ndc1-Nup	13	0	0
IPR019174	NADH dehydrogenase 1, beta subcomplex, subunit 6	13	0	0
IPR019313	Mediator complex, subunit Med17	13	0	0
IPR019349	Ribosomal protein S24/S35 mitochondrial conserved domain	13	0	0
IPR019358	NEMP family	13	0	0
IPR019363	Lipid droplet-associated hydrolase	13	0	0

**InterPro signature**	**InterPro description**	**Shared *Papaipema* genes**	***Papaipema sp.4* SSGs**	***Papaipema speciosissima* SSGs**
IPR019407	Cytoplasmic tRNA 2-thiolation protein 2	13	0	0
IPR019538	26S proteasome non-ATPase regulatory subunit 5	13	0	0
IPR019574	NADH:ubiquinone oxidoreductase subunit G iron-sulphur binding	13	0	0
IPR020581	Glycine cleavage system P protein	13	0	0
IPR020821	Extracellular Endonuclease subunit A	13	0	0
IPR021643	Mediator complex subunit Med13 N-terminal metazoa/fungi	13	0	0
IPR021789	KHA domain	13	0	0
IPR021850	Symplekin/Pta1	13	0	0
IPR021854	WASH1 WAHD domain	13	0	0
IPR021917	Uncharacterised protein family, zinc metallopeptidase-like	13	0	0
IPR022030	Splicing factor 3A subunit 1	13	0	0
IPR022535	Golgi pH regulator conserved domain	13	0	0
IPR023302	Peptidase S9A N-terminal domain	13	0	0
IPR024100	Transcription factor E3	13	0	0
IPR024137	Histone deacetylase complex subunit SAP130	13	0	0
IPR024205	Mst1 SARAH domain	13	0	0
IPR024372	Proteasome component Ecm29	13	0	0
IPR024567	Ribonuclease HII/HIII domain	13	0	0
IPR024733	Alpha-N-acetylglucosaminidase tim-barrel domain	13	0	0
IPR024867	Nuclear factor related to kappa-B-binding protein	13	0	0

InterPro signature	InterPro description	Shared *Papaipema* genes	*Papaipema sp.4* SSGs	*Papaipema speciosissima* SSGs
IPR024868	Four-jointed box protein 1/four-jointed protein	13	0	0
IPR024875	Protein Lines	13	0	0
IPR024986	Sister chromatid cohesion C-terminal domain	13	0	0
IPR025650	Alkyldihydroxyacetonephosphate synthase	13	0	0
IPR025777	GMP synthetase ATP pyrophosphatase domain	13	0	0
IPR025812	Mitochondrial ribonuclease P, tRNA methyltransferase protein 1	13	0	0
IPR026113	Methyltransferase-like	13	0	0
IPR026218	Heme transporter HRG	13	0	0
IPR026306	Round spermatid basic protein 1	13	0	0
IPR026610	3'-RNA ribose 2'-O-methyltransferase, Hen1	13	0	0
IPR026678	INO80 complex subunit E	13	0	0
IPR026849	Autophagy-related protein 2	13	0	0
IPR026928	Failed axon connections	13	0	0
IPR027668	Actin-related protein 8/Plant actin-related protein 9	13	0	0
IPR027684	Tubulin-specific chaperone C	13	0	0
IPR027844	Protein of unknown function DUF4507	13	0	0
IPR028045	Protein of unknown function DUF4539	13	0	0
IPR028226	Protein LIN37	13	0	0
IPR028275	Clustered mitochondria protein N-terminal	13	0	0
IPR028290	WASH1	13	0	0

InterPro signature	InterPro description	Shared *Papaipema* genes	*Papaipema sp.4* SSGs	*Papaipema speciosissima* SSGs
IPR028458	Twinfilin	13	0	0
IPR028755	Tissue alpha-L-fucosidase	13	0	0
IPR029338	Tumour suppressing sub-chromosomal transferable candidate 4	13	0	0
IPR029523	INO80 complex, subunit Ies2	13	0	0
IPR029732	Pre-mRNA-splicing regulator WTAP	13	0	0
IPR030100	SWI/SNF complex subunit BRG1	13	0	0
IPR030392	Intramolecular chaperone auto-processing domain	13	0	0
IPR030444	Liprin-beta	13	0	0
IPR030450	Transcription factor Sp1 family	13	0	0
IPR030633	Dihydropyrimidinase	13	0	0
IPR030701	Transcription factor TFIIIB component B''	13	0	0
IPR031082	Laminin subunit gamma	13	0	0
IPR031331	Neutral/alkaline non-lysosomal ceramidase C-terminal	13	0	0
IPR031341	Ribosomal RNA small subunit methyltransferase F N-terminal	13	0	0
IPR031631	Glycosyl hydrolase family 63 N-terminal	13	0	0
IPR031801	Virilizer N-terminal	13	0	0
IPR031925	Tubulin-specific chaperone C N-terminal	13	0	0
IPR031963	Histone deacetylase complex subunit SAP130 C-terminal domain	13	0	0
IPR032189	DNA mismatch repair protein Mlh1 C-terminal	13	0	0
IPR032446	S phase cyclin A-associated protein in the endoplasmic reticulum N-terminal	13	0	0

InterPro signature	InterPro description	Shared *Papaipema* genes	*Papaipema sp.4* SSGs	*Papaipema speciosissima* SSGs
IPR032679	Sin1 N-terminal	13	0	0
IPR032705	Origin recognition complex subunit 4 C-terminal	13	0	0
IPR032794	Protein Lines N-terminal	13	0	0
IPR032828	tRNA nucleotidyltransferase/polyA polymerase RNA and SrmB- binding domain	13	0	0
IPR033008	COP9 signalosome complex subunit 1	13	0	0
IPR033060	Integrator complex subunit 7	13	0	0
IPR033177	Phosphatidylserine decarboxylase	13	0	0
IPR027124	SWR1-complex protein 5/Craniofacial development protein	12	2	2
IPR001585	Transaldolase/Fructose-6-phosphate aldolase	12	3	0
IPR018105	Translationally controlled tumour protein	12	3	0
IPR023610	Phosphatidylinositol-4-phosphate 5-kinase	12	2	1
IPR008037	Pacifastin domain	12	1	1
IPR011657	Concentrative nucleoside transporter C-terminal domain	12	2	0
IPR013659	Adenosine/AMP deaminase N-terminal	12	2	0
IPR018940	Elongation factor 1 beta central acidic region eukaryote	12	1	1
IPR019412	Outer membrane protein Iml2/Tetratricopeptide repeat protein 39	12	0	2
IPR001362	Glycoside hydrolase, family 32	12	1	0
IPR001380	Ribosomal protein L13e	12	1	0
IPR001830	Glycosyl transferase family 20	12	1	0
IPR003196	Transcription initiation factor IIF, beta subunit	12	1	0

**InterPro signature**	**InterPro description**	**Shared *Papaipema* genes**	***Papaipema sp.4* SSGs**	***Papaipema speciosissima* SSGs**
IPR003789	Aspartyl/glutamyl-tRNA amidotransferase subunit B-related	12	0	1
IPR004133	DAN	12	1	0
IPR004277	Phosphatidyl serine synthase	12	0	1
IPR004403	Peptide chain release factor eRF1/aRF1	12	1	0
IPR004625	Pyridoxal phosphate active vitamin B6 biosynthesis, pyridoxal kinase	12	0	1
IPR004939	Anaphase-promoting complex subunit 10/DOC domain	12	1	0
IPR004948	Nucleoside-triphosphatase, THEP1 type	12	1	0
IPR007307	Low temperature viability protein	12	0	1
IPR008496	Protein of unknown function DUF778	12	0	1
IPR009422	Gemin6	12	0	1
IPR009602	FAM92 protein	12	1	0
IPR010280	Uracil-5)-methyltransferase family	12	0	1
IPR014038	Translation elongation factor EF1B beta/delta subunit guanine nucleotide exchange	12	0	1
IPR017665	Guanylate kinase	12	1	0
IPR017959	Aspartyl/glutamyl-tRNAAsn/Gln amidotransferase, subunit B /E	12	1	0
IPR018027	Asn/Gln amidotransferase	12	0	1
IPR018951	Fumarase C C-terminal	12	0	1
IPR023564	Ribosomal protein L13 domain	12	0	1
IPR024049	Peptide Chain Release Factor eRF1/aRF1 N-terminal	12	0	1
IPR025192	Succinate dehydogenase/fumarate reductase N-terminal	12	0	1

InterPro signature	InterPro description	Shared *Papaipema* genes	*Papaipema sp.4* SSGs	*Papaipema speciosissima* SSGs
IPR026074	Microtubule associated protein 1	12	1	0
IPR026147	Rab3 GTPase-activating protein catalytic subunit	12	1	0
IPR026489	CXC domain	12	1	0
IPR027120	Structural maintenance of chromosomes Smc2	12	1	0
IPR027681	I-BAR domain containing protein IRSp53/IRTKS/Pinkbar	12	1	0
IPR027749	Tubulin--tyrosine ligase-like protein 12	12	0	1
IPR032478	Staufen C-terminal	12	0	1
IPR000115	Phosphoribosylglycinamide synthetase	12	0	0
IPR000146	Fructose-1,6-bisphosphatase class 1/Sedoheputulose-1,7-bisphosphatase	12	0	0
IPR000473	Ribosomal protein L36	12	0	0
IPR000493	Inositol 1,4,5-trisphosphate-binding protein receptor	12	0	0
IPR000601	PKD domain	12	0	0
IPR000702	Ribosomal protein L6	12	0	0
IPR000788	Ribonucleotide reductase large subunit C-terminal	12	0	0
IPR000885	Fibrillar collagen C-terminal	12	0	0
IPR000889	Glutathione peroxidase	12	0	0
IPR000915	60S ribosomal protein L6E	12	0	0
IPR000965	GPR domain	12	0	0
IPR001096	Peptidase C13, legumain	12	0	0
IPR001351	Ribosomal protein S3 C-terminal	12	0	0

InterPro signature	InterPro description	Shared *Papaipema* genes	*Papaipema sp.4* SSGs	*Papaipema speciosissima* SSGs
IPR001754	Orotidine 5'-phosphate decarboxylase domain	12	0	0
IPR001817	Vasopressin receptor	12	0	0
IPR001892	Ribosomal protein S13	12	0	0
IPR001898	Sodium/sulphate symporter	12	0	0
IPR001944	Glycoside hydrolase, family 35	12	0	0
IPR002099	DNA mismatch repair protein family	12	0	0
IPR002209	Fibroblast growth factor family	12	0	0
IPR002291	Phosphorylase kinase, gamma catalytic subunit	12	0	0
IPR002302	Leucine-tRNA ligase	12	0	0
IPR002315	Glycyl-tRNA synthetase	12	0	0
IPR002443	Na/K/Cl co-transporter	12	0	0
IPR002569	Peptide methionine sulphoxide reductase MsrA	12	0	0
IPR002618	UTP--glucose-1-phosphate uridylyltransferase family	12	0	0
IPR002666	Reduced folate carrier	12	0	0
IPR002715	Nascent polypeptide-associated complex NAC domain	12	0	0
IPR002794	Protein of unknown function DUF92, TMEM19	12	0	0
IPR002872	Proline dehydrogenase domain	12	0	0
IPR003011	Cell cycle checkpoint protein, Rad1	12	0	0
IPR003152	FATC domain	12	0	0
IPR003226	Metal-dependent protein hydrolase	12	0	0

**InterPro signature**	**InterPro description**	**Shared *Papaipema* genes**	***Papaipema sp.4* SSGs**	***Papaipema speciosissima* SSGs**
IPR003293	Nudix hydrolase 6-like	12	0	0
IPR003428	Mitochondrial glycoprotein	12	0	0
IPR003511	HORMA domain	12	0	0
IPR003694	NAD+ synthetase	12	0	0
IPR003732	D-aminoacyl-tRNA deacylase DTD	12	0	0
IPR003780	COX15/CtaA family	12	0	0
IPR003788	Putative S-adenosyl-L-methionine-dependent methyltransferase MidA	12	0	0
IPR003937	Potassium channel, voltage dependent, KCNQ	12	0	0
IPR004140	Exocyst complex protein Exo70	12	0	0
IPR004167	E3-binding domain	12	0	0
IPR004192	Ubiquinol cytochrome reductase transmembrane domain	12	0	0
IPR004313	Acireductone dioxygenase ARD family	12	0	0
IPR004327	Phosphotyrosyl phosphatase activator, PTPA	12	0	0
IPR004343	Plus-3 domain	12	0	0
IPR004353	Vacuolar fusion protein MON1	12	0	0
IPR004489	Succinate dehydrogenase/fumarate reductase iron-sulphur protein	12	0	0
IPR004574	Alkylated DNA repair protein AlkB	12	0	0
IPR004728	Translocation protein Sec62	12	0	0
IPR004815	Lon protease, bacterial/eukaryotic-type	12	0	0
IPR004908	ATPase, V1 complex, subunit H	12	0	0

InterPro signature	InterPro description	Shared *Papaipema* genes	*Papaipema sp.4* SSGs	*Papaipema speciosissima* SSGs
IPR005027	Glycosyl transferase, family 43	12	0	0
IPR005079	Peptidase C45	12	0	0
IPR005352	Erg28	12	0	0
IPR005467	Histidine kinase domain	12	0	0
IPR005707	Ribosomal protein S2, eukaryotic/archaeal	12	0	0
IPR005715	Glutamate 5-kinase/delta-1-pyrroline-5-carboxylate synthase	12	0	0
IPR005919	Higher eukaryotic phosphomevalonate kinase	12	0	0
IPR006095	Glutamate/phenylalanine/leucine/valine dehydrogenase	12	0	0
IPR006097	Glutamate/phenylalanine/leucine/valine dehydrogenase dimerisation domain	12	0	0
IPR006116	2-5-oligoadenylate synthetase N-terminal	12	0	0
IPR006183	6-phosphogluconate dehydrogenase	12	0	0
IPR006205	Mevalonate kinase	12	0	0
IPR006349	2-phosphoglycolate phosphatase, eukaryotic	12	0	0
IPR006572	Zinc finger DBF-type	12	0	0
IPR006785	Peroxisome membrane anchor protein Pex14p N-terminal	12	0	0
IPR006926	Vps16 N-terminal	12	0	0
IPR007135	Autophagy-related protein 3	12	0	0
IPR007198	Ssl1-like	12	0	0
IPR007252	Nuclear pore protein 84/107	12	0	0
IPR007273	SCAMP	12	0	0

**InterPro signature**	**InterPro description**	**Shared *Papaipema* genes**	***Papaipema sp.4* SSGs**	***Papaipema speciosissima* SSGs**
IPR007474	ApaG domain	12	0	0
IPR007523	NDUFAF3/Mth938 domain-containing protein	12	0	0
IPR007667	Hypoxia induced protein domain	12	0	0
IPR007708	Lariat debranching enzyme C-terminal	12	0	0
IPR007715	Ubiquinone biosynthesis protein Coq4	12	0	0
IPR007763	NADH dehydrogenase [ubiquinone] 1 alpha subcomplex subunit 12	12	0	0
IPR007810	Pep3/Vps18/deep orange	12	0	0
IPR007823	Methyltransferase-related	12	0	0
IPR007853	Zinc finger DNL-type	12	0	0
IPR007881	UNC-50	12	0	0
IPR008111	RNA-binding motif protein 8	12	0	0
IPR008343	Mitogen-activated protein MAP kinase phosphatase	12	0	0
IPR008468	DNA methyltransferase 1-associated 1	12	0	0
IPR008504	ER membrane protein complex subunit 6	12	0	0
IPR008591	GINS complex subunit Sld5	12	0	0
IPR008826	Selenium-binding protein	12	0	0
IPR008847	Suppressor of forked	12	0	0
IPR009049	Argininosuccinate lyase	12	0	0
IPR009244	Mediator complex, subunit Med7	12	0	0
IPR009286	Inositol-pentakisphosphate 2-kinase	12	0	0

InterPro signature	InterPro description	Shared *Papaipema* genes	*Papaipema sp.4* SSGs	*Papaipema speciosissima* SSGs
IPR009348	Nitrogen permease regulator 2	12	0	0
IPR009675	TPX2	12	0	0
IPR009685	Male enhanced antigen 1	12	0	0
IPR010228	NADH:ubiquinone oxidoreductase, subunit G	12	0	0
IPR010300	Cysteine dioxygenase type I	12	0	0
IPR010334	mRNA-decapping enzyme subunit 1	12	0	0
IPR010487	Neugrin/Rrg9	12	0	0
IPR010508	Domain of unknown function DUF1088	12	0	0
IPR010561	Protein LIN-9/Protein ALWAYS EARLY	12	0	0
IPR010721	Protein of unknown function DUF1295	12	0	0
IPR010733	Protein of unknown function DUF1308	12	0	0
IPR010895	CHRD	12	0	0
IPR010912	Spen paralogue/orthologue C-terminal metazoa	12	0	0
IPR010979	Ribosomal protein S13-like H2TH	12	0	0
IPR011082	Exosome-associated factor Rrp47/DNA strand repair C1D	12	0	0
IPR011107	Protein phosphatase inhibitor	12	0	0
IPR011430	Down-regulated-in-metastasis protein	12	0	0
IPR011691	Vesicle transport protein, SFT2-like	12	0	0
IPR011833	Glycogen/starch/alpha-glucan phosphorylase	12	0	0
IPR012170	TFIIH subunit Ssl1/p44	12	0	0

**InterPro signature**	**InterPro description**	**Shared *Papaipema* genes**	***Papaipema sp.4* SSGs**	***Papaipema speciosissima* SSGs**
IPR012173	U3 small nucleolar ribonucleoprotein complex, subunit Mpp10	12	0	0
IPR012178	Replication factor C subunit 1	12	0	0
IPR012908	GPI inositol-deacylase PGAP1-like	12	0	0
IPR012959	CPL domain	12	0	0
IPR013180	Beta-catenin-like protein 1 N-terminal	12	0	0
IPR013197	RNA polymerase III subunit RPC82-related helix-turn-helix	12	0	0
IPR013260	mRNA splicing factor SYF2	12	0	0
IPR013284	Beta-catenin	12	0	0
IPR013724	GIT Spa2 homology SHD domain	12	0	0
IPR013868	Tethering factor for nuclear proteasome Cut8/Sts1	12	0	0
IPR013926	CGI121/TPRKB	12	0	0
IPR014349	Rieske iron-sulphur protein	12	0	0
IPR014369	Glycine/Sarcosine N-methyltransferase	12	0	0
IPR014786	Anaphase-promoting complex subunit 2 C-terminal	12	0	0
IPR014837	EF-hand Ca insensitive	12	0	0
IPR014848	Reduced growth phenotype protein 1	12	0	0
IPR014851	BCS1 N-terminal	12	0	0
IPR015009	Vinculin-binding site-containing domain	12	0	0
IPR015012	Phenylalanine zipper	12	0	0
IPR015187	BRCA2 oligonucleotide/oligosaccharide-binding 1	12	0	0

InterPro signature	InterPro description	Shared *Papaipema* genes	*Papaipema sp.4* SSGs	*Papaipema speciosissima* SSGs
IPR015324	Ribosomal protein Rsm22, bacterial-type	12	0	0
IPR015659	Proline oxidase family	12	0	0
IPR015661	Mitotic spindle checkpoint protein Bub1/Mad3	12	0	0
IPR015699	DNA-directed RNA pol I, largest subunit	12	0	0
IPR015761	Lipoamide Acyltransferase	12	0	0
IPR015848	Polyribonucleotide nucleotidyltransferase RNA-binding domain	12	0	0
IPR016208	Aldehyde oxidase/xanthine dehydrogenase	12	0	0
IPR016248	Fibroblast growth factor receptor family	12	0	0
IPR016591	Suppressor of fused, eukaryotic	12	0	0
IPR016812	Protein phosphatase methylesterase, eukaryotic	12	0	0
IPR016827	Transcriptional adaptor 2	12	0	0
IPR016858	Histone H4-K20 methyltransferase	12	0	0
IPR016900	Alpha-2-glucosyltransferase Alg10	12	0	0
IPR016965	Phosphatase PHOSPHO-type	12	0	0
IPR017375	Peroxisome assembly protein 12	12	0	0
IPR018094	Thymidylate kinase	12	0	0
IPR018144	Plus-3 domain subgroup	12	0	0
IPR018586	Brinker DNA-binding domain	12	0	0
IPR018619	Hyccin	12	0	0
IPR018627	Elongation complex protein 6	12	0	0

**InterPro signature**	**InterPro description**	**Shared *Papaipema* genes**	***Papaipema sp.4* SSGs**	***Papaipema speciosissima* SSGs**
IPR018801	Putative transmembrane protein precursor	12	0	0
IPR019137	Nck-associated protein 1	12	0	0
IPR019140	Mini-chromosome maintenance complex-binding protein	12	0	0
IPR019152	Protein of unknown function DUF2046	12	0	0
IPR019165	Peptidase M76, ATP23	12	0	0
IPR019175	Prp31 C-terminal	12	0	0
IPR019192	Ribosomal protein L28/L40, mitochondrial	12	0	0
IPR019321	Nucleoporin Nup88	12	0	0
IPR019324	M-phase phosphoprotein 6	12	0	0
IPR019365	TVP18/Calcium channel flower	12	0	0
IPR019374	Ribosomal protein S22, mitochondrial	12	0	0
IPR019379	Gamma-secretase aspartyl protease complex, presenilin enhancer-2 subunit	12	0	0
IPR019414	RNA polymerase II assembly factor Rtp1 C-terminal domain 2	12	0	0
IPR019495	Exosome complex component CSL4	12	0	0
IPR019498	MENTAL domain	12	0	0
IPR019516	Glomulin/ALF4	12	0	0
IPR019537	Transmembrane protein 65	12	0	0
IPR019554	Soluble ligand binding domain	12	0	0
IPR019575	NADH-ubiquinone oxidoreductase 51 kDa subunit iron-sulphur binding domain	12	0	0
IPR020040	Ribosomal protein L6 alpha-beta domain	12	0	0

InterPro signature	InterPro description	Shared *Papaipema* genes	*Papaipema sp.4* SSGs	*Papaipema speciosissima* SSGs
IPR020580	Glycine cleavage system P-protein N-terminal	12	0	0
IPR021726	THO complex subunitTHOC2 N-terminal	12	0	0
IPR021827	Nucleoporin Nup186/Nup192/Nup205	12	0	0
IPR021861	THO complex, subunit THOC1	12	0	0
IPR021930	Heparan sulphate-N-deacetylase	12	0	0
IPR021950	Transcription factor Spt20	12	0	0
IPR021983	PRP8 domain IV core	12	0	0
IPR022100	Minichromosome loss protein Mcl1 middle region	12	0	0
IPR022159	Tuftelin interacting protein N-terminal domain	12	0	0
IPR022165	Polo kinase kinase	12	0	0
IPR022418	Porphobilinogen deaminase C-terminal	12	0	0
IPR022617	Rad60/SUMO-like domain	12	0	0
IPR022816	Condensin complex subunit 2/barren	12	0	0
IPR023509	D-Tyr tRNAtyr deacylase-like domain	12	0	0
IPR023582	Impact family	12	0	0
IPR023601	Golgi SNAP receptor complex, subunit 1	12	0	0
IPR024158	Mitochondrial import protein TIM15	12	0	0
IPR024647	DNA polymerase alpha catalytic subunit N-terminal domain	12	0	0
IPR024670	B-cell lymphoma 9 beta-catenin binding domain	12	0	0
IPR024738	Transcriptional coactivator Hfi1/Transcriptional adapter 1	12	0	0

InterPro signature	InterPro description	Shared *Papaipema* genes	*Papaipema sp.4* SSGs	*Papaipema speciosissima* SSGs
IPR024779	2OGFeDO domain nucleic acid-modifying type	12	0	0
IPR024929	Nucleolar GTP-binding protein 2	12	0	0
IPR025602	BCP1 family	12	0	0
IPR025742	Cleavage stimulation factor subunit 2 hinge domain	12	0	0
IPR026064	Terminal deoxynucleotidyltransferase-interacting factor 1	12	0	0
IPR026070	Tyrosine-protein phosphatase CDC14	12	0	0
IPR026085	Activating transcription factor 7-interacting protein	12	0	0
IPR026183	Taxilin family	12	0	0
IPR026315	Out at first protein	12	0	0
IPR026316	KAT8 regulatory NSL complex subunit 2	12	0	0
IPR026317	Protein C10	12	0	0
IPR026505	Solute carrier family 35 member F3/F4	12	0	0
IPR026730	Mitochondrial inner membrane protease subunit 1	12	0	0
IPR026740	AP-3 complex subunit beta	12	0	0
IPR026807	Protein tiptop/teashirt	12	0	0
IPR027008	Teashirt family	12	0	0
IPR027038	Ran GTPase-activating protein	12	0	0
IPR027079	TFIIH subunit Tfb1/p62	12	0	0
IPR027095	Golgin-45	12	0	0
IPR027132	Structural maintenance of chromosomes protein 6	12	0	0

InterPro signature	InterPro description	Shared *Papaipema* genes	*Papaipema sp.4* SSGs	*Papaipema speciosissima* SSGs
IPR027243	Mitochondrial chaperone BCS1/plant AAA ATPases	12	0	0
IPR027307	WASH complex subunit 7	12	0	0
IPR027389	Beta-1,4-mannosyltransferase Bre-3/Egh	12	0	0
IPR027667	Actin-related protein 4	12	0	0
IPR028268	Pianissimo family	12	0	0
IPR028343	Fructose-1,6-bisphosphatase	12	0	0
IPR028361	GPI-anchor transamidase	12	0	0
IPR028426	Huntingtin family	12	0	0
IPR028564	tRNA methyltransferase TRM10-type domain	12	0	0
IPR028784	Bardet-Biedl syndrome 1 protein	12	0	0
IPR028916	Tox-GHH domain	12	0	0
IPR029014	NiFe hydrogenase-like	12	0	0
IPR029145	Neuroblastoma-amplified sequence N-terminal	12	0	0
IPR029201	Jiraiya	12	0	0
IPR029262	DNA-directed RNA polymerase N-terminal domain	12	0	0
IPR029415	Protein Lines C-terminal	12	0	0
IPR029451	Rapamycin-insensitive companion of mTOR middle domain	12	0	0
IPR029633	Calcyphosin-like protein	12	0	0
IPR030054	Actin-related protein 6	12	0	0
IPR030228	GPN-loop GTPase 3	12	0	0

**InterPro signature**	**InterPro description**	**Shared *Papaipema* genes**	***Papaipema sp.4* SSGs**	***Papaipema speciosissima* SSGs**
IPR030468	NEDD8-activating enzyme E1 catalytic subunit	12	0	0
IPR030523	SH2B adapter protein	12	0	0
IPR031102	RNA polymerase-associated protein Rtf1	12	0	0
IPR031366	Protein of unknown function DUF4663	12	0	0
IPR031953	mRNA-decapping enzyme C-terminal	12	0	0
IPR031993	Domain of unknown function DUF4789	12	0	0
IPR032010	Domain of unknown function DUF4793	12	0	0
IPR032041	Paf1 complex subunit Cdc73 N-terminal domain	12	0	0
IPR032055	Transmembrane protein 72	12	0	0
IPR032199	Recq-mediated genome instability protein 1 C-terminal OB-fold domain	12	0	0
IPR032263	ATP-citrate synthase citrate-binding domain	12	0	0
IPR032324	Clp1 N-terminal beta-sandwich domain	12	0	0
IPR032911	WD repeat domain phosphoinositide-interacting protein 2	12	0	0
IPR002113	Adenine nucleotide translocator 1	11	7	0
IPR000597	Ribosomal protein L3	11	5	1
IPR000941	Enolase	11	3	0
IPR021922	Domain of unknown function DUF3534	11	2	0
IPR001731	Delta-aminolevulinic acid dehydratase	11	0	2
IPR001950	Translation initiation factor SUI1	11	0	2
IPR005680	Ribosomal protein S23, eukaryotic/archaeal	11	2	0

**InterPro signature**	**InterPro description**	**Shared *Papaipema* genes**	***Papaipema sp.4* SSGs**	***Papaipema speciosissima* SSGs**
IPR006633	Carbohydrate-binding/sugar hydrolysis domain	11	2	0
IPR011876	Isopentenyl-diphosphate delta-isomerase, type 1	11	0	2
IPR019402	Frag1/DRAM/Sfk1	11	1	1
IPR029799	F-box only protein 11	11	0	2
IPR025956	Cytoplasmic dynein 1 intermediate chain 1/2	11	1	0
IPR000257	Uroporphyrinogen decarboxylase URO-D	11	1	0
IPR001474	GTP cyclohydrolase I	11	0	1
IPR001664	Intermediate filament protein	11	1	0
IPR002023	NADH-quinone oxidoreductase subunit E-like	11	0	1
IPR003140	Phospholipase/carboxylesterase/thioesterase	11	1	0
IPR003337	Trehalose-phosphatase	11	0	1
IPR004877	Cytochrome b561 domain	11	1	0
IPR005120	Regulator of nonsense-mediated decay UPF3	11	1	0
IPR005990	Inosine-5'-monophosphate dehydrogenase	11	1	0
IPR007064	NMD3	11	0	1
IPR007342	Pseudouridine-5'-phosphate glycosidase	11	0	1
IPR008547	Protein of unknown function DUF829, TMEM53	11	1	0
IPR010995	DNA repair Rad51/transcription factor NusA alpha-helical	11	0	1
IPR012879	Protein of unknown function DUF1682	11	0	1
IPR013602	Dynein heavy chain domain-2	11	1	0

**InterPro signature**	**InterPro description**	**Shared *Papaipema* genes**	***Papaipema sp.4* SSGs**	***Papaipema speciosissima* SSGs**
IPR013907	Sds3-like	11	1	0
IPR015310	Activator of Hsp90 ATPase N-terminal	11	0	1
IPR017978	GPCR family 3 C-terminal	11	0	1
IPR019136	Transcription factor IIIC, subunit 5	11	0	1
IPR019351	Protein of unknown function DUF2039	11	0	1
IPR019368	Ribosomal protein S23/S29, mitochondrial	11	0	1
IPR023571	Ribosomal protein L14 domain	11	0	1
IPR026151	Maspardin	11	0	1
IPR027992	Possible tRNA binding domain	11	0	1
IPR029473	Cell morphogenesis central region	11	0	1
IPR032353	Ubiquitin-protein ligase E3A N-terminal zinc-binding domain	11	1	0
IPR033010	The WD repeat Cdc20/Fizzy family	11	0	1
IPR000022	Carboxyl transferase	11	0	0
IPR000277	Cys/Met metabolism, pyridoxal phosphate-dependent enzyme	11	0	0
IPR000439	Ribosomal protein L15e	11	0	0
IPR000558	Histone H2B	11	0	0
IPR000715	Glycosyl transferase, family 4	11	0	0
IPR000945	Dopamine beta-hydroxylase-related	11	0	0
IPR000998	MAM domain	11	0	0
IPR001135	NADH-quinone oxidoreductase subunit D	11	0	0

InterPro signature	InterPro description	Shared *Papaipema* genes	*Papaipema sp.4* SSGs	*Papaipema speciosissima* SSGs
IPR001270	ClpA/B family	11	0	0
IPR001293	Zinc finger TRAF-type	11	0	0
IPR001398	Macrophage migration inhibitory factor	11	0	0
IPR001453	MoaB/Mog domain	11	0	0
IPR001576	Phosphoglycerate kinase	11	0	0
IPR001904	Paxillin	11	0	0
IPR002143	Ribosomal protein L1	11	0	0
IPR002229	Blood group Rhesus C/E/D polypeptide	11	0	0
IPR002550	Domain of unknown function DUF21	11	0	0
IPR002575	Aminoglycoside phosphotransferase	11	0	0
IPR002720	Retinoblastoma-associated protein A-box	11	0	0
IPR002737	MEMO1 family	11	0	0
IPR002765	Uncharacterised protein family UPF0145	11	0	0
IPR002836	PDCD5-related protein	11	0	0
IPR002908	Frataxin/CyaY	11	0	0
IPR002944	Sodium:neurotransmitter symporter, inebriated	11	0	0
IPR002994	Surfeit locus 1/Shy1	11	0	0
IPR003070	Orphan nuclear receptor	11	0	0
IPR003151	PIK-related kinase FAT	11	0	0
IPR003171	Methylenetetrahydrofolate reductase	11	0	0

**InterPro signature**	**InterPro description**	**Shared *Papaipema* genes**	***Papaipema sp.4* SSGs**	***Papaipema speciosissima* SSGs**
IPR003617	Transcription elongation factor TFIIS/CRSP70 N-terminal sub-type	11	0	0
IPR003782	Copper chaperone SCO1/SenC	11	0	0
IPR003812	Fido domain	11	0	0
IPR004033	UbiE/COQ5 methyltransferase	11	0	0
IPR004308	Glutamate-cysteine ligase catalytic subunit	11	0	0
IPR004385	Nucleoside diphosphate pyrophosphatase	11	0	0
IPR004524	Aspartate-tRNA ligase, bacterial/mitochondrial-type	11	0	0
IPR004649	Ribonuclease H2, subunit A	11	0	0
IPR004729	Transient receptor potential channel	11	0	0
IPR004870	Nucleoporin, Nup155-like	11	0	0
IPR005031	Coenzyme Q-binding protein COQ10 START domain	11	0	0
IPR005147	tRNA synthetase B5-domain	11	0	0
IPR005195	Glycoside hydrolase family 65 central catalytic	11	0	0
IPR005251	Methylthioribose-1-phosphate isomerase	11	0	0
IPR005343	Nucleolar complex protein 2	11	0	0
IPR005542	PBX	11	0	0
IPR005703	Ribosomal protein S3, eukaryotic/archaeal	11	0	0
IPR005710	Ribosomal protein S4/S9, eukaryotic/archaeal	11	0	0
IPR005764	Adenine phosphoribosyl transferase	11	0	0
IPR005766	Delta l-pyrroline-5-carboxylate synthetase	11	0	0

InterPro signature	InterPro description	Shared *Papaipema* genes	*Papaipema sp.4* SSGs	*Papaipema speciosissima* SSGs
IPR005854	Amidophosphoribosyltransferase	11	0	0
IPR005880	Ribosomal protein L2, bacterial/organellar-type	11	0	0
IPR005910	Histone acetyltransferase ELP3	11	0	0
IPR005958	Tyrosine/nicotianamine aminotransferase	11	0	0
IPR006056	RidA family	11	0	0
IPR006070	YrdC-like domain	11	0	0
IPR006096	Glutamate/phenylalanine/leucine/valine dehydrogenase C-terminal	11	0	0
IPR006165	Ku70	11	0	0
IPR006255	Dihydrolipoamide succinyltransferase	11	0	0
IPR006384	Pyridoxal phosphate phosphatase-related	11	0	0
IPR006413	P-type ATPase, subfamily IIA, PMR1-type	11	0	0
IPR006434	Pyrimidine 5'-nucleotidase, eukaryotic	11	0	0
IPR006775	Glucosylceramidase	11	0	0
IPR006786	Pinin/SDK/MemA protein	11	0	0
IPR006846	Ribosomal protein S30	11	0	0
IPR006982	Glutamate synthase central-N	11	0	0
IPR007005	XAP5 protein	11	0	0
IPR007009	SHQ1 protein	11	0	0
IPR007051	CHORD domain	11	0	0
IPR007133	RNA polymerase II associated factor Paf1	11	0	0

**InterPro signature**	**InterPro description**	**Shared *Papaipema* genes**	***Papaipema sp.4* SSGs**	***Papaipema speciosissima* SSGs**
IPR007149	Leo1-like protein	11	0	0
IPR007175	RNAse P, Rpr2/Rpp21 subunit	11	0	0
IPR007196	CCR4-Not complex component Not1 C-terminal	11	0	0
IPR007205	Protein HGH1 N-terminal	11	0	0
IPR007235	Glycosyl transferase family 28 C-terminal	11	0	0
IPR007277	Transmembrane adaptor Erv26	11	0	0
IPR007705	Vesicle transport v-SNARE N-terminal	11	0	0
IPR007785	Anamorsin	11	0	0
IPR007854	Pre-mRNA polyadenylation factor Fip1	11	0	0
IPR007859	Electron transfer flavoprotein-ubiquinone oxidoreductase	11	0	0
IPR007873	Glycosyltransferase, ALG3	11	0	0
IPR008010	Membrane protein,Tapt1/CMV receptor	11	0	0
IPR008373	Saposin	11	0	0
IPR008395	Agenet-like domain	11	0	0
IPR008493	Domain of unknown function DUF775	11	0	0
IPR008518	FATE/Miff/Tango-11	11	0	0
IPR008594	Scavenger mRNA decapping enzyme DcpS/DCS2	11	0	0
IPR008721	Origin recognition complex, subunit 6	11	0	0
IPR008828	TORC2 component Sin1/Avo1	11	0	0
IPR008926	Ribonucleotide reductase R1 subunit N-terminal	11	0	0

**InterPro signature**	**InterPro description**	**Shared *Papaipema* genes**	***Papaipema sp.4* SSGs**	***Papaipema speciosissima* SSGs**
IPR009029	Hydroxymethylglutaryl-CoA reductase class I/II substrate-binding domain	11	0	0
IPR009062	Smac/DIABLO-like	11	0	0
IPR009076	Rapamycin-binding domain	11	0	0
IPR009551	Protein wntless	11	0	0
IPR009600	GPI transamidase subunit PIG-U	11	0	0
IPR009622	NADH dehydrogenase [ubiquinone] 1 alpha subcomplex assembly factor 4	11	0	0
IPR009668	RNA polymerase I associated factor, A49-like	11	0	0
IPR009787	Protein jagunal	11	0	0
IPR010014	Diphthamide synthesis DHP2, eukaryotic	11	0	0
IPR010044	Methylthioadenosine phosphorylase MTAP)	11	0	0
IPR010422	Coiled-coil domain-containing protein 124	11	0	0
IPR010431	Fascin	11	0	0
IPR010492	GINS complex, subunit Psf3	11	0	0
IPR010591	ATP11	11	0	0
IPR010742	Rab5-interacting protein	11	0	0
IPR010750	SGF29 tudor-like domain	11	0	0
IPR010793	Ribosomal protein L37/S30	11	0	0
IPR011023	Nop2p	11	0	0
IPR011145	Scavenger mRNA decapping enzyme N-terminal	11	0	0
IPR011236	Serine/threonine protein phosphatase 5	11	0	0

**InterPro signature**	**InterPro description**	**Shared *Papaipema* genes**	***Papaipema sp.4* SSGs**	***Papaipema speciosissima* SSGs**
IPR011502	Nucleoporin Nup85-like	11	0	0
IPR011537	NADH ubiquinone oxidoreductase, F subunit	11	0	0
IPR011559	Initiation factor 2B alpha/beta/delta	11	0	0
IPR011566	Ubiquinone biosynthesis protein Coq7	11	0	0
IPR011760	Pseudouridine synthase TruD insertion domain	11	0	0
IPR011778	Hydantoinase/dihydropyrimidinase	11	0	0
IPR011987	ATPase V1 complex subunit H C-terminal	11	0	0
IPR012255	Electron transfer flavoprotein, beta subunit	11	0	0
IPR012459	Protein of unknown function DUF1665	11	0	0
IPR012492	Protein RED C-terminal	11	0	0
IPR012590	POPLD	11	0	0
IPR012875	Protein of unknown function DUF1674	11	0	0
IPR012929	Nucleoprotein TPR/MLP1	11	0	0
IPR012958	CHD N-terminal	11	0	0
IPR012973	NOG C-terminal	11	0	0
IPR013005	50S ribosomal protein uL4	11	0	0
IPR013212	Mad3/Bub1 homology region 1	11	0	0
IPR013235	PPP domain	11	0	0
IPR013256	Chromatin SPT2	11	0	0
IPR013346	Ribonucleotide reductase class I alpha subunit	11	0	0

InterPro signature	InterPro description	Shared *Papaipema* genes	*Papaipema sp.4* SSGs	*Papaipema speciosissima* SSGs
IPR013509	Ribonucleotide reductase large subunit N-terminal	11	0	0
IPR013877	YAP-binding/ALF4/Glomulin	11	0	0
IPR013961	RAI1-like	11	0	0
IPR014027	UDP-glucose/GDP-mannose dehydrogenase C-terminal	11	0	0
IPR014387	CDP-diacylglycerol-inositol 3-phosphatidyltransferase, eukaryote	11	0	0
IPR014445	Glutamine-dependent NAD+ synthetase	11	0	0
IPR014732	Orotidine 5'-phosphate decarboxylase	11	0	0
IPR014767	Diaphanous autoregulatory DAD domain	11	0	0
IPR014816	tRNA 1-methyladenosine methyltransferase catalytic subunit Gcd14	11	0	0
IPR014881	Nin one binding NOB1 Zn-ribbon-like	11	0	0
IPR014929	E2 binding	11	0	0
IPR015257	Repressor of RNA polymerase III transcription Maf1	11	0	0
IPR015377	Fumarylacetoacetase N-terminal	11	0	0
IPR015381	XLF family	11	0	0
IPR015429	Cyclin L/T	11	0	0
IPR015803	Cysteine-tRNA ligase	11	0	0
IPR016341	Clathrin, heavy chain	11	0	0
IPR016589	tRNA-splicing endonuclease, SEN2 subunit	11	0	0
IPR016615	Ubiquitin thioesterase Otubain	11	0	0
IPR016657	Phosphoacetylglucosamine mutase	11	0	0

InterPro signature	InterPro description	Shared *Papaipema* genes	*Papaipema sp.4* SSGs	*Papaipema speciosissima* SSGs
IPR016967	Splicing factor, SPF45	11	0	0
IPR017061	DNA polymerase eta/kappa	11	0	0
IPR017117	D-site 20S pre-rRNA nuclease Nob1	11	0	0
IPR017383	Actin-related protein 2/3 complex subunit 1	11	0	0
IPR017390	Ubiquitinyl hydrolase, UCH37 type	11	0	0
IPR017476	UDP-glucose/GDP-mannose dehydrogenase	11	0	0
IPR017789	Frataxin	11	0	0
IPR017939	Gamma-glutamylcyclotransferase	11	0	0
IPR017945	DHBP synthase RibB-like alpha/beta domain	11	0	0
IPR018167	S-adenosylmethionine decarboxylase subgroup	11	0	0
IPR018305	Ribosomal protein L50, mitochondria	11	0	0
IPR018379	BEN domain	11	0	0
IPR018514	Rabaptin coiled-coil domain	11	0	0
IPR018545	Btz domain	11	0	0
IPR018864	Nucleoporin Nup188	11	0	0
IPR018867	Cell division protein borealin	11	0	0
IPR018908	Putative transmembrane family 234	11	0	0
IPR019024	Ribonuclease H2, subunit B	11	0	0
IPR019138	De-etiolated protein 1, Det1	11	0	0
IPR019150	Vesicle transport protein, Use1	11	0	0

InterPro signature	InterPro description	Shared *Papaipema* genes	*Papaipema sp.4* SSGs	*Papaipema speciosissima* SSGs
IPR019163	THO complex, subunit 5	11	0	0
IPR019337	Telomere length regulation protein conserved domain	11	0	0
IPR019356	Protein of unknown function DUF2181	11	0	0
IPR019376	Myeloid leukemia factor	11	0	0
IPR019400	Peptidase C65, otubain	11	0	0
IPR019461	Autophagy-related protein 3 C-terminal	11	0	0
IPR019496	Nuclear fragile X mental retardation-interacting protein 1, conserved domain	11	0	0
IPR019558	Mammalian uncoordinated homology 13 subgroup domain 2	11	0	0
IPR019564	Mitochondrial outer membrane transport complex Sam37/metaxin N-terminal domain	11	0	0
IPR020456	Acylphosphatase	11	0	0
IPR020529	Origin recognition complex, subunit 6, metazoa/plant	11	0	0
IPR020562	Phosphoribosylglycinamide synthetase N-terminal	11	0	0
IPR020825	Phenylalanyl-tRNA synthetase B3/B4	11	0	0
IPR021019	Mediator complex subunit Med30 metazoa	11	0	0
IPR021567	Lens epithelium-derived growth factor LEDGF)	11	0	0
IPR021713	Folliculin	11	0	0
IPR021757	Ribosomal protein L46	11	0	0
IPR021987	Protein of unknown function DUF3588	11	0	0
IPR022035	Phosphorylated CTD interacting factor 1 WW domain	11	0	0
IPR022043	Chromatin assembly factor 1 subunit A	11	0	0

InterPro signature	InterPro description	Shared *Papaipema* genes	*Papaipema sp.4* SSGs	*Papaipema speciosissima* SSGs
IPR022127	Store-operated calcium entry regulator STIMATE	11	0	0
IPR022145	Domain of unknown function DUF3677	11	0	0
IPR022181	Bcl2-/adenovirus E1B 19kDa-interacting protein 2	11	0	0
IPR022417	Porphobilinogen deaminase N-terminal	11	0	0
IPR022533	Cox20/FAM36A	11	0	0
IPR022768	Fascin domain	11	0	0
IPR022786	Geminin/Multicilin	11	0	0
IPR023242	FAM36A	11	0	0
IPR023252	Aurora borealis protein	11	0	0
IPR023370	TsaA-like domain	11	0	0
IPR024119	Transcription factor DEAF-1	11	0	0
IPR024337	tRNA-splicing endonuclease, subunit Sen54	11	0	0
IPR024371	Acetyl-coenzyme A transporter 1	11	0	0
IPR024395	CLASP N-terminal domain	11	0	0
IPR024574	Domain of unknown function DUF3361	11	0	0
IPR024582	Limkain b1 conserved domain	11	0	0
IPR024657	COMPASS complex Set1 subunit N-SET domain	11	0	0
IPR024687	MMS19 C-terminal	11	0	0
IPR024790	Anaphase-promoting complex subunit 4 long domain	11	0	0
IPR025848	mRNA 2-O-methyladenosine-N6)-)-methyltransferase	11	0	0

InterPro signature	InterPro description	Shared *Papaipema* genes	*Papaipema sp.4* SSGs	*Papaipema speciosissima* SSGs
IPR025946	CABIT domain	11	0	0
IPR025986	RNA-polymerase II-associated protein 3-like C-terminal domain	11	0	0
IPR026040	Hydroxypyruvate isomerase-like	11	0	0
IPR026109	G kinase-anchoring protein 1	11	0	0
IPR026224	Protein DPCD	11	0	0
IPR026299	Mitochondrial 28S ribosomal protein S31	11	0	0
IPR026515	ARF14 effector protein	11	0	0
IPR026642	Glucocorticoid-induced transcript 1/FAM117	11	0	0
IPR026748	Clarin	11	0	0
IPR026872	Protein farnesyltransferase subunit beta	11	0	0
IPR027027	GOSR2/Membrin/Bos1	11	0	0
IPR027059	Coatomer delta subunit	11	0	0
IPR027081	CyclinH/Ccl1	11	0	0
IPR027104	U4/U6 small nuclear ribonucleoprotein Prp3	11	0	0
IPR027111	Mitochondrial import inner membrane translocase subunit Tim50	11	0	0
IPR027165	Condensin complex subunit 3	11	0	0
IPR027193	Nucleolar complex protein 4	11	0	0
IPR027512	Eukaryotic translation initiation factor 3 subunit A	11	0	0
IPR027674	Exostosin 3/Exostosin-like 3	11	0	0
IPR027711	Rmd5	11	0	0

InterPro signature	InterPro description	Shared *Papaipema* genes	*Papaipema sp.4* SSGs	*Papaipema speciosissima* SSGs
IPR027967	Protein of unknown function DUF4612	11	0	0
IPR027973	Protein of unknown function DUF4602	11	0	0
IPR028031	Domain of unknown function DUF4460	11	0	0
IPR028086	Folliculin-interacting protein C-terminal domain	11	0	0
IPR028091	FAM91 N-terminal domain	11	0	0
IPR028174	Fibroblast growth factor receptor 1	11	0	0
IPR028213	PTIP-associated protein 1	11	0	0
IPR028309	Retinoblastoma protein family	11	0	0
IPR028544	Protein CASC3	11	0	0
IPR028896	Aminomethyltransferase/Dimethylsulfonioproprionate demethylase DmdA	11	0	0
IPR029004	Ribosomal L28e/Mak16	11	0	0
IPR029205	Aftiphilin clathrin-binding box	11	0	0
IPR029207	FAM198 family	11	0	0
IPR029209	DML1/Misato tubulin domain	11	0	0
IPR029264	ARF7 effector protein C-terminal	11	0	0
IPR029332	PEHE domain	11	0	0
IPR029419	Argininosuccinate lyase C-terminal	11	0	0
IPR029453	Rapamycin-insensitive companion of mTOR domain 4	11	0	0
IPR029616	Protein winged eye	11	0	0
IPR029685	Structural maintenance of chromosomes protein 3	11	0	0

**InterPro signature**	**InterPro description**	**Shared *Papaipema* genes**	***Papaipema sp.4* SSGs**	***Papaipema speciosissima* SSGs**
IPR029925	Uridine-cytidine kinase 2	11	0	0
IPR029929	Growth arrest-specific protein 2	11	0	0
IPR030092	SWI/SNFcomplex subunit SMARCC2	11	0	0
IPR030231	GPN-loop GTPase 2	11	0	0
IPR030298	Ubiquitin thioesterase OTUB1	11	0	0
IPR030382	SAM-dependent methyltransferase TRM5/TYW2-type	11	0	0
IPR030662	Diphthine--ammonia ligase/Uncharacterised protein MJ0570	11	0	0
IPR031317	Tom37 C-terminal domain	11	0	0
IPR031318	OPI10 family	11	0	0
IPR031330	Glycoside hydrolase 35 catalytic domain	11	0	0
IPR031600	Domain of unknown function DUF4706	11	0	0
IPR031984	Solute carrier family 3 member 2 N-terminal domain	11	0	0
IPR032008	Domain of unknown function DUF4792	11	0	0
IPR032064	Protein of unknown function DUF4805	11	0	0
IPR032429	Nibrin second BRCT domain	11	0	0
IPR032469	Endophilin-A3	11	0	0
IPR032486	JNK-interacting protein leucine zipper II	11	0	0
IPR032830	Helicase XPB/Ssl2 N-terminal domain	11	0	0
IPR033100	Exosome complex component RRP45	11	0	0
IPR010468	Hormone-sensitive lipase N-terminal	10	4	0

**InterPro signature**	**InterPro description**	**Shared *Papaipema* genes**	***Papaipema sp.4* SSGs**	***Papaipema speciosissima* SSGs**
IPR018028	Catalase, mono-functional, haem-containing	10	4	0
IPR020835	Catalase-like domain	10	0	4
IPR002784	Ribosomal protein L14	10	2	1
IPR002938	FAD-binding domain	10	0	3
IPR011614	Catalase core domain	10	0	3
IPR023573	Ribosomal protein L18a/LX	10	1	2
IPR000131	ATPase, F1 complex, gamma subunit	10	0	2
IPR002241	Glycoside hydrolase, family 27	10	2	0
IPR002587	Myo-inositol-1-phosphate synthase	10	0	2
IPR008709	Neurochondrin	10	2	0
IPR009040	Ferritin- like diiron domain	10	0	2
IPR010582	Catalase immune-responsive domain	10	0	2
IPR023633	ATPase F1 complex gamma subunit domain	10	2	0
IPR018902	Uncharacterised protein family UPF0573/UPF0605	10	1	0
IPR019193	Ubiquitin-conjugating enzyme E2-binding protein	10	1	0
IPR031420	UPF0669 protein C6orf120	10	0	1
IPR013594	Dynein heavy chain domain-1	10	0	1
IPR013845	Ribosomal protein S4e central region	10	0	1
IPR000181	Formylmethionine deformylase	10	0	1
IPR000367	G-protein alpha subunit, group S	10	0	1

InterPro signature	InterPro description	Shared *Papaipema* genes	*Papaipema sp.4* SSGs	*Papaipema speciosissima* SSGs
IPR000580	TSC-22 / Dip / Bun	10	1	0
IPR000876	Ribosomal protein S4e	10	1	0
IPR001638	Solute-binding protein family 3/N-terminal domain of MltF	10	1	0
IPR003508	CIDE-N domain	10	1	0
IPR004203	Cytochrome c oxidase subunit IV family	10	1	0
IPR004947	Deoxyribonuclease II	10	0	1
IPR004977	Ribosomal protein S25	10	1	0
IPR006614	Peroxin/Ferlin domain	10	0	1
IPR006906	Timeless protein	10	1	0
IPR007587	SIT4 phosphatase-associated protein family	10	1	0
IPR008092	Ribosomal protein S29, mitochondrial	10	0	1
IPR008610	Eukaryotic rRNA processing	10	1	0
IPR008859	Thrombospondin C-terminal	10	0	1
IPR009073	Co-chaperone HscB C-terminal oligomerisation domain	10	1	0
IPR009110	Nuclear receptor coactivator interlocking	10	0	1
IPR010945	Malate dehydrogenase, type 2	10	1	0
IPR012983	PHR	10	1	0
IPR013288	Cytochrome c oxidase subunit IV	10	1	0
IPR013906	Eukaryotic translation initiation factor 3 subunit J	10	1	0
IPR014744	Nuclear receptor coactivator CREB-bp-like interlocking	10	0	1

InterPro signature	InterPro description	Shared *Papaipema* genes	*Papaipema sp.4* SSGs	*Papaipema speciosissima* SSGs
IPR016443	RNA 3'-terminal phosphate cyclase type 2	10	1	0
IPR020811	Enolase N-terminal	10	0	1
IPR023471	Cytochrome c oxidase assembly protein CtaG/Cox11 domain	10	1	0
IPR027377	Zinc-binding domain	10	0	1
IPR027468	Alpha-dystroglycan domain 2	10	1	0
IPR027524	Eukaryotic translation initiation factor 3 subunit H	10	1	0
IPR028433	Parvin	10	1	0
IPR030225	Sterol regulatory element-binding protein cleavage-activating protein	10	1	0
IPR030481	Staphylococcal nuclease domain-containing protein 1	10	1	0
IPR030702	Ras-related protein Rab14	10	0	1
IPR000238	Ribosome-binding factor A	10	0	0
IPR000361	FeS cluster biogenesis	10	0	0
IPR000415	Nitroreductase-like	10	0	0
IPR000700	PAS-associated C-terminal	10	0	0
IPR000740	GrpE nucleotide exchange factor	10	0	0
IPR000895	Transthyretin/hydroxyisourate hydrolase	10	0	0
IPR000911	Ribosomal protein L11/L12	10	0	0
IPR000969	Structure-specific recognition protein	10	0	0
IPR000979	Phosphodiesterase MJ0936/Vps29	10	0	0
IPR001114	Adenylosuccinate synthetase	10	0	0

InterPro signature	InterPro description	Shared *Papaipema* genes	*Papaipema sp.4* SSGs	*Papaipema speciosissima* SSGs
IPR001170	Adenylyl cyclase class-4/guanylyl cyclase	10	0	0
IPR001250	Mannose-6-phosphate isomerase, type I	10	0	0
IPR001266	Ribosomal protein S19e	10	0	0
IPR001268	NADH:ubiquinone oxidoreductase 30kDa subunit	10	0	0
IPR001368	TNFR/NGFR cysteine-rich region	10	0	0
IPR001473	Clathrin heavy chain propeller N-terminal	10	0	0
IPR001717	Anion exchange protein	10	0	0
IPR001857	Ribosomal protein L19	10	0	0
IPR001951	Histone H4	10	0	0
IPR002042	Uricase	10	0	0
IPR002189	F-actin-capping protein subunit alpha	10	0	0
IPR002205	DNA topoisomerase type IIA subunit A/C-terminal	10	0	0
IPR002298	DNA polymerase A	10	0	0
IPR002347	Short-chain dehydrogenase/reductase SDR	10	0	0
IPR002374	cGMP-dependent kinase	10	0	0
IPR002699	ATPase, V1 complex, subunit D	10	0	0
IPR002731	ATPase BadF/BadG/BcrA/BcrD type	10	0	0
IPR002759	Ribonuclease P/MRP protein subunit	10	0	0
IPR002761	Diphthamide synthase domain	10	0	0
IPR002773	Deoxyhypusine synthase	10	0	0

InterPro signature	InterPro description	Shared *Papaipema* genes	*Papaipema sp.4* SSGs	*Papaipema speciosissima* SSGs
IPR002853	Transcription factor TFIIE, alpha subunit	10	0	0
IPR002921	Fungal lipase-like domain	10	0	0
IPR002995	Surfeit locus 4	10	0	0
IPR003410	HYR domain	10	0	0
IPR003697	Maf-like protein	10	0	0
IPR003702	Acetyl-CoA hydrolase/transferase	10	0	0
IPR003726	Homocysteine-binding domain	10	0	0
IPR003737	N-acetylglucosaminyl phosphatidylinositol deacetylase-related	10	0	0
IPR003750	Putative RNA methyltransferase	10	0	0
IPR003764	N-acetylglucosamine-6-phosphate deacetylase	10	0	0
IPR003837	Glu-tRNAGln amidotransferase C subunit	10	0	0
IPR003923	Transcription initiation factor TFIID, 23-30kDa subunit	10	0	0
IPR003994	Ubiquitously expressed transcript protein UXT	10	0	0
IPR004104	Oxidoreductase C-terminal	10	0	0
IPR004342	EXS C-terminal	10	0	0
IPR004405	Translation release factor pelota	10	0	0
IPR004468	CTP synthase	10	0	0
IPR004526	Glutamyl-tRNA synthetase, archaeal/eukaryotic cytosolic	10	0	0
IPR004531	Phenylalanyl-tRNA synthetase, class IIc, beta subunit, archae/euk cytosolic	10	0	0
IPR004575	Cdk-activating kinase assembly factor MAT1/Tfb3	10	0	0

InterPro signature	InterPro description	Shared *Papaipema* genes	*Papaipema sp.4* SSGs	*Papaipema speciosissima* SSGs
IPR004595	TFIIH C1-like domain	10	0	0
IPR004640	Co-chaperone Hsc20	10	0	0
IPR004854	Ubiquitin fusion degradation protein UFD1	10	0	0
IPR004971	mRNA guanine-N7-methyltransferase domain	10	0	0
IPR005013	Dolichyl-diphosphooligosaccharide--protein glycosyltransferase 48 kDa subunit	10	0	0
IPR005061	Vacuolar protein sorting-associated protein Ist1	10	0	0
IPR005144	ATP-cone domain	10	0	0
IPR005160	Ku70/Ku80 C-terminal arm	10	0	0
IPR005240	Protein of unknown function DUF389	10	0	0
IPR005317	Dipeptidyl-peptidase 3	10	0	0
IPR005474	Transketolase N-terminal	10	0	0
IPR005578	Yif1 family	10	0	0
IPR005719	Dihydroorotate dehydrogenase, class 2	10	0	0
IPR005727	Ribosomal protein L22, bacterial/chloroplast-type	10	0	0
IPR005744	Hly-III	10	0	0
IPR005749	Ribosomal protein L15, bacterial-type	10	0	0
IPR005952	Phosphoglycerate mutase 1	10	0	0
IPR005955	Maleylacetoacetate isomerase	10	0	0
IPR005956	4-hydroxyphenylpyruvate dioxygenase	10	0	0
IPR006137	NADH:ubiquinone oxidoreductase-like 20kDa subunit	10	0	0

InterPro signature	InterPro description	Shared *Papaipema* genes	*Papaipema sp.4* SSGs	*Papaipema speciosissima* SSGs
IPR006138	NADH-ubiquinone oxidoreductase, 20 Kd subunit	10	0	0
IPR006225	Pseudouridine synthase, RluC/RluD	10	0	0
IPR006232	Sucrose-6-phosphate hydrolase	10	0	0
IPR006252	Malate synthase A	10	0	0
IPR006338	Thioredoxin/glutathione reductase selenoprotein	10	0	0
IPR006361	Uroporphyrinogen decarboxylase HemE	10	0	0
IPR006370	4-hydroxybenzoate polyprenyltransferase	10	0	0
IPR006393	Sepiapterin reductase	10	0	0
IPR006424	Glyceraldehyde-3-phosphate dehydrogenase, type I	10	0	0
IPR006674	HD domain	10	0	0
IPR006694	Fatty acid hydroxylase	10	0	0
IPR006708	Pex19 protein	10	0	0
IPR006809	TAFII28-like protein	10	0	0
IPR006822	Coatomer, epsilon subunit	10	0	0
IPR006840	ChaC-like protein	10	0	0
IPR006916	Popeye protein	10	0	0
IPR007062	Protein phosphatase inhibitor 2 IPP-2)	10	0	0
IPR007115	6-pyruvoyl tetrahydropterin synthase/QueD family protein	10	0	0
IPR007134	Autophagy-related protein 3 N-terminal	10	0	0
IPR007145	Microtubule-associated protein, MAP65/Ase1/PRC1	10	0	0

InterPro signature	InterPro description	Shared *Papaipema* genes	*Papaipema sp.4* SSGs	*Papaipema speciosissima* SSGs
IPR007177	Ribosome biogenesis protein C-terminal	10	0	0
IPR007180	Domain of unknown function DUF382	10	0	0
IPR007193	Up-frameshift suppressor 2	10	0	0
IPR007206	Protein HGH1 C-terminal	10	0	0
IPR007234	Vps53-like N-terminal	10	0	0
IPR007239	Autophagy-related protein 5	10	0	0
IPR007265	Conserved oligomeric Golgi complex, subunit 3	10	0	0
IPR007472	Arginine-tRNA-protein transferase C-terminal	10	0	0
IPR007515	Mss4	10	0	0
IPR007594	RFT1	10	0	0
IPR007657	Glycosyltransferase AER61, uncharacterised	10	0	0
IPR007725	Timeless C-terminal	10	0	0
IPR007783	Eukaryotic translation initiation factor 3 subunit D	10	0	0
IPR007875	Sprouty	10	0	0
IPR007992	Succinate dehydrogenase [ubiquinone] cytochrome b small subunit, CybS	10	0	0
IPR008081	Cytoplasmic FMR1-interacting	10	0	0
IPR008146	Glutamine synthetase catalytic domain	10	0	0
IPR008148	DNA photolyase class 2	10	0	0
IPR008278	4'-phosphopantetheinyl transferase superfamily	10	0	0
IPR008331	Ferritin/DPS protein domain	10	0	0

**InterPro signature**	**InterPro description**	**Shared *Papaipema* genes**	***Papaipema sp.4* SSGs**	***Papaipema speciosissima* SSGs**
IPR008352	Mitogen-activated protein MAP kinase, p38	10	0	0
IPR008381	ACN9	10	0	0
IPR008409	Pre-mRNA-splicing factor SPF27	10	0	0
IPR008491	Protein of unknown function DUF773	10	0	0
IPR008584	Protein of unknown function DUF866, eukaryotic	10	0	0
IPR008590	Protein of unknown function DUF872, transmembrane	10	0	0
IPR008656	Inositol-tetrakisphosphate 1-kinase	10	0	0
IPR008689	ATPase, F0 complex, subunit D, mitochondrial	10	0	0
IPR008814	Dolichyl-diphosphooligosaccharide--protein glycosyltransferase subunit Swp1	10	0	0
IPR008913	Zinc finger CHY-type	10	0	0
IPR009006	Alanine racemase/group IV decarboxylase C-terminal	10	0	0
IPR009023	Hydroxymethylglutaryl-CoA reductase class I/II NAD/NADP-binding domain	10	0	0
IPR009109	Ran-GTPase activating protein 1 C-terminal	10	0	0
IPR009349	Zinc finger C2HC5-type	10	0	0
IPR009360	Isy1-like splicing	10	0	0
IPR009448	UDP-glucose:Glycoprotein Glucosyltransferase	10	0	0
IPR009464	PCAF N-terminal	10	0	0
IPR009801	Transmembrane protein 126	10	0	0
IPR009917	Steroid receptor RNA activator-protein/coat protein complex II, Sec31	10	0	0
IPR010218	NADH dehydrogenase, subunit C	10	0	0

InterPro signature	InterPro description	Shared *Papaipema* genes	*Papaipema sp.4* SSGs	*Papaipema speciosissima* SSGs
IPR010301	Nucleolar, Nop52	10	0	0
IPR010405	Cofactor of BRCA1	10	0	0
IPR010482	Peroxin domain	10	0	0
IPR010585	DNA repair protein XRCC4	10	0	0
IPR010655	Pre-mRNA cleavage complex subunit Clp1	10	0	0
IPR010684	RNA polymerase II transcription factor SIII, subunit A	10	0	0
IPR010711	Group XII secretory phospholipase A2 precursor	10	0	0
IPR010714	Coatomer alpha subunit C-terminal	10	0	0
IPR010921	Trp repressor/replication initiator	10	0	0
IPR011033	PRC-barrel-like	10	0	0
IPR011304	L-lactate dehydrogenase	10	0	0
IPR011400	Eukaryotic translation initiation factor 3 subunit B	10	0	0
IPR011641	Tyrosine-protein kinase ephrin type A/B receptor-like	10	0	0
IPR011671	tRNA uracil-O2)-)-methyltransferase	10	0	0
IPR011762	Acetyl-coenzyme A carboxyltransferase N-terminal	10	0	0
IPR011766	Thiamine pyrophosphate enzyme C-terminal TPP-binding	10	0	0
IPR011877	Ribokinase, bacterial	10	0	0
IPR012013	Integrin beta-4 subunit	10	0	0
IPR012251	N-acetylglucosamine-6-sulfatase	10	0	0
IPR012315	KASH domain	10	0	0

**InterPro signature**	**InterPro description**	**Shared *Papaipema* genes**	***Papaipema sp.4* SSGs**	***Papaipema speciosissima* SSGs**
IPR012479	SAP30-binding protein	10	0	0
IPR012485	Centromere protein I	10	0	0
IPR012942	Sensitivity To Red Light Reduced-like SRR1	10	0	0
IPR012952	BING4 C-terminal domain	10	0	0
IPR012982	PADR1 domain	10	0	0
IPR013132	N-acetylneuraminic acid synthase N-terminal	10	0	0
IPR013234	PIGA GPI anchor biosynthesis	10	0	0
IPR013535	PUL domain	10	0	0
IPR013538	Activator of Hsp90 ATPase homologue 1-like	10	0	0
IPR013568	SEFIR domain	10	0	0
IPR013579	FAST kinase-like protein subdomain 2	10	0	0
IPR013583	Phosphoribosyltransferase C-terminal	10	0	0
IPR013637	Lysine-specific demethylase-like domain	10	0	0
IPR013684	Mitochondrial Rho-like	10	0	0
IPR013699	Signal recognition particle SRP72 subunit RNA-binding	10	0	0
IPR013730	Fyv7/TAP26	10	0	0
IPR013760	DNA topoisomerase type IIA-like domain	10	0	0
IPR013861	Transmembrane protein DUF1751, eukaryotic	10	0	0
IPR013865	Protein of unknown function DUF1754, eukaryotic	10	0	0
IPR013870	Ribosomal protein L37, mitochondrial	10	0	0

InterPro signature	InterPro description	Shared *Papaipema* genes	*Papaipema sp.4* SSGs	*Papaipema speciosissima* SSGs
IPR013878	Mo25-like	10	0	0
IPR013883	Transcription factor Iwr1	10	0	0
IPR013892	Cytochrome c oxidase biogenesis protein Cmc1-like	10	0	0
IPR013921	Mediator complex, subunit Med20	10	0	0
IPR013932	TATA-binding protein interacting TIP20	10	0	0
IPR013949	U3 small nucleolar RNA-associated protein 6	10	0	0
IPR013954	Polynucleotide kinase 3 phosphatase	10	0	0
IPR013969	Oligosaccharide biosynthesis protein Alg14-like	10	0	0
IPR014026	UDP-glucose/GDP-mannose dehydrogenase dimerisation	10	0	0
IPR014306	Hydroxyisourate hydrolase	10	0	0
IPR014646	Replication factor A protein 2	10	0	0
IPR014797	CKK domain	10	0	0
IPR014824	Scaffold protein Nfu/NifU N-terminal	10	0	0
IPR014892	Replication protein A C-terminal	10	0	0
IPR015188	BRCA2 oligonucleotide/oligosaccharide-binding 3	10	0	0
IPR015348	Clathrin heavy chain linker core motif	10	0	0
IPR015350	Beta-trefoil DNA-binding domain	10	0	0
IPR015362	WIBG family	10	0	0
IPR015411	Replication factor Mcm10 C-terminal	10	0	0
IPR015576	Spermine synthase, animal	10	0	0

**InterPro signature**	**InterPro description**	**Shared *Papaipema* genes**	***Papaipema sp.4* SSGs**	***Papaipema speciosissima* SSGs**
IPR015789	Twist-related	10	0	0
IPR015877	Cdk-activating kinase assembly factor MAT1 centre	10	0	0
IPR016009	tRNA methyltransferase TRMD/TRM10-type domain	10	0	0
IPR016300	Arsenical pump ATPase, ArsA/GET3	10	0	0
IPR016305	Mannose-6-phosphate isomerase	10	0	0
IPR016343	Spectrin, beta subunit	10	0	0
IPR016357	Transferrin	10	0	0
IPR016437	Translation-associated RNA-binding, predicted	10	0	0
IPR016439	Ceramide synthase component Lag1/Lac1	10	0	0
IPR016473	Deoxycytidylate deaminase	10	0	0
IPR016492	Transcription elongation factor, TFIIS-related	10	0	0
IPR016579	Synaptogyrin	10	0	0
IPR016685	RNA-induced silencing complex, nuclease component Tudor-SN	10	0	0
IPR016818	Nitric oxide synthase-interacting	10	0	0
IPR016973	Integral membrane protein SYS1	10	0	0
IPR017065	NFU1 iron-sulfur cluster scaffold homologue	10	0	0
IPR017067	Ribonuclease H1, eukaryote	10	0	0
IPR017072	Transcription elongation factor Spt6	10	0	0
IPR017137	Arginine-tRNA-protein transferase 1, eukaryotic	10	0	0
IPR017151	5'-3' exoribonuclease 2/3	10	0	0

InterPro signature	InterPro description	Shared *Papaipema* genes	*Papaipema sp.4* SSGs	*Papaipema speciosissima* SSGs
IPR017182	S-adenosyl-L-methionine dependent methyltransferase, Mett10D, predicted	10	0	0
IPR017364	Gem-associated protein 2	10	0	0
IPR017380	Histone acetyltransferase type B, catalytic subunit	10	0	0
IPR017406	Serine/threonine-protein kinase Rio3	10	0	0
IPR017456	CTP synthase N-terminal	10	0	0
IPR017903	COS domain	10	0	0
IPR017919	Transcription factor TFE/TFIIEalpha HTH domain	10	0	0
IPR017943	Bactericidal permeability-increasing protein alpha/beta domain	10	0	0
IPR018020	Oxo-4-hydroxy-4-carboxy-5-ureidoimidazoline decarboxylase	10	0	0
IPR018034	KRR1 interacting protein 1	10	0	0
IPR018326	Rad4 beta-hairpin domain 1	10	0	0
IPR018327	Rad4 beta-hairpin domain 2	10	0	0
IPR018438	Carbonic anhydrase, CA-VII	10	0	0
IPR018472	Growth arrest/ DNA-damage-inducible protein-interacting protein 1	10	0	0
IPR018612	Domain of unknown function DUF2040	10	0	0
IPR018614	Uncharacterised protein family KRTCAP2	10	0	0
IPR018737	Protein LIN52	10	0	0
IPR018780	Tumour suppressor protein, LOH1CR12	10	0	0
IPR018791	UV radiation resistance protein/autophagy-related protein 14	10	0	0
IPR018799	TRAF3-interacting protein 1	10	0	0

**InterPro signature**	**InterPro description**	**Shared *Papaipema* genes**	***Papaipema sp.4* SSGs**	***Papaipema speciosissima* SSGs**
IPR018800	Proline-rich protein PRCC	10	0	0
IPR018882	Calmodulin-binding domain C0 NMDA receptor NR1 subunit	10	0	0
IPR019002	Ribosome biogenesis protein Nop16	10	0	0
IPR019008	Domain of unknown function DUF2012	10	0	0
IPR019009	Signal recognition particle receptor, beta subunit	10	0	0
IPR019013	Vacuolar ATPase assembly integral membrane protein Vma21	10	0	0
IPR019038	DNA polymerase subunit Cdc27	10	0	0
IPR019095	Mediator complex subunit Med18 metazoa/fungi	10	0	0
IPR019142	Dymeclin	10	0	0
IPR019162	Fanconi anemia complex subunit FancL WD-repeat containing domain	10	0	0
IPR019171	Caffeine-induced death protein 2	10	0	0
IPR019183	N-acetyltransferase B complex, non-catalytic subunit	10	0	0
IPR019185	Integral membrane protein SYS1-related	10	0	0
IPR019187	Cyclin-dependent kinase 2-associated protein	10	0	0
IPR019189	Ribosomal protein L27/L41, mitochondrial	10	0	0
IPR019266	Ribosomal protein S27, mitochondrial	10	0	0
IPR019306	Transmembrane protein 231	10	0	0
IPR019312	Protein of unknown function DUF2363	10	0	0
IPR019331	FAM192A/Fyv6 N-terminal	10	0	0
IPR019342	NADH:ubiquinone oxidoreductase, iron-sulphur subunit 5	10	0	0

**InterPro signature**	**InterPro description**	**Shared *Papaipema* genes**	***Papaipema sp.4* SSGs**	***Papaipema speciosissima* SSGs**
IPR019343	Uncharacterised domain KLRAQ/TTKRSYEDQ N-terminal	10	0	0
IPR019352	Uncharacterised protein family UPF0454	10	0	0
IPR019359	CCDC85 family	10	0	0
IPR019361	Protein of unknown function DUF2228, C2H2, APLF-like	10	0	0
IPR019364	Mediator complex, subunit Med8, fungi/metazoa	10	0	0
IPR019373	Ribosomal protein L51, mitochondrial	10	0	0
IPR019385	Phosphorylated adapter RNA export protein RNA-binding domain	10	0	0
IPR019392	Mitoguardin	10	0	0
IPR019398	Pre-rRNA-processing protein TSR2	10	0	0
IPR019467	Histone acetyl transferase HAT1 N-terminal	10	0	0
IPR019514	Protein of unknown function DUF2451 C-terminal	10	0	0
IPR019560	Mitochondrial 18kDa protein	10	0	0
IPR019576	Pyridoxine 5'-phosphate oxidase dimerisation C-terminal	10	0	0
IPR019605	Misato Segment II tubulin-like domain	10	0	0
IPR020164	Cytochrome c oxidase assembly protein COX16	10	0	0
IPR020783	Ribosomal protein L11 C-terminal	10	0	0
IPR020784	Ribosomal protein L11 N-terminal	10	0	0
IPR021563	Rab interacting lysosomal protein	10	0	0
IPR021625	PI31 proteasome regulator N-terminal	10	0	0
IPR021640	Mediator complex, subunit Med28	10	0	0

InterPro signature	InterPro description	Shared *Papaipema* genes	*Papaipema sp.4* SSGs	*Papaipema speciosissima* SSGs
IPR021715	Pre-mRNA splicing Prp18-interacting factor	10	0	0
IPR021774	Protein of unknown function DUF3338	10	0	0
IPR021816	Dedicator of cytokinesis C/D N-terminal	10	0	0
IPR021967	Nuclear protein 96	10	0	0
IPR022056	CpG binding protein C-terminal	10	0	0
IPR022083	KIF-1 binding protein	10	0	0
IPR022233	TRAPP II complex, TRAPPC10	10	0	0
IPR022577	Tubulin-specific chaperone D C-terminal	10	0	0
IPR022643	Orn/DAP/Arg decarboxylase 2 C-terminal	10	0	0
IPR022968	Ribosome biogenesis protein	10	0	0
IPR023340	UMA domain	10	0	0
IPR023379	ADP-ribosylation factor-like 2-binding protein domain	10	0	0
IPR023416	Transthyretin/hydroxyisourate hydrolase superfamily	10	0	0
IPR023799	Ribosome-binding factor A domain	10	0	0
IPR024095	Vesicle tethering protein p115-like	10	0	0
IPR024114	Islet cell autoantigen 1/Ica1-like	10	0	0
IPR024116	Resistance to inhibitors of cholinesterase protein 19	10	0	0
IPR024131	Uncharacterised protein family UPF0489	10	0	0
IPR024132	Akirin	10	0	0
IPR024224	DENND6	10	0	0

**InterPro signature**	**InterPro description**	**Shared *Papaipema* genes**	***Papaipema sp.4* SSGs**	***Papaipema speciosissima* SSGs**
IPR024236	Serine/threonine-protein kinase 40	10	0	0
IPR024420	TRAPP III complex, Trs85	10	0	0
IPR024448	Xylosyltransferase	10	0	0
IPR024550	TFIIEalpha/SarR/Rpc3 HTH domain	10	0	0
IPR024585	Domain of unknown function DUF3385 target of rapamycin protein	10	0	0
IPR024646	Angiomotin C-terminal	10	0	0
IPR024654	Calcineurin-like phosphoesterase domain lpxH type	10	0	0
IPR024661	DNA-directed RNA polymerase III, subunit Rpc31	10	0	0
IPR024668	GA-binding protein alpha subunit N-terminal	10	0	0
IPR024682	Nuclear pore localisation protein Npl4 ubiquitin-like domain	10	0	0
IPR024811	Polycomb protein ASX/ASX-like	10	0	0
IPR024814	Polycomb protein ASX	10	0	0
IPR024826	DNA polymerase delta/II small subunit family	10	0	0
IPR024858	Golgin subfamily A	10	0	0
IPR024887	Ashwin	10	0	0
IPR024888	U1 small nuclear ribonucleoprotein A/U2 small nuclear ribonucleoprotein B"	10	0	0
IPR024943	Enhancer of polycomb protein	10	0	0
IPR024954	SSRP1 domain	10	0	0
IPR024970	Maelstrom domain	10	0	0
IPR024974	Sde2 N-terminal domain	10	0	0

InterPro signature	InterPro description	Shared *Papaipema* genes	*Papaipema sp.4* SSGs	*Papaipema speciosissima* SSGs
IPR025066	Protein of unknown function DUF4078	10	0	0
IPR025271	Domain of unknown function DUF4061	10	0	0
IPR025300	Beta-galactosidase jelly roll domain	10	0	0
IPR025654	Peroxisome biogenesis factor 10	10	0	0
IPR025655	Peroxisomal membrane protein 14	10	0	0
IPR025723	Anion-transporting ATPase-like domain	10	0	0
IPR025766	ADD domain	10	0	0
IPR025790	Histone-lysine N-methyltransferase, Suvar4-20	10	0	0
IPR025807	Adrift-type ribose 2-O-methyltransferase domain	10	0	0
IPR025829	Zinc knuckle CX2CX3GHX4C	10	0	0
IPR025969	Abscisic acid G-protein coupled receptor-like domain	10	0	0
IPR026100	Transmembrane protein 223	10	0	0
IPR026126	BRISC and BRCA1-A complex member 1	10	0	0
IPR026213	DNA-directed RNA polymerase II subunit GRINL1	10	0	0
IPR026310	LAMTOR1-like	10	0	0
IPR026510	Peroxisomal membrane protein 11C, metazoa	10	0	0
IPR026620	Transmembrane protein 177	10	0	0
IPR026769	MICOS complex subunit Mic13	10	0	0
IPR026888	Acetyl-CoA hydrolase/transferase C-terminal domain	10	0	0
IPR026932	Myelin gene regulatory factor C-terminal domain 1	10	0	0

**InterPro signature**	**InterPro description**	**Shared *Papaipema* genes**	***Papaipema sp.4* SSGs**	***Papaipema speciosissima* SSGs**
IPR027012	Enkurin domain	10	0	0
IPR027040	Proteasome subunit Rpn10	10	0	0
IPR027057	CAAX prenyl protease 1	10	0	0
IPR027110	Pyruvate dehydrogenase E1 component subunit beta	10	0	0
IPR027138	TNF receptor-associated factor 4	10	0	0
IPR027159	Nuclear cap-binding protein subunit 1	10	0	0
IPR027235	Prefoldin subunit 2	10	0	0
IPR027306	Actin-related protein 2	10	0	0
IPR027755	Transcription factor Ovo-like	10	0	0
IPR027761	NudC domain-containing protein 3	10	0	0
IPR027993	Protein of unknown function DUF4495	10	0	0
IPR027999	Death-like domain of Spt6	10	0	0
IPR028133	Dynamitin	10	0	0
IPR028209	LAMTOR1/MEH1	10	0	0
IPR028231	Transcription elongation factor Spt6 YqgF domain	10	0	0
IPR028293	Verprolin	10	0	0
IPR028463	DBH-like monooxygenase protein 1	10	0	0
IPR028525	Stomatin-2	10	0	0
IPR029003	Kinetochore component CENP-S	10	0	0
IPR029159	Casein Kinase 2 substrate	10	0	0

InterPro signature	InterPro description	Shared *Papaipema* genes	*Papaipema sp.4* SSGs	*Papaipema speciosissima* SSGs
IPR029175	Exocyst complex component EXOC2/Sec5	10	0	0
IPR029295	Snf2 ATP coupling domain	10	0	0
IPR029445	Integrator complex subunit 5 N-terminal	10	0	0
IPR029448	Fanconi anaemia protein FANCD2	10	0	0
IPR029479	Nitroreductase	10	0	0
IPR029825	cAMP-dependent transcription factor ATF1	10	0	0
IPR030271	Voltage-dependent anion-selective channel protein 3	10	0	0
IPR030482	p53 and DNA damage-regulated protein 1	10	0	0
IPR030700	Arginine-tRNA-protein transferase	10	0	0
IPR031090	Serine/threonine-protein phosphatase 2A subunit A, metazoa	10	0	0
IPR031319	Alpha-amylase C-terminal domain	10	0	0
IPR031336	Cell division control protein 73 C-terminal	10	0	0
IPR031567	Sin1 middle CRIM domain	10	0	0
IPR031633	DNA replication complex GINS protein SLD5 C-terminal	10	0	0
IPR031657	Replication protein A OB domain	10	0	0
IPR031686	ATPsynthase alpha/beta subunit N-terminal extension	10	0	0
IPR031691	Lipoyl synthase N-terminal	10	0	0
IPR031720	Protein of unknown function DUF4728	10	0	0
IPR031736	ElonginA binding-protein 1	10	0	0
IPR031776	Splicing factor 3A subunit 3	10	0	0

InterPro signature	InterPro description	Shared *Papaipema* genes	*Papaipema sp.4* SSGs	*Papaipema speciosissima* SSGs
IPR031790	Nitric oxide synthase-interacting protein zinc-finger	10	0	0
IPR031983	Protein of unknown function DUF4786	10	0	0
IPR032016	Protein of unknown function DUF4796	10	0	0
IPR032028	Cleavage stimulation factor subunit 1 dimerisation domain	10	0	0
IPR032072	Protein of unknown function DUF4807	10	0	0
IPR032108	CLIP1 zinc knuckle	10	0	0
IPR032174	Intron-binding protein aquarius N-terminal	10	0	0
IPR032190	Niemann-Pick C1 N-terminal	10	0	0
IPR032198	E2F transcription factor CC-MB domain	10	0	0
IPR032343	Methyl-CpG-binding domain protein 2/3 p55-binding region	10	0	0
IPR032392	SATB ubiquitin-like oligomerisation domain	10	0	0
IPR032419	NF-kappa-B essential modulator NEMO CC2-LZ domain	10	0	0
IPR032430	Proteasome activator Blm10 mid region	10	0	0
IPR032435	Band 7 C-terminal extension	10	0	0
IPR032436	Nucleolar pre-ribosomal-associated protein 1 C-terminal domain	10	0	0
IPR032438	ERCC3/RAD25/XPB helicase C-terminal domain	10	0	0
IPR032442	Cytoplasmic tRNA 2-thiolation protein 1 C-terminal	10	0	0
IPR032464	Intron-binding protein aquarius	10	0	0
IPR032473	Protein argonaute Mid domain	10	0	0
IPR032476	Carnitine O-palmitoyltransferase N-terminal	10	0	0

InterPro signature	InterPro description	Shared *Papaipema* genes	*Papaipema sp.4* SSGs	*Papaipema speciosissima* SSGs
IPR032506	Domain of unknown function DUF4976	10	0	0
IPR032629	Mon2 dimerisation and cyclophilin-binding domain	10	0	0
IPR032706	Transcription elongation factor Spt6 helix-hairpin-helix motif	10	0	0
IPR032801	Peroxiredoxin-like FAM213/AAED1	10	0	0
IPR032880	Calcium permeable stress-gated cation channel 1 N-terminal transmembrane domain	10	0	0
IPR032910	WD repeat domain phosphoinositide-interacting protein 4	10	0	0
IPR033034	NADH dehydrogenase [ubiquinone] 1 beta subcomplex subunit 9	10	0	0
IPR033077	Ets DNA-binding protein pokkuri	10	0	0
IPR033102	Negative elongation factor E	10	0	0
IPR033110	Polypyrimidine tract-binding protein 3	10	0	0
IPR015416	Zinc finger H2C2-type histone UAS binding	9	5	4
IPR005162	Retrotransposon gag domain	9	4	3
IPR001377	Ribosomal protein S6e	9	3	1
IPR020810	Enolase C-terminal	9	0	3
IPR006942	TH1 protein	9	0	2
IPR007369	Peptidase A22B, signal peptide peptidase	9	1	1
IPR007742	Periplasmic copper-binding protein NosD beta helix domain	9	2	0
IPR008147	Glutamine synthetase beta-Grasp domain	9	2	0
IPR010418	ECSIT	9	1	1
IPR021980	Transcription factor homeodomain male germ-cell	9	2	0

InterPro signature	InterPro description	Shared *Papaipema* genes	*Papaipema sp.4* SSGs	*Papaipema speciosissima* SSGs
IPR026832	Asteroid	9	0	2
IPR000307	Ribosomal protein S16	9	0	1
IPR000463	Cytosolic fatty-acid binding	9	1	0
IPR000554	Ribosomal protein S7e	9	0	1
IPR000630	Ribosomal protein S8	9	1	0
IPR000701	Succinate dehydrogenase/Fumarate reductase, transmembrane subunit	9	1	0
IPR001667	DDH domain	9	1	0
IPR001884	Translation elongation factor IF5A	9	1	0
IPR002871	NIF system FeS cluster assembly NifU N-terminal	9	0	1
IPR004396	Ribosome-binding ATPase YchF/Obg-like ATPase 1	9	1	0
IPR004539	Translation elongation factor EF1A, eukaryotic/archaeal	9	0	1
IPR005633	Ribosomal protein L23/L25 N-terminal	9	0	1
IPR005819	Histone H5	9	0	1
IPR006921	Interferon-related developmental regulator C-terminal	9	1	0
IPR007269	Isoprenylcysteine carboxyl methyltransferase	9	1	0
IPR007704	Mannosyltransferase, DXD	9	1	0
IPR008699	NADH dehydrogenase [ubiquinone] 1 beta subcomplex subunit 8	9	1	0
IPR008905	Eukaryotic translation initiation factor 3 subunit C N-terminal domain	9	0	1
IPR009081	Acyl carrier protein-like	9	1	0
IPR009652	Programmed cell death protein 10	9	0	1

InterPro signature	InterPro description	Shared *Papaipema* genes	*Papaipema sp.4* SSGs	*Papaipema speciosissima* SSGs
IPR010674	Nucleolar GTP-binding protein 1 Rossman-fold domain	9	0	1
IPR014854	Non-structural maintenance of chromosome element 4 C-terminal	9	0	1
IPR015866	Serine-tRNA synthetase type1 N-terminal	9	0	1
IPR019473	Transcription factor TFIID subunit 8 C-terminal	9	0	1
IPR021018	Mediator complex, subunit Med29, metazoa	9	0	1
IPR023803	Ribosomal protein S16 domain	9	1	0
IPR025257	Domain of unknown function DUF4205	9	0	1
IPR025954	DBC1/CARP1 catalytically inactive NUDIX hydrolase domain	9	0	1
IPR026532	Ribosome biogenesis protein BRX1	9	1	0
IPR027328	Microtubule-associated protein RP/EB	9	1	0
IPR027487	Mitochondrial ribosomal protein L48	9	0	1
IPR027786	Nse4/EID family	9	1	0
IPR031942	Domain of unknown function DUF4774	9	0	1
IPR000056	Ribulose-phosphate 3-epimerase-like	9	0	0
IPR000072	PDGF/VEGF domain	9	0	0
IPR000114	Ribosomal protein L16	9	0	0
IPR000244	Ribosomal protein L9	9	0	0
IPR000469	G-protein alpha subunit, group 12/13	9	0	0
IPR000529	Ribosomal protein S6	9	0	0
IPR000620	EamA domain	9	0	0

InterPro signature	InterPro description	Shared *Papaipema* genes	*Papaipema sp.4* SSGs	*Papaipema speciosissima* SSGs
IPR000652	Triosephosphate isomerase	9	0	0
IPR000730	Proliferating cell nuclear antigen, PCNA	9	0	0
IPR000781	Enhancer of rudimentary	9	0	0
IPR000819	Peptidase M17 leucyl aminopeptidase C-terminal	9	0	0
IPR001015	Ferrochelatase	9	0	0
IPR001209	Ribosomal protein S14	9	0	0
IPR001288	Translation initiation factor 3	9	0	0
IPR001339	mRNA capping enzyme catalytic domain	9	0	0
IPR001533	Transcriptional coactivator/pterin dehydratase	9	0	0
IPR001684	Ribosomal protein L27	9	0	0
IPR001698	F-actin-capping protein subunit beta	9	0	0
IPR001720	PI3K p85 subunit	9	0	0
IPR001759	Pentaxin-related	9	0	0
IPR001774	Delta/Serrate/lag-2 DSL protein	9	0	0
IPR001779	Two pore domain potassium channel, TWIK-1	9	0	0
IPR001837	Adenylate cyclase-associated CAP	9	0	0
IPR001854	Ribosomal protein L29	9	0	0
IPR001863	Glypican	9	0	0
IPR001875	Death effector domain	9	0	0
IPR001907	ATP-dependent Clp protease proteolytic subunit	9	0	0

InterPro signature	InterPro description	Shared *Papaipema* genes	*Papaipema sp.4* SSGs	*Papaipema speciosissima* SSGs
IPR001915	Peptidase M48	9	0	0
IPR001975	Ribosomal protein L40e	9	0	0
IPR002112	Transcription factor Jun	9	0	0
IPR002115	Protein-tyrosine phosphatase, low molecular weight, mammalian	9	0	0
IPR002124	Cytochrome c oxidase, subunit Vb	9	0	0
IPR002454	Gamma tubulin	9	0	0
IPR002468	Peptidase M24A, methionine aminopeptidase, subfamily 2	9	0	0
IPR002625	Smr domain	9	0	0
IPR002637	Ham1-like protein	9	0	0
IPR002661	Ribosome recycling factor	9	0	0
IPR002738	RNase P subunit p30	9	0	0
IPR002782	Mut7-C RNAse domain	9	0	0
IPR003115	ParB/Sulfiredoxin	9	0	0
IPR003156	DHHA1 domain	9	0	0
IPR003162	Transcription initiation factor TAFII31	9	0	0
IPR003204	Cytochrome c oxidase, subunit Va/VI	9	0	0
IPR003228	Transcription initiation factor TFIID	9	0	0
IPR003565	Bis5'-nucleosyl)-tetraphosphatase	9	0	0
IPR003701	DNA double-strand break repair protein Mre11	9	0	0
IPR003754	Tetrapyrrole biosynthesis uroporphyrinogen III synthase	9	0	0

**InterPro signature**	**InterPro description**	**Shared *Papaipema* genes**	***Papaipema sp.4* SSGs**	***Papaipema speciosissima* SSGs**
IPR003769	Adaptor protein ClpS core	9	0	0
IPR003819	TauD/TfdA-like domain	9	0	0
IPR003864	Calcium-dependent channel 7TM region putative phosphate	9	0	0
IPR004044	K Homology domain type 2	9	0	0
IPR004102	PolyADP-ribose polymerase regulatory domain	9	0	0
IPR004226	Tubulin binding cofactor A	9	0	0
IPR004301	Nucleoplasmin family	9	0	0
IPR004331	SPX domain	9	0	0
IPR004530	Phenylalanyl-tRNA synthetase, class IIc, mitochondrial	9	0	0
IPR004557	Eukaryotic/archaeal PrmC-related	9	0	0
IPR004591	Replication factor A protein 1	9	0	0
IPR004730	Transaldolase type 1	9	0	0
IPR004856	Glycosyl transferase, ALG6/ALG8	9	0	0
IPR004918	Cdc37	9	0	0
IPR004953	EB1 C-terminal	9	0	0
IPR004963	Pectinacetylesterase/NOTUM	9	0	0
IPR005002	Eukaryotic phosphomannomutase	9	0	0
IPR005043	CAS/CSE C-terminal	9	0	0
IPR005121	Phenylalanine-tRNA ligase beta subunit ferrodoxin-fold anticodon-binding	9	0	0
IPR005164	Allantoicase	9	0	0

InterPro signature	InterPro description	Shared *Papaipema* genes	*Papaipema sp.4* SSGs	*Papaipema speciosissima* SSGs
IPR005341	Mitochondrial import inner membrane translocase subunit Tim16	9	0	0
IPR005349	TMEM14 family	9	0	0
IPR005480	Carbamoyl-phosphate synthetase large subunit oligomerisation domain	9	0	0
IPR005570	RNA polymerase, Rpb8	9	0	0
IPR005706	Ribosomal protein S2, bacteria/mitochondria/plastid	9	0	0
IPR005721	Ribosomal protein L22/L17, eukaryotic/archaeal	9	0	0
IPR005786	Branched-chain amino acid aminotransferase II	9	0	0
IPR005805	Rieske iron-sulphur protein C-terminal	9	0	0
IPR005857	Cystathionine beta-synthase	9	0	0
IPR005957	Tyrosine aminotransferase	9	0	0
IPR006003	Carbohydrate kinase, FGGY-related	9	0	0
IPR006114	6-phosphogluconate dehydrogenase C-terminal	9	0	0
IPR006122	Heavy metal-associated domain copper ion-binding	9	0	0
IPR006155	Machado-Joseph disease protein MJD	9	0	0
IPR006317	Ubiquinol-cytochrome c reductase iron-sulphur subunit	9	0	0
IPR006369	Protohaem IX farnesyltransferase	9	0	0
IPR006602	Uncharacterised domain DM10	9	0	0
IPR006631	Protein of unknown function DM4/12	9	0	0
IPR006687	Small GTPase superfamily, SAR1-type	9	0	0
IPR006746	26S proteasome non-ATPase regulatory subunit Rpn12	9	0	0

InterPro signature	InterPro description	Shared *Papaipema* genes	*Papaipema sp.4* SSGs	*Papaipema speciosissima* SSGs
IPR006828	Association with the SNF1 complex ASC domain	9	0	0
IPR006973	Pre-mRNA-splicing factor Cwf15/Cwc15	9	0	0
IPR006993	SH3-binding, glutamic acid-rich protein	9	0	0
IPR007018	Mediator complex, subunit Med6	9	0	0
IPR007023	Ribosomal biogenesis regulatory protein	9	0	0
IPR007174	Las1-like	9	0	0
IPR007187	Nucleoporin Nup133/Nup155-like C-terminal	9	0	0
IPR007188	Actin-related protein 2/3 complex subunit 2	9	0	0
IPR007192	Cdc23	9	0	0
IPR007199	Replication factor-A protein 1 N-terminal	9	0	0
IPR007204	Actin-related protein 2/3 complex subunit 3	9	0	0
IPR007231	Nucleoporin interacting component Nup93/Nic96	9	0	0
IPR007241	Autophagy-related protein 9	9	0	0
IPR007242	Ubiquitin-like protein Atg12	9	0	0
IPR007243	Atg6/Beclin	9	0	0
IPR007262	Vacuolar protein sorting 55	9	0	0
IPR007303	TIP41-like protein	9	0	0
IPR007315	GPI mannosyltransferase 2	9	0	0
IPR007374	ASCH domain	9	0	0
IPR007531	Dysbindin	9	0	0

**InterPro signature**	**InterPro description**	**Shared *Papaipema* genes**	***Papaipema sp.4* SSGs**	***Papaipema speciosissima* SSGs**
IPR007537	tRNAHis guanylyltransferase Thg1	9	0	0
IPR007716	NPL4 zinc-binding putative	9	0	0
IPR007724	PolyADP-ribose glycohydrolase	9	0	0
IPR007740	Ribosomal protein L49/IMG2	9	0	0
IPR007747	Menin	9	0	0
IPR007940	SH3-binding 5	9	0	0
IPR007949	SDA1 domain	9	0	0
IPR007955	Bystin	9	0	0
IPR008048	DNA replication licensing factor Mcm	9	0	0
IPR008257	Renal dipeptidase family	9	0	0
IPR008476	Protein PBDC1, metazoa/fungi	9	0	0
IPR008485	Protein of unknown function DUF766	9	0	0
IPR008559	Uncharacterised conserved protein UCP023322, transmembrane eukaryotic	9	0	0
IPR008606	Eukaryotic translation initiation factor 4E binding	9	0	0
IPR008685	Centromere protein Mis12	9	0	0
IPR008855	Translocon-associated	9	0	0
IPR008862	T-complex 11	9	0	0
IPR008932	Ribosomal protein L7/L12 oligomerisation	9	0	0
IPR008983	Tumour necrosis factor-like domain	9	0	0
IPR009048	Alpha-macroglobulin receptor-binding	9	0	0

InterPro signature	InterPro description	Shared *Papaipema* genes	*Papaipema sp.4* SSGs	*Papaipema speciosissima* SSGs
IPR009069	Cysteine alpha-hairpin motif superfamily	9	0	0
IPR009114	Angiomotin	9	0	0
IPR009161	ATP-dependent 6-phosphofructokinase, eukaryotic-type	9	0	0
IPR009316	COG complex component, COG2	9	0	0
IPR009332	Mediator of RNA polymerase II transcription subunit 22	9	0	0
IPR009548	Protein of unknown function DUF1168	9	0	0
IPR009580	GPI biosynthesis protein Pig-F	9	0	0
IPR009582	Signal peptidase complex subunit 2	9	0	0
IPR009771	Ribosome control protein 1	9	0	0
IPR009779	Translocon-associated protein subunit gamma	9	0	0
IPR009786	Spot 14 family	9	0	0
IPR009890	Etoposide-induced 2.4	9	0	0
IPR010099	Epimerase family protein SDR39U1	9	0	0
IPR010233	Ubiquinone biosynthesis O-methyltransferase	9	0	0
IPR010342	Protein of unknown function DUF938	9	0	0
IPR010491	PRP1 splicing factor N-terminal	9	0	0
IPR010531	Zinc finger protein NOA36	9	0	0
IPR010675	RNA methyltransferase bin3 C-terminal	9	0	0
IPR010729	Ribosomal protein L47, mitochondrial	9	0	0
IPR010754	Optic atrophy 3-like	9	0	0

**InterPro signature**	**InterPro description**	**Shared *Papaipema* genes**	***Papaipema sp.4* SSGs**	***Papaipema speciosissima* SSGs**
IPR010971	Ubiquinone biosynthesis hydroxylase UbiH/COQ6	9	0	0
IPR011048	Cytochrome cd1-nitrite reductase-like haem d1 domain	9	0	0
IPR011160	Sphingomyelin phosphodiesterase	9	0	0
IPR011282	2-amino-3-ketobutyrate coenzyme A ligase	9	0	0
IPR011339	ISC system FeS cluster assembly, IscU scaffold	9	0	0
IPR011356	Peptidase M17, leucine aminopeptidase/peptidase B	9	0	0
IPR011421	BCNT-C domain	9	0	0
IPR011422	BRCA1-associated 2	9	0	0
IPR011501	Nucleolar complex-associated protein 3 N-terminal	9	0	0
IPR011546	Peptidase M41 FtsH extracellular	9	0	0
IPR011548	3-hydroxyisobutyrate dehydrogenase	9	0	0
IPR011680	Fasciculation and elongation protein zeta, FEZ	9	0	0
IPR012404	Nucleotide-sugar transporter-related	9	0	0
IPR012430	Transmembrane protein 43 family	9	0	0
IPR012476	GLE1-like	9	0	0
IPR012541	DBP10CT	9	0	0
IPR012719	T-complex protein 1, gamma subunit	9	0	0
IPR012803	Fatty acid oxidation complex, alpha subunit, mitochondrial	9	0	0
IPR012848	Aspartic peptidase N-terminal	9	0	0
IPR012864	Cysteine oxygenase/2-aminoethanethiol dioxygenase	9	0	0

InterPro signature	InterPro description	Shared *Papaipema* genes	*Papaipema sp.4* SSGs	*Papaipema speciosissima* SSGs
IPR012911	Protein serine/threonine phosphatase 2C C-terminal	9	0	0
IPR012920	Ribosomal RNA methyltransferase Spb1 C-terminal	9	0	0
IPR012971	Nucleolar GTP-binding protein 2 N-terminal domain	9	0	0
IPR012988	Ribosomal protein L30 N-terminal	9	0	0
IPR012989	SEP domain	9	0	0
IPR013169	mRNA splicing factor, Cwf18	9	0	0
IPR013238	RNA polymerase III subunit Rpc25	9	0	0
IPR013498	DNA topoisomerase type IA zn finger	9	0	0
IPR013540	Chitinase A N-terminal	9	0	0
IPR013599	TRAM1-like protein	9	0	0
IPR013721	STAG	9	0	0
IPR013823	Ribosomal protein L7/L12 C-terminal	9	0	0
IPR013830	SGNH hydrolase-type esterase domain	9	0	0
IPR013866	Sphingolipid delta4-desaturase N-terminal	9	0	0
IPR013874	Cdc37 Hsp90 binding	9	0	0
IPR013881	Pre-mRNA-splicing factor 3	9	0	0
IPR013912	Adenylate cyclase-associated CAP C-terminal	9	0	0
IPR013930	RNA polymerase II-associated protein 1 N-terminal	9	0	0
IPR013955	Replication factor A C-terminal	9	0	0
IPR013970	Replication factor A protein 3	9	0	0

InterPro signature	InterPro description	Shared *Papaipema* genes	*Papaipema sp.4* SSGs	*Papaipema speciosissima* SSGs
IPR014030	Beta-ketoacyl synthase N-terminal	9	0	0
IPR014031	Beta-ketoacyl synthase C-terminal	9	0	0
IPR014183	Alcohol dehydrogenase class III/S-hydroxymethyl)glutathione dehydrogenase	9	0	0
IPR014314	Succinate dehydrogenase, cytochrome b556 subunit	9	0	0
IPR014637	Sorting nexin-5/6/32	9	0	0
IPR014813	Guanine nucleotide-binding protein-like 3 N-terminal domain	9	0	0
IPR014878	Domain of unknown function DUF1794	9	0	0
IPR015007	Nuclear pore complex NUP2/50/61	9	0	0
IPR015019	Ragulator complex protein LAMTOR3	9	0	0
IPR015033	HBS1-like protein N-terminal	9	0	0
IPR015036	USP8 interacting	9	0	0
IPR015047	Domain of unknown function DUF1866	9	0	0
IPR015096	Domain of unknown function DUF1897	9	0	0
IPR015120	Siah interacting protein N-terminal	9	0	0
IPR015155	PLAA family ubiquitin binding PFU	9	0	0
IPR015174	MIF4G-like type 2	9	0	0
IPR015248	Ubiquinol-cytochrome c reductase 8kDa N-terminal	9	0	0
IPR015311	Apoptosis DNA fragmentation factor 40kDa	9	0	0
IPR015351	LAG1 DNA binding	9	0	0
IPR015443	Aldose 1-epimerase	9	0	0

InterPro signature	InterPro description	Shared *Papaipema* genes	*Papaipema sp.4* SSGs	*Papaipema speciosissima* SSGs
IPR015445	TATA-Box binding protein-like	9	0	0
IPR015482	Syntrophin	9	0	0
IPR015567	Peptidase M14B, caboxypeptidase D	9	0	0
IPR015815	3-hydroxyisobutyrate dehydrogenase-related	9	0	0
IPR015865	Riboflavin kinase domain bacterial/eukaryotic	9	0	0
IPR015908	Allantoicase domain	9	0	0
IPR016195	Polymerase/histidinol phosphatase-like	9	0	0
IPR016238	Ribosomal protein S6 kinase	9	0	0
IPR016454	Cysteine desulfurase	9	0	0
IPR016478	GTPase, MTG1	9	0	0
IPR016576	Ribosomal protein 63, mitochondrial	9	0	0
IPR016650	Eukaryotic translation initiation factor 3 subunit E	9	0	0
IPR016689	ESCRT-2 complex, Snf8	9	0	0
IPR016690	tRNA-splicing endonuclease, SEN34 subunit	9	0	0
IPR016692	Sulfiredoxin	9	0	0
IPR016696	TRAPP I complex, subunit 5	9	0	0
IPR016706	Cleavage/polyadenylation specificity factor subunit 5	9	0	0
IPR016722	DNA polymerase alpha, subunit B	9	0	0
IPR016819	Ribonuclease P/MRP protein subunit Pop5	9	0	0
IPR016901	Anaphase-promoting complex, subunit 10	9	0	0

InterPro signature	InterPro description	Shared *Papaipema* genes	*Papaipema sp.4* SSGs	*Papaipema speciosissima* SSGs
IPR017106	Coatomer gamma subunit	9	0	0
IPR017246	Snapin	9	0	0
IPR017303	Mitochondrial import inner membrane translocase subunit Tim44	9	0	0
IPR017334	Eukaryotic translation initiation factor 3 subunit G	9	0	0
IPR017340	U1 small nuclear ribonucleoprotein C	9	0	0
IPR017568	3-oxoacyl-[acyl-carrier-protein] synthase 2	9	0	0
IPR017597	Pyruvate dehydrogenase acetyl-transferring E1 component, alpha subunit, subgroup y	9	0	0
IPR017867	Protein-tyrosine phosphatase, low molecular weight	9	0	0
IPR018075	Ubiquitin-activating enzyme E1	9	0	0
IPR018178	Putative SWEET transporter, Insecta	9	0	0
IPR018325	Rad4/PNGase transglutaminase-like fold	9	0	0
IPR018328	Rad4 beta-hairpin domain 3	9	0	0
IPR018607	Chromosome transmission fidelity protein 8	9	0	0
IPR018617	Ima1 N-terminal domain	9	0	0
IPR018618	Vacuolar import/degradation protein Vid24	9	0	0
IPR018788	Proteasome assembly chaperone 3	9	0	0
IPR018790	Protein of unknown function DUF2358	9	0	0
IPR018795	Protein of unknown function DUF2152	9	0	0
IPR018796	Uncharacterised protein family UPF0671	9	0	0
IPR018808	Muniscin C-terminal	9	0	0

InterPro signature	InterPro description	Shared *Papaipema* genes	*Papaipema sp.4* SSGs	*Papaipema speciosissima* SSGs
IPR018972	Sas10 C-terminal domain	9	0	0
IPR018999	RNA helicase UPF1 UPF2-interacting domain	9	0	0
IPR019010	Eukaryotic translation initiation factor 3 subunit E N-terminal	9	0	0
IPR019144	Membralin	9	0	0
IPR019168	Nuclear envelope phosphatase-regulatory subunit 1	9	0	0
IPR019178	Phosphatidylinositol 4,5-bisphosphate 4-phosphatase	9	0	0
IPR019194	Transcription elognation factor Eaf N-terminal	9	0	0
IPR019265	Protein of unknown function UPF0568	9	0	0
IPR019319	Protein of unknown function DUF2368	9	0	0
IPR019328	GPI-GlcNAc transferase complex, PIG-H component, conserved domain	9	0	0
IPR019330	Mesoderm development candidate 2	9	0	0
IPR019334	Transmembrane protein 170	9	0	0
IPR019338	Ribosomal protein L35, mitochondrial	9	0	0
IPR019348	Uncharacterised domain KLRAQ/TTKRSYEDQ C-terminal	9	0	0
IPR019367	PDZ-binding protein, CRIPT	9	0	0
IPR019375	Ribosomal protein S28, mitochondrial	9	0	0
IPR019389	Selenoprotein T	9	0	0
IPR019391	Storkhead-box protein winged-helix domain	9	0	0
IPR019404	Mediator complex, subunit Med11	9	0	0
IPR019460	Autophagy-related protein 11	9	0	0

InterPro signature	InterPro description	Shared *Papaipema* genes	*Papaipema sp.4* SSGs	*Papaipema speciosissima* SSGs
IPR019519	Elongator complex protein 5	9	0	0
IPR019520	Ribosomal protein S23/Rsm25, mitochondrial	9	0	0
IPR019528	Pericentrin/AKAP-450 centrosomal targeting domain	9	0	0
IPR019610	Iron sulphur domain-containing mitoNEET N-terminal	9	0	0
IPR019815	Translation initiation factor 3 C-terminal	9	0	0
IPR020070	Ribosomal protein L9 N-terminal	9	0	0
IPR020309	Uncharacterised protein family, CD034/YQF4	9	0	0
IPR020839	Stromalin conservative domain	9	0	0
IPR020841	Polyketide synthase beta-ketoacyl synthase domain	9	0	0
IPR020859	Roc domain	9	0	0
IPR021066	Proteinase inhibitor I83, AmFPI-1	9	0	0
IPR021137	Ribosomal protein L35	9	0	0
IPR021148	Polysaccharide biosynthesis domain	9	0	0
IPR021163	Ferredoxin-NADP+ reductase, adrenodoxin-type	9	0	0
IPR021183	N-terminal acetyltransferase A, auxiliary subunit	9	0	0
IPR021384	Mediator complex, subunit Med21	9	0	0
IPR021569	TUG ubiquitin-like domain	9	0	0
IPR021627	Mediator complex, subunit Med27	9	0	0
IPR021648	Vacuolar protein sorting protein 36 GLUE domain	9	0	0
IPR021752	Transcription initiation factor Rrn7	9	0	0

InterPro signature	InterPro description	Shared *Papaipema* genes	*Papaipema sp.4* SSGs	*Papaipema speciosissima* SSGs
IPR021977	D domain of beta-TrCP	9	0	0
IPR022034	Fragile X-related 1 protein C-terminal core	9	0	0
IPR022092	TATA element modulatory factor 1 DNA binding	9	0	0
IPR022122	Protein FAM135	9	0	0
IPR022154	Trafficking kinesin-binding protein domain	9	0	0
IPR022170	Mitochondrial ubiquitin ligase activator of NFKB 1	9	0	0
IPR022209	Pre-mRNA splicing factor	9	0	0
IPR022232	Peptidase S8A tripeptidyl peptidase II arthropoda	9	0	0
IPR022581	Spt5 transcription elongation factor N-terminal	9	0	0
IPR022648	Proliferating cell nuclear antigen PCNA N-terminal	9	0	0
IPR022649	Proliferating cell nuclear antigen PCNA C-terminal	9	0	0
IPR022772	von Hippel-Lindau disease tumour suppressor beta/alpha domain	9	0	0
IPR022773	Siva	9	0	0
IPR023128	Protein N-terminal glutamine amidohydrolase alpha beta roll	9	0	0
IPR023238	FAM175 family	9	0	0
IPR023468	Riboflavin kinase	9	0	0
IPR023485	Phosphotyrosine protein phosphatase I superfamily	9	0	0
IPR023562	Clp protease proteolytic subunit /Translocation-enhancing protein TepA	9	0	0
IPR023584	Ribosome recycling factor domain	9	0	0
IPR023611	Ribosomal protein S23/S25 mitochondrial	9	0	0

**InterPro signature**	**InterPro description**	**Shared *Papaipema* genes**	***Papaipema sp.4* SSGs**	***Papaipema speciosissima* SSGs**
IPR024057	Nucleoplasmin core domain	9	0	0
IPR024061	NDT80 DNA-binding domain	9	0	0
IPR024145	Histone deacetylase complex subunit SAP30/SAP30-like	9	0	0
IPR024160	Bin3-type S-adenosyl-L-methionine binding domain	9	0	0
IPR024166	Ribosomal RNA assembly KRR1	9	0	0
IPR024326	Ribosomal RNA-processing protein 7	9	0	0
IPR024336	tRNA-splicing endonuclease subunit Sen54 N-terminal	9	0	0
IPR024599	Retinoblastoma-associated protein N-terminal	9	0	0
IPR024641	Hepatocyte growth factor-regulated tyrosine kinase substrate helical domain	9	0	0
IPR024660	UNC-45/Cro1/She4 central domain	9	0	0
IPR024675	Eukaryotic translation initiation factor 3 subunit G N-terminal	9	0	0
IPR024689	Proteasome beta subunit C-terminal	9	0	0
IPR024705	Spermatogenesis-associated protein 20	9	0	0
IPR024711	Catalase, mono-functional, haem-containing, clades 1 and 3	9	0	0
IPR024732	Alpha-N-acetylglucosaminidase C-terminal	9	0	0
IPR024956	tRNAHis guanylyltransferase catalytic domain	9	0	0
IPR025069	Cleavage and polyadenylation specificity factor 2 C-terminal	9	0	0
IPR025283	Domain of unknown function DUF4042	9	0	0
IPR025286	MOFRL-associated domain	9	0	0
IPR025640	Domain of unknown function DUF4339	9	0	0

InterPro signature	InterPro description	Shared *Papaipema* genes	*Papaipema sp.4* SSGs	*Papaipema speciosissima* SSGs
IPR025718	Histone deacetylase complex subunit SAP30 Sin3 binding domain	9	0	0
IPR025845	Thg1 C-terminal domain	9	0	0
IPR025884	Methyl-CpG binding protein 2/3 C-terminal domain	9	0	0
IPR025999	Microspherule protein N-terminal domain	9	0	0
IPR026019	Ribulose-phosphate 3-epimerase	9	0	0
IPR026051	Chitobiosyldiphosphodolichol beta-mannosyltransferase ALG1-like	9	0	0
IPR026057	PC-Esterase	9	0	0
IPR026073	Gametogenetin-binding protein 2	9	0	0
IPR026140	28S ribosomal protein S26	9	0	0
IPR026146	28S ribosomal protein S24, mitochondrial	9	0	0
IPR026164	Integrator complex subunit 10	9	0	0
IPR026258	Signal recognition particle subunit SRP68	9	0	0
IPR026283	Beta-galactosidase 1-like	9	0	0
IPR026303	ATP synthase subunit s-like protein	9	0	0
IPR026533	Non-canonical purine NTP phosphatase/PRRC1	9	0	0
IPR026534	Protein PRRC1	9	0	0
IPR026561	Frizzled, invertebrates	9	0	0
IPR026569	Ribosomal protein L28/L24	9	0	0
IPR026572	Transmembrane protein C5orf28-like	9	0	0
IPR026581	T-complex protein 10 family	9	0	0

**InterPro signature**	**InterPro description**	**Shared *Papaipema* genes**	***Papaipema sp.4* SSGs**	***Papaipema speciosissima* SSGs**
IPR026828	Suppressor APC domain-containing protein 1/2	9	0	0
IPR026846	E3 SUMO-protein ligase Nse2 Mms21)	9	0	0
IPR026848	E3 ubiquitin-protein ligase FANCL	9	0	0
IPR026992	Non-haem dioxygenase N-terminal domain	9	0	0
IPR027093	EAF family	9	0	0
IPR027108	Pre-mRNA-processing factor 6/Prp1/STA1	9	0	0
IPR027170	Transcriptional activator NFYC/HAP5 subunit	9	0	0
IPR027312	Sda1	9	0	0
IPR027329	TPX2 C-terminal	9	0	0
IPR027477	Succinate dehydrogenase/fumarate reductase flavoprotein catalytic domain	9	0	0
IPR027710	Enkurin domain-containing protein 1	9	0	0
IPR028077	Ubiquitin/SUMO-activating enzyme ubiquitin-like domain	9	0	0
IPR028119	Snapin/Pallidin/Snn1	9	0	0
IPR028191	WASH complex subunit 7 N-terminal	9	0	0
IPR028260	FAM177 family	9	0	0
IPR028310	Retinoblastoma-like protein 1	9	0	0
IPR028421	Suppressor of cytokine signalling 6	9	0	0
IPR028589	AdoMet-dependent rRNA methyltransferase, Spb1	9	0	0
IPR028638	Inhibitor of growth protein 5	9	0	0
IPR028816	Caprin	9	0	0

InterPro signature	InterPro description	Shared *Papaipema* genes	*Papaipema sp.4* SSGs	*Papaipema speciosissima* SSGs
IPR028820	Sodium channel protein 60E	9	0	0
IPR028945	WRB/Get1 family	9	0	0
IPR029169	PEST proteolytic signal-containing nuclear protein	9	0	0
IPR029260	Dual specificity/tyrosine protein phosphatase N-terminal	9	0	0
IPR029321	Integrator complex subunit 2	9	0	0
IPR029397	Tube Death domain	9	0	0
IPR029454	ODR-4-like	9	0	0
IPR029525	INO80 complex, subunit Ies6	9	0	0
IPR030080	5'-AMP-activated protein kinase subunit beta-1	9	0	0
IPR030408	AT-rich interactive domain-containing protein 5B	9	0	0
IPR030710	Ras-GEF domain-containing family member 1B	9	0	0
IPR031072	Syntrophin-1	9	0	0
IPR031101	RNA polymerase-associated protein Ctr9	9	0	0
IPR031313	SAPK-interacting protein 1 Pleckstrin-homology domain	9	0	0
IPR031615	MYND-like zinc finger mRNA-binding	9	0	0
IPR031693	Sin3 C-terminal	9	0	0
IPR031761	Folded gastrulation N-terminal domain	9	0	0
IPR031804	Domain of unknown function DUF4743	9	0	0
IPR031896	Type II inositol 145-trisphosphate 5-phosphatase PH domain	9	0	0
IPR032053	Mitochondrial 28S ribosomal protein S34	9	0	0

InterPro signature	InterPro description	Shared *Papaipema* genes	*Papaipema sp.4* SSGs	*Papaipema speciosissima* SSGs
IPR032067	FOXO protein transactivation domain	9	0	0
IPR032147	Domain of unknown function DUF4819	9	0	0
IPR032281	40S ribosomal protein SA C-terminal domain	9	0	0
IPR032350	Next to BRCA1 central domain	9	0	0
IPR032412	Myosin VI cargo binding domain	9	0	0
IPR032498	PI3K p85 subunit inter-SH2 domain	9	0	0
IPR032640	AMP-activated protein kinase glycogen-binding domain	9	0	0
IPR032641	Exonuclease 1	9	0	0
IPR032660	Protein atonal homologue 8	9	0	0
IPR032734	DDB1- and CUL4-associated factor 15 WD40 repeat-containing domain	9	0	0
IPR032849	Transcriptional factor cubitus interruptus	9	0	0
IPR032963	Glutamate--cysteine ligase regulatory subunit	9	0	0
IPR033105	Cleavage stimulation factor subunit 2	9	0	0
IPR032135	Protein of unknown function DUF4817	8	1	5
IPR032071	Domain of unknown function DUF4806	8	5	0
IPR005485	Ribosomal protein L5 eukaryotic/L18 archaeal	8	4	0
IPR025398	Domain of unknown function DUF4371	8	1	2
IPR000266	Ribosomal protein S17/S11	8	2	0
IPR001147	Ribosomal protein L21e	8	2	0
IPR001282	Glucose-6-phosphate dehydrogenase	8	2	0

**InterPro signature**	**InterPro description**	**Shared *Papaipema* genes**	***Papaipema sp.4* SSGs**	***Papaipema speciosissima* SSGs**
IPR022674	Glucose-6-phosphate dehydrogenase NAD-binding	8	0	2
IPR032450	SMARCC N-terminal	8	0	2
IPR013837	ATPase, F0 complex, B chain/subunit B	8	1	0
IPR013847	POU domain	8	1	0
IPR000039	Ribosomal protein L18e	8	1	0
IPR000231	Ribosomal protein L30e	8	1	0
IPR000327	POU-specific domain	8	1	0
IPR002132	Ribosomal protein L5	8	1	0
IPR002192	Pyruvate phosphate dikinase PEP/pyruvate-binding	8	0	1
IPR002326	Cytochrome c1	8	1	0
IPR003177	Cytochrome c oxidase, subunit VIIa	8	1	0
IPR003377	Cornichon	8	0	1
IPR003386	Lecithin:cholesterol/phospholipid:diacylglycerol acyltransferase	8	1	0
IPR003675	CAAX amino terminal protease	8	1	0
IPR005139	Peptide chain release factor	8	0	1
IPR006148	Glucosamine/galactosamine-6-phosphate isomerase	8	0	1
IPR007676	Ribophorin I	8	1	0
IPR008283	Peptidase M17 leucyl aminopeptidase N-terminal	8	1	0
IPR008688	ATPase, F0 complex, B chain/subunit B/MI25	8	1	0
IPR012136	NADP transhydrogenase, beta subunit	8	1	0

InterPro signature	InterPro description	Shared *Papaipema* genes	*Papaipema sp.4* SSGs	*Papaipema speciosissima* SSGs
IPR012923	Chromosome segregation in meiosis protein 3	8	0	1
IPR019164	Protein of unknown function DUF2053, membrane	8	0	1
IPR019329	NADH:ubiquinone oxidoreductase, ESSS subunit	8	1	0
IPR024546	Ribosome biogenesis protein RLP24	8	1	0
IPR025770	Protein-S-isoprenylcysteine O-methyltransferase	8	1	0
IPR027963	Meiosis-specific coiled-coil domain-containing protein MEIOC	8	1	0
IPR029915	ISWI family	8	1	0
IPR031662	GTP binding protein second domain	8	0	1
IPR032134	Protein of unknown function DUF4816	8	1	0
IPR032418	Ubiquitin-activating enzyme E1 FCCH domain	8	0	1
IPR032420	Ubiquitin-activating enzyme E1 four-helix bundle	8	0	1
IPR032832	E3 ubiquitin-protein ligase synoviolin/Hrd1	8	0	1
IPR000305	GIY-YIG nuclease superfamily	8	0	0
IPR000398	Thymidylate synthase	8	0	0
IPR000456	Ribosomal protein L17	8	0	0
IPR000509	Ribosomal protein L36e	8	0	0
IPR000544	Octanoyltransferase	8	0	0
IPR000692	Fibrillarin	8	0	0
IPR000903	Myristoyl-CoA:protein N-myristoyltransferase	8	0	0
IPR000974	Glycoside hydrolase, family 22, lysozyme	8	0	0

InterPro signature	InterPro description	Shared *Papaipema* genes	*Papaipema sp.4* SSGs	*Papaipema speciosissima* SSGs
IPR001045	Spermidine/spermine synthases	8	0	0
IPR001047	Ribosomal protein S8e	8	0	0
IPR001154	DNA topoisomerase II, eukaryotic-type	8	0	0
IPR001200	Phosducin	8	0	0
IPR001233	tRNA-splicing ligase, RtcB	8	0	0
IPR001241	DNA topoisomerase, type IIA	8	0	0
IPR001347	Sugar isomerase SIS	8	0	0
IPR001387	Cro/C1-type helix-turn-helix domain	8	0	0
IPR001408	G-protein alpha subunit, group I	8	0	0
IPR001445	Acetylcholinesterase, insect	8	0	0
IPR001733	Peptidase S26B	8	0	0
IPR001738	Rab protein geranylgeranyltransferase component A	8	0	0
IPR001748	G10 protein	8	0	0
IPR001820	Protease inhibitor I35 TIMP)	8	0	0
IPR001844	Chaperonin Cpn60	8	0	0
IPR001925	Porin, eukaryotic type	8	0	0
IPR002082	Aspartate carbamoyltransferase	8	0	0
IPR002087	Anti-proliferative protein	8	0	0
IPR002222	Ribosomal protein S19/S15	8	0	0
IPR002227	Tyrosinase copper-binding domain	8	0	0

InterPro signature	InterPro description	Shared *Papaipema* genes	*Papaipema sp.4* SSGs	*Papaipema speciosissima* SSGs
IPR002232	5-Hydroxytryptamine 6 receptor	8	0	0
IPR002316	Proline-tRNA ligase, class IIa	8	0	0
IPR002413	Ves allergen	8	0	0
IPR002415	H/ACA ribonucleoprotein complex, subunit Nhp2, eukaryote	8	0	0
IPR002719	Retinoblastoma-associated protein B-box	8	0	0
IPR002730	Ribonuclease P/MRP subunit p29	8	0	0
IPR002769	Translation initiation factor IF6	8	0	0
IPR002775	DNA/RNA-binding protein Alba-like	8	0	0
IPR003038	DAD/Ost2	8	0	0
IPR003149	Iron hydrogenase small subunit	8	0	0
IPR003164	Clathrin adaptor alpha-adaptin appendage C-terminal subdomain	8	0	0
IPR003256	Ribosomal protein L24	8	0	0
IPR003406	Glycosyl transferase, family 14	8	0	0
IPR003437	Glycine dehydrogenase decarboxylating)	8	0	0
IPR003736	Phenylacetic acid degradation-related domain	8	0	0
IPR003874	CDC45 family	8	0	0
IPR004114	THUMP domain	8	0	0
IPR004125	Signal recognition particle SRP54 subunit M-domain	8	0	0
IPR004241	Autophagy protein Atg8 ubiquitin-like	8	0	0
IPR004394	Protein Iojap/ribosomal silencing factor RsfS	8	0	0

**InterPro signature**	**InterPro description**	**Shared *Papaipema* genes**	***Papaipema sp.4* SSGs**	***Papaipema speciosissima* SSGs**
IPR004493	Leucyl-tRNA synthetase, class Ia, archaeal/eukaryotic cytosolic	8	0	0
IPR004514	Glutamine-tRNA synthetase	8	0	0
IPR004529	Phenylalanyl-tRNA synthetase, class IIc, alpha subunit	8	0	0
IPR004541	Translation elongation factor EFTu/EF1A, bacterial/organelle	8	0	0
IPR004545	PA2G4 family	8	0	0
IPR004554	Hydroxymethylglutaryl-CoA reductase, eukaryotic/arcaheal type	8	0	0
IPR004572	Protoporphyrinogen oxidase	8	0	0
IPR004631	4-aminobutyrate aminotransferase, eukaryotic	8	0	0
IPR004769	Adenylosuccinate lyase	8	0	0
IPR004776	Auxin efflux carrier	8	0	0
IPR004788	Ribose 5-phosphate isomerase, type A	8	0	0
IPR004808	AP endonuclease 1	8	0	0
IPR004855	Transcription factor IIA, alpha/beta subunit	8	0	0
IPR004942	Roadblock/LAMTOR2 domain	8	0	0
IPR005100	Transcription elongation factor Spt5 NGN domain	8	0	0
IPR005110	MoeA N-terminal and linker domain	8	0	0
IPR005155	Ribosome biogenesis factor NIP7-like	8	0	0
IPR005199	Glycoside hydrolase, family 79	8	0	0
IPR005304	Ribosomal biogenesis, methyltransferase, EMG1/NEP1	8	0	0
IPR005322	Peptidase C69, dipeptidase A	8	0	0

**InterPro signature**	**InterPro description**	**Shared *Papaipema* genes**	***Papaipema sp.4* SSGs**	***Papaipema speciosissima* SSGs**
IPR005334	Tctex-1	8	0	0
IPR005344	Per33/Pom33 family	8	0	0
IPR005483	Carbamoyl-phosphate synthase large subunit CPSase domain	8	0	0
IPR005491	EMSY N-terminal	8	0	0
IPR005595	Translocon-associated protein TRAP), alpha subunit	8	0	0
IPR005606	Sec20	8	0	0
IPR005645	Serine hydrolase FSH	8	0	0
IPR005651	Uncharacterised protein family UPF0434/Trm112	8	0	0
IPR005677	Fumarate hydratase, class II	8	0	0
IPR005678	Mitochondrial inner membrane translocase complex, subunit Tim17	8	0	0
IPR005683	Mitochondrial import receptor subunit Tom22	8	0	0
IPR005713	Ribosomal protein S19A/S15e	8	0	0
IPR005723	ATPase, V1 complex, subunit B	8	0	0
IPR005756	Ribosomal protein L26/L24P, eukaryotic/archaeal	8	0	0
IPR005900	6-phosphogluconolactonase DevB-type	8	0	0
IPR005930	Pyruvate carboxylase	8	0	0
IPR005931	Delta-1-pyrroline-5-carboxylate dehydrogenase	8	0	0
IPR005961	Phenylalanine-4-hydroxylase, tetrameric form	8	0	0
IPR005962	Tyrosine 3-monooxygenase	8	0	0
IPR005998	Ribosomal protein L7, eukaryotic	8	0	0

InterPro signature	InterPro description	Shared *Papaipema* genes	*Papaipema sp.4* SSGs	*Papaipema speciosissima* SSGs
IPR006019	Phosphotyrosine interaction domain Shc-like	8	0	0
IPR006052	Tumour necrosis factor domain	8	0	0
IPR006075	Aspartyl/Glutamyl-tRNAGln amidotransferase subunit B/E catalytic	8	0	0
IPR006110	RNA polymerase, subunit omega/K/RPB6	8	0	0
IPR006113	6-phosphogluconate dehydrogenase, decarboxylating	8	0	0
IPR006130	Aspartate/ornithine carbamoyltransferase	8	0	0
IPR006131	Aspartate/ornithine carbamoyltransferase Asp/Orn-binding domain	8	0	0
IPR006132	Aspartate/ornithine carbamoyltransferase carbamoyl-P binding	8	0	0
IPR006190	Antifreeze-like/N-acetylneuraminic acid synthase C-terminal	8	0	0
IPR006248	Aconitase, mitochondrial-like	8	0	0
IPR006262	Cytidine deaminase, homotetrameric	8	0	0
IPR006275	Carbamoyl-phosphate synthase, large subunit	8	0	0
IPR006325	Signal recognition particle, SRP54 subunit, eukaryotic	8	0	0
IPR006466	MiaB-like tRNA modifying enzyme, archaeal-type	8	0	0
IPR006515	Polyadenylate binding protein, human types 1, 2, 3, 4	8	0	0
IPR006532	Poly-U binding splicing factor, half-pint	8	0	0
IPR006549	HAD-superfamily hydrolasesubfamily IIIA	8	0	0
IPR006551	Polynucleotide 3'-phosphatase	8	0	0
IPR006645	NusG N-terminal	8	0	0
IPR006683	Thioesterase domain	8	0	0

InterPro signature	InterPro description	Shared *Papaipema* genes	*Papaipema sp.4* SSGs	*Papaipema speciosissima* SSGs
IPR006723	Islet cell autoantigen Ica1 C-terminal	8	0	0
IPR006751	TAFII55 protein conserved region	8	0	0
IPR006789	Actin-related protein 2/3 complex subunit 5	8	0	0
IPR006804	BCL7	8	0	0
IPR006806	ETC complex I subunit	8	0	0
IPR006811	RNA polymerase II subunit A	8	0	0
IPR006885	NADH dehydrogenase ubiquinone Fe-S protein 4, mitochondrial	8	0	0
IPR006888	XLR/SYCP3/FAM9 domain	8	0	0
IPR006931	Calcipressin	8	0	0
IPR006958	Mak16 protein	8	0	0
IPR006966	Peroxin-3	8	0	0
IPR007073	RNA polymerase Rpb1 domain 7	8	0	0
IPR007143	Vacuolar protein sorting-associated, VPS28	8	0	0
IPR007213	Leucine carboxyl methyltransferase	8	0	0
IPR007216	Rcd1/Caf40	8	0	0
IPR007222	Signal recognition particle receptor alpha subunit N-terminal	8	0	0
IPR007287	Sof1-like protein	8	0	0
IPR007317	Uncharacterised protein family UPF0363	8	0	0
IPR007471	Arginine-tRNA-protein transferase N-terminal	8	0	0
IPR007583	GRASP55/65	8	0	0

InterPro signature	InterPro description	Shared *Papaipema* genes	*Papaipema sp.4* SSGs	*Papaipema speciosissima* SSGs
IPR007714	Cilia/flagella-associated protein 20/WDR90/C3orf67	8	0	0
IPR007835	MOFRL domain	8	0	0
IPR007866	TRIC channel	8	0	0
IPR007919	Uncharacterised protein family UPF0220	8	0	0
IPR007946	A1 cistron-splicing factor, AAR2	8	0	0
IPR007967	Protein of unknown function DUF727	8	0	0
IPR008009	Putative Ig	8	0	0
IPR008012	Proteasome maturation factor UMP1	8	0	0
IPR008113	Septin 2	8	0	0
IPR008180	Deoxyuridine triphosphate nucleotidohydrolase/Deoxycytidine triphosphate deaminase	8	0	0
IPR008181	Deoxyuridine triphosphate nucleotidohydrolase	8	0	0
IPR008195	Ribosomal protein L34Ae	8	0	0
IPR008279	PEP-utilising enzyme mobile domain	8	0	0
IPR008465	Dystroglycan	8	0	0
IPR008516	Na,K-Atpase Interacting protein	8	0	0
IPR008576	Alpha-N-methyltransferase NTM1	8	0	0
IPR008698	NADH:ubiquinone oxidoreductase, B18 subunit	8	0	0
IPR008831	Mediator complex, subunit Med31	8	0	0
IPR008883	Ubiquitin E2 variant N-terminal	8	0	0
IPR008893	WGR domain	8	0	0

InterPro signature	InterPro description	Shared *Papaipema* genes	*Papaipema sp.4* SSGs	*Papaipema speciosissima* SSGs
IPR008901	Ceramidase	8	0	0
IPR009287	Transcription initiation Spt4	8	0	0
IPR009292	rRNA biogenesis protein RRP36	8	0	0
IPR009345	BMP and activin membrane-bound inhibitor	8	0	0
IPR009346	GRIM-19	8	0	0
IPR009361	RZZ complex, subunit Zw10	8	0	0
IPR009423	NADH:ubiquinone oxidoreductase, subunit b14.5b	8	0	0
IPR009432	Protein of unknown function DUF1075	8	0	0
IPR009450	Phosphatidylinositol N-acetylglucosaminyltransferase subunit C	8	0	0
IPR009463	Domain of unknown function DUF1087	8	0	0
IPR009674	DNA-directed RNA polymerase I subunit RPA2 domain 4	8	0	0
IPR009724	TMEM70 family	8	0	0
IPR009947	NADH:ubiquinone oxidoreductase subunit B14.5a	8	0	0
IPR009991	Dynactin subunit p22	8	0	0
IPR010122	Hydroxymethylglutaryl-CoA synthase, eukaryotic	8	0	0
IPR010240	Cysteine desulfurase IscS	8	0	0
IPR010376	Domain of unknown function DUF971	8	0	0
IPR010378	Protein of unknown function DUF974	8	0	0
IPR010490	Conserved oligomeric Golgi complex subunit 6	8	0	0
IPR010516	Sin3 associated polypeptide p18	8	0	0

InterPro signature	InterPro description	Shared *Papaipema* genes	*Papaipema sp.4* SSGs	*Papaipema speciosissima* SSGs
IPR010541	Domain of unknown function DUF1115	8	0	0
IPR010613	Pescadillo	8	0	0
IPR010961	Tetrapyrrole biosynthesis 5-aminolevulinic acid synthase	8	0	0
IPR011008	Dimeric alpha-beta barrel	8	0	0
IPR011381	Histone H3-K9 methyltransferase	8	0	0
IPR011488	Translation initiation factor 2, alpha subunit	8	0	0
IPR011941	DNA recombination/repair protein Rad51	8	0	0
IPR011943	HAD-superfamily hydrolase subfamily IIID	8	0	0
IPR011947	FCP1-like phosphatase phosphatase domain	8	0	0
IPR011949	HAD-superfamily hydrolase, subfamily IA, REG-2-like	8	0	0
IPR012259	Dihydrofolate reductase	8	0	0
IPR012313	Zinc finger FCS-type	8	0	0
IPR012445	Autophagy-related protein 1010	8	0	0
IPR012466	NECAP-1 N-terminal	8	0	0
IPR012501	Vps54-likeVacuolar protein sorting-associated protein 54 C-terminal	8	0	0
IPR012502	Wings apart-like, metazoan/plants	8	0	0
IPR012577	NIPSNAP	8	0	0
IPR012715	T-complex protein 1, alpha subunit	8	0	0
IPR012716	T-complex protein 1, beta subunit	8	0	0
IPR012722	T-complex protein 1, zeta subunit	8	0	0

InterPro signature	InterPro description	Shared *Papaipema* genes	*Papaipema sp.4* SSGs	*Papaipema speciosissima* SSGs
IPR012762	Ubiquinone biosynthesis protein COQ9	8	0	0
IPR012918	RTP801-like	8	0	0
IPR012978	Uncharacterised domain NUC173	8	0	0
IPR013015	Laminin IV type B	8	0	0
IPR013134	RAD50 zinc hook	8	0	0
IPR013246	SAGA complex, Sgf11 subunit	8	0	0
IPR013255	Chromosome segregation protein Spc25	8	0	0
IPR013258	Striatin N-terminal	8	0	0
IPR013261	Mitochondrial import inner membrane translocase subunit Tim21	8	0	0
IPR013506	DNA topoisomerase type IIA subunit B domain 2	8	0	0
IPR013528	Hydroxymethylglutaryl-coenzyme A synthase N-terminal	8	0	0
IPR013543	Calcium/calmodulin-dependent protein kinase II association-domain	8	0	0
IPR013549	Domain of unknown function DUF1731	8	0	0
IPR013586	26S proteasome regulatory subunit C-terminal	8	0	0
IPR013718	COQ9	8	0	0
IPR013729	Multiprotein bridging factor 1 N-terminal	8	0	0
IPR013843	Ribosomal protein S4e N-terminal	8	0	0
IPR013846	mRNA capping enzyme C-terminal	8	0	0
IPR013873	Cdc37 C-terminal	8	0	0
IPR013876	TFIIH p62 subunit N-terminal	8	0	0

**InterPro signature**	**InterPro description**	**Shared *Papaipema* genes**	***Papaipema sp.4* SSGs**	***Papaipema speciosissima* SSGs**
IPR013899	Domain of unknown function DUF1771	8	0	0
IPR013919	Peroxisome membrane protein, Pex16	8	0	0
IPR013922	Cyclin PHO80-like	8	0	0
IPR013929	RNA polymerase II-associated protein 1 C-terminal	8	0	0
IPR013934	Small-subunit processome Utp13	8	0	0
IPR014401	Ribosomal protein S6, eukaryotic	8	0	0
IPR014612	Ribonucleases P/MRP protein subunit Rpp20	8	0	0
IPR014731	Electron transfer flavoprotein alpha subunit C-terminal	8	0	0
IPR014806	Ubiquitin-fold modifier-conjugating enzyme 1	8	0	0
IPR014807	Cytochrome oxidase assembly protein 1	8	0	0
IPR014810	Fcf2 pre-rRNA processing	8	0	0
IPR014857	Zinc finger RING-like	8	0	0
IPR014886	La protein RNA-binding domain	8	0	0
IPR014898	Zinc finger C2H2 LYAR-type	8	0	0
IPR014939	CDT1 Geminin-binding domain-like	8	0	0
IPR015212	Regulator of G protein signalling-like domain	8	0	0
IPR015267	Protein phosphatase 4 core regulatory subunit R2	8	0	0
IPR015353	Rubisco LSMT substrate-binding domain	8	0	0
IPR015360	XPC-binding domain	8	0	0
IPR015388	FCP1-like phosphatase C-terminal	8	0	0

InterPro signature	InterPro description	Shared *Papaipema* genes	*Papaipema sp.4* SSGs	*Papaipema speciosissima* SSGs
IPR015623	Actin-related protein 3, metazoa	8	0	0
IPR015782	Dual specificity testis-specific protein kinase 1	8	0	0
IPR015828	NADH dehydrogenase [ubiquinone] 1 alpha subcomplex subunit 10, mitochondrial	8	0	0
IPR015939	Fumarate reductase/succinate dehydrogenase flavoprotein-like C-terminal	8	0	0
IPR016046	Transcription initiation Spt4-like	8	0	0
IPR016072	SKP1 component dimerisation	8	0	0
IPR016272	Lipase, LIPH-type	8	0	0
IPR016275	Glucose-6-phosphatase	8	0	0
IPR016543	Mitochondria fission 1 protein	8	0	0
IPR016656	Transcription initiation factor TFIIE, beta subunit	8	0	0
IPR016680	NADH dehydrogenase [ubiquinone] complex I), alpha subcomplex, subunit 8	8	0	0
IPR016721	Bet3 family	8	0	0
IPR016820	Mediator complex, subunit Med6, metazoa/plant	8	0	0
IPR016897	S-phase kinase-associated protein 1	8	0	0
IPR017074	mRNA capping enzyme, bifunctional	8	0	0
IPR017131	Small ribonucleoprotein associated, SmB/SmN	8	0	0
IPR017250	Histone deacetylase complex, SAP18 subunit	8	0	0
IPR017379	Uncharacterised conserved protein UCP038083, PDZ	8	0	0
IPR017453	Glycine cleavage system H-protein, subgroup	8	0	0
IPR017898	Vacuolar protein sorting-associated VPS28 N-terminal	8	0	0

InterPro signature	InterPro description	Shared *Papaipema* genes	*Papaipema sp.4* SSGs	*Papaipema speciosissima* SSGs
IPR017899	Vacuolar protein sorting-associated VPS28 C-terminal	8	0	0
IPR018409	Na+/H+ exchanger, isoform 8	8	0	0
IPR018613	Protein of unknown function DUF2052, coiled-coil	8	0	0
IPR018713	Domain of unknown function DUF2236	8	0	0
IPR018965	Ubiquitin-activating enzyme E1 C-terminal	8	0	0
IPR018978	Ribosome maturation protein SBDS C-terminal	8	0	0
IPR018996	Inner nuclear membrane protein MAN1	8	0	0
IPR019035	Mediator complex subunit Med12	8	0	0
IPR019130	Macoilin	8	0	0
IPR019151	Proteasome assembly chaperone 2	8	0	0
IPR019153	DDRGK domain containing protein	8	0	0
IPR019173	NADH:ubiquinone oxidoreductase, NDUFB5/SGDH subunit	8	0	0
IPR019317	Brain protein I3	8	0	0
IPR019322	Protein of unknown function DUF2366	8	0	0
IPR019327	Protein of unknown function DUF2373	8	0	0
IPR019333	Integrator complex subunit 3	8	0	0
IPR019340	Histone acetyltransferases subunit 3	8	0	0
IPR019345	Armet protein	8	0	0
IPR019346	Ribosomal protein S32, mitochondrial	8	0	0
IPR019357	Predicted coiled-coil protein DUF2205)	8	0	0

InterPro signature	InterPro description	Shared *Papaipema* genes	*Papaipema sp.4* SSGs	*Papaipema speciosissima* SSGs
IPR019377	NADH-ubiquinone oxidoreductase, subunit 10	8	0	0
IPR019382	Translation initiation factor 3 complex subunit L	8	0	0
IPR019397	Uncharacterised protein family TMEM39	8	0	0
IPR019403	Mediator complex, subunit Med19, metazoa	8	0	0
IPR019409	FMP27 domain of unknown function DUF2405	8	0	0
IPR019468	Adenylosuccinate lyase C-terminal	8	0	0
IPR019481	Transcription factor TFIIIC tau55-related	8	0	0
IPR019497	Sorting nexin protein WASP-binding domain	8	0	0
IPR019716	Ribosomal protein L53, mitochondrial	8	0	0
IPR020234	Mite allergen, group-7	8	0	0
IPR020796	Origin recognition complex, subunit 5	8	0	0
IPR020989	Histone-arginine methyltransferase CARM1 N-terminal	8	0	0
IPR021063	NF-kappa-B essential modulator NEMO N-terminal	8	0	0
IPR021154	H/ACA ribonucleoprotein complex, subunit Gar1	8	0	0
IPR021346	Translation machinery-associated protein 16	8	0	0
IPR021538	Syntaxin-5 N-terminal Sly1p-binding domain	8	0	0
IPR021600	Transcription factor TFIIE alpha subunit C-terminal	8	0	0
IPR021633	Antagonist of EGFR signalling, Argos	8	0	0
IPR021718	Pre-mRNA 3'-end-processing endonuclease polyadenylation factor C-term	8	0	0
IPR021720	Malectin	8	0	0

InterPro signature	InterPro description	Shared *Papaipema* genes	*Papaipema sp.4* SSGs	*Papaipema speciosissima* SSGs
IPR021773	Foie gras liver health family 1	8	0	0
IPR021819	Protein of unknown function DUF3402	8	0	0
IPR021867	25S rRNA adenine2142)-N1))-methyltransferase, Bmt2	8	0	0
IPR021887	Domain of unknown function DUF3498	8	0	0
IPR021910	Protein of unknown function DUF3522	8	0	0
IPR021911	ATPase family AAA domain-containing protein 3 domain of unknown function DUF3523	8	0	0
IPR021990	Mediator complex subunit Med12 LCEWAV-domain	8	0	0
IPR022136	Domain of unknown function DUF3668	8	0	0
IPR022238	Uncharacterised protein family, methyltransferase, Williams-Beuren syndrome	8	0	0
IPR022450	tRNA N6-adenosine threonylcarbamoyltransferase, TsaD	8	0	0
IPR022495	Serine/threonine-protein kinase Bud32	8	0	0
IPR022675	Glucose-6-phosphate dehydrogenase C-terminal	8	0	0
IPR022676	Myristoyl-CoA:protein N-myristoyltransferase N-terminal	8	0	0
IPR022677	Myristoyl-CoA:protein N-myristoyltransferase C-terminal	8	0	0
IPR022771	Wings apart-like protein	8	0	0
IPR022800	Spt4/RpoE2 zinc finger	8	0	0
IPR023260	Cysteine/serine-rich nuclear protein family	8	0	0
IPR023451	Thymidylate synthase/dCMP hydroxymethylase domain	8	0	0
IPR023534	Rof/RNase P-like	8	0	0
IPR023603	Threonine aldolase	8	0	0

InterPro signature	InterPro description	Shared *Papaipema* genes	*Papaipema sp.4* SSGs	*Papaipema speciosissima* SSGs
IPR024054	Translation initiation factor 2 alpha subunit middle domain	8	0	0
IPR024055	Translation initiation factor 2 alpha subunit C-terminal	8	0	0
IPR024111	Peroxisomal targeting signal 1 receptor family	8	0	0
IPR024133	Transmembrane protein 138	8	0	0
IPR024240	Alpha-N-acetylglucosaminidase N-terminal	8	0	0
IPR024576	Ribosomal RNA methyltransferase Spb1 domain of unknown function DUF3381	8	0	0
IPR024598	Domain of unknown function DUF3449	8	0	0
IPR024603	COG complex component COG2 C-terminal	8	0	0
IPR024604	Domain of unknown function DUF3635	8	0	0
IPR024766	Zinc finger RING-H2-type	8	0	0
IPR024785	Transducer of regulated CREB activity C-terminal	8	0	0
IPR024824	Growth arrest and DNA damage-inducible protein GADD45	8	0	0
IPR024872	HEXIM	8	0	0
IPR024881	T-cell immunomodulatory protein	8	0	0
IPR024882	Nucleoporin p58/p45	8	0	0
IPR024958	GRASP55/65 PDZ-like domain	8	0	0
IPR025160	AATF leucine zipper-containing domain	8	0	0
IPR025204	Centromere subunit L	8	0	0
IPR025304	ALIX V-shaped domain	8	0	0
IPR025423	Domain of unknown function DUF4149	8	0	0

InterPro signature	InterPro description	Shared *Papaipema* genes	*Papaipema sp.4* SSGs	*Papaipema speciosissima* SSGs
IPR025712	Nucleoporin Nup54 alpha-helical domain	8	0	0
IPR025717	Histone deacetylase complex subunit SAP30 zinc-finger	8	0	0
IPR025754	TRC8 N-terminal domain	8	0	0
IPR025993	Ceramide glucosyltransferase	8	0	0
IPR026000	Anaphase-promoting complex subunit 5	8	0	0
IPR026170	FAM173 family	8	0	0
IPR026236	Integrator complex subunit 2, metazoa	8	0	0
IPR026554	Frizzled-3, invertebrates	8	0	0
IPR026635	Protein-lysine N-methyltransferase Efm4	8	0	0
IPR026682	Proline-rich AKT1 substrate 1 protein	8	0	0
IPR026721	Transmembrane protein 18	8	0	0
IPR026805	GW182 M domain	8	0	0
IPR026900	Tetratricopeptide repeat protein 7A	8	0	0
IPR026937	Strawberry notch helicase C domain	8	0	0
IPR026957	CSC1-like protein 1	8	0	0
IPR026963	Aprataxin	8	0	0
IPR026966	Neurofascin/L1/NrCAM C-terminal domain	8	0	0
IPR027248	Small nuclear ribonucleoprotein Sm D2	8	0	0
IPR027330	TPX2 central domain	8	0	0
IPR027370	RING-type zinc-finger LisH dimerisation motif	8	0	0

InterPro signature	InterPro description	Shared *Papaipema* genes	*Papaipema sp.4* SSGs	*Papaipema speciosissima* SSGs
IPR027777	Dynactin subunit 6	8	0	0
IPR028088	Helix-turn-helix DNA-binding domain of Spt6	8	0	0
IPR028097	FAM91 C-terminal domain	8	0	0
IPR028098	Glycosyltransferase subfamily 4-like N-terminal domain	8	0	0
IPR028163	HAUS augmin-like complex subunit 6 N-terminal	8	0	0
IPR028171	Codanin-1 C-terminal domain	8	0	0
IPR028184	Transcription cofactor vestigial-like protein 4	8	0	0
IPR028241	RAVE subunit 2/Rogdi	8	0	0
IPR028273	Myocardial zonula adherens protein	8	0	0
IPR028282	WASH complex subunit 7 central domain	8	0	0
IPR028283	WASH complex subunit 7 C-terminal	8	0	0
IPR028363	DNA-directed RNA polymerase, subunit RPB6	8	0	0
IPR028566	Telomere-associated protein Rif1	8	0	0
IPR028650	Sorting nexin Lst-4	8	0	0
IPR028741	Serine/threonine-protein kinase LATS1/ Warts	8	0	0
IPR028759	Cyclin-related protein FAM58	8	0	0
IPR028909	Ribosomal protein L21-like	8	0	0
IPR028923	SAICAR synthetase/ADE2 N-terminal	8	0	0
IPR029040	RNA polymerase subunit RPABC4/transcription elongation factor Spt4	8	0	0
IPR029054	dUTPase-like	8	0	0

**InterPro signature**	**InterPro description**	**Shared *Papaipema* genes**	***Papaipema sp.4* SSGs**	***Papaipema speciosissima* SSGs**
IPR029148	FACT complex subunit Spt16 N-terminal lobe domain	8	0	0
IPR029151	Periplasmic sensor domain-like	8	0	0
IPR029160	CCSMST1 family	8	0	0
IPR029304	A-kinase anchor protein 2 C-terminal domain	8	0	0
IPR029615	ER membrane protein complex subunit 10	8	0	0
IPR029693	Transmembrane protein 35	8	0	0
IPR029875	Rho-associated protein kinase LET-502	8	0	0
IPR030434	Cleft lip and palate transmembrane protein 1-like protein	8	0	0
IPR030435	Liprin-beta-2	8	0	0
IPR030485	Chromodomain-helicase-DNA-binding protein 2	8	0	0
IPR030661	SUMO-activating enzyme subunit SAE2/Uba2	8	0	0
IPR030670	60S acidic ribosomal protein P0	8	0	0
IPR031146	Invertebrate plexin-B	8	0	0
IPR031559	Serine/threonine-protein kinase SMG1	8	0	0
IPR031570	Domain of unknown function DUF4704	8	0	0
IPR031821	SOSS complex subunit C	8	0	0
IPR031926	Transmembrane protein 135 N-terminal domain	8	0	0
IPR031972	Cysteine/serine-rich nuclear protein N-terminal domain	8	0	0
IPR031974	Programmed cell death protein 7	8	0	0
IPR032003	Ribosome-associated complex head domain	8	0	0

InterPro signature	InterPro description	Shared *Papaipema* genes	*Papaipema sp.4* SSGs	*Papaipema speciosissima* SSGs
IPR032040	ELYS beta-propeller domain	8	0	0
IPR032049	E3 ubiquitin-protein ligase Msl2 CXC domain	8	0	0
IPR032054	DNA replication factor Cdt1 C-terminal	8	0	0
IPR032245	RecQ-mediated genome instability protein 2	8	0	0
IPR032448	SMARCC SWIRM-associated domain	8	0	0
IPR032456	CAAX prenyl protease 1 N-terminal	8	0	0
IPR032460	Symplekin/Pta1 N-terminal	8	0	0
IPR032647	Max-like protein X	8	0	0
IPR032808	DoxX family	8	0	0
IPR032870	Alpha-ketoglutarate-dependent dioxygenase alkB homologue 7	8	0	0
IPR033053	Histone transcription regulator 3/CABIN1	8	0	0
IPR003286	RNA-directed DNA polymerase, eukaryota	7	3	3
IPR002104	Integrase catalytic domain	7	1	4
IPR015021	Domain of unknown function DUF1907	7	0	3
IPR001189	Manganese/iron superoxide dismutase	7	1	1
IPR004680	Citrate transporter-like domain	7	2	0
IPR005716	Ribosomal protein S5/S7, eukaryotic/archaeal	7	2	0
IPR010009	Apolipophorin-III	7	0	2
IPR018798	FAM125	7	2	0
IPR021138	60S ribosomal protein L18a/ L20, eukaryotes	7	1	1

InterPro signature	InterPro description	Shared *Papaipema* genes	*Papaipema sp.4* SSGs	*Papaipema speciosissima* SSGs
IPR016250	Tyrosine-protein kinase, Fes/Fps type	7	1	0
IPR026896	Transcription termination and cleavage factor C-terminal domain	7	0	1
IPR000341	Phosphatidylinositol 3-kinase Ras-binding PI3K RBD domain	7	0	1
IPR001197	Ribosomal protein L10e	7	1	0
IPR005711	Ribosomal protein S5, eukaryotic/archaeal	7	0	1
IPR007734	Heparan sulphate 2-O-sulfotransferase	7	0	1
IPR008657	Jumping translocation breakpoint	7	0	1
IPR009097	RNA ligase/cyclic nucleotide phosphodiesterase	7	0	1
IPR000031	Phosphoribosylaminoimidazole carboxylase PurE domain	7	0	0
IPR000091	Huntingtin	7	0	0
IPR000133	ER lumen protein retaining receptor	7	0	0
IPR000711	ATPase, OSCP/delta subunit	7	0	0
IPR000747	Homeodomain engrailed	7	0	0
IPR000778	Cytochrome b245, heavy chain	7	0	0
IPR000806	Rab GDI protein	7	0	0
IPR000892	Ribosomal protein S26e	7	0	0
IPR001050	Syndecan	7	0	0
IPR001075	NIF system FeS cluster assembly NifU C-terminal	7	0	0
IPR001141	Ribosomal protein L27e	7	0	0
IPR001308	Electron transfer flavoprotein, alpha subunit	7	0	0

InterPro signature	InterPro description	Shared *Papaipema* genes	*Papaipema sp.4* SSGs	*Papaipema speciosissima* SSGs
IPR001349	Cytochrome c oxidase, subunit VIa	7	0	0
IPR001614	Myelin proteolipid protein PLP	7	0	0
IPR001616	Alphaherpesvirus alkaline exonuclease	7	0	0
IPR001732	UDP-glucose/GDP-mannose dehydrogenase N-terminal	7	0	0
IPR001780	Ribosomal protein L35A	7	0	0
IPR001848	Ribosomal protein S10	7	0	0
IPR002140	Ribosome maturation protein Sdo1/SBDS	7	0	0
IPR002208	SecY/SEC61-alpha family	7	0	0
IPR002233	Adrenoceptor family	7	0	0
IPR002394	Nicotinic acetylcholine receptor	7	0	0
IPR002474	Carbamoyl-phosphate synthase small subunit N-terminal domain	7	0	0
IPR002671	Ribosomal protein L22e	7	0	0
IPR002778	Signal recognition particle, SRP19 subunit	7	0	0
IPR002804	Archease	7	0	0
IPR002821	Hydantoinase/oxoprolinase	7	0	0
IPR003101	Coactivator CBP KIX domain	7	0	0
IPR003119	Saposin A-type domain	7	0	0
IPR003173	Transcriptional coactivator p15 PC4	7	0	0
IPR003197	Cytochrome b-c1 complex subunit 7	7	0	0
IPR003644	Na-Ca exchanger/integrin-beta4	7	0	0

InterPro signature	InterPro description	Shared *Papaipema* genes	*Papaipema sp.4* SSGs	*Papaipema speciosissima* SSGs
IPR004023	Mago nashi protein	7	0	0
IPR004467	Orotate phosphoribosyl transferase domain	7	0	0
IPR004527	Glutamate-tRNA ligase, bacterial/mitochondrial	7	0	0
IPR004536	Selenophosphate synthetase	7	0	0
IPR004540	Translation elongation factor EFG/EF2	7	0	0
IPR004806	UV excision repair protein Rad23	7	0	0
IPR004910	Yippee/Mis18	7	0	0
IPR004932	Retrieval of early ER protein Rer1	7	0	0
IPR005455	Profilin	7	0	0
IPR005571	RNA polymerase Rpb5 N-terminal	7	0	0
IPR005679	Ribosomal protein S12, bacteria	7	0	0
IPR005725	ATPase, V1 complex, subunit A	7	0	0
IPR005729	Ribosomal protein S10, eukaryotic/archaeal	7	0	0
IPR005873	Density-regulated protein DRP1	7	0	0
IPR005938	AAA ATPase, CDC48 family	7	0	0
IPR005993	GMP reductase	7	0	0
IPR006223	Glycine cleavage system T protein	7	0	0
IPR006249	Aconitase/Iron-responsive element-binding protein 2	7	0	0
IPR006258	Dihydrolipoamide dehydrogenase	7	0	0
IPR006266	UMP-CMP kinase	7	0	0

InterPro signature	InterPro description	Shared *Papaipema* genes	*Papaipema sp.4* SSGs	*Papaipema speciosissima* SSGs
IPR006274	Carbamoyl-phosphate synthase, small subunit	7	0	0
IPR006289	Transcription elongation factor, TFIIS	7	0	0
IPR006368	GDP-mannose 4,6-dehydratase	7	0	0
IPR006426	Asparagine synthase, glutamine-hydrolyzing	7	0	0
IPR006529	U2 snRNP auxilliary factor, large subunit, splicing factor	7	0	0
IPR006548	Splicing factor ELAV/HuD	7	0	0
IPR006639	Presenilin/signal peptide peptidase	7	0	0
IPR006953	Vesicle tethering protein Uso1/P115-like head domain	7	0	0
IPR007075	RNA polymerase Rpb1 domain 6	7	0	0
IPR007281	Mre11 DNA-binding	7	0	0
IPR007309	B-block binding subunit of TFIIIC	7	0	0
IPR007638	Glutaminyl-tRNA synthetase class Ib non-specific RNA-binding domain 2	7	0	0
IPR007639	Glutaminyl-tRNA synthetase class Ib non-specific RNA-binding domain N-terminal	7	0	0
IPR007653	Signal peptidase 22kDa subunit	7	0	0
IPR007722	mRNA decapping protein 2 Box A	7	0	0
IPR007872	Zinc finger DPH-type	7	0	0
IPR008155	Amyloidogenic glycoprotein	7	0	0
IPR008379	Band 4.1 C-terminal	7	0	0
IPR008384	Actin-related protein 2/3 complex subunit 4	7	0	0
IPR008555	Suppressor of IKBKE 1	7	0	0

InterPro signature	InterPro description	Shared *Papaipema* genes	*Papaipema sp.4* SSGs	*Papaipema speciosissima* SSGs
IPR008856	Translocon-associated protein subunit beta	7	0	0
IPR008925	Aminoacyl-tRNA synthetase class I anticodon-binding	7	0	0
IPR009088	Transcription factor IIA beta-barrel	7	0	0
IPR009121	Beta-secretase BACE2	7	0	0
IPR009169	Calreticulin	7	0	0
IPR009374	Eukaryotic translation initiation factor 3 subunit K	7	0	0
IPR009395	Biogenesis of lysosome-related organelles complex 1 subunit 1	7	0	0
IPR009445	Protein of unknown function DUF1077, TMEM85	7	0	0
IPR009447	GWT1	7	0	0
IPR009462	Domain of unknown function DUF1086	7	0	0
IPR009852	T-complex protein 10 C-terminal domain	7	0	0
IPR009866	NADH:ubiquinone oxidoreductase, subunit NDUFB4	7	0	0
IPR010073	Phosphoribosylformylglycinamidine synthase	7	0	0
IPR010097	Malate dehydrogenase, type 1	7	0	0
IPR010109	Citrate synthase, eukaryotic-type	7	0	0
IPR010432	RDD	7	0	0
IPR010439	Calcium-dependent secretion activator	7	0	0
IPR010542	Vertebrate heat shock transcription factor C-terminal domain	7	0	0
IPR010565	Muskelin N-terminal	7	0	0
IPR010666	Zinc finger GRF-type	7	0	0

InterPro signature	InterPro description	Shared *Papaipema* genes	*Papaipema sp.4* SSGs	*Papaipema speciosissima* SSGs
IPR010756	Hepatocellular carcinoma-associated antigen 59	7	0	0
IPR011388	Sphingolipid delta4-desaturase	7	0	0
IPR011555	V-ATPase proteolipid subunit C, eukaryotic	7	0	0
IPR011599	Prefoldin alpha subunit, archaea-type	7	0	0
IPR011763	Acetyl-coenzyme A carboxyltransferase C-terminal	7	0	0
IPR011904	Acetate-CoA ligase	7	0	0
IPR012265	Protein-tyrosine phosphatase, non-receptor type-1/2	7	0	0
IPR012486	N1221-like	7	0	0
IPR012599	Peptidase C1A propeptide	7	0	0
IPR012617	Apoptosis-antagonizing transcription factor C-terminal	7	0	0
IPR012718	T-complex protein 1, epsilon subunit	7	0	0
IPR012721	T-complex protein 1, theta subunit	7	0	0
IPR012954	BP28 C-terminal domain	7	0	0
IPR012957	CHD C-terminal 2	7	0	0
IPR012977	Uncharacterised domain NUC130/133 N-terminal	7	0	0
IPR013122	Polycystin cation channel PKD1/PKD2	7	0	0
IPR013177	Domain of unknown function DUF1713 mitochondria	7	0	0
IPR013295	Myelin and lymphocyte MAL protein	7	0	0
IPR013717	PIG-P	7	0	0
IPR013746	Hydroxymethylglutaryl-coenzyme A synthase C-terminal domain	7	0	0

InterPro signature	InterPro description	Shared *Papaipema* genes	*Papaipema sp.4* SSGs	*Papaipema speciosissima* SSGs
IPR013821	Potassium channel voltage dependent KCNQ C-terminal	7	0	0
IPR013855	Cdc37 N-terminal domain	7	0	0
IPR013947	Mediator complex, subunit Med14	7	0	0
IPR014307	Xanthine dehydrogenase small subunit	7	0	0
IPR014375	Protein kinase C, alpha/beta/gamma types	7	0	0
IPR014877	CRM1 C-terminal domain	7	0	0
IPR015172	MIF4G-like type 1	7	0	0
IPR015256	Translation initiation factor 2 gamma subunit C-terminal	7	0	0
IPR015405	NADH-quinone oxidoreductase chain G C-terminal	7	0	0
IPR016239	Ribosomal protein S6 kinase II	7	0	0
IPR016279	Phosphatidylinositol-4, 5-bisphosphate phosphodiesterase gamma	7	0	0
IPR016304	Cyclophilin-type peptidyl-prolyl cis-trans isomerase E	7	0	0
IPR016391	Coatomer alpha subunit	7	0	0
IPR016565	Proteasome assembly chaperone 1	7	0	0
IPR016641	Nascent polypeptide-associated complex subunit alpha	7	0	0
IPR016686	Ribosome biogenesis factor, NIP7	7	0	0
IPR016848	Ribonuclease P/MRP, p29 subunit	7	0	0
IPR016861	Mitochondrial transcription factor TFB2	7	0	0
IPR017276	Synthesis of cytochrome c oxidase, Sco1/Sco2	7	0	0
IPR017287	Retinal rod rhodopsin-sensitive cGMP 3', 5'-cyclic phosphodiesterase, delta subunit	7	0	0

**InterPro signature**	**InterPro description**	**Shared *Papaipema* genes**	***Papaipema sp.4* SSGs**	***Papaipema speciosissima* SSGs**
IPR017580	2-oxo-4-hydroxy-4-carboxy-5-ureidoimidazoline decarboxylase type 1	7	0	0
IPR017904	ADF/Cofilin/Destrin	7	0	0
IPR017916	Steadiness box SB domain	7	0	0
IPR018611	E3 UFM1-protein ligase 1	7	0	0
IPR018983	U3 small nucleolar RNA-associated protein 15 C-terminal	7	0	0
IPR019082	Neurogenic mastermind-like N-terminal	7	0	0
IPR019181	Anticodon-binding domain	7	0	0
IPR019184	Uncharacterised protein family, transmembrane-17	7	0	0
IPR019314	Protein of unknown function DUF2365	7	0	0
IPR019344	Mitochondrial F1-F0 ATP synthase subunit F, predicted	7	0	0
IPR019489	Clp ATPase C-terminal	7	0	0
IPR019499	Valyl-tRNA synthetase tRNA-binding arm	7	0	0
IPR019533	Peptidase S26	7	0	0
IPR019549	Homeobox engrailed C-terminal	7	0	0
IPR019561	Translocon Sec61/SecY plug domain	7	0	0
IPR019565	Alpha-2-macroglobulin thiol-ester bond-forming	7	0	0
IPR019783	Ribosome maturation protein SBDS N-terminal	7	0	0
IPR019814	Translation initiation factor 3 N-terminal	7	0	0
IPR019985	Ribosomal protein L23	7	0	0
IPR020459	AMP-binding	7	0	0

InterPro signature	InterPro description	Shared *Papaipema* genes	*Papaipema sp.4* SSGs	*Papaipema speciosissima* SSGs
IPR020587	DNA recombination and repair protein RecA monomer-monomer interface	7	0	0
IPR020818	GroES chaperonin family	7	0	0
IPR021157	Cytochrome c1 transmembrane anchor C-terminal	7	0	0
IPR021475	Protein of unknown function DUF3128	7	0	0
IPR021565	Rabenosyn Rab binding domain	7	0	0
IPR021721	Zinc finger CCCH-type TRM13	7	0	0
IPR021786	Pre-mRNA splicing factor component Cdc5p/Cef1	7	0	0
IPR022175	Breast carcinoma amplified sequence 3	7	0	0
IPR022879	V-type ATP synthase regulatory subunit B/beta	7	0	0
IPR022885	NADP)H-quinone oxidoreductase subunit D/H	7	0	0
IPR023201	SecY subunit domain	7	0	0
IPR024626	Kri1-like C-terminal	7	0	0
IPR024721	Snurportin-1 N-terminal	7	0	0
IPR024767	Pre-mRNA-splicing factor 38 C-terminal	7	0	0
IPR025481	Cell morphogenesis protein C-terminal	7	0	0
IPR025877	MobA-like NTP transferase	7	0	0
IPR026298	Blc2 family	7	0	0
IPR027048	Secretion-regulating guanine nucleotide exchange factor	7	0	0
IPR027157	Nuclear cap-binding protein subunit 2	7	0	0
IPR027918	Hydrolethalus syndrome protein 1 C-terminal domain	7	0	0

InterPro signature	InterPro description	Shared *Papaipema* genes	*Papaipema sp.4* SSGs	*Papaipema speciosissima* SSGs
IPR028055	Membrane insertase YidC/Oxa1 C-terminal	7	0	0
IPR028090	JAB domain prokaryotic	7	0	0
IPR028107	Spatacsin C-terminal domain	7	0	0
IPR028927	Mannose-6-phosphate receptor	7	0	0
IPR028938	Remodeling and spacing factor 1	7	0	0
IPR029245	Protein of unknown function DUF4528	7	0	0
IPR029342	ECSIT C-terminal domain	7	0	0
IPR029386	TMEM169 protein	7	0	0
IPR029840	Zinc finger protein Gfi-1/1b	7	0	0
IPR030223	Protein dispatched	7	0	0
IPR030294	Palmitoyl-protein thioesterase 1	7	0	0
IPR030547	DNA repair protein XRCC2	7	0	0
IPR031198	Syndecan, invertebrate	7	0	0
IPR031435	Transmembrane protein TMEM132 N-terminal	7	0	0
IPR031640	Glucose dehydrogenase C-terminal	7	0	0
IPR031660	C-terminal associated domain of TOPRIM	7	0	0
IPR031774	SF3A3 domain	7	0	0
IPR031943	CARMIL C-terminal domain	7	0	0
IPR031996	NVL2 nucleolin binding domain	7	0	0
IPR032277	40S ribosomal protein S4 C-terminal domain	7	0	0

InterPro signature	InterPro description	Shared *Papaipema* genes	*Papaipema sp.4* SSGs	*Papaipema speciosissima* SSGs
IPR032403	Exocyst component Exo84 C-terminal	7	0	0
IPR032410	Mitochondrial ABC-transporter N-terminal five TM domain	7	0	0
IPR032416	Peptidase M24 C-terminal domain	7	0	0
IPR032451	SMARCC C-terminal	7	0	0
IPR032751	Protein fuseless	7	0	0
IPR032799	Xylanase inhibitor C-terminal	7	0	0
IPR032843	Cleavage inducing molecular chaperone Jiv	7	0	0
IPR008906	HAT C-terminal dimerisation domain	6	0	5
IPR031688	Voltage-gated calcium channel subunit alpha C-terminal	6	4	0
IPR000054	Ribosomal protein L31e	6	2	0
IPR001515	Ribosomal protein L32e	6	2	0
IPR005874	Eukaryotic translation initiation factor SUI1	6	2	0
IPR004408	Biotin--acetyl-CoA-carboxylase ligase	6	0	1
IPR005345	PHF5-like	6	0	1
IPR005755	Ribosomal protein L13, eukaryotic/archaeal	6	1	0
IPR006605	G2 nidogen/fibulin G2F	6	0	1
IPR009017	Green fluorescent protein	6	0	1
IPR009818	Ataxin-2 C-terminal	6	1	0
IPR015369	Follistatin/Osteonectin EGF domain	6	1	0
IPR018826	WW-domain-binding protein	6	0	1

**InterPro signature**	**InterPro description**	**Shared *Papaipema* genes**	***Papaipema sp.4* SSGs**	***Papaipema speciosissima* SSGs**
IPR022018	ARF GTPase-activating protein GIT1 C-terminal	6	1	0
IPR026971	Condensin subunit 1/Condensin-2 complex subunit D3	6	1	0
IPR030089	SWI/SNF complex subunit BAF57	6	1	0
IPR000405	Galanin receptor family	6	0	0
IPR000530	Ribosomal protein S12e	6	0	0
IPR000783	RNA polymerase subunit H/Rpb5 C-terminal	6	0	0
IPR001210	Ribosomal protein S17e	6	0	0
IPR001498	Impact N-terminal	6	0	0
IPR001602	Uncharacterised protein family UPF0047	6	0	0
IPR001634	Adenosine receptor	6	0	0
IPR001674	GMP synthase C-terminal	6	0	0
IPR001976	Ribosomal protein S24e	6	0	0
IPR002181	Fibrinogen alpha/beta/gamma chain C-terminal globular domain	6	0	0
IPR002672	Ribosomal protein L28e	6	0	0
IPR002677	Ribosomal protein L32p	6	0	0
IPR002925	Dienelactone hydrolase	6	0	0
IPR003090	Alpha-crystallin N-terminal	6	0	0
IPR003146	Proteinase inhibitor carboxypeptidase propeptide	6	0	0
IPR003210	Signal recognition particle, SRP14 subunit	6	0	0
IPR003417	Core binding factor, beta subunit	6	0	0

**InterPro signature**	**InterPro description**	**Shared *Papaipema* genes**	***Papaipema sp.4* SSGs**	***Papaipema speciosissima* SSGs**
IPR003533	Doublecortin domain	6	0	0
IPR003633	Phospholipase C variant-surface-glycoprotein	6	0	0
IPR004487	Clp protease, ATP-binding subunit ClpX	6	0	0
IPR004519	DNA-directed RNA polymerase, subunit E/RPC8	6	0	0
IPR005148	Arginyl tRNA synthetase N-terminal domain	6	0	0
IPR005550	Kinetochore protein Ndc80	6	0	0
IPR005643	Jun-like transcription factor	6	0	0
IPR006005	Glutamate synthase, NADH/NADPH, small subunit 1	6	0	0
IPR006104	Glycosyl hydrolases family 2 sugar binding domain	6	0	0
IPR006297	Elongation factor 4	6	0	0
IPR006585	Fucolectin tachylectin-4 pentraxin-1	6	0	0
IPR006787	Pinin/SDK	6	0	0
IPR007848	Methyltransferase small domain	6	0	0
IPR007947	CD164-related protein	6	0	0
IPR008154	Amyloidogenic glycoprotein extracellular	6	0	0
IPR009012	GrpE nucleotide exchange factor head	6	0	0
IPR009083	Transcription factor IIA helical	6	0	0
IPR009087	Rab geranylgeranyltransferase alpha subunit insert-domain	6	0	0
IPR009089	DNA double-strand break repair and VJ recombination XRCC4 N-terminal	6	0	0
IPR009263	SERTA domain	6	0	0

InterPro signature	InterPro description	Shared *Papaipema* genes	*Papaipema sp.4* SSGs	*Papaipema speciosissima* SSGs
IPR009755	Colon cancer-associated Mic1-like	6	0	0
IPR010440	Lipopolysaccharide kinase	6	0	0
IPR011031	Multihaem cytochrome	6	0	0
IPR011178	Amyloidogenic glycoprotein copper-binding	6	0	0
IPR011274	Malate dehydrogenase, NAD-dependent, cytosolic	6	0	0
IPR011407	10-formyltetrahydrofolate dehydrogenase	6	0	0
IPR011425	Mediator complex, subunit Med9	6	0	0
IPR011651	Notch ligand N-terminal domain	6	0	0
IPR012008	Serine/threonine-protein phosphatase with EF-hands	6	0	0
IPR012603	RBB1NT	6	0	0
IPR013040	Coatomer gamma subunit appendage Ig-like subdomain	6	0	0
IPR013167	Conserved oligomeric Golgi complex subunit 4	6	0	0
IPR013240	DNA-directed RNA polymerase I, subunit RPA34.5	6	0	0
IPR013913	Nucleoporin Nup153 N-terminal	6	0	0
IPR013992	Adenylate cyclase-associated CAP N-terminal	6	0	0
IPR014309	Xanthine dehydrogenase molybdopterin binding subunit	6	0	0
IPR014347	Tautomerase/MIF superfamily	6	0	0
IPR014381	DNA-directed RNA polymerase RPB5 subunit, eukaryote/virus	6	0	0
IPR014644	Protein arginine N-methyltransferase PRMT7	6	0	0
IPR015195	SLIDE domain	6	0	0

**InterPro signature**	**InterPro description**	**Shared *Papaipema* genes**	***Papaipema sp.4* SSGs**	***Papaipema speciosissima* SSGs**
IPR015224	Talin central	6	0	0
IPR015399	Domain of unknown function DUF1977 DnaJ-like	6	0	0
IPR015408	Zinc finger Mcm10/DnaG-type	6	0	0
IPR015628	Supervillin	6	0	0
IPR015849	Amyloidogenic glycoprotein heparin-binding	6	0	0
IPR016295	Proteasome endopeptidase complex, beta subunit	6	0	0
IPR016315	Protohaem IX farnesyltransferase, mitochondria	6	0	0
IPR016453	Coatomer beta' subunit COPB2)	6	0	0
IPR016488	NADH dehydrogenase [ubiquinone] complex I), alpha subcomplex, subunit 6	6	0	0
IPR016642	26S proteasome regulatory complex, non-ATPase subcomplex, Rpn2/Psmd1 subunit	6	0	0
IPR016899	mRNA guanine-N7))-methyltransferase	6	0	0
IPR017104	Adaptor protein complex AP-2, alpha subunit	6	0	0
IPR017252	Dynein regulator LIS1	6	0	0
IPR017593	Allantoinase	6	0	0
IPR018556	Domain of unknown function DUF2013	6	0	0
IPR019050	FDF domain	6	0	0
IPR019103	Aspartic peptidase DDI1-type	6	0	0
IPR019145	Mediator complex, subunit Med10	6	0	0
IPR019545	DM13 domain	6	0	0
IPR022075	Symplekin C-terminal	6	0	0

**InterPro signature**	**InterPro description**	**Shared *Papaipema* genes**	***Papaipema sp.4* SSGs**	***Papaipema speciosissima* SSGs**
IPR022125	U3 small nucleolar RNA-associated protein 10	6	0	0
IPR022278	Phosphoserine aminotransferase	6	0	0
IPR023208	NADPH-cytochrome P450 reductase	6	0	0
IPR024078	Putative deacetylase LmbE-like domain	6	0	0
IPR024329	Amyloidogenic glycoprotein E2 domain	6	0	0
IPR024605	NADP transhydrogenase alpha subunit C-terminal	6	0	0
IPR024609	Extracellular sulfatase C-terminal	6	0	0
IPR024639	DNA polymerase epsilon subunit B N-terminal	6	0	0
IPR024679	Pre-rRNA-processing protein IPI1/Testis-expressed sequence 10 protein	6	0	0
IPR024761	Transcription factor IIIC 90kDa subunit N-terminal	6	0	0
IPR025532	Glucose-6-phosphate 1-epimerase	6	0	0
IPR025687	C4-type zinc-finger of DNA polymerase delta	6	0	0
IPR025762	DFDF domain	6	0	0
IPR025785	Histone-lysine N-methyltransferase, SETD3	6	0	0
IPR025977	Nuclear condensin complex subunit 3 C-terminal domain	6	0	0
IPR026046	UbiA prenyltransferase domain containing protein 1	6	0	0
IPR026091	Hermansky-Pudlak syndrome 4 protein	6	0	0
IPR026122	Putative helicase MOV-10	6	0	0
IPR026711	Protein male-specific lethal-1	6	0	0
IPR027230	SUMO-conjugating enzyme Ubc9	6	0	0

InterPro signature	InterPro description	Shared *Papaipema* genes	*Papaipema sp.4* SSGs	*Papaipema speciosissima* SSGs
IPR027765	Zinc finger protein PLAG1	6	0	0
IPR028083	Spt6 acidic N-terminal domain	6	0	0
IPR028267	Rapamycin-insensitive companion of mTOR N-terminal domain	6	0	0
IPR028356	UDP-glucose 6-dehydrogenase, eukaryotic type	6	0	0
IPR028419	Adenylyl cyclase-associated protein CAP, fungal type	6	0	0
IPR030260	ATP-binding cassette subfamily B member 10	6	0	0
IPR031488	WD repeat protein mio zinc-ribbon like domain	6	0	0
IPR031544	WD40-like domain containing protein	6	0	0
IPR031781	SF3A2 domain	6	0	0
IPR031940	Domain of unknown function DUF4772	6	0	0
IPR032011	Domain of unknown function DUF4794	6	0	0
IPR032043	E3 ubiquitin-protein ligase Msl2 zinc RING finger	6	0	0
IPR032065	E3 ubiquitin-protein ligase RNF31 UB-associated-like domain	6	0	0
IPR032154	Coatomer subunit gamma C-terminal	6	0	0
IPR032270	AMPK C-terminal adenylate sensor domain	6	0	0
IPR032387	Acetyl-coenzyme A synthetase N-terminal domain	6	0	0
IPR032485	Domain of unknown function DUF5050	6	0	0
IPR032739	Uncharacterised protein family UPF0544	6	0	0
IPR002327	Cytochrome c, class IA/ IB	5	4	0
IPR018061	Retropepsins	5	2	0

InterPro signature	InterPro description	Shared *Papaipema* genes	*Papaipema sp.4* SSGs	*Papaipema speciosissima* SSGs
IPR032368	UCH-binding domain	5	1	1
IPR003127	SoHo domain	5	1	0
IPR010515	Collagenase NC10/endostatin	5	0	1
IPR013189	Glycosyl hydrolase family 32 C-terminal	5	0	1
IPR020189	Translation elongation factor IF5A C-terminal	5	0	1
IPR020602	GTP cyclohydrolase I domain	5	1	0
IPR031542	Cation channel complex component UNC80 N-terminal	5	0	1
IPR000576	LacY/RafB permease family	5	0	0
IPR000586	Somatostatin receptor family	5	0	0
IPR001265	Formin homology family, Cappuccino subfamily	5	0	0
IPR001556	Bombesin receptor-like	5	0	0
IPR001792	Acylphosphatase-like domain	5	0	0
IPR002475	Bcl2-like	5	0	0
IPR004094	Antistasin-like domain	5	0	0
IPR004193	Glycoside hydrolase family 13 N-terminal	5	0	0
IPR005158	Bacterial transcriptional activator domain	5	0	0
IPR005292	Multi drug resistance-associated protein	5	0	0
IPR005568	Ribosomal protein L6 N-terminal	5	0	0
IPR005794	Methionyl-tRNA formyltransferase	5	0	0
IPR005855	Glucosamine-fructose-6-phosphate aminotransferase, isomerising	5	0	0

**InterPro signature**	**InterPro description**	**Shared *Papaipema* genes**	***Papaipema sp.4* SSGs**	***Papaipema speciosissima* SSGs**
IPR006167	DNA repair protein	5	0	0
IPR006407	1,4-alpha-glucan-branching enzyme, GlgB	5	0	0
IPR006550	Polynucleotide kinase 3-phosphatase, metazoan	5	0	0
IPR007698	Alanine dehydrogenase/pyridine nucleotide transhydrogenase NADH-binding domain	5	0	0
IPR007717	Nuclear pore localisation protein NPL4 C-terminal	5	0	0
IPR008040	Hydantoinaseoxoprolinase N-terminal	5	0	0
IPR008290	Phosphatidylinositol 3-kinase, Vps34 type	5	0	0
IPR010465	DRF autoregulatory	5	0	0
IPR010625	CHCH	5	0	0
IPR011281	Succinate dehydrogenase, flavoprotein subunit	5	0	0
IPR013915	Pre-mRNA-splicing factor 19	5	0	0
IPR014006	Succinate dehydrogenase/fumarate reductase, flavoprotein subunit	5	0	0
IPR014536	Sorting nexin 9 subfamily	5	0	0
IPR014608	ATP-citrate synthase	5	0	0
IPR014891	DWNN domain	5	0	0
IPR016257	Ephrin receptor type-A /type-B	5	0	0
IPR016307	Protease inhibitor with pacifastin repeats	5	0	0
IPR016353	Chordin	5	0	0
IPR016555	Phospholipase D1/D2	5	0	0
IPR017153	Glutathione degradosome, DUG1	5	0	0

InterPro signature	InterPro description	Shared *Papaipema* genes	*Papaipema sp.4* SSGs	*Papaipema speciosissima* SSGs
IPR017198	DNA cytosine-5)-methyltransferase 1, metazoa	5	0	0
IPR017213	Peptidase S54, rhomboid, metazoan	5	0	0
IPR019012	RNA cap guanine-N2 methyltransferase	5	0	0
IPR019441	FMP27 GFWDK domain	5	0	0
IPR021419	Mediator complex, subunit Med25, von Willebrand factor type A	5	0	0
IPR021772	Protein of unknown function DUF3337	5	0	0
IPR021846	Domain of unknown function DUF3441	5	0	0
IPR021891	Telomerase ribonucleoprotein complex - RNA-binding domain	5	0	0
IPR021966	Splicing factor SF3a60 binding domain	5	0	0
IPR022141	Plasma membrane calcium transporting P-type ATPase C-terminal	5	0	0
IPR022702	DNA cytosine-5-methyltransferase 1 replication foci domain	5	0	0
IPR024602	Conserved oligomeric Golgi complex subunit 2 N-terminal	5	0	0
IPR024703	Fascin, metazoans	5	0	0
IPR024731	EGF domain	5	0	0
IPR024857	Cappuccino	5	0	0
IPR025261	Domain of unknown function DUF4210	5	0	0
IPR025451	Domain of unknown function DUF4211	5	0	0
IPR026187	Cell death regulator Aven	5	0	0
IPR026193	NADH-ubiquinone oxidoreductase flavoprotein 3	5	0	0
IPR026300	CWF11 family	5	0	0

InterPro signature	InterPro description	Shared *Papaipema* genes	*Papaipema sp.4* SSGs	*Papaipema speciosissima* SSGs
IPR026933	Myelin gene regulatory factor	5	0	0
IPR028761	Protein disabled	5	0	0
IPR029311	Coiled-coil domain-containing protein 50 N-terminal	5	0	0
IPR029341	FAM21/CAPZIP domain	5	0	0
IPR029746	Protein tamozhennic	5	0	0
IPR030403	Disabled homologue 2-interacting protein	5	0	0
IPR030664	Succinate dehydrogenase/fumarate reductase, alpha/adenylylsulphate reductase subunit	5	0	0
IPR030667	NEDD8-activating enzyme E1 regulatory subunit APP-BP1	5	0	0
IPR031075	High mobility group protein HMGB2	5	0	0
IPR031099	BRCA1-associated	5	0	0
IPR031575	E1A-binding protein p400 N-terminal	5	0	0
IPR031907	Germinal-centre associated nuclear protein MCM3AP domain	5	0	0
IPR031916	DNA ligase 3 BRCT domain	5	0	0
IPR032297	Torus domain	5	0	0
IPR032715	Nuclear receptor coactivator 6 putative nucleic acid-binding region	5	0	0
IPR032732	Spermatogenesis-associated protein 6 N-terminal	5	0	0
IPR032733	HAUS augmin-like complex subunit 3 N-terminal	5	0	0
IPR032959	Diamine acetyltransferase 2	5	0	0
IPR033495	Mitochondrial ribonuclease P protein 3	5	0	0
IPR013783	Immunoglobulin-like fold	4	0	44

**InterPro signature**	**InterPro description**	**Shared *Papaipema* genes**	***Papaipema sp.4* SSGs**	***Papaipema speciosissima* SSGs**
IPR025607	Ribosomal protein L5 eukaryotic/L18 archaeal C-terminal	4	0	3
IPR028669	Syntaxin 1	4	1	2
IPR023621	Ribosomal protein L31e domain	4	0	2
IPR031968	Domain of unknown function DUF4782	4	1	1
IPR012606	Ribosomal protein S13/S15 N-terminal	4	0	1
IPR000196	Ribosomal protein L19/L19e domain	4	1	0
IPR002190	MAGE homology domain	4	0	1
IPR005108	HELP	4	0	1
IPR007794	Ribosome receptor lysine/proline rich	4	1	0
IPR021714	Nucleolar pre-ribosomal-associated protein 1 N-terminal	4	0	1
IPR023635	Peptide deformylase	4	1	0
IPR029131	HAUS augmin-like complex subunit 5	4	0	1
IPR032073	Fibronectin type III domain-containing protein 5 C-terminal domain	4	0	1
IPR000178	Translation initiation factor aIF-2, bacterial-like	4	0	0
IPR000241	Putative RNA methylase domain	4	0	0
IPR000751	M-phase inducer phosphatase	4	0	0
IPR000849	Sugar phosphate transporter	4	0	0
IPR001134	Netrin domain	4	0	0
IPR001322	Lamin Tail Domain	4	0	0
IPR001662	Translation elongation factor EF1B gamma chain conserved	4	0	0

InterPro signature	InterPro description	Shared *Papaipema* genes	*Papaipema sp.4* SSGs	*Papaipema speciosissima* SSGs
IPR001787	Ribosomal protein L21	4	0	0
IPR001796	Dihydrofolate reductase domain	4	0	0
IPR002449	Retinol binding protein/Purpurin	4	0	0
IPR002951	Atrophin-like	4	0	0
IPR003024	Sodium bicarbonate cotransporter	4	0	0
IPR003105	SRA-YDG	4	0	0
IPR003113	Phosphatidylinositol 3-kinase adaptor-binding PI3K ABD domain	4	0	0
IPR003886	NIDO domain	4	0	0
IPR003894	TAFH/NHR1	4	0	0
IPR004398	RNA methyltransferase, RsmD	4	0	0
IPR004607	Phosphoribosylglycinamide formyltransferase	4	0	0
IPR005111	MoeA C-terminal domain IV	4	0	0
IPR005326	Plectin/S10 N-terminal	4	0	0
IPR006103	Glycoside hydrolase family 2 catalytic domain	4	0	0
IPR006769	Coiled-coil domain containing protein 109 C-terminal	4	0	0
IPR006884	Fzo/mitofusin HR2 domain	4	0	0
IPR008288	Poly [ADP-ribose] polymerase	4	0	0
IPR008332	Methylguanine DNA methyltransferase ribonuclease-like domain	4	0	0
IPR008580	PPPDE putative peptidase domain	4	0	0
IPR008993	Tissue inhibitor of metalloproteinases-like OB-fold	4	0	0

InterPro signature	InterPro description	Shared *Papaipema* genes	*Papaipema sp.4* SSGs	*Papaipema speciosissima* SSGs
IPR009044	ssDNA-binding transcriptional regulator	4	0	0
IPR009738	BAT2 N-terminal	4	0	0
IPR010598	D-glucuronyl C5-epimerase	4	0	0
IPR011026	Wiscott-Aldrich syndrome protein C-terminal	4	0	0
IPR011678	Domain of unknown function DUF1620	4	0	0
IPR012270	CCR4-NOT complex, subunit 3/ 5	4	0	0
IPR012429	Protein of unknown function DUF1624	4	0	0
IPR012582	NUC194	4	0	0
IPR012984	PROCT domain	4	0	0
IPR013123	RNA 2-O ribose methyltransferase substrate binding	4	0	0
IPR013244	Sec39 domain	4	0	0
IPR013342	Mandelate racemase/muconate lactonizing enzyme C-terminal	4	0	0
IPR013653	FR47-like	4	0	0
IPR013740	Redoxin	4	0	0
IPR014048	Methylated-DNA-[protein]-cysteine S-methyltransferase DNA binding	4	0	0
IPR015088	Zinc finger DNA-directed DNA polymerase family B alpha	4	0	0
IPR015194	ISWI HAND domain	4	0	0
IPR015260	Syntaxin 6 N-terminal	4	0	0
IPR015876	Acyl-CoA desaturase	4	0	0
IPR016314	Cell division protein Cdc6/18	4	0	0

InterPro signature	InterPro description	Shared *Papaipema* genes	*Papaipema sp.4* SSGs	*Papaipema speciosissima* SSGs
IPR016558	DNA primase, large subunit, eukaryotic	4	0	0
IPR016563	Nuclear protein localization protein 4	4	0	0
IPR016651	Leucine carboxyl methyltransferase 1, LCMT1	4	0	0
IPR016652	Ubiquitinyl hydrolase	4	0	0
IPR017350	Peptidase C14A, interleukin-1 beta convertase type	4	0	0
IPR018574	Structure-specific endonuclease subunit Slx4	4	0	0
IPR018993	FGFR1 oncogene partner FOP N-terminal dimerisation domain	4	0	0
IPR019336	Intimal thickness related receptor IRP	4	0	0
IPR019381	Phosphofurin acidic cluster sorting protein 1	4	0	0
IPR019543	Beta-amyloid precursor protein C-terminal	4	0	0
IPR019547	B domain of TMEM189 localisation domain	4	0	0
IPR019607	Putative zinc-finger domain	4	0	0
IPR020381	Proteinase inhibitor I25 cystatin conserved region	4	0	0
IPR020941	Suppressor of fused-like domain	4	0	0
IPR021392	Focadhesin	4	0	0
IPR021536	DNA ligase IV domain	4	0	0
IPR022707	Domain of unknown function DUF3535	4	0	0
IPR023572	Archease domain	4	0	0
IPR023575	Ribosomal protein S19 superfamily	4	0	0
IPR024072	Dihydrofolate reductase-like domain	4	0	0

InterPro signature	InterPro description	Shared *Papaipema* genes	*Papaipema sp.4* SSGs	*Papaipema speciosissima* SSGs
IPR024298	Ancestral coatomer element 1 Sec16/Sec31	4	0	0
IPR024804	G-protein-signalling modulator 1	4	0	0
IPR024880	COPII coat assembly protein, Sec16	4	0	0
IPR024926	Nucleolar GTP-binding protein 1	4	0	0
IPR025761	FFD box	4	0	0
IPR025768	TFG box	4	0	0
IPR026015	F1F0 ATP synthase OSCP/delta subunit N-terminal domain	4	0	0
IPR026638	Nuclear receptor coactivator 6	4	0	0
IPR026905	Protein ASX-like PHD domain	4	0	0
IPR027256	P-type ATPase, subfamily IB	4	0	0
IPR028149	Tantalus-like	4	0	0
IPR028297	Wiskott-Aldrich syndrome protein homologue, fungal	4	0	0
IPR028731	Tankyrase-1	4	0	0
IPR029135	Protein phosphatase 1 regulatory subunit 35 C-terminal	4	0	0
IPR029524	Transcription factor GATA-1	4	0	0
IPR029630	Venom acid phosphatase Acph-1	4	0	0
IPR029637	Alpha-actinin-4	4	0	0
IPR030428	Clathrin coat assembly protein AP180, vertebrate	4	0	0
IPR030437	Liprin-beta-1	4	0	0
IPR030544	Clathrin interactor 1	4	0	0

**InterPro signature**	**InterPro description**	**Shared *Papaipema* genes**	***Papaipema sp.4* SSGs**	***Papaipema speciosissima* SSGs**
IPR030545	WD repeat-containing protein 62	4	0	0
IPR031642	VPS13 repeated coiled region	4	0	0
IPR031950	116 kDa U5 small nuclear ribonucleoprotein component N-terminal	4	0	0
IPR032035	Folliculin C-terminal	4	0	0
IPR032738	TBC1 domain family member 30 C-terminal	4	0	0
IPR033294	Erlin1/2	4	0	0
IPR013021	Myo-inositol-1-phosphate synthase GAPDH-like	3	2	0
IPR019832	Manganese/iron superoxide dismutase C-terminal	3	1	1
IPR025755	60S ribosomal protein L4 C-terminal domain	3	0	2
IPR002906	Ribosomal protein S27a	3	0	1
IPR013923	Autophagy-related protein 16	3	0	1
IPR019831	Manganese/iron superoxide dismutase N-terminal	3	1	0
IPR022803	Ribosomal protein L5 domain	3	0	1
IPR024783	Transducer of regulated CREB activity N-terminal	3	0	1
IPR024786	CREB-regulated transcription coactivator	3	1	0
IPR031309	Ribosomal protein L5 C-terminal	3	0	1
IPR031310	Ribosomal protein L5 N-terminal	3	0	1
IPR000689	Ubiquinone biosynthesis monooxygenase COQ6	3	0	0
IPR001032	Leghaemoglobin	3	0	0
IPR001090	Ephrin receptor ligand binding domain	3	0	0

InterPro signature	InterPro description	Shared *Papaipema* genes	*Papaipema sp.4* SSGs	*Papaipema speciosissima* SSGs
IPR002004	Polyadenylate-binding protein/Hyperplastic disc protein	3	0	0
IPR002969	Apolipoprotein D	3	0	0
IPR003585	Neurexin/syndecan/glycophorin C	3	0	0
IPR004570	CDP-diacylglycerol--glycerol-3-phosphate 3-phosphatidyltransferase	3	0	0
IPR005124	Vacuolar H+)-ATPase G subunit	3	0	0
IPR005428	CD36/scavenger receptor class B member 1	3	0	0
IPR005635	Inner centromere protein ARK-binding domain	3	0	0
IPR006880	INO80 complex subunit B-like conserved region	3	0	0
IPR006955	Uso1/p115-like vesicle tethering protein C-terminal	3	0	0
IPR007191	Sec8 exocyst complex component specific domain	3	0	0
IPR007223	Peroxin 13 N-terminal	3	0	0
IPR009018	Signal recognition particle SRP9/SRP14 subunit	3	0	0
IPR009218	Predicted HD phosphohydrolase	3	0	0
IPR009721	O-acyltransferase WSD1 C-terminal	3	0	0
IPR010449	NUMB domain	3	0	0
IPR010513	KEN domain	3	0	0
IPR011665	Brf1 TBP-binding domain	3	0	0
IPR012089	2-thiocytidine tRNA biosynthesis protein, TtcA	3	0	0
IPR012220	Glutamate synthase, eukaryotic	3	0	0
IPR012717	T-complex protein 1, delta subunit	3	0	0

InterPro signature	InterPro description	Shared *Papaipema* genes	*Papaipema sp.4* SSGs	*Papaipema speciosissima* SSGs
IPR012725	Chaperone DnaK	3	0	0
IPR013242	Retroviral aspartyl protease	3	0	0
IPR013257	SRI Set2 Rpb1 interacting	3	0	0
IPR015121	DNA fragmentation factor 45kDa middle domain	3	0	0
IPR015123	Bcr-Abl oncoprotein oligomerisation	3	0	0
IPR015142	Smac/DIABLO protein	3	0	0
IPR015151	Beta-adaptin appendage C-terminal subdomain	3	0	0
IPR015412	Autophagy-related C-terminal	3	0	0
IPR016017	GDNF/GAS1	3	0	0
IPR016266	DNA polymerase epsilon, subunit B	3	0	0
IPR016280	Phosphatidylinositol-4, 5-bisphosphate phosphodiesterase beta	3	0	0
IPR016691	tRNA guanosine-2'-O-methyltransferase, TRM11	3	0	0
IPR017366	Histone lysine-specific demethylase	3	0	0
IPR017432	Distrobrevin	3	0	0
IPR018798	Multivesicular body subunit 12	3	0	0
IPR019387	Uncharacterised domain SAYSvFN	3	0	0
IPR019601	Oxoglutarate/iron-dependent oxygenase C-terminal degradation domain	3	0	0
IPR021097	CPH domain	3	0	0
IPR021843	Protein of unknown function DUF3437	3	0	0
IPR021991	UHRF1 tandem tudor domain	3	0	0

**InterPro signature**	**InterPro description**	**Shared *Papaipema* genes**	***Papaipema sp.4* SSGs**	***Papaipema speciosissima* SSGs**
IPR022091	TATA element modulatory factor 1 TATA binding	3	0	0
IPR022385	Rhs repeat-associated core	3	0	0
IPR024340	Sec16 central conserved domain	3	0	0
IPR024945	Spt5 C-terminal domain	3	0	0
IPR024963	MAP6/FAM154	3	0	0
IPR025870	Glyoxalase-like domain	3	0	0
IPR025901	Kinesin-associated microtubule-binding domain	3	0	0
IPR026325	Protein of unknown function DUF932	3	0	0
IPR026680	Coiled-coil domain-containing protein 137	3	0	0
IPR026709	Myb/SANT-like DNA-binding domain-containing protein 3	3	0	0
IPR027789	Syndecan/Neurexin domain	3	0	0
IPR027936	Ephrin receptor transmembrane domain	3	0	0
IPR028150	Lustrin cysteine-rich repeated domain	3	0	0
IPR028423	Suppressor of cytokine signalling 7	3	0	0
IPR028502	Plenty of SH3 domains protein 1	3	0	0
IPR028511	Plenty of SH3 domains protein 2	3	0	0
IPR028722	Chromodomain-helicase-DNA-binding protein 3	3	0	0
IPR028730	Zinc finger FYVE domain-containing protein 26	3	0	0
IPR028826	Voltage gated sodium channel, alpha-4 subunit	3	0	0
IPR030026	Glutamate receptor-interacting protein 1	3	0	0

InterPro signature	InterPro description	Shared *Papaipema* genes	*Papaipema sp.4* SSGs	*Papaipema speciosissima* SSGs
IPR031132	Kielin/chordin-like protein	3	0	0
IPR031646	Vacuolar protein sorting-associated protein 13 second N-terminal domain	3	0	0
IPR031701	Homeobox protein SIX1 N-terminal SD domain	3	0	0
IPR031831	cGMP-dependent protein kinase N-terminal coiled-coil domain	3	0	0
IPR032452	Sodium/calcium exchanger domain C-terminal extension	3	0	0
IPR032566	Aprataxin C2HE/C2H2/C2HC zinc finger	3	0	0
IPR032770	Domain of unknown function DUF4537	3	0	0
IPR033016	Echinoderm microtubule-associated protein-like 2	3	0	0
IPR013087	Zinc finger C2H2-type/integrase DNA-binding domain	2	0	19
IPR013083	Zinc finger RING/FYVE/PHD-type	2	0	5
IPR025314	Domain of unknown function DUF4219	2	3	2
IPR000749	ATP:guanido phosphotransferase	2	1	0
IPR003560	2,3-dihydro-2,3-dihydroxybenzoate dehydrogenase	2	1	0
IPR015143	L27-1	2	0	1
IPR022708	Serine/threonine-protein kinase C-terminal	2	1	0
IPR025476	Helitron helicase-like domain	2	0	1
IPR026939	At2g23090-like	2	1	0
IPR027699	Vimentin	2	1	0
IPR028030	Domain of unknown function DUF4592	2	1	0
IPR031076	High mobility group protein HMGB1	2	0	1

**InterPro signature**	**InterPro description**	**Shared *Papaipema* genes**	***Papaipema sp.4* SSGs**	***Papaipema speciosissima* SSGs**
IPR000720	Peptidylglycine alpha-hydroxylating monooxygenase/peptidyl-hydroxyglycine alpha-amidating lyase	2	0	0
IPR000770	SAND domain	2	0	0
IPR001087	GDSL lipase/esterase	2	0	0
IPR001139	Glycoside hydrolase family 30	2	0	0
IPR001304	C-type lectin-like	2	0	0
IPR001469	ATPase, F1 complex, delta/epsilon subunit	2	0	0
IPR002038	Osteopontin	2	0	0
IPR003614	Knottin scorpion toxin-like	2	0	0
IPR004413	Aspartyl/glutamyl-tRNAAsn/Gln amidotransferase, B subunit	2	0	0
IPR004499	Proline-tRNA ligase, class IIa, archaeal-type	2	0	0
IPR006194	Glycine-tRNA synthetase, heterodimeric	2	0	0
IPR006519	Ribosomal protein L11, bacterial-type	2	0	0
IPR006552	VWC out	2	0	0
IPR007084	BRICHOS domain	2	0	0
IPR007213	Methyltransferase Ppm1/Ppm2/Tcmp	2	0	0
IPR007886	Alanine dehydrogenase/pyridine nucleotide transhydrogenase N-terminal	2	0	0
IPR008297	Notch	2	0	0
IPR009120	Beta-secretase BACE1	2	0	0
IPR009604	LsmAD domain	2	0	0
IPR010307	Laminin domain II	2	0	0

**InterPro signature**	**InterPro description**	**Shared *Papaipema* genes**	***Papaipema sp.4* SSGs**	***Papaipema speciosissima* SSGs**
IPR010423	Plasmodium ookinete surface Pvs28	2	0	0
IPR010474	Bovine leukaemia virus receptor	2	0	0
IPR010506	DNMT1 DMAP1-binding Domain	2	0	0
IPR010919	SAND domain-like	2	0	0
IPR011939	DNA repair and recombination protein RadB	2	0	0
IPR012233	Protein kinase C	2	0	0
IPR012274	Alpha-crystallin, subunit A	2	0	0
IPR013309	Wnt inhibitory factor WIF)-1	2	0	0
IPR013324	RNA polymerase sigma factor region 3/4	2	0	0
IPR013341	Mandelate racemase/muconate lactonizing enzyme N-terminal domain	2	0	0
IPR013905	Lethal giant larvae Lgl-like C-terminal domain	2	0	0
IPR014815	Phospholipase C-beta C-terminal domain	2	0	0
IPR014978	Glutamine-Leucine-Glutamine QLQ	2	0	0
IPR015008	Rho binding domain	2	0	0
IPR015163	Cdc6 C-terminal domain	2	0	0
IPR015446	Bone morphogenetic protein 1/tolloid-like protein	2	0	0
IPR015503	Cortactin	2	0	0
IPR016432	Ribonuclease P, protein component 4	2	0	0
IPR017071	Transcription elongation factor Spt5	2	0	0
IPR017073	Ubiquitin binding protein, Hrs/VPS27	2	0	0

InterPro signature	InterPro description	Shared *Papaipema* genes	*Papaipema sp.4* SSGs	*Papaipema speciosissima* SSGs
IPR017105	Adaptor protein complex AP-3, delta subunit	2	0	0
IPR018863	Fragile site-associated protein C-terminal	2	0	0
IPR018982	RQC domain	2	0	0
IPR019135	Polycomb protein VEFS-Box	2	0	0
IPR020546	ATPase F1 complex delta/epsilon subunit N-terminal	2	0	0
IPR020547	ATP synthase delta/epsilon subunit C-terminal domain	2	0	0
IPR021110	DNA replication/checkpoint protein	2	0	0
IPR021893	Protein of unknown function DUF3504	2	0	0
IPR022414	ATP:guanido phosphotransferase catalytic domain	2	0	0
IPR022428	Diphthamide synthesis DPH2, archaea	2	0	0
IPR024130	DAP1/DAPL1	2	0	0
IPR024314	Suppressor of fused C-terminal	2	0	0
IPR024928	E3 ubiquitin-protein ligase, SMURF1 type	2	0	0
IPR025719	Myelin gene regulatory factor C-terminal domain 2	2	0	0
IPR025786	Mononegavirus L protein 2-O-ribose methyltransferase	2	0	0
IPR025852	Ataxin 2 SM domain	2	0	0
IPR026104	Zinc finger C2HC domain-containing protein 1C	2	0	0
IPR026165	Cytoskeleton-associated protein 2 family	2	0	0
IPR026255	NADP transhydrogenase, alpha subunit	2	0	0
IPR026504	Meiosis-specific nuclear structural protein 1	2	0	0

InterPro signature	InterPro description	Shared *Papaipema* genes	*Papaipema sp.4* SSGs	*Papaipema speciosissima* SSGs
IPR026732	Centrosomal protein of 135kDa	2	0	0
IPR027030	DNA polymerase subunit gamma-2, mitochondrial	2	0	0
IPR027319	Epsin-2, metazoa	2	0	0
IPR027429	Target of Myb1-like 2	2	0	0
IPR027650	Disheveled-associated activator of morphogenesis 1	2	0	0
IPR028183	Uncharacterised protein family UPF0640	2	0	0
IPR028357	UDP-glucose 6-dehydrogenase, bacterial type	2	0	0
IPR028467	DNA topoisomerase II-beta	2	0	0
IPR028516	Sorbin and SH3 domain-containing protein 2	2	0	0
IPR028563	MICAL-like protein 1	2	0	0
IPR028596	Katanin p60 subunit A1	2	0	0
IPR028634	Clavesin-1	2	0	0
IPR028701	Synaptotagmin-6	2	0	0
IPR028867	Neuropilin and tolloid-like protein 1	2	0	0
IPR029390	AP-3 complex subunit beta C-terminal domain	2	0	0
IPR029544	Calpain-13/Calpain-C	2	0	0
IPR029683	Structural maintenance of chromosomes protein 1A, metazoan	2	0	0
IPR029829	Hepatic leukemia factor	2	0	0
IPR030265	Nesprin-1	2	0	0
IPR030289	Palmitoyltransferase ZDHHC17	2	0	0

InterPro signature	InterPro description	Shared *Papaipema* genes	*Papaipema sp.4* SSGs	*Papaipema speciosissima* SSGs
IPR030327	Sarcoplasmic/endoplasmic reticulum calcium ATPase 2	2	0	0
IPR030507	Bem1/Scd2	2	0	0
IPR030606	Kelch-like protein 5	2	0	0
IPR030668	Spermidine/spermine synthase, eukaryotes	2	0	0
IPR030672	Adenylate cyclase	2	0	0
IPR030724	ELMO domain-containing protein 2	2	0	0
IPR030762	Monocarboxylate transporter 10	2	0	0
IPR031255	Vacuolar protein sorting-associated protein 4A	2	0	0
IPR031630	Coiled-coil domain-containing protein 117	2	0	0
IPR032106	2-oxoglutarate dehydrogenase E1 component N-terminal domain	2	0	0
IPR032171	C-terminal of Roc COR domain	2	0	0
IPR032728	Bardet-Biedl syndrome 1 N-terminal	2	0	0
IPR032916	Cysteine protease ATG4B, metazoa	2	0	0
IPR033173	Translational activator Gcn1	2	0	0
IPR002492	Transposase Tc1-like	1	8	4
IPR015943	WD40/YVTN repeat-like-containing domain	1	0	12
IPR027359	Voltage-dependent channel four helix bundle domain	1	0	4
IPR006588	Peptide N glycanase PAW domain	1	0	2
IPR011989	Armadillo-like helical	1	0	2
IPR013886	PI31 proteasome regulator C-terminal	1	1	0

InterPro signature	InterPro description	Shared *Papaipema* genes	*Papaipema sp.4* SSGs	*Papaipema speciosissima* SSGs
IPR016444	Synaptobrevin/Vesicle-associated membrane protein	1	1	0
IPR017878	TB domain	1	1	0
IPR022023	U1 small nuclear ribonucleoprotein of 70kDa N-terminal	1	0	1
IPR022413	ATP:guanido phosphotransferase N-terminal	1	0	1
IPR024655	Uncharacterised protein family glycosyl hydrolase catalytic domain	1	0	1
IPR026103	Harbinger transposase-derived nuclease, animal	1	0	1
IPR027754	Tubulin polyglutamylase TTLL6	1	1	0
IPR029658	Calcium-binding mitochondrial carrier protein Aralar2	1	0	1
IPR029703	DNA polymerase epsilon catalytic subunit	1	1	0
IPR029903	RmlD-like substrate binding domain	1	0	1
IPR000103	Pyridine nucleotide-disulphide oxidoreductase, class-II	1	0	0
IPR000167	Dehydrin	1	0	0
IPR000218	Ribosomal protein L14P	1	0	0
IPR000760	Inositol monophosphatase-like	1	0	0
IPR000799	Steroidogenic acute regulatory protein-like	1	0	0
IPR001203	Aldehyde ferredoxin oxidoreductase C-terminal	1	0	0
IPR001309	Peptidase C14 ICE catalytic subunit p20	1	0	0
IPR001427	Pancreatic ribonuclease	1	0	0
IPR001469	ATP synthase, F1 complex, delta/epsilon subunit	1	0	0
IPR001482	Type II secretion system protein E	1	0	0

**InterPro signature**	**InterPro description**	**Shared *Papaipema* genes**	***Papaipema sp.4* SSGs**	***Papaipema speciosissima* SSGs**
IPR001526	CD59 antigen	1	0	0
IPR002144	GPCR, family 2, secretin receptor	1	0	0
IPR002273	Lutropin-choriogonadotropic hormone receptor	1	0	0
IPR002345	Lipocalin	1	0	0
IPR002424	Alcohol dehydrogenase, insect-type	1	0	0
IPR002611	IstB-like ATP-binding protein	1	0	0
IPR003285	Yeast eukaryotic release factor	1	0	0
IPR003654	OAR domain	1	0	0
IPR003851	Zinc finger Dof-type	1	0	0
IPR003967	Potassium channel, voltage-dependent, ERG	1	0	0
IPR004140	Exocyst complex component Exo70	1	0	0
IPR004161	Translation elongation factor EFTu-like domain 2	1	0	0
IPR004625	Pyridoxine kinase	1	0	0
IPR005329	Sorting nexin N-terminal	1	0	0
IPR005662	GTP-binding protein Era	1	0	0
IPR005679	Ribosomal protein S12, bacterial-type	1	0	0
IPR006580	Zinc finger TTF-type	1	0	0
IPR007349	Domain of unknown function DUF418	1	0	0
IPR007404	LexA-binding, inner membrane-associated putative hydrolase	1	0	0
IPR007528	RINT-1/Tip20	1	0	0

InterPro signature	InterPro description	Shared *Papaipema* genes	*Papaipema sp.4* SSGs	*Papaipema speciosissima* SSGs
IPR007735	Pecanex	1	0	0
IPR007778	Dictyostelium REP	1	0	0
IPR007900	Transcription initiation factor TFIID component TAF4	1	0	0
IPR007926	Borrelia P83100	1	0	0
IPR008256	Peptidase S1B	1	0	0
IPR008361	Melanin-concentrating hormone receptor	1	0	0
IPR008509	Molybdate-anion transporter	1	0	0
IPR008554	Glutaredoxin-like	1	0	0
IPR009147	Cystic fibrosis transmembrane conductance regulator	1	0	0
IPR010488	Zeta toxin domain	1	0	0
IPR011061	Hirudin/antistatin	1	0	0
IPR011425	Mediator of RNA polymerase II transcription subunit 9	1	0	0
IPR011600	Peptidase C14 caspase domain	1	0	0
IPR012399	Cyclin Y	1	0	0
IPR012577	NIPSNAP	1	0	0
IPR013217	Methyltransferase type 12	1	0	0
IPR013632	DNA recombination and repair protein Rad51-like C-terminal	1	0	0
IPR013697	DNA polymerase epsilon catalytic subunit A C-terminal	1	0	0
IPR013918	Nucleotide exchange factor Fes1	1	0	0
IPR013927	Transcription factor Opi1	1	0	0

InterPro signature	InterPro description	Shared *Papaipema* genes	*Papaipema sp.4* SSGs	*Papaipema speciosissima* SSGs
IPR013967	Rad54 N-terminal	1	0	0
IPR014010	REJ domain	1	0	0
IPR014183	Alcohol dehydrogenase class III	1	0	0
IPR014434	Monothiol glutaredoxin	1	0	0
IPR015266	Domain of unknown function DUF1947	1	0	0
IPR015652	Retinoblastoma-related protein	1	0	0
IPR015676	Tob1/2	1	0	0
IPR015917	Peptidase C14A caspase precursor p45 core	1	0	0
IPR015981	N-acetylglucosamine-6-sulfatase, eukaryotic	1	0	0
IPR016061	Proline-tRNA ligase class II C-terminal	1	0	0
IPR016243	Tyrosine-protein kinase, CSF-1/PDGF receptor family	1	0	0
IPR017194	Transforming growth factor-beta receptor, type II	1	0	0
IPR017328	Sirtuin, class I	1	0	0
IPR017449	Prolyl-tRNA synthetase class II	1	0	0
IPR017904	ADF/Cofilin	1	0	0
IPR018270	Concentrative nucleoside transporter, metazoan/bacterial	1	0	0
IPR018416	Na+/H+ exchanger 9	1	0	0
IPR018629	XK-related protein	1	0	0
IPR018647	Domain of unknown function DUF2075	1	0	0
IPR018833	Domain of unknown function DUF2437	1	0	0

InterPro signature	InterPro description	Shared *Papaipema* genes	*Papaipema sp.4* SSGs	*Papaipema speciosissima* SSGs
IPR019167	Topoisomerase II-associated protein PAT1	1	0	0
IPR019314	BLOC-1-related complex subunit 6	1	0	0
IPR019325	NEDD4/Bsd2	1	0	0
IPR019443	FMP27 C-terminal	1	0	0
IPR020546	ATP synthase F1 complex delta/epsilon subunit N-terminal	1	0	0
IPR021934	Sox C-terminal	1	0	0
IPR022127	Store-operated calcium entry regulator STIMATE/YPL162C	1	0	0
IPR022453	Zinc finger MqsA-type	1	0	0
IPR022694	3-hydroxyacyl-CoA dehydrogenase	1	0	0
IPR022742	Serine aminopeptidase S33	1	0	0
IPR023617	Tyrosine-tRNA ligase, archaeal/eukaryotic-type	1	0	0
IPR023635	Peptide deformylase	1	0	0
IPR024098	Transcription factor EB	1	0	0
IPR024322	Protein of unknown function DUF2682	1	0	0
IPR024508	Protein of unknown function DUF3226	1	0	0
IPR024671	Autophagy-related protein 22-like	1	0	0
IPR025752	High pH protein family	1	0	0
IPR026611	Serine/threonine-protein kinase MRCK alpha	1	0	0
IPR026753	Nuclear apoptosis-inducing factor 1	1	0	0
IPR026836	Adenomatous polyposis coli	1	0	0

**InterPro signature**	**InterPro description**	**Shared *Papaipema* genes**	***Papaipema sp.4* SSGs**	***Papaipema speciosissima* SSGs**
IPR026901	DnaJ homologue subfamily C member 3	1	0	0
IPR026921	Alpha-actinin 1	1	0	0
IPR027088	Mitofusin-1	1	0	0
IPR027228	E3 SUMO-protein ligase PIAS2	1	0	0
IPR027239	Calumenin	1	0	0
IPR028026	Domain of unknown function DUF4502	1	0	0
IPR028312	Transcription factor E2F4	1	0	0
IPR028359	UDP-N-acetyl-D-mannosamine/glucosamine dehydrogenase	1	0	0
IPR028437	Transcription factor GATA-6	1	0	0
IPR028527	Stomatin-3	1	0	0
IPR028636	Clavesin-2	1	0	0
IPR028733	Matrix metalloproteinase-25	1	0	0
IPR028788	Cyclin-dependent kinase 6	1	0	0
IPR028806	RNA-binding protein 6	1	0	0
IPR028895	Cyclin-G2	1	0	0
IPR029307	INTS6/SAGE1/DDX26B/CT45 C-terminal	1	0	0
IPR029428	Mapk-regulated corepressor-interacting protein	1	0	0
IPR029452	Rapamycin-insensitive companion of mTOR domain 5	1	0	0
IPR029511	Dehydrogenase/reductase SDR member 4-like	1	0	0
IPR029731	OKL38 family	1	0	0

**InterPro signature**	**InterPro description**	**Shared *Papaipema* genes**	***Papaipema sp.4* SSGs**	***Papaipema speciosissima* SSGs**
IPR029920	Uridine-cytidine kinase 1	1	0	0
IPR030029	Glutamate receptor-interacting protein 2	1	0	0
IPR030047	Alpha E-catenin	1	0	0
IPR030120	BMP-binding endothelial regulator protein	1	0	0
IPR030301	Ankyrin-B	1	0	0
IPR030352	Cell cycle control protein 50B	1	0	0
IPR030563	Kelch-like ECH-associated protein 1	1	0	0
IPR031252	ATPase family AAA domain-containing protein 2	1	0	0
IPR032513	Alpha-glucosidase domain of unknown function DUF4968	1	0	0
IPR032572	Nuclear migration protein nudC	1	0	0
IPR032682	Condensin complex subunit 1 C-terminal	1	0	0
IPR032802	Peroxiredoxin-like FAM213 family	1	0	0
IPR032927	Erbin	1	0	0
IPR033086	RNA-binding protein Nova-1	1	0	0
IPR033144	Cathepsin D	1	0	0
IPR033196	Exosome complex component Rrp43	1	0	0
IPR033268	Structural maintenance of chromosomes protein 5/6	1	0	0
IPR033275	E3 ubiquitin-protein ligase MARCH-like	1	0	0
IPR033310	Mms4/EME1/EME2	1	0	0
IPR033387	Sentrin-specific protease 1	1	0	0

InterPro signature	InterPro description	Shared *Papaipema* genes	*Papaipema sp.4* SSGs	*Papaipema speciosissima* SSGs
IPR033393	Nuclear receptor-binding factor 2 MIT domain	1	0	0
IPR033452	Glycosyl hydrolase family 30 beta sandwich domain	1	0	0
IPR033453	Glycosyl hydrolase family 30 TIM-barrel domain	1	0	0
IPR033490	Leucine-rich PPR motif-containing protein	1	0	0
IPR033502	UfSP1 peptidase	1	0	0
IPR033620	Coiled-coil-helix-coiled-coil-helix domain-containing protein 1	1	0	0
IPR033851	Mitochondrial intermediate peptidase	1	0	0
IPR033961	Exocyst complex component Exo84	1	0	0
IPR006072	Odorant/pheromone binding protein, Lepidoptera	0	2	0
IPR000898	Indoleamine 2,3-dioxygenase	0	2	0
IPR001108	Peptidase A22A, presenilin	0	1	1
IPR002002	Octopamine receptor	0	2	0
IPR002642	Lysophospholipase catalytic domain	0	0	2
IPR002745	Phosphotransferase KptA/Tpt1	0	0	2
IPR003440	Glycosyl transferase, family 48	0	2	0
IPR004582	Checkpoint protein Rad17/Rad24	0	0	2
IPR014752	Arrestin C-terminal	0	0	3
IPR023211	DNA polymerase palm domain	0	0	3
IPR018862	Eukaryotic translation initiation factor 4E binding protein	0	2	1
IPR003645	Follistatin-like N-terminal	0	3	0

InterPro signature	InterPro description	Shared *Papaipema* genes	*Papaipema sp.4* SSGs	*Papaipema speciosissima* SSGs
IPR006254	Isocitrate lyase	0	3	0
IPR011323	Mss4/translationally controlled tumour-associated TCTP	0	0	3
IPR028553	Neurofibromin	0	2	1
IPR004878	Otopetrin	0	1	2
IPR011237	Peptidase M16 domain	0	0	3
IPR013035	Phosphoenolpyruvate carboxykinase C-terminal	0	0	3
IPR026777	Plasma membrane fusion protein PRM1	0	3	0
IPR000009	Protein phosphatase 2A regulatory subunit PR55	0	1	2
IPR014721	Ribosomal protein S5 domain 2-type fold subgroup	0	0	3
IPR016162	Aldehyde dehydrogenase N-terminal domain	0	0	4
IPR013816	ATP-grasp fold subdomain 2	0	0	4
IPR000454	ATPase, F0 complex, subunit C	0	3	1
IPR016186	C-type lectin-like	0	0	5
IPR001272	Phosphoenolpyruvate carboxykinase, ATP-utilising	0	5	0
IPR015422	Pyridoxal phosphate-dependent transferase major region subdomain 2	0	0	5
IPR000647	CTF transcription factor/nuclear factor 1	0	3	3
IPR001360	Glycoside hydrolase, family 1	0	6	0
IPR013785	Aldolase-type TIM barrel	0	0	7
IPR013762	Integrase-like catalytic domain	0	0	7
IPR004117	Olfactory receptor, Drosophila	0	6	1

InterPro signature	InterPro description	Shared *Papaipema* genes	*Papaipema sp.4* SSGs	*Papaipema speciosissima* SSGs
IPR015688	Elongation Factor 3	0	8	0
IPR011042	Six-bladed beta-propeller TolB-like	0	0	10
IPR004875	DDE superfamily endonuclease, CENP-B-like	0	11	0
IPR023298	P-type ATPase transmembrane domain	0	0	11
IPR013781	Glycoside hydrolase catalytic domain	0	0	16
IPR027291	Glycoside hydrolase 38/57 N-terminal domain	0	0	1
IPR029430	Ciliary BBSome complex subunit 2 N-terminal	0	0	1
IPR029344	Histone RNA hairpin-binding protein RNA-binding domain	0	1	0
IPR001699	Transcription factor, T-box	0	0	1
IPR000654	G-protein alpha subunit, group Q	0	0	1
IPR016021	MIF4G-like domain	0	0	1
IPR026980	Dynein heavy chain 6, axonemal	0	1	0
IPR001694	NADH:ubiquinone oxidoreductase, subunit 1/F420H2 oxidoreductase subunit H	0	0	1
IPR000581	Dihydroxy-acid/6-phosphogluconate dehydratase	0	1	0
IPR000435	Tektin	0	0	1
IPR000568	ATPase, F0 complex, subunit A	0	0	1
IPR001138	Zn2-C6 fungal-type DNA-binding domain	0	0	1
IPR001204	Phosphate transporter	0	1	0
IPR001372	Dynein light chain, type 1/2	0	0	1
IPR001391	Opsin lateral eye type	0	1	0

InterPro signature	InterPro description	Shared *Papaipema* genes	*Papaipema sp.4* SSGs	*Papaipema speciosissima* SSGs
IPR001735	Opsin RH1/RH2	0	0	1
IPR001764	Glycoside hydrolase family 3 N-terminal	0	0	1
IPR002016	Haem peroxidase plant/fungal/bacterial	0	0	1
IPR002159	CD36 antigen	0	1	0
IPR002455	GPCR family 3, GABA-B receptor	0	1	0
IPR002456	GPCR family 3, gamma-aminobutyric acid receptor, type B1	0	1	0
IPR002457	GPCR family 3, gamma-aminobutyric acid receptor, type B2	0	1	0
IPR002527	Picornavirus 2B protein	0	0	1
IPR002559	Transposase IS4-like	0	0	1
IPR002657	Bile acid:sodium symporter/arsenical resistance protein Acr3	0	1	0
IPR002975	Fungal G-protein, alpha subunit	0	1	0
IPR003603	U2A'/phosphoprotein 32 family A C-terminal	0	1	0
IPR003712	Cyanate lyase C-terminal	0	0	1
IPR003949	Potassium channel, voltage-dependent, EAG	0	1	0
IPR004030	Nitric oxide synthase N-terminal	0	0	1
IPR004211	Recombination endonuclease VII	0	1	0
IPR004294	Carotenoid oxygenase	0	1	0
IPR004943	Lepidopteran low molecular weight lipoprotein	0	0	1
IPR005198	Glycoside hydrolase, family 76	0	1	0
IPR006534	P-type ATPase, subfamily IIIA	0	1	0

InterPro signature	InterPro description	Shared *Papaipema* genes	*Papaipema sp.4* SSGs	*Papaipema speciosissima* SSGs
IPR006579	Pre-C2HC domain	0	0	1
IPR006821	Intermediate filament head DNA-binding domain	0	0	1
IPR006861	Hyaluronan/mRNA-binding protein	0	0	1
IPR007021	Domain of unknown function DUF659	0	0	1
IPR007319	Small-subunit processome Utp21	0	0	1
IPR007451	Uncharacterised protein family UPF0274	0	1	0
IPR007648	Mitochondrial ATPase inhibitor	0	1	0
IPR008030	NmrA-like domain	0	0	1
IPR008076	Cyanate hydratase	0	1	0
IPR008386	ATPase, F0 complex, subunit E, mitochondrial	0	0	1
IPR008604	Microtubule-associated protein 7 family	0	1	0
IPR009434	Neuroendocrine secretory protein 55	0	1	0
IPR009571	Membrane protein SUR7/Rim9-like, fungi	0	1	0
IPR009836	Protein of unknown function DUF1399	0	1	0
IPR009959	Polyketide cyclase SnoaL-like domain	0	0	1
IPR011019	KIND domain	0	1	0
IPR011398	Fibrillin	0	0	1
IPR011500	GPCR family 3 nine cysteines domain	0	0	1
IPR011515	Shugoshin C-terminal	0	1	0
IPR011633	Protein of unknown function DUF1602	0	1	0

InterPro signature	InterPro description	Shared *Papaipema* genes	*Papaipema sp.4* SSGs	*Papaipema speciosissima* SSGs
IPR011759	Cytochrome C oxidase subunit II transmembrane domain	0	0	1
IPR012341	Six-hairpin glycosidase	0	0	1
IPR012388	CDK5 and ABL1 enzyme substrate 1/2	0	0	1
IPR012675	Beta-grasp domain	0	0	1
IPR013319	Glycoside hydrolase, family 11/12	0	1	0
IPR013780	Glycosyl hydrolase family 13 all-beta	0	0	1
IPR014352	FERM/acyl-CoA-binding protein 3-helical bundle	0	0	1
IPR014717	Translation elongation factor EF1B/ribosomal protein S6	0	0	1
IPR015428	Synaptotagmin 1	0	0	1
IPR015664	P53-induced protein	0	0	1
IPR015931	Aconitase/3-isopropylmalate dehydratase large subunit alpha/beta/alpha subdomain 1/3	0	0	1
IPR016094	Ribosomal protein L1 2-layer alpha/beta-sandwich	0	0	1
IPR016095	Ribosomal protein L1 3-layer alpha/beta-sandwich	0	0	1
IPR016142	Citrate synthase-like large alpha subdomain	0	0	1
IPR016167	FAD-binding type 2 subdomain 1	0	0	1
IPR016616	Bardet-Biedl syndrome 2 protein	0	1	0
IPR017384	NADH dehydrogenase [ubiquinone] complex I), alpha subcomplex subunit 1	0	0	1
IPR017995	Homeobox protein, antennapedia type	0	1	0
IPR018792	Nuclear phosphoprotein p8, DNA binding	0	1	0
IPR018827	Uncharacterised domain YCR061W C-terminal	0	0	1

InterPro signature	InterPro description	Shared *Papaipema* genes	*Papaipema sp.4* SSGs	*Papaipema speciosissima* SSGs
IPR018969	Xylulose 5-phosphate/Fructose 6-phosphate phosphoketolase C-terminal	0	0	1
IPR018970	Xylulose 5-phosphate/Fructose 6-phosphate phosphoketolase N-terminal	0	0	1
IPR019141	Protein of unknown function DUF2045	0	0	1
IPR019347	Axonemal dynein light chain	0	1	0
IPR019380	Casein kinase substrate phosphoprotein PP28	0	0	1
IPR021031	Hyphally-regulated cell wall protein N-terminal	0	0	1
IPR022048	Envelope fusion protein-like	0	1	0
IPR022173	Protein of unknown function DUF3704	0	0	1
IPR022190	Protein of unknown function DUF3716	0	1	0
IPR022730	DAZ associated protein 2	0	0	1
IPR023029	Ribosomal protein S15P	0	1	0
IPR023192	TGS-like domain	0	0	1
IPR023366	ATP synthase subunit alpha-like domain	0	0	1
IPR023393	START-like domain	0	0	1
IPR024324	Condensin complex subunit 1 N-terminal	0	0	1
IPR024983	CHAT domain	0	1	0
IPR026169	Mitochondria-eating protein	0	0	1
IPR026502	Histone RNA stem-loop-binding protein SLBP1/SLBP2	0	0	1
IPR026798	Dedicator of cytokinesis 6	0	0	1
IPR026892	Glycoside hydrolase family 3	0	1	0

InterPro signature	InterPro description	Shared *Papaipema* genes	*Papaipema sp.4* SSGs	*Papaipema speciosissima* SSGs
IPR026899	13-beta-glucan synthase subunit FKS1-like domain-1	0	0	1
IPR027361	Nicotinic acetylcholine-gated receptor transmembrane domain	0	0	1
IPR027397	Catenin binding domain	0	0	1
IPR027408	PNPase/RNase PH domain	0	0	1
IPR027483	Phosphatidylinositol-4-phosphate 5-kinase C-terminal	0	0	1
IPR028010	Gamma-secretase-activating protein C-terminal domain	0	0	1
IPR028792	Insulin receptor-related protein	0	1	0
IPR029006	ADF-H/Gelsolin-like domain	0	0	1
IPR029199	THRAP3/BCLAF1 family	0	1	0
IPR029274	Protein of unknown function DUF4615	0	1	0
IPR029299	ALMS motif	0	1	0
IPR030083	Melanoma-associated antigen D1	0	1	0
IPR030371	ATP-binding cassette subfamily A member 12	0	1	0
IPR030799	Low-density lipoprotein receptor-related protein 4	0	0	1
IPR031258	Eukaryotic initiation factor 4A-I/II	0	1	0
IPR031828	Myofilin	0	0	1
IPR031970	Anillin N-terminal domain	0	0	1
IPR032345	Protein of unknown function DUF4863	0	1	0
IPR032381	Intracellular hyaluronan-binding protein 4 N-terminal domain	0	0	1
IPR032664	Protein atonal/lin-32	0	1	0

InterPro signature	InterPro description	Shared *Papaipema* genes	*Papaipema sp.4* SSGs	*Papaipema speciosissima* SSGs
IPR032763	Resistance to inhibitors of cholinesterase protein 3 N-terminal	0	1	0
IPR032800	ML-like domain	0	0	1
IPR032984	Hemicentin-2	0	0	1
IPR033171	Caspase-9	0	1	0
IPR028877	50S ribosomal protein L18Ae/60S ribosomal protein L20 and L18a	0	2	0
IPR006840	Glutathione-specific gamma-glutamylcyclotransferase	0	0	2
IPR007702	Janus	0	0	2
IPR007999	Protein of unknown function DUF745	0	2	0
IPR008849	Synaphin	0	2	0
IPR011604	Exonuclease phage-type/RecB C-terminal	0	0	2
IPR012347	Ferritin-related	0	0	2
IPR013879	Protein of unknown function DUF1761	0	2	0
IPR014722	Ribosomal protein L2 domain 2	0	0	2
IPR014753	Arrestin N-terminal	0	0	2
IPR015421	Pyridoxal phosphate-dependent transferase major region subdomain 1	0	0	2
IPR016163	Aldehyde dehydrogenase C-terminal	0	0	2
IPR017849	Alkaline phosphatase-like alpha/beta/alpha	0	0	2
IPR019134	Cactin C-terminal	0	2	0
IPR019186	Nucleolar protein 12	0	1	1
IPR023109	Integrase/recombinase N-terminal	0	0	2

**InterPro signature**	**InterPro description**	**Shared *Papaipema* genes**	***Papaipema sp.4* SSGs**	***Papaipema speciosissima* SSGs**
IPR023173	NADPH-cytochrome p450 reductase FAD-binding alpha-helical domain-3	0	0	2
IPR025452	Domain of unknown function DUF4218	0	1	1
IPR026806	Protein CDV3	0	1	1
IPR026975	Dynein heavy chain 1, axonemal	0	2	0
IPR027410	TCP-1-like chaperonin intermediate domain	0	0	2
IPR028333	Ribosomal protein S17, archaeal/eukaryotic	0	2	0
IPR028559	Filamin A	0	0	2
IPR028833	Tetratricopeptide repeat protein 28	0	0	2
IPR029015	D-3-phosphoglycerate dehydrogenase, type2	0	2	0
IPR029158	Stimulator of interferon genes protein	0	2	0
IPR029901	Protein Spire	0	1	1
IPR032440	40S ribosomal protein S11 N-terminal	0	0	2
IPR033371	Arginine and glutamate-rich protein 1	0	1	1
